# Hydrogen Embrittlement as a Conspicuous Material Challenge—Comprehensive Review and Future Directions

**DOI:** 10.1021/acs.chemrev.3c00624

**Published:** 2024-05-09

**Authors:** Haiyang Yu, Andrés Díaz, Xu Lu, Binhan Sun, Yu Ding, Motomichi Koyama, Jianying He, Xiao Zhou, Abdelali Oudriss, Xavier Feaugas, Zhiliang Zhang

**Affiliations:** a Division of Applied Mechanics, Department of Materials Science and Engineering, Uppsala University, SE-75121 Uppsala, Sweden; b Department of Civil Engineering, Universidad de Burgos, Escuela Politécnica Superior, 09006 Burgos, Spain; c Department of Mechanical and Industrial Engineering, Norwegian University of Science and Technology (NTNU), 7491 Trondheim, Norway; d School of Mechanical and Power Engineering, East China University of Science and Technology, Shanghai 200237, China; e Department of Structural Engineering, Norwegian University of Science and Technology (NTNU), Trondheim 7491, Norway; f Institute for Materials Research, Tohoku University, Sendai 980-8577, Japan; g State Key Laboratory of Metal Matrix Composites, School of Materials Science and Engineering, Shanghai Jiao Tong University, 200240 Shanghai, China; h Laboratoire des Sciences de l’Ingénieur pour l’Environnement, La Rochelle University, CNRS UMR 7356, 17042 La Rochelle, France

## Abstract

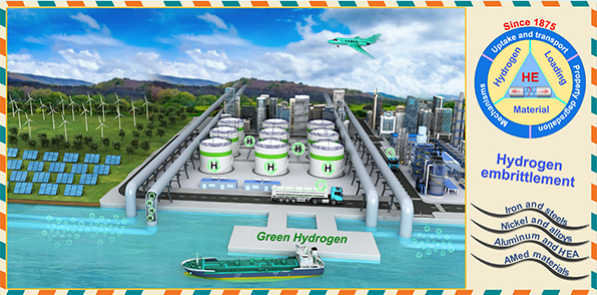

Hydrogen is considered a clean and efficient energy carrier crucial for shaping the net-zero future. Large-scale production, transportation, storage, and use of green hydrogen are expected to be undertaken in the coming decades. As the smallest element in the universe, however, hydrogen can adsorb on, diffuse into, and interact with many metallic materials, degrading their mechanical properties. This multifaceted phenomenon is generically categorized as hydrogen embrittlement (HE). HE is one of the most complex material problems that arises as an outcome of the intricate interplay across specific spatial and temporal scales between the mechanical driving force and the material resistance fingerprinted by the microstructures and subsequently weakened by the presence of hydrogen. Based on recent developments in the field as well as our collective understanding, this Review is devoted to treating HE as a whole and providing a constructive and systematic discussion on hydrogen entry, diffusion, trapping, hydrogen–microstructure interaction mechanisms, and consequences of HE in steels, nickel alloys, and aluminum alloys used for energy transport and storage. HE in emerging material systems, such as high entropy alloys and additively manufactured materials, is also discussed. Priority has been particularly given to these less understood aspects. Combining perspectives of materials chemistry, materials science, mechanics, and artificial intelligence, this Review aspires to present a comprehensive and impartial viewpoint on the existing knowledge and conclude with our forecasts of various paths forward meant to fuel the exploration of future research regarding hydrogen-induced material challenges.

## 1 Introduction

### 1.1 Hydrogen Embrittlement (HE) as a Conspicuous Material Challenge

Green hydrogen (H) is defined as the H produced from water using renewable energy sources. Its production and utilization are free from greenhouse gas emission, which marks a stark contrast to conventional H production. Green H is not just an alternative energy source but a versatile and clean solution integral to achieving a sustainable and low-carbon future. As technological advancements continue and costs decrease, it is poised to become a cornerstone in the global pursuit of decarbonization and sustainability.

Green H functions as a versatile clean energy carrier and storage medium, addressing the intermittency challenge of renewable resources by storing surplus energy and releasing it when needed. As feedstock, green H is revolutionizing a number of industrial processes like refining, ammonia production, and steel manufacturing.^1^ For instance, it has great potential to replace coking coal in steel production, significantly reducing CO_2_ emission. The transportation sector will also benefit, with green H fueling its industries that have suffered from resource exhaustion and pollution, such as trucking, shipping, and aviation.^2^

However, the safe and economical use of green H faces challenges across its lifecycle, encompassing production, transport, and storage.^3^ The viability of H as an energy solution is contingent upon robust structures capable of sustaining long-term, reliable operation throughout the H value chain, catering to current and prospective H energy markets. This compels the development of safe and sustainable H transport and storage systems. For instance, pipelines have been identified as one of the feasible options for regional and inter-regional massive transport and storage of H; refueling stations are demanded for H-driven transportation; high pressure or cryogenic H storage tanks can be viable solutions for maritime and aviation applications. Structural failure of these engineering components can result in a catastrophic consequence and is unacceptable, which necessitates accurate engineering failure assessment of the structures and appropriate material selection.^4^

Metal alloys are perhaps the primary materials for H transport and storage today due to their optimal blend of strength, ductility, and fatigue resistance. Despite the extensive understanding of metallic material degradation, the applicability of conventional engineering failure assessment and material selection strategies for H-related applications remains uncertain. HE is the key threat to the structural integrity of metallic materials in green H applications.^5−7^ Being the smallest atom in the universe, H can adsorb on the surface, diffuse into the bulk, and distribute heterogeneously in and interact with the microstructure of a metal, which can severely undermine the strength, ductility, and fracture toughness.^8^ HE affects nearly all engineering alloys employed in green H transport and storage systems, e.g., ferritic steels,^9,10^ austenitic stainless steels,^11,12^ nickel alloys,^13−15^ aluminum alloys,^16^ and titanium alloys.^17−19^ The impact of H extends beyond fracture characteristics to include elasticity, strain hardening, and fatigue life. An example elaborated in Section 2.4.2 shows that H reduced the ultimate strength of a pipeline steel by over 50% and the ductility over 90%. High-strength alloys are favored in many industries for their ability to improve performance and efficiency. In the pipeline industry, for instance, using high-strength steels allows for higher H gas pressures, enhancing the capacity for H transport and storage. Similarly, the aviation industry benefits from high-strength alloys for H storage tanks, as they provide a beneficial strength-to-weight ratio. However, the issue of HE typically exacerbates as the strength of alloys increases. HE is therefore a universal, conspicuous material challenge to green H applications. Understanding and mitigating the problem will contribute enormously to the safety and cost efficiency of the applications.

**Figure 1:**
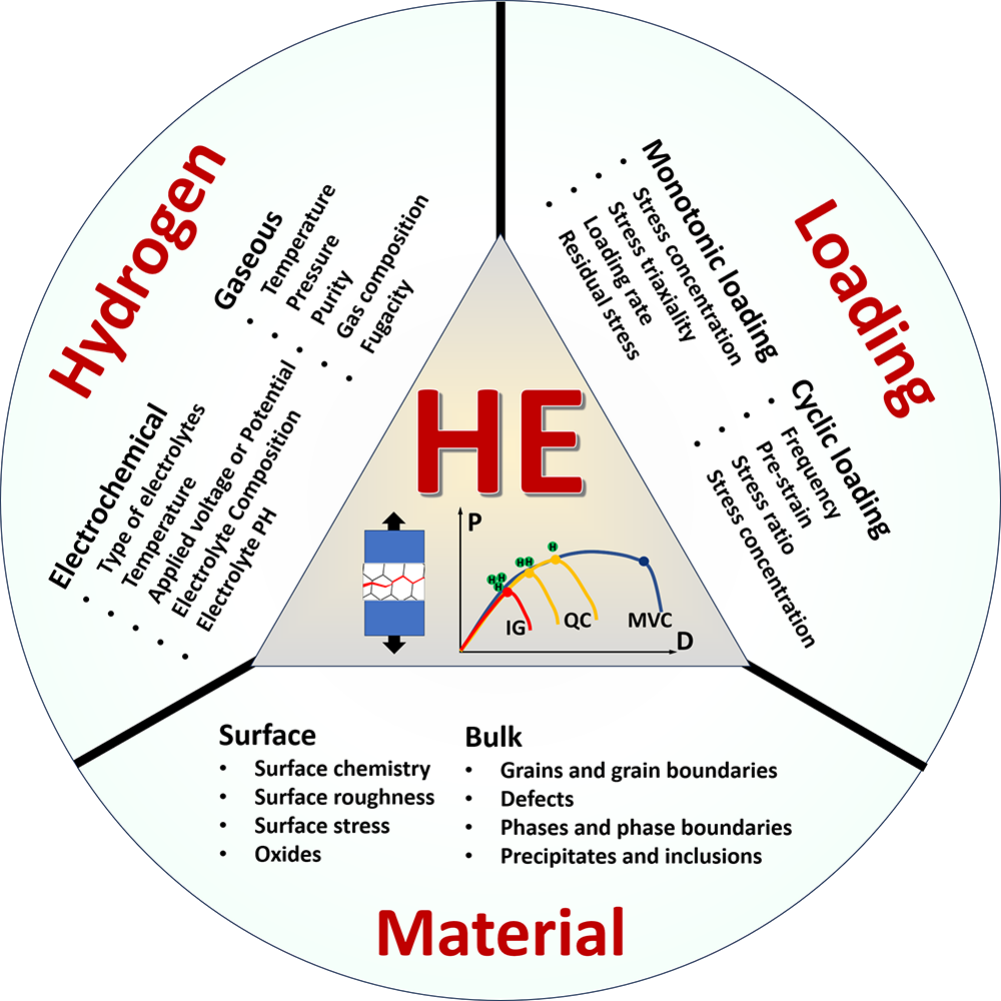
Schematic illustration of the nature of HE and the influencing factors. The triangle in the center shows the reduction of ductility of a tensile specimen with the increase of H concentration, where the fracture mode can change from ductile microvoid coalescence (MVC) failure to quasi-cleavage (QC), and in some cases intergranular (IG) fracture. The three areas surrounding the triangle represent the main categories of typical influencing factors.

“HE is a three-body problem.” This metaphor may not be entirely precise, but it aptly illustrates the extreme complexity of the subject. HE results from the interplay among three primary influencing factors: load, H environment, and susceptible materials, spanning a broad range of spatial and temporal scales. Figure 1 schematically shows the complex nature of HE and the typical influencing factors. Despite a long history of research and tremendous global efforts, HE remains an open and active research area. The difficulty of HE study lies mainly in two aspects. First, measuring the effect of H experimentally is extremely challenging, and precise mapping of H distribution in materials became possible only recently.^10^ Secondly, HE is a multidisciplinary subject that calls for expertise in a number of areas, e.g., electrochemistry, material science, and mechanics, to comprehend.

Through this Review, we endeavor to facilitate a comprehensive understanding of the processes of HE, including H uptake and transport, H–material interaction, as well as the consequences it brings about. This hopefully paves the way for the prediction and mitigation of HE in existing and emerging H energy systems, thus enabling a sustainable future with wide application of green H.

### 1.2 Objective and Scope of the Review

Recently, the growing prominence of green H has propelled a tremendous surge in interest in HE, inviting a multitude of researchers into the field. With the advent of new applications and emergence of new evidence, our understanding of the underlying mechanisms of HE is constantly evolving. A large number of review articles on HE have been published in the last five years. These review articles zoomed into some specific aspects of HE, being theory-specific,^20−22^ method-specific,^7,23−26^ or material-specific.^7,24,27,28^ Due to its long history, diverse compelling theories, and evolving nature, there is a need for a comprehensive review that not only summarizes the existing knowledge but also identifies the knowledge gaps, unresolved issues, and future research directions.

We intended to make the current review **holistic** by covering the whole process of HE, including H entry, redistribution, interactions with the microstructure, and influence on the mechanical property, **extensive** by covering a broad range of temporal and spatial scales, ranging from the atomistic level to the microscopic and ultimately to the macroscopic scale relevant to engineering components, and **objective** by considering and evaluating all prevailing HE theories using common eligibility criteria.

With this Review, we strive to provide beginners in the field with a solid foundation of knowledge. We also aim to present experienced researchers with intriguing updates that can expand their horizons. As a novel feature of this Review, by addressing the key challenges for each aspect of HE and the various emerging approaches, we hope that it can serve as an inspiring and valuable resource for those who are seeking new research directions.

In-depth analyses of existing HE theories from multiple perspectives, including experimental, atomistic, and numerical approaches, have been conducted. This comprehensive evaluation allows us to provide objective and if necessary, critical assessments regarding the applicability of these theories. Additionally, we aim to propose a paradigm for assessing the suitability of an HE theory for a specific material. This paradigm encompasses an evaluation of the foundation of the theories as well as its consistency with both experimental observations and numerical evidence. To limit the scope, we cover the following materials: iron and steels that are of body-centered cubic (*bcc*) structure, steels that are of face-centered cubic (*fcc*) structure, dual and multiple phase steels that possess a combination of *bcc* and *fcc* structures, nickel and its alloys that are of *fcc* structure, aluminum and its alloys of *fcc* structure, and high entropy alloys (HEAs) primarily with *fcc* structure. We also draw a line between HE and other types of H-induced damage, such as H blistering under extreme radiation conditions or brittle fracture in hydride-forming materials.

After the brief introduction, the Review is organized in two primary parts. The first part, Section 2, delves into a comprehensive examination of the current knowledge base, including H uptake and transport, HE mechanisms, and the resulting mechanical property degradation. Specifically, the first part covers the following contents:

- **H uptake and transport**. Knowledge of the precise quantity and location of H atoms both on the surface and inside a material is crucial for understanding HE. We explore the fascinating domain of H entry, transport and redistribution within materials, covering various topics, including H–surface interactions, transport processes, role of trapping, fundamental theories, atomistic approaches, and state-of-the-art experimental H mapping techniques. We also discuss laboratory charging methods and present an overview of the measured H-related properties in steels and nickel alloys.
- **HE mechanisms** concern the interactions of H with material microstructures in the presence of mechanical stress. We take a deep dive into the realm of H–material interactions. At the start, we carefully compare and examine the different theories of HE mechanisms that have gained recognition in the field. We assign equal weight to concepts that have been systematically developed and extensively cited, even if they do not possess specific names. Then we provide a closer look at how each mechanism is linked to specific material groups. As we go further, we introduce the advanced experimental tools, atomistic and continuum modeling approaches used to study these mechanisms together with the main findings. Going beyond a conventional review article, we methodically analyze the mechanisms that might seem unrelated or conflicting, all with the goal of revealing hidden connections and similarities, as well as the key challenges.
- **Influence of H on mechanical properties**. The influence of H extends beyond reducing material ductility; it also impacts a variety of other important mechanical properties. The degradation of mechanical properties due to H is categorized into fracture and non-fracture ones. A well-known and significant question concerning whether H causes hardening or softening is thoroughly discussed. Additionally, the distinct effects of H-induced degradation under cyclic loading are separately addressed. The Review calls for attention to a number of critical unsolved issues of testing methods, emphasizing the lack of a common protocol or standardization to effectively obtain transferrable material and modeling parameters. Furthermore, the status of available models that can be employed to describe the H-induced degradation is discussed, highlighting the need for further development of predictive models.

Building on the thorough examination of the current knowledge base presented in Section 2, the second part of the paper, Section 3, is devoted to exploring **perspectives and future research directions**. Within this section, various critical aspects are addressed, including some thought-provoking questions, knowledge needs in H entry and transport within materials, remaining issues in comprehending the HE mechanisms, as well as the development of effective mitigation strategies. Novel approaches such as machine learning-based methods which offer promising avenues to the solution of the problem are also elaborated.

Finally, the paper ends with a summary comprising overarching statements and suggestions for several topics that merit immediate investigation, as well as outlines of some long-term research directions that hold groundbreaking potentials. The Review concludes that, through gaining a thorough understanding of the underlying mechanisms of the relevant materials, coupled with the development of mechanism-based predictive models, enhancing the transferability of testing methods from laboratory conditions to real-world in-service environments, and leveraging the power of emerging artificial intelligence (AI)-based tools, the conspicuous challenges of HE can be effectively addressed. With these efforts, it is hoped that H energy systems can be safely designed and harnessed for a sustainable future.

## 2 Knowledge Base about HE

HE is a result of the interplay of three factors in general: material microstructures, H environment, and loading. To properly understand HE, we need to know precisely the quantity and location of H in the material to unveil how H interacts with the microstructures and the associated microscopic degradation mechanisms, and how the resultant macroscopic properties can be modified by H. This section, the main body of the Review, is divided into four parts. After a brief introduction of the nature of HE in Section 2.1, Section 2.2 will focus on H uptake and transport. The HE mechanisms will be presented in Section 2.3. Finally, Section 2.4 will be dedicated to the H induced property changes.

### 2.1 Complex Nature of HE

While having a unified theory that adequately explains all aspects of HE is desirable, the realization of such a theory may be impossible. Recognizing this reality can be advantageous, enabling one to approach the field without preconceptions and effectively build a knowledge base that incorporates theories and evidence that may seem conflicting. The complexity permeates virtually every aspect of the exploration of HE.

#### 2.1.1 Controversial Mechanisms

In his seminal work reporting the first experimental evidence of HE in iron and steel in 1875, Johnson^29^ attempted to attribute the macroscopically observed reduction of ductility to the interaction of H with micropores in iron, *“..., so in like manner iron becomes brittle when these pores are filled up by condensed hydrogen gas; and naturally, when the hydrogen is driven out of the molecular interspaces, movement of the molecules on one another is less impeded, and hence the former toughness or elasticity is restored.”* While this theory may not have correctly revealed the underlying processes of HE, it touched upon the interaction between H and the material at a microscopic scale and even indicated a pinning effect of H, which is an important piece of modern HE mechanisms.

Over the past 150 years, more than 6000 papers have been published on this topic,^30^ and numerous HE theories were proposed, debated upon, and proved or disapproved. During the 1970s, researchers made several key breakthroughs in understanding HE, leading to the development of important theories including H-enhanced decohesion (HEDE),^31^ adsorption-induced dislocation emission (AIDE),^32^ and H-enhanced localized plasticity (HELP).^33^ Later, theories like H-enhanced strain-induced vacancies (HESIV)^34^ and Defactant concept^35^ emerged in the 2000s. Post-2010, novel theoretical frameworks were developed thanks to the advancement in experimental and numerical approaches. The main achievements include suggesting and proving a synergistic action of several HE mechanisms, and re-examining some established HE mechanisms with supporting or contradictory atomistic insights. For instance, contradictory to the HELP mechanism, many of these studies claim that H actually suppresses the activity of dislocations, at least of certain types of dislocations. This represents an emerging important genre of opinions, to which a specific term has not been assigned. In this Review, we refer to these as H-suppressed dislocation activities.

The current section is dedicated to elucidating the complexities inherent in HE mechanisms by highlighting the divergences among the previously mentioned theories. Think of this as a teaser, a scene-setter for the in-depth theoretical discussion that unfolds in Section 2.3.2. Here, we’re laying out the puzzle pieces, the varying perspectives, and debates surrounding HE theories to prepare for a more thorough examination subsequently.

The HEDE mechanism focuses on the influence of H on atomistic lattice and microstructural interfaces, such as phase and precipitate interfaces and grain boundary (GB); the HELP mechanism is about the influence on dislocation mobility; the AIDE mechanism is centered around dislocation emission at a free surface; the HESIV mechanism deals with nanovacancies; and the Defactant concept concerns a variety of microstructural defects, such as the multiplication of dislocations and the creation of stacking faults. These theories have covered practically all types of impurities in a metal, which interact with H.

Historically, there had been an endeavor to resolve which type of interaction dominates in HE. There was a famous debate whether the primary role of H is to induce early decohesion or to promote plasticity.^8^ The former, which is essentially the HEDE mechanism, is supported by a number of first principles calculations and can easily rationalize the brittle fracture promoted by H. Advocates of the latter, i.e., the HELP mechanism, argued that HEDE could hardly operate in a perfect lattice due to the extremely high theoretical strength, while HELP was supported by *in situ* experimental observation of H enhanced dislocation mobility. The debate persisted for decades but is now close to being settled thanks to a series of experimental evidence where intensive plasticity was observed beneath H induced intergranular (IG) and transgranular (TG) fracture surfaces.^36−38^ The evidence strongly suggested that both the HEDE and HELP mechanisms operated during the process, and a so-called H-enhanced and plasticity-mediated decohesion mechanism was proposed. This new mechanism elegantly overcomes the limitations of both mechanisms by endowing the HELP mechanism with a link to fracture and lowering the threshold of the HEDE mechanism by considering a material interface instead of a perfect lattice. An alternative view suggests simultaneous, independent operation of HEDE and HELP, with dominance contingent on local H concentration.^39^ This concept was then made more generic by claiming a synergistic action of the HEDE and HELP mechanisms.^40^ Despite not simplifying the study of HE, this debate has significantly contributed to understanding its multifaceted nature.

It becomes increasingly apparent that the validity of each or the synergy of several mechanisms depends on the specific combination of microstructure and H concentration. H interacts practically with all kinds of microstructural defects in a material, and HE is a collective product of these interactions at a macroscopic scale. A specific HE mechanism usually focuses on one of the interactions; therefore, several HE mechanisms can take effect simultaneously in an H-induced failure event. Extra care needs to be taken when dealing with several HE mechanisms about the similar type of microstructural defect. The HELP mechanism, the AIDE mechanism, and the Defactant concept are all closely related to the interaction between H and dislocations. Confusion can arise (and did arise) if the distinction between the mechanisms is not made. For instance, both the HELP mechanism and the Defactant concept operate in H enhanced generation of dislocations, but their roles are often inadequately clarified. The AIDE mechanism is about dislocation emission at a free surface, which leads to suppressed plasticity in the bulk ahead of a crack tip, while the HELP mechanism and the Defactant concept are relevant to dislocation multiplication in the bulk, which leads to enhanced plasticity ahead of a crack tip. Many times, a simplistic term of dislocation emission is employed to describe these distinct processes, leading to a major misunderstanding of the mechanisms. To tackle these complexities and eliminate confusion, a comprehensive review of the HE mechanisms with a clear distinction of their scopes is urgently needed, and this is elaborated in Section 2.3.2.

It should be noted that the existing theories are far from perfect, and they face constant challenges as new evidence of HE is revealed. An early version of the HELP theory stated that H enhances the mobility of an edge dislocation via an elastic shielding effect.^41^ However, a series of recent molecular dynamics (MD) studies suggested that H actually lowers the mobility of a pure edge dislocation,^23,42,43^ and this should apply to both *bcc* and *fcc* materials. It has also been demonstrated that the long-range elastic shielding of H is too weak to influence dislocation mobility in *bcc* material.^44^ The Defactant theory indicates that the activation of a Frank-Read (FR) source of edge type is eased by H since the accommodation of H in dislocation core reduces the line tension of the dislocation.^35,45^ However, H was shown to obstruct the activation of an FR source due to the different tendencies of H segregation to edge and screw parts of a dislocation line.^23^ This is demonstrated via an MD simulation which accounted for the redistribution of H atoms along an elongating dislocation line, during the activation of an FR source. Meanwhile, Huang et al.^46^ very recently simulated the bow-out of a screw dislocation and observed that H facilitated the process. This finding supported H promoted activation of a screw type FR source, which is consistent with the Defactant concept. The AIDE mechanism states that adsorbed H promotes the emission of dislocations from the free surface at a crack tip, and the dislocation emission on suitably inclined slip planes produces crack advance as well as crack opening. This suppresses crack tip blunting which is primarily due to the multiplication of dislocations from sources in the plastic zone ahead of a crack. On the contrary, Song and Curtin^47^ found that H suppressed dislocation emission at the crack tip in iron, which suppresses crack tip blunting and allows more load to be transferred to the crack tip and facilitates advancing the crack. Interestingly, the two seemingly contradictory opinions both point to a suppressed plasticity zone evolution ahead of the crack tip, and a sharper and more easily extending crack due to H, which is consistent with the observation of “embrittlement”. In other words, both the AIDE mechanism and the viewpoint of H suppressed dislocation emission support H suppressed local plasticity in the bulk ahead of the crack tip, which is not fully coincident with, if not contradictory to, the HELP mechanism. These are typical examples of some contradictory views about aforementioned HE mechanisms. Obviously, it is necessary to view the mechanisms with a developmental and even critical perspective, which is elaborated in Section 2.3.2.

#### 2.1.2 Diversity of HE Phenomena

The complexity with HE mechanisms is inherently linked to the complexity with the HE phenomena observed experimentally. Tensile test is one of the most popular experimental approaches to measuring a material’s susceptibility to HE. Without a doubt, H has an influence on the loading curve of a material or more specifically, the load-displacement curve recorded in a tensile test or fracture toughness test. Most prominently and well agreed on, the failure strain or ductility of the specimen is severely reduced by H. In addition, H was also observed to influence other features reflected by a loading curve, including elasticity, strain hardening, and even hardness. There exists much discrepancy in these aspects.

There was a period when the influence of H on the flow behavior as reflected by a tensile loading curve was discussed extensively. A decrease of flow stress due to H is termed H induced plastic softening. In contrast, H can increase the flow stress, which is then termed H induced plastic hardening. Both H induced plastic softening and hardening have been evidenced in tensile experiments on a variety of materials. H-induced plastic softening was observed in a variety of steels,^48−51^ alpha titanium^52,53^ and its alloys,^17,54^ as well as in nickel^55,56^ and its alloys under certain circumstances.^57^ Meanwhile, H-induced plastic hardening was reported on various steels,^58−61^ nickel and its alloy,^62^ and titanium and its alloy.^19,63,64^ A large number of studies also reported negligible influence of H on the tensile loading curve in a number of steels.^12,65,66^ The influence of H is highly material dependent. However, contradictory experimental observations exist even for the same type of material. Zhang et al.^50^ reported H-induced plastic softening in X80 grade steel, while negligible influence of H on the tensile loading curve in X80 grade steel was observed by Moro et al.^65^ and Zhou et al.^66^ Similarly, plastic softening,^49,51^ hardening,^58,60^ and negligible influence^12^ of H were all reported for austenitic stainless steels. Even for pure nickel, contradictory observations of softening^55,56^ and hardening^62^ exist. An in-depth discussion of the influence of H on these non-fracture properties of metals is presented in Section 2.4.1.

Another frequently investigated subject in HE experiments is the morphology of the fracture surface. By comparing the observations of H-charged and H-free specimens, the mechanisms of H induced fracture can be deduced. Although the term “embrittlement” implies a transition of fracture from a ductile mode to a brittle mode, HE is phenomenologically and mechanistically more complex than a typical ductile-to-brittle transition at a low temperature. Low-temperature embrittlement results in a brittle fracture surface which is almost featureless, and this is produced through cleavage across well-established weak crystallographic planes in a metal because plasticity is practically inhibited at a low temperature. A TG fracture surface can occur in HE and appear very differently from a typical ductile fracture surface in the H-free case; however, it seldom follows a proper cleavage plane of the metal, neither does it have a proper “brittle” appearance that is flat and featureless. The surface is often decorated with tearing ridges or small dimples if observed at a high magnification. Such a fracture mode is termed quasi-cleavage, to be distinguished from the cleavage fracture along pre-defined crystallographic planes. In addition, HE can lead to an IG fracture mode in a large number of metallic materials. Such a fracture surface appears smoother compared to a TG one but may still display fine dimples if the magnification is sufficiently high. As to be elaborated in Section 2.4.2, plasticity is a crucial part of HE, while the location of crack initiation is highly influenced by the microstructure of a material, depending on whether the critical condition is first reached at impurities in grain interior or at GBs, TG or IG fracture may take place. Often, a mixture of both fracture types is observed. Further, it should be noted that the appearance of a fracture surface in HE is also highly dependent on the loading condition, for instance, the fracture surface produced in an H-charged fatigue test is distinct from that in a monotonic loading scenario. This is discussed in detail in Section 2.4.3.

#### 2.1.3 Uncertainty of H Residential Sites

The complexity with HE mechanisms and the challenge in predicting HE are partly attributed to the difficulty in determining the residential sites of H in a material. While it is generally accepted that H absorbed in a material can be classified as lattice H at interstitial sites and trapped H bound by microstructural impurities, such as dislocations, precipitates, and GBs, it is still an open question where exactly H resides.

In the lattice of *bcc* iron and *fcc* nickel, there exist two possible interstitial sites for H dissolution, namely tetrahedral interstitial and octahedral interstitial sites. At a dislocation, H can reside in the dislocation elastic stress field or the dislocation core regime, and it can segregate to different segments along a dislocation line, e.g., edge segment and screw segment. At a precipitate, H can reside either at the interface with the matrix or inside the precipitate, and the stress field of an incoherent precipitate can also accommodate a considerable amount of H. There are enormous types of residential sites for H, associated with the various geometric characteristics.

Determination of the exact residential sites of H is key to interpreting the HE phenomena and pinpointing the underlying HE mechanisms. However, due to the small scale of a residential site and the high mobility of H at that scale, it is still impossible to experimentally locate an H atom at room temperature, as discussed in Section 2.2.3. Atomistic calculations remain the most popular option to deduce the location of H in a material, as elaborated in Section 2.2.4.

#### 2.1.4 Challenges in Prediction of HE

Predictive modeling is a powerful tool for engineering failure assessment; however, the nature of complexity makes predictive modeling of HE extremely challenging. A successful modeling practice should first of all be based on appropriate HE mechanism(s), meaning that one needs to determine the operating HE mechanism(s) once the material, environmental, and loading conditions are specified. This requires a deterministic mapping of HE mechanisms to material, H, and loading conditions. Even the boundaries of some prevailing HE mechanisms are not yet drawn clearly, and a lot of controversy and confusion exist. Early attempts to draw such a mapping with applicability boundaries for the HEDE and HELP mechanisms have been reported in a number of steels,^40^ while developing a deterministic mapping and quantitative relationship in other engineering alloys is still a formidable challenge. A mechanism for HE roots in the interaction between H and microstructural features at a nano- and even atomistic scale, while the modeling parameters relevant to engineering practice are formulated at a macroscopic level. Even if the HE mechanism is quantified at the small scale, it is unlikely that the relation can pass through several orders of magnitude in temporal and spatial scales and remain applicable. To summarize, it is inherently difficult to identify the precise HE mechanism for predictive modeling purposes; executing multiscale modeling based on an identified HE mechanism is equally demanding. The current status of mechanism-based HE modeling as well as some future perspectives is presented in Section 2.3.6 and Section 3.4.

### 2.2 H Uptake and Transport

H uptake is an essential stage of HE. Precise knowledge about H uptake is a prerequisite for proper prediction and prevention of the problem. In any case, micromechanical processes involved in embrittlement require a sufficient amount of H atoms to trigger, i.e., a critical H content. The location and concentration of H within the bulk material, either at the interstitial sites, at microstructural defects, or on the metal surface, need to be characterized in order to critically evaluate the contribution of each HE mechanism involved. The aim of the present discussion is to give a detailed overview of how H enters and moves through metals.

In this section, we distinctly address H entry into and H transport within materials, recognizing that these aspects have been traditionally studied using different disciplinary approaches and techniques. The interaction between H and metal surface is explored, covering the fundamentals of H dissociation, adsorption, and absorption processes, highlighting the microstructural and loading factors that influence H uptake. State-of-the-art experimental techniques for mapping H inside a metal are presented, along with a summary regarding the pros and cons of these techniques. This section also describes the atomistic simulations, which serve as a powerful supplement to the experimental techniques, providing insights into H distribution within a metal. The influence of laboratory H charging methods on the results is discussed in a dedicated section, with a focus on discrepancies between laboratory and in-service charging conditions, as well as between gaseous and electrochemical charging conditions. An interesting relationship between gaseous and electrochemical charging is outlined. Finally, an overview of the measured H transport properties in both steels and nickel alloys is provided. A significant feature of this section is that, for each aspect reviewed, we present not only established facts but also a thorough examination of the key challenges, limitations, and unresolved questions.

#### 2.2.1 H–Metal Surface Interactions

In the application of green H, the main source of H leading to HE, is external, where it enters the material during service. For example, pipeline steel uptakes H when in contact with high-pressure H gas, and Ni-based alloys used in subsea energy production and transport absorb H through electrochemical reactions. On the other hand, internal H introduced during manufacturing is not a significant source of H in green H applications. Therefore, the entry of environmental H into a metal is considered the initial step of HE, involving complex surface phenomena and spanning multiple disciplines. The entry of H from the gas phase is rooted in dissociative chemisorption, a subject studied in catalysis research, while H entry driven by cathodic protection potential is studied in the field of electrochemistry, with thermodynamics of solid solutions being essential for both cases. Moreover, stress and strain profoundly influence H entry. We provide an overview of the current understanding of the H community on this highly complex electro-chemo-mechanical problem. Along with a theoretical account, we introduce modeling approaches and important variables, such as coverage and subsurface concentration, that can be applied to quantify H entry. We also discuss factors influencing the analysis outcomes and the limitations of these approaches.

##### 2.2.1.1 Fundamentals of H Dissociation and Adsorption

**Figure 2:**
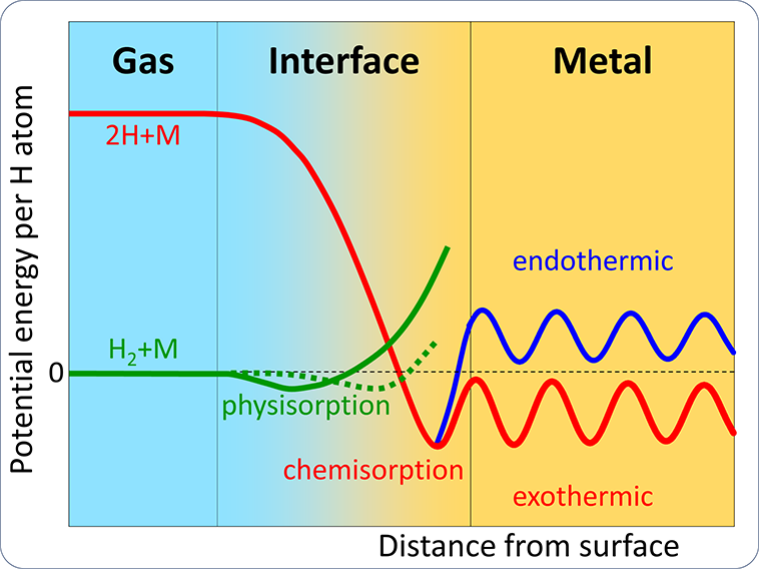
Schematic showing the potential energy landscape for a gaseous H molecule (H_2_) and the corresponding atomic H (2H) approaching a metal surface. An initial physisorbed state can be observed for molecular H. H atoms need to overcome an activation dissociation barrier to be chemisorbed, and finally absorbed H diffuses through the metal lattice. Redrawn based on the input from ref (72). Copyright 2003 Elsevier under [CC BY-NC-ND 3.0 DEED] [https://creativecommons.org/licenses/by-nc-nd/3.0/].

The dissociation of H_2_ into atomic H is probably the rate limiting step in the entry of H, and recombination of H into H_2_ is also an inevitable step during the catalytic reaction, which promotes desorption and further limits the rate of entry. The basic theory of H_2_ dissociation deals with the condition for the breakage of H–H bond, which requires overcoming an energy barrier, i.e., the dissociation energy. The theoretical dissociation energy of H_2_ is reported to be approximately 4.48 eV,^67^ which means that much energy is required to break the bond between the two H atoms. However, in the presence of a surface, the dissociation energy can be lowered due to the interaction between the H_2_ and the surface, as shown in Figure 2; an overview of binding energies of surface H was given by Greely and Mavrikakis.^68^ The stability of adsorption configurations depends on the exothermic or endothermic nature of the surface sites relative to H gas.^69^ A review of H adsorption onto metals was conducted by Pisarev.^70^

It is important to discuss the implications of chemisorption on H entry as the precursor to HE. Adsorption of dissociated H atoms on the metal surface involves electron sharing and the formation of H–M bonds, as indicated by the term chemisorption. Chemisorption can sometimes be indirect, e.g., through molecular precursors.^71^ In contrast, H_2_ is adsorbed to the metal surface by physisorption, a weak interaction that is mainly due to van der Waals forces. In physisorption, the H_2_ is weakly adsorbed on the surface without any significant change in its electronic structure, and it does not make a big contribution to the entry of H. Therefore, the key step for H entry is the dissociative chemisorption from gaseous H or from an equivalent electrochemical environment.

The kinetics of H adsorption and the evolution of coverage *θ* can be simply described by Langmuir’s model.^73^ Langmuir’s reactions assume that the surface is uniform and that the chemisorption of H_2_ is a reversible process consisting of adsorption (*r*_a_) and desorption (*r*_d_) rates:
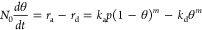
1 where *N*_0_ is the surface density of adsorption sites, *p* is the pressure, *m* is the order of the reaction, and *k*_a_ and *k*_d_ the adsorption and desorption constants, respectively. The adsorption term is proportional to the rate of incidence of the molecules which, according to the kinetic theory of gases, is proportional to the pressure.^74^ This assumption allows one to determine the equilibrium coverage just as a function of the gas pressure, *p*, and a constant *b* = *k*_a_/*k*_d_:
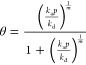
2For physisorbed molecules, it is assumed *m* = 2, whereas *m* = 1 for chemisorbed atoms.^70^ The second-order assumption is based on two vacant elementary sites for molecule adsorption, but this approach can be oversimplified due to the complexity of active sites in metals.^75^

Mechanisms for recombinative desorption of adsorbed H are also under debate:^76^ will two adjacent adsorbed atoms recombine (Langmuir–Hinshelwood mechanism) or will an adsorbed atom interact with molecules in gas phase (Eley–Rideal mechanism)? The surface migration of H atoms and molecules is fast; therefore, the limiting step for desorption is the recombination process.^70^ According to eq 2, equilibrium conditions result in a zero coverage when the H partial pressure is equal to zero. However, kinetic recombination and desorption rates can be non-negligible, and the validity of instantaneous desorption needs to be assessed. For instance, the assumption of instantaneous desorption is common in thermal desorption spectroscopy (TDS) modeling,^77^ but recombination and desorption become the rate limiting steps at low temperatures.^78^ Nevertheless, some authors have enriched H effusion models to circumvent the oversimplification of instantaneous desorption. Guterl et al.^79^ included molecular recombination in a modeling framework for H outgassing and identified different regimes depending on the limiting step. Similarly, Zaika et al.^80^ considered dynamic boundary conditions and explicit recombination coefficients to reinterpret TDS peaks. These kinds of approaches are essential for better evaluating TDS or permeation profiles, and thus for an accurate determination of trapping parameters. TDS experiments and desorption modeling from catalysis characterization^81^ could also be incorporated to better discriminate recombinative desorption from diffusion and trapping phenomena.

##### 2.2.1.2 Characterization of Adsorption

Different techniques are available in the literature to characterize chemisorption of H on metal surfaces. Low energy diffraction (LEED) is a commonly used technique to determine the surface structure of single crystal surfaces and to measure the H–metal bond distance.^82^ Work function measurement can also be interpreted as an indicator of H surface coverage.^83^

A number of other techniques have been used to measure the H adsorption properties on metal surfaces, including Auger electron spectroscopy (AES), ultraviolet photoelectron spectroscopy (UPS), or X-ray photoelectron spectroscopy (XPS).^84−87^ Nuclear reaction analysis (NRA) has also been carried out to measure H depth profiles.^88^ Limitations of indirect techniques can be overcome by advanced mapping methods, such as low energy ion scattering (LEIS) and direct recoil spectroscopy (DRS), which are claimed to be the only methods to directly detect adsorbed H atoms.^89,90^

TDS can be employed to assess surface chemisorption, and it is usually performed after a low temperature exposure to minimize diffusion into the bulk.^91^ The relationship between TDS peaks and heating rates can be used to fit desorption activation energies, and the evolution of coverage can be used to find the expression for the sticking coefficient.^85^ The adsorption activation energy is a measure of the energy required to adsorb an H molecule on a metal surface, and the sticking coefficient is the probability of an H molecule adsorbing onto a metal surface after colliding with it. The adsorption parameters depend on the crystal structure of the metal surface, and a variation in surface characteristics can significantly influence the behavior of the system. However, only indirect information is derived from TDS measurement, but the individual active states cannot be differentiated.^92^

As a matter of fact, the TDS technique has been widely employed by the H community to study the properties of H in the bulk, such as bulk diffusion and trapping,^93^ see Section 2.2.3.2. The logics behind the use of TDS for surface characterization and for bulk measurement of H are totally different, which remains a matter of debate. Surface scientists apply the TDS technique based on the assumption that bulk effects are negligible, whereas the H community neglects surface desorption and applies the same technique. For example, during adsorption characterization, the tails at high temperatures in TDS profiles can be attributed to diffused H in solid solution.^94,95^ The dominance of surface or diffusion-controlled TDS has been discussed in detail by Castro and Meyer.^96^

##### 2.2.1.3 Absorption and Subsurface Concentration

Surface coverage (*θ*) is a variable commonly used by the surface science community; it is also addressed in HE research in several circumstances as a variable governing electrochemical adsorption reactions during H uptake^97^ or for quantifying H segregation at interfaces where fracture is triggered, e.g., at a GB associated with IG fracture.^98^ The coverage *θ* is rarely used to describe uptake of H from gaseous sources. Many theoretical approaches are based on Sievert’s law that correlates H_2_ environment with a concentration in solid solution, but the adsorption process is often overlooked. Regarding H uptake, three different states should be identified: (i) chemisorbed, (ii) sub-surface, and (iii) dissolved H, depending on the depth at which H atoms interact with the host metal lattice. There is evidence supporting the existence and significance of the intermediate state of sub-surface H in a number of alloys,^69,99,100^ but the definition must be linked to an activation energy profile. Reviews discussing subsurface states can be found,^101,102^ with a specific focus on Pd and Ni due to the large amount of theoretical and experimental studies there.

To determine the H concentration in the first layers of lattice sites, two approaches can be considered.

*Thermodynamic theory for solubility of a diatomic gas*:
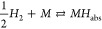
3San Marchi et al.^103^ and Di Leo and Anand^104^ adopted this relationship neglecting the intermediate adsorbed state and assumed equilibrium to derive an extended Sievert’s law that accounts for: (i) the physical meaning of solubility, (ii) the need of fugacity, *f*_*H*_2__ to replace pressure for real gases, and (iii) an uptake condition coherent with steady state distributions. However, both authors assumed low occupancy of lattice sites, *θ_L_* ≪ 1 and *μ*_*H*_2__^0^ = 0. The full expression without this simplification reads:
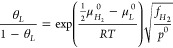
4where *μ*_*H*_2__^0^ and *μ*_*L*_^0^ are the chemical potentials in a reference state for H_2_ and H at lattice sites, respectively, *p*^0^ a reference pressure, *T* the temperature, and *R* the universal gas constant. For *θ_L_* ≪ 1, an expression for the lattice concentration *C_L_* in the subsurface layers can be obtained:
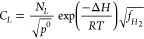
5where *N_L_* is the volume density of lattice sites and defines occupancy, i.e., *θ_L_* = *C_L_*/*N_L_*. The difference between the reference chemical potential of atomic and molecular H, Δ*H* = *μ*_*L*_^0^–1/2*μ*_*H*_2__^0^, is defined as an absorption enthalpy. Absorbed H has been demonstrated to obey this Langmuir’s type function for high-solubility metals, e.g., in Pd,^105^ where the application of traditional Sievert’s law is limited. However, for low solubility metals, the assumption of *θ_L_* ≪ 1 is always valid.

**Figure 3:**
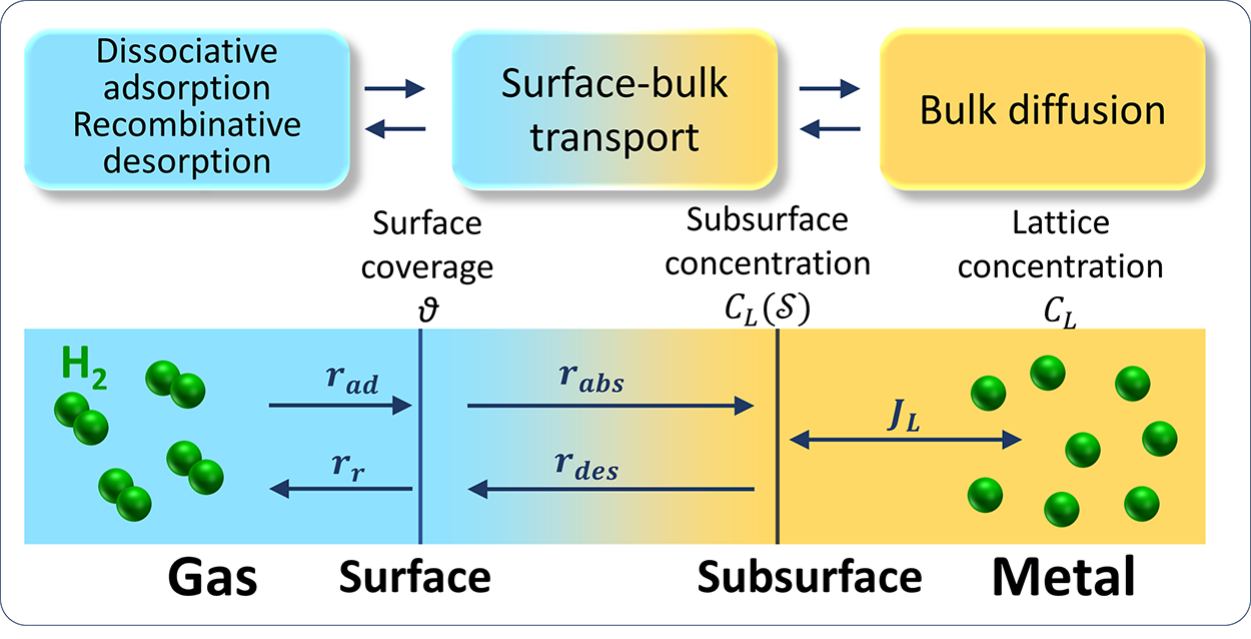
Schematic showing the 2-step kinetic adsorption/absorption of H. Redrawn based on ref (111). Copyright 2019 MDPI under [CC BY 4.0 DEED] [https://creativecommons.org/licenses/by/4.0/].

*Kinetic exchange between adsorbed and absorbed H: MH_ads_ ⇄ MH_abs_*. This approach derives from electrochemical studies on H uptake that postulate an intermediate step that equilibrates dissolved H with H coverage.^106^ The corresponding adsorption–absorption reaction has been widely used to link subsurface concentration and surface coverage with electrochemical constants, following the so-called IPZ fitting procedure^107^ from permeation tests. Turnbull et al.^108,109^ used this to define generalized boundary fluxes for electrochemical H uptake from absorption and desorption rates , *r*_abs_ and *r*_des_, and Martínez-Pañeda et al.^110^ rearranged the terms for dimensional consistency:
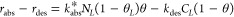
6where *k*_abs_* and *k*_des_ are temperature-dependent constants that have velocity units. However, this reaction is also valid for gaseous uptake since it is independent on the adsorption mechanism. In equilibrium, a Langmuir-type function is again obtained for the H occupancy of lattice sites:
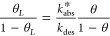
7For *θ_L_* ≪ 1:
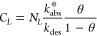
8Both processes can be considered using 2-step kinetic reaction models for gaseous H,^105^ i.e., *H*_2_ ⇄ *MH*_ads_ ⇄ *MH*_abs_. The scheme of this process as a precursor of H lattice diffusion is illustrated in Figure 3. The adsorption process is reversible, meaning that the adsorbed H molecules can detach from the surface and re-enter the gas phase. Similarly, the absorption process is also reversible, meaning that dissolved H atoms can diffuse out of the material and enter the gas phase. At equilibrium, the rates of adsorption/desorption and absorption/desorption are equal, and the concentration of H in the adsorbed and absorbed states reaches a steady-state value. Therefore, the coverage of adsorbed H (*θ*) and subsurface concentration, *C_L_*, are directly related.

The measurement of subsurface concentration can be performed using techniques described above for adsorption characterization, but sometimes subsurface concentration is derived indirectly from permeation experiments.^112,113^ This approach requires some simplifications, e.g., that the diffusion coefficient is solely related to the permeation data, that subsurface concentration is constant over the duration of the test and that the steady state concentration profile is linear. Limitation and validity of these assumptions are detailed in Section 2.2.2.6.

##### 2.2.1.4 H Entry during Electrochemical Charging

As elaborated in Section 2.2.5.3, electrochemical H charging is a widely adopted laboratorial approach to introducing H into the material because of its high accessibility and low cost. During the H evolution reaction (HER),^107^ protons in the aqueous electrolyte are reduced at the metal surface, resulting in the adsorption of H atoms. In a similar way to that previously considered for the gas phase, the extent of electrochemical H adsorption can be quantified by the coverage of H atoms on the metal surface, which is typically expressed as the fraction of available surface sites that are occupied by H atoms.

The coverage evolution of H atoms on the metal surface during HER can be modelled considering adsorption or recombination steps. Constants can be grouped in charging and recombination parameters to define adsorption and recombination rates, *r*_ad_ and *r*_r_:
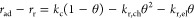
9where the *k*_c_, *k*_r, ch_ and *k*_r, el_ are the adsorption, recombination (charging), and recombination (electron transfer) constants derived from HER theory, i.e., Volmer, Tafel, and Heyrovsky steps in acid media:^110,114−116^
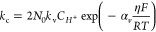
10
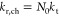
11
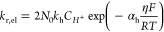
12where *N*_0_ is the surface concentration of adsorption sites and *F* the constant of Faraday. *k*_v_, *k*_t_, and *k*_h_ represent constants for forward Volmer, Tafel, and Heyvrosky reactions, respectively, while *α*_v_ and *α*_h_ are the corresponding charge transfer coefficients. It can be observed that the coverage evolution is thus dependent on the overpotential (*η*), the concentration of protons (*C*_*H*^+^_), and temperature. It must also be noted that for the common overpotential ranges, the backward reactions are neglected.^114^ The balance between adsorption/recombination (*r*_ad_ – *r*_r_) and absorption/desorption (*r*_abs_ – *r*_des_) has been used to derive a simplified procedure to fit the involved constants from permeation test results. In the literature, this procedure is usually named as IPZ fitting, after the work by Iyer, Pickering, and Zamanzadeh.^107^

HER theory was firstly exploited by Bockris and Subramanyan^117^ who proposed a simple relationship between fugacity and overpotential. Liu et al.^114^ defined two overpotential regimes and established a simple fitting method from permeation experiments where only two empirical constants, *A* and *ξ*, govern the relationship for each regime:
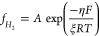
13The validity of this procedure has been confirmed by Koren et al.^118^ by comparing electrochemical and gaseous permeation. The possible equivalence between electrochemical and gaseous permeation is an important topic which is elaborated in Section 2.2.5.3.

##### 2.2.1.5 Factors Influencing H Entry

*Impurity Elements*. The presence of impurity elements has a significant impact on H adsorption onto metal surfaces. Some elements, such as potassium and other alkali metals, can act as adsorption promoters^119^ and therefore are extensively added to catalysts, increasing the overall H adsorption capacity on the metal surface.^120,121^ On the other hand, the presence of adsorption inhibitors, such as oxygen, can significantly reduce the adsorption capacity of many metals, including iron^84^ and nickel.^122^ Molecules of O_2_ compete with H_2_ for adsorption, and therefore, H dissociation is notably precluded.^123^ This effect caused by oxygen has been studied experimentally^124^ and with atomistic calculations.^123^ Mechanical testing in high-pressure H_2_ with O_2_ traces has demonstrated a substantial reduction in HE.^125,126^ Similar inhibiting mechanisms have been found for CO^127^ but with a smaller effect. On the other hand, some density functional theory (DFT) results^123^ indicate that CH_4_ does not compete with H dissociative adsorption, while a mitigation effect of HE was experimentally observed at high CH_4_ contents.^128^ The role of water vapor is not clear since it could act as a mild inhibitor but promote cracking at the same time.^129^

Addition of inhibitor gas traces, especially O_2_, has been proposed as a mitigation method for HE in pipelines,^130^ which has significance for repurposing gas grids.^131^ According to Michler et al.,^132^ the partial pressure of O_2_ is the dominant factor that controls embrittlement. Empirical evidence of gas inhibitors on HE is summarized in several review articles^129,133,134^ which have a consensus that oxygen has a mitigation effect on HE, whereas the influence of other impurities needs further investigation.

*Effects of Surface Defects and Stress State*. The influence of stress on H uptake naturally emerges from a thermodynamic condition considering the equilibrium between the chemical potential of H_2_ and that of absorbed H.^104^ Therefore, expressions of the chemical potential as a function of the stress components need to be derived from theoretical or experimental arguments.^135,136^ The simpler case assumes a linear reduction as a function of hydrostatic stress *σ*_h_ and proportional to *V̅*_H_, the partial volume of H in the host metal. This discussion is elaborated in Section 2.2.2 for the effects of stress and strain on diffusion. Assuming again thermodynamic equilibrium between diatomic H gas and H in the lattice sites, an extended Sievert’s law arises including the influence of the hydrostatic stress *σ*_h_:
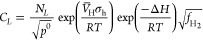
14The relationship between fugacity and pressure for real gases must be considered,^103^ and realistic values for the absorption enthalpy, Δ*H*, must be determined from experiments^137^ or *ab initio* calculations.^138^ Similarly, the hydrostatic stress term is considered to multiply the absorption constant in the adsorption–absorption equilibrium, and therefore, it modifies the relationship between subsurface concentration and coverage:
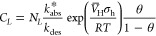
15The stress enhanced H uptake has been assessed by determining subsurface concentrations in permeation experiments of stressed specimens, considering gaseous^139^ and electrochemical^140^ charging. A possible rupture of the Pd film on the exit surface during electrochemical permeation under stress should be considered as a side effect that influences H oxidation and compromises the reliability of permeation transients.^141,142^

There is a consensus that stress levels near or above the yield stress will lead to the formation of lattice defect, e.g., dislocations and vacancies, which results in an enhanced apparent H concentration.^143^ For tensile stress levels within the elastic regime, some studies have observed a notable increase of H absorption,^144^ while others observed only a weak effect both for diffusion and absorption.^145^ The controversial results could be attributed to the fact that thin permeation samples present a low triaxiality at surfaces and therefore the influence of hydrostatic stress *σ*_h_ on uptake is minor. The influence of compressive stresses on H uptake is relatively less studied. Bockris et al.^146^ experimentally demonstrated that compressive residual stresses led to a reduction of H absorption, which could be attributed to that compressive stresses reduce lattice spacing, thereby suppressing H uptake. As a matter of fact, introducing compressive residual stresses, e.g., via shot peening or laser peening, has been proposed as a promising way to increase the resistance to HE.^147,148^ However, it is challenging to control the process of peening so as not to introduce extensive plasticity which has been shown to increase the apparent concentration of H.^143^

As already mentioned, the determination of apparent or lattice concentrations relies on a proper fitting of diffusion coefficient together with appropriate simplifications. The apparent concentration on the entry side is usually associated to trapping sites and thus expected to increase with plastic strain,^149^ whereas hydrostatic surface stress should only influence the capacity of lattice sites and enhance *C_L_* at the surface. There exist two viable approaches to treat these two aspects:

- To consider that only perfect lattice sites are in contact with the H_2_ gas phase. In this case, the effects of stress- and strain-enhanced H solubility only emerge from the change on *μ_L_* in the strained surface sites. Once H is absorbed, a kinetic exchange occurs until equilibrium is reached between H in these lattice sites and the neighboring traps/defects. Despite that this trapping process will be influenced by stress and strain states, traps do not directly capture H from the H_2_ and are insulated from the environment. This approach is modelled with boundary conditions for lattice H from the extended form of Sievert’s law (eq 13).
- To assume that straining will alter the configuration of lattice defects and therefore the equilibrium between H_2_ and the subsurface H concentration. A thermodynamic treatment for the chemical potential of H in traps is also possible^104^ to account for these effects and to define the corresponding boundary conditions in modeling. In equilibrium, this approach is equivalent to the former because *μ_L_* = *μ_T_*, and therefore the assumption of trap insulation becomes irrelevant.

It is important to note that some studies have hypothesized a significant change in the electronic structure during straining,^150^ which could influence H adsorption states and also H evolution reaction during electrochemical uptake. Additionally, passive films preventing H uptake can interact with H-induced defects^151^ or be broken during straining.^152^ These mechanical aspects on oxide protective layers should also be considered when predicting H absorption.

Despite the indirect observations of an increased bulk concentration caused by strain-induced defects, the role of surface dislocations and vacancies in H adsorption/absorption remains unclear and has not been sufficiently addressed. Some atomistic simulations have explored the possible implications of vacancies on dissociation and adsorption.^153^ Rendulic^154^ studied the influence of point defects and foreign atoms on H_2_ adsorption on Ni, and also analyzed the influence of stepped surfaces, which have been extensively investigated as catalyst promoters due to the increase in the sticking coefficient.^122^

At a larger scale, the influence of surface roughness has also been analyzed in many studies, especially from permeation tests^155,156^ but also numerically.^157^ A rough surface involves a larger specific area and a higher number of adsorption sites but a possible reduction in the effective charging current.^158^

Nanostructured materials have shown improved H_2_ dissociation kinetics for catalytic applications, and thus, a GB effect on adsorption has also been assessed by different studies. Panholzer et al.^159^ found by ab initio calculations that the dissociation barrier was lowered at Mg GBs. Similarly, Sun et al.^160^ studied dissociative adsorption of H_2_ at high-angle GBs in iron and observed a more favorable adsorption compared to the grains. These findings indicate that a synergistic effect between favorable adsorption and short-circuit GB diffusion must be considered.

##### 2.2.1.6 Key Challenges in H–Surface Interaction

In HE research, there has been greater emphasis on studying the interaction between H and the bulk material as well as the microstructure, rather than focusing on surface effects. Additionally, HE research has been traditionally limited in addressing coverage-based mechanisms from surface science, and Sievert’s law is usually considered accurate enough for H uptake from high-pressure gaseous H_2_. Electrochemical theory has enriched mechanistic explanations of environmentally assisted cracking, and a similar synergy should be pursued between disciplines studying H_2_–surface interactions and the H community. The growing interest in H storage materials and in some heterogeneous catalysis applications will surely be accompanied by further insights into the H_2_ dissociative chemisorption on metal surfaces, and this knowledge should be adopted to better predict surface concentrations and its influence on HE. As for many physical processes of interest in material science, bridging scales from the atomistic adsorption phenomena to a real surface behavior is a challenging task that requires multiscale and homogenization procedures. Equilibrium conditions are frequently adopted in the analyses, but a critical validity check is often lacking. Kinetics of surface processes need to be incorporated in predictive models to investigate whether the transient aspects of HE are surface-controlled or diffusion/trapping-controlled.

When determining H adsorption/absorption parameters from indirect experimental observations, it is challenging to differentiate between surface and bulk phenomena. For instance, the surface concentration corresponding to a particular charging condition is usually derived from permeation tests, for which it is necessary to postulate bulk diffusion properties.

The effect of mechanical stresses on H absorption at microscopic and macroscopic levels requires further investigation, which can help explain the influence of loading conditions and factors introduced in the manufacturing process, such as residual stresses. In addition, the role of surface defects, e.g., dislocations nucleated at the surface, is not only important for absorption but also crucial in unraveling the underlying mechanism of H induced fracture. The deviation of a surface from an ideally clean and polished state needs also be addressed when predicting H entry into engineering components. Finally, surface roughness and the barrier effect of passivation films remains to be elucidated.

#### 2.2.2 H Transport Inside a Material

HE roots in the intricate interactions between H and the microstructure, such as the material lattice and various defects like dislocations, precipitates, and GBs. To exert its influence on the mechanical properties of a material, absorbed H needs to reach these specific microstructural sites. Therefore, it is crucial to determine the H content at these locations in order to quantify the impact of H and predict HE. In this section, we explore where H resides within a material, how H is transported to these residential sites, and how to measure and predict the amount of H.

##### 2.2.2.1 H Lattice Diffusion

Interstitial H diffusion obeys a random walk mechanism and thus follows an Einstein-Smoluchovski relation, which is derived for the Brownian motion in fluids but can be extended to atomic solid diffusion. This link between the macroscopic diffusion behavior and individual H atom jumps is the basis of continuum approaches describing stochastic events.

**Figure 4:**
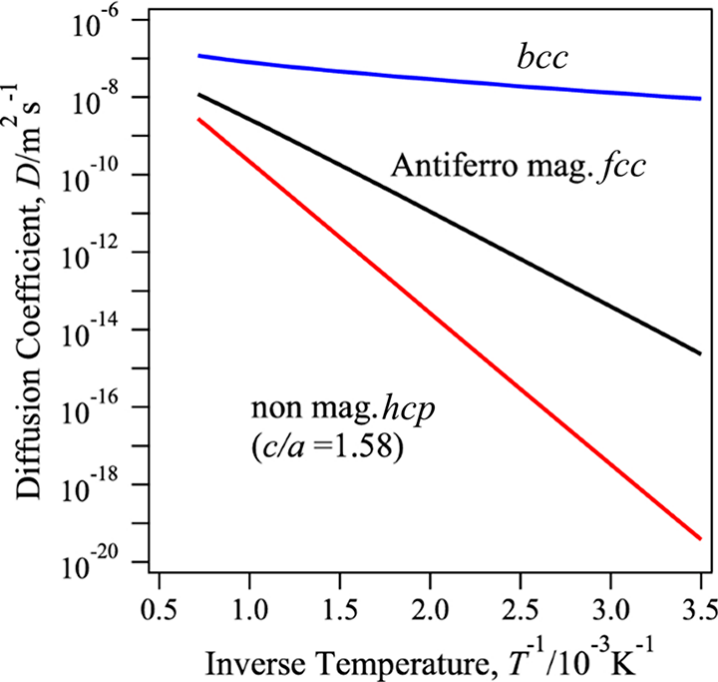
Structure-dependent diffusion coefficients. Diffusion coefficients for H in three different iron structures: *bcc*, *fcc*, and *hcp*. Values were determined through first-principle calculations.^162^ These results consider an antiferromagnetic state in *fcc* and non-magnetic in *hcp* with lattice parameters *a* = 0.246 and *c* = 0.389 nm. Adapted with permission from ref (162). Copyright 2018 Springer Nature.

Interstitial diffusivity, i.e., without trapping effects, correlates directly with the hopping attempt frequency, which is inherently a thermally activated process. Hence, an Arrhenius expression is utilized to predict the diffusion coefficient:
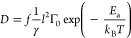
16where *l* represents the diffusion distance, *Γ*_0_ the vibration frequency, *γ* the coordination number, and *f* a correlation factor. The parameter *l* is interpreted as the interatomic distance across two stable lattice sites, serving as a constant for a given lattice structure.^161^ The coordination number, indicative of the count of neighboring sites, depends on the diffusion pathway, i.e., trajectories between octahedral and/or tetrahedral sites.^162^ Both pre-exponential values, *γ* and *l*, are crucial for first principles calculation of diffusion coefficients but are often not discussed in sufficient depth. The correlation factor *f* is included to reflect the dependency of a jump on preceding jumps, thus accounting for the deviation from an ideal random walk. The correlation is a function of the concentration.^163^Figure 4 illustrates Arrhenius plots derived from first-principle calculations for three different crystalline structures, *bcc*, *fcc*, and hexagonal close-packed (*hcp*). A deviation from the classical Arrhenius behavior is highlighted. Non-Arrhenius phenomena have also been experimentally observed. For instance, a strong increase of H diffusivity was observed at low temperatures for *bcc* iron due to quantum effects, deviating from Arrhenius predictions. These quantum contributions encompass discrete vibrational energy levels and quantum tunneling,^164^ which also manifest in the non-classical isotope dependence of H diffusion and the non-Arrhenius form of trapping rates.^165,166^

For amorphous material, the deviation from Arrhenius behavior naturally emerges from the temperature dependence of the short-range order of the structure and thus of the coordination number and activation energies.^167^ Gaussian distributions of energy sites and concentration dependency of diffusivity are also causes for the deviation.^168^

The influence of phonons has been recently explored by analyzing new optical modes in *bcc* iron and their contribution to the Helmholtz free energy.^169^ It was shown that phonon correction improves the agreement with experimental results at high temperatures.^169^ Tang et al.^170^ decoupled phonon behavior from the motion of host atoms in palladium and found that phonons promoted H diffusion.

##### 2.2.2.2 H Trapping

**Figure 5:**
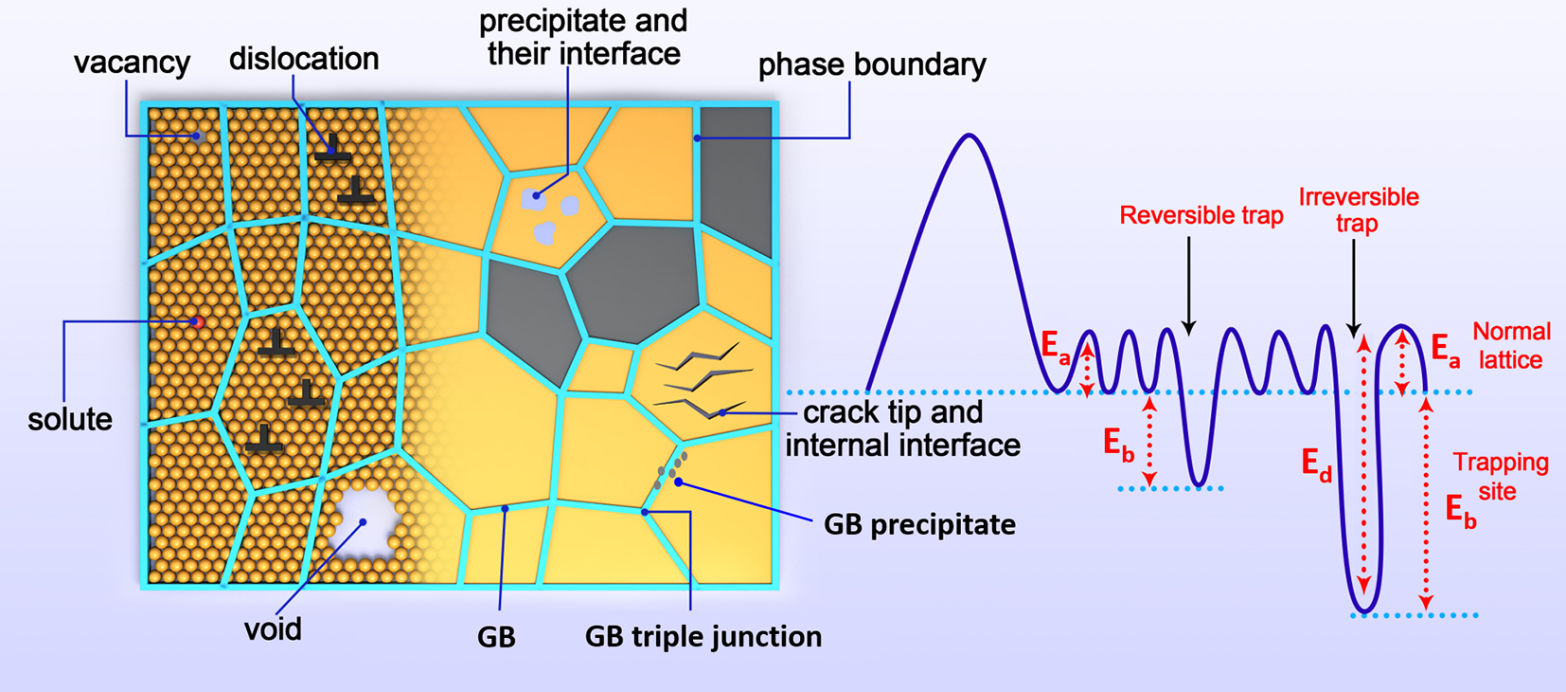
Illustration of typical H trapping sites in the microstructure of a material. The capacity of a site to trap H is scaled by its binding energy *E*_b_, which is the difference between the detrapping energy *E*_d_ and the lattice activation energy *E*_a_. Note that *E*_a_ is sometimes taken as the lattice solution energy *E*_L_ or solution energy *E*_s_. Redrawn based on ref (171). Copyright 2018 Elsevier.

The retention of H atoms at microstructural defects during interstitial diffusion is referred to as H trapping, which is the main cause of the anomalous delay of H transport within a material. H atoms absorbed into a metallic material typically present in three forms in the crystalline structure, i.e., diffusible H, reversibly trapped H, and irreversibly trapped H, depending on their binding energies to different microstructural sites. The well-recognized H trapping sites include defects of various dimensions: zero-dimensional point defects such as vacancies, one-dimensional line defects, i.e., dislocations, two-dimensional interfaces, e.g., grain and phase boundaries, and three-dimensional defects, for instance, phases, microvoids, and microcracks. An illustration of H trapping sites is presented in Figure 5.

*H Trapping at Vacancies and Dislocations*. H retention in vacancies is experimentally difficult to assess but plays a crucial role in some embrittlement theories. Numerical predictions of vacancy binding energy for different metals using effective medium theory have been shown in good agreement with experimental measurement, e.g., ion-beam analysis.^172^ The good agreement might be due to the simple topology of a vacancy in comparison to other types of defects. Atomistic calculation is also a powerful tool to determine the interactions of H with vacancies. A detailed discussion about atomistic modeling of H distribution is presented in Section 2.2.4.

Dislocations are another crucial type of trapping sites for H transport within metals due to their inherent relation with plastic deformation, as well as to their high mobility and active interaction with other types of defects. Sato and Takai^173^ attributed the first peak appearing in low-temperature TDS to dislocation traps in iron because the fitted energy agrees with literature for screw dislocations (28.6 kJ/mol) and the peak value increases with plastic strain. Trapping of H at the core of dislocations has been experimentally demonstrated by atom probe tomography (APT) in a low-carbon martensitic steel with a high dislocation density.^174^ In that case, the TDS peak associated with dislocation trapping is much higher than those corresponding to secondary trapping sites. Similarly, Sugiyama and Takai^175^ found a much higher TDS peak for dislocations in comparison to vacancy traps.

H can also be trapped in the elastic stress field of a dislocation, both for screw and edge types. DFT calculations have shown a difference in preferential site and diffusion path for H trapped in the dislocation elastic stress field^176^ in comparison with H in an unstrained lattice. Based on elasticity theory, H binding energy to dislocation stress field was evaluated and compared to the outcome of atomistic calculation.^177^ H transport in a metal with a high dislocation density is significantly influenced by the formation of dislocation substructure, e.g., dislocation cell walls that can be regarded as embryos of new GBs.^178^

*H Trapping at Interfaces*. Segregation of H along microstructural interfaces is an important trapping phenomenon that tends to delay H diffusion and is essential for understanding IG embrittlement. GB trapping sites can be differentiated between those only associated to the geometrical misorientation between adjacent grains and those to vacancies and dislocations that accommodate the misorientation and that are generated during straining in the GB region. In the former scenario, favorable interstitial sites are responsible for H binding to a GB, which is the case both in iron and in nickel, while in the latter, vacancies and dislocations make a large contribution in the trapping.

The interface between metal matrix and precipitate is another typical trapping site. Vanadium, titanium, and niobium carbides have been extensively studied in the context of H trapping^179−182^ and indicated as beneficial traps that reduce the amount of mobile H. Retention of H in these trapping sites might mitigate or delay embrittlement due to their high binding energies.^183^ However, alloying elements to promote carbide dispersion and beneficial trapping also lead to a solute strengthening effect. This effect causes higher local stresses and thus elevates H accumulation in the stress field. Additionally, coarse precipitates are prone to brittle cracking. Moreover, the carbides can suppress some favorable textures that potentially mitigate HE.^183^ Therefore, it is important to consider both the beneficial and detrimental effects of the precipitates when attempting to engineer them for better HE resistance. For example, those detrimental effects can be minimized by decreasing particle size,^184^ reducing the amount of large undissolved carbides^185^ or controlling tempering temperature.^186^

As mentioned in Section 2.1.3, H can be trapped inside the precipitate, at the matrix–precipitate interface or in the surrounding stress field. Takahashi et al.^187^ discussed different possible trapping sites in vanadium carbides and showed that binding energy drastically changes with ageing conditions due to the different vanadium/carbon atomic ratios, and attributed deep traps to carbon vacancies at the precipitate interface. APT is an excellent approach to mapping H at precipitates; see the detailed discussion in Section 2.2.3.2. The coherent, semi-coherent, or incoherent types of precipitates differ significantly in H trapping properties, as discussed by Shi et al.^182^ In addition, the presence of undesired non-metallic inclusions is inevitable in steels, and their effects on H trapping and induced cracking need further investigation.^188−191^

##### 2.2.2.3 Diffusible vs Non-diffusible H

**Figure 6:**
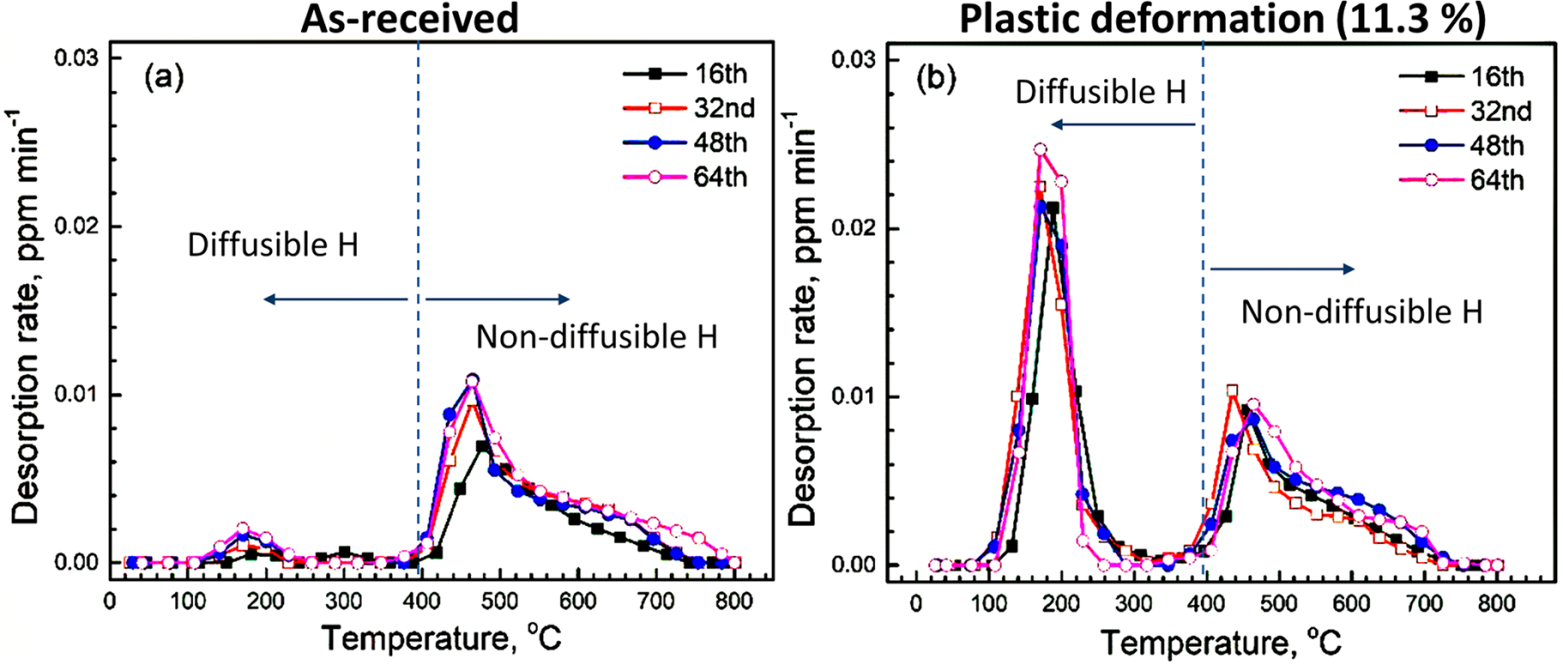
TDS peaks for diffusible and strongly trapped H in high strength steels with different wet-dry testing cycles (cycle numbers shown in the figure legend).^196^ Peaks found at temperatures lower than 400 °C can be attributed to lattice or weakly trapped H. The magnitude of the diffusible H peak increases after plastic straining indicating a weak trapping at dislocations. Strongly trapped H is not altered after plastic deformation for this case. Adapted with permission from ref (196). Copyright 2022 Elsevier under [CC BY 4.0 DEED] [https://creativecommons.org/licenses/by/4.0/].

A critical examination of H retention and its impact on embrittlement necessitates distinguishing between strong and weak H traps. However, the energy threshold defining this distinction remains arbitrary. Similarly, categorizing H as diffusible or mobile lacks a universally accepted methodology, requiring either experimental or numerical benchmarks for clarification. Some studies^192,193^ assumed that H desorption peaks observed in TDS ranging from ambient temperature up to 600 K correspond to diffusible H. Consequently, desorption peaks detected at temperatures below approximately 600 K are attributed to diffusible H, whereas those exceeding this threshold are indicative of H strongly trapped. Similarly, Depover et al.^194^ identified a cut-off at 300 °C for hot extraction measurements to categorize H. In contrast, the ISO 3690:2018 standard for H content determination in arc weld metal via thermal conductivity detection sets the temperature at 400 °C. It is noteworthy that the measured quantity of effused H is influenced by both the temperature and duration of the analysis. Maeda et al.^195^ also adopted the 400 °C benchmark in TDS to define diffusible H and attributed the reduced level of embrittlement to a decrease in that diffusible content. Figure 6 shows TDS profiles of a QP1180 high-strength steel under corrosive conditions, in both as-received and pre-deformed states,^196^ where the 400 °C limit is useful to differentiate between diffusible and non-diffusible H.

The quantification of H content evolution in a sample post-exposure at room temperature serves as a potent methodology for identifying diffusible H, defined by the difference between initial concentration and asymptotic values following infinite rest periods.^186,197^ Strongly trapped H is sometimes referred to as residual H because it quantifies the remaining H concentration after long exposure times. Moreover, the categorization of H trapping effects into reversible or irreversible phenomena is discussed, with weak traps being reversible and strong traps deemed irreversible, as outlined by Frappart et al.^198^ A detailed review of the strength of H traps in various engineering alloys is presented in Subsection 2.2.6 and in Table 3. Additionally, alternative methodologies, including the examination of permeation decay transients, have been employed to differentiate between diffusible and non-diffusible H.^199^

##### 2.2.2.4 Internal vs External H

**Figure 7:**
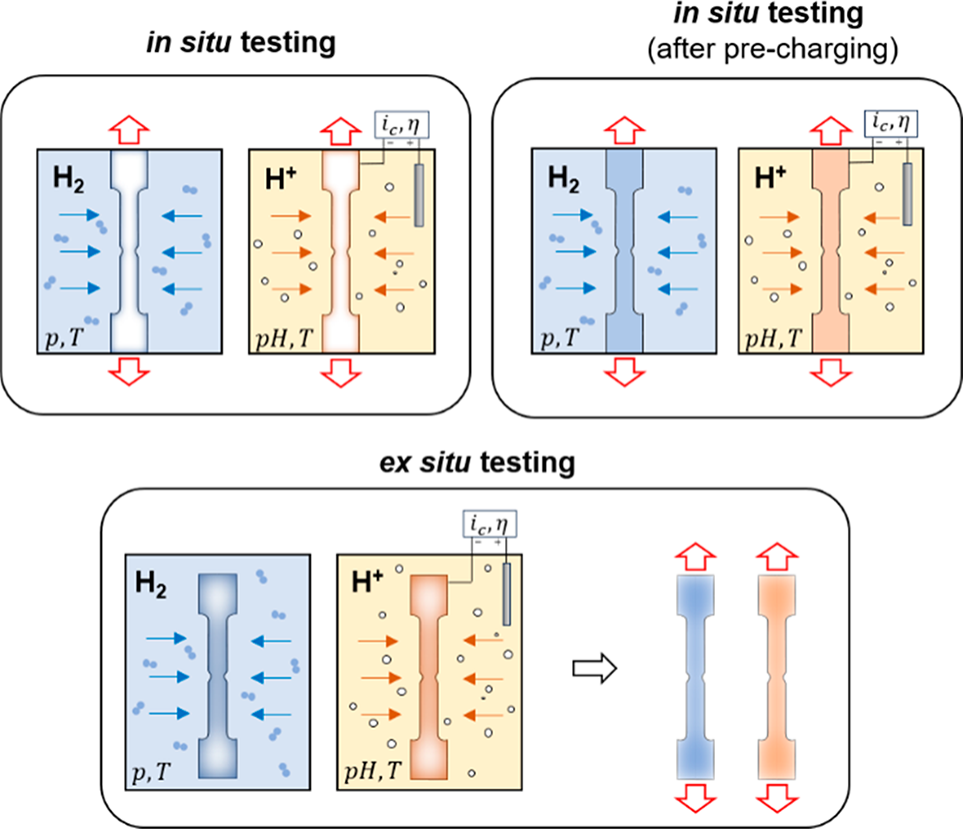
Scheme of gaseous or electrochemical *in situ* versus *ex situ* testing. Mechanical testing, indicated by red arrows, is simultaneous to H uptake for *in situ* procedures. *Ex situ* testing is defined by a pre-charging stage and a subsequent mechanical loading in air. Pre-charging before *in situ* testing is also frequently applied. Gaseous charging is characterized by the pressure and temperature, as well as the purity of the gas. Parameters defining electrochemical charging are more complex and include the electrolyte composition, pH, temperature, charging current (*i*_c_), and overpotential (η).

It may be intuitive to assume that the same H distribution should lead to the same degree of embrittlement. However, the uptake, transport, and possible H degassing during mechanical loading are coupled processes that complicate the comparison between pre-charged testing, also known as *ex situ* charging, and *in situ* H charging approaches. An apparent difference between these two charging approaches is that *ex situ* is done before the mechanical testing, during which H stored in the material can release from the sample surface, and at the same time, interacts with the microstructure. *In situ* H charging provides a continuous H source, which results in different H distribution during mechanical testing. Electrochemical charging and gaseous H charging can be applied in both *in situ* and *ex situ* experiments. On the one hand, *ex situ* H charging is simpler and easily enables an *in situ* observation and record of microstructure evolution during mechanical testing.^200,201^ However, *ex situ* H condition is not best suitable for *bcc* metals considering their high H diffusivity^202^ because pre-charging and mechanical testing may not be able to resolve H effect when H atoms diffuse out quickly.^202^ On the other hand, only *in situ* H charging mimics the real application where H is continuously supplied during loading. However, such H charging requires special setups that can incorporate with mechanical testing. In this sense, *in situ* observation is more challenging, as the experimental setups for *in situ* testing are most complex and expensive. Figure 7 illustrates the *in situ* testing and *ex situ* testing. *In situ* testing can be performed with or without pre-charging.

*In situ* H environment usually results in a more severe mechanical degradation compared with *ex situ* charging.^203,204^ The reason can be attributed to the enhanced apparent solubility and diffusivity of H under *in situ* conditions, which facilitate greater H accumulation at the crack tip, thereby accelerating crack propagation,^204^ while possible degassing of H during *ex situ* testing reduces the amount of H in the material. Caution should be taken when transferring *ex situ* test results to engineering application, as H uptake could be underestimated.

In corrosion science and industrial contexts, two distinct mechanisms of H-assisted cracking are recognized, differentiated by the H source: external H assisted cracking (EHAC) and internal H assisted cracking (IHAC). EHAC pertains to conditions where H is absorbed *in situ* during service, reflecting H uptake from environmental exposure. Conversely, IHAC involves H that pre-exists within the material, typically introduced during manufacturing processes.^205^ In other words, internal H corresponds to *ex situ* H charging condition, while external H corresponds to *in situ* charging condition.

**Figure 8:**
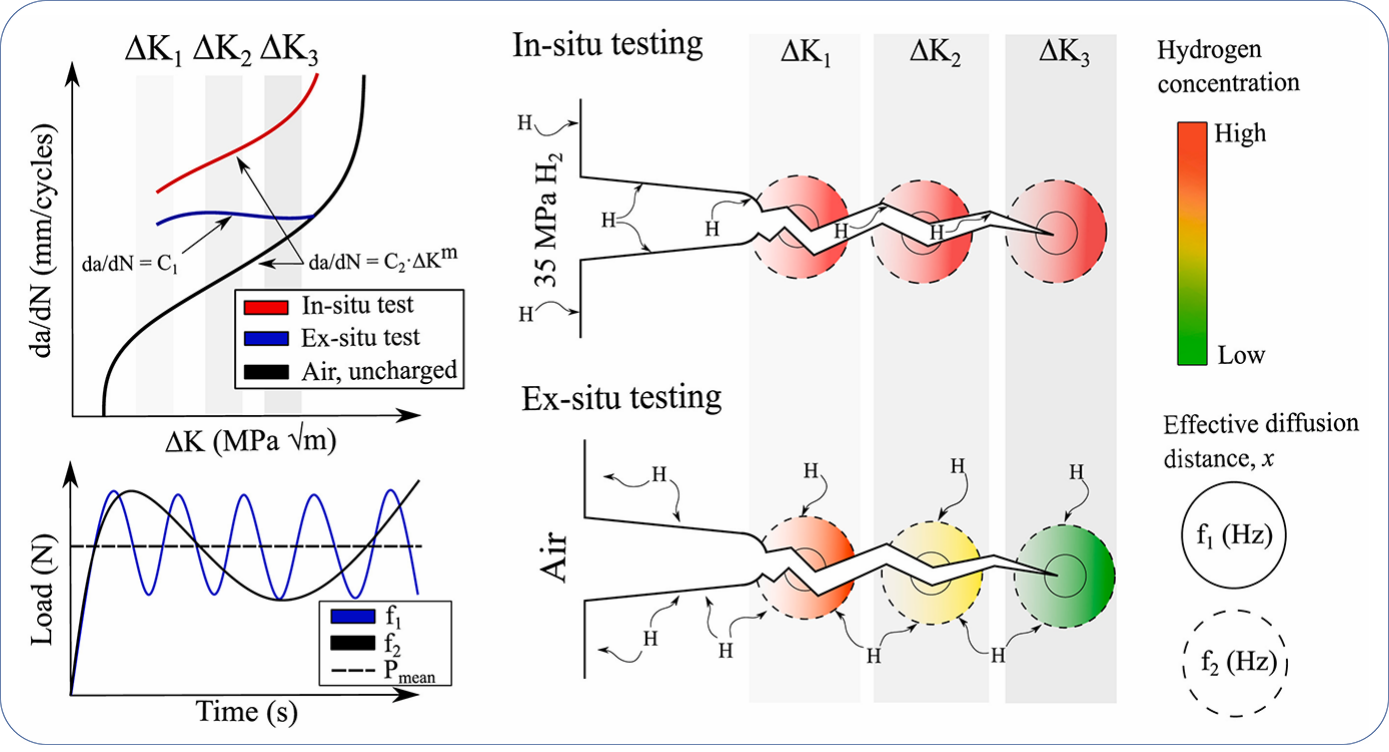
H loss during *ex situ* fatigue testing. A mechanistic interpretation of the effects of *in situ* and *ex situ* testing on the H accumulation process responsible for the acceleration of the crack growth rate during fatigue tests. The figure showcases the H loss associated with *ex situ* experiments and the role of the loading frequency, where lower frequency (*f*_2_) would result in a longer time for H to accumulate. Adapted with permission from ref (203). Copyright 2023 Elsevier under [CC BY 4.0 DEED] [https://creativecommons.org/licenses/by/4.0/].

In SSRT and low-frequency cyclic testing, the distinction between *ex situ* and *in situ* H charging conditions becomes increasingly apparent over extended test durations. Zafra et al.^203^ elucidated the mechanism behind the characteristic plateau observed in *ex situ* fatigue crack growth (FCG) tests: the crack growth curve approaches the uncharged results due to diminished H concentration at the advancing crack front, as depicted in Figure 8. More specifically, the diffusion distance of H is significantly influenced by loading frequency, with a higher frequency reducing the amount of H that can diffuse to the crack tip. This underscores the propensity of lower frequencies to facilitate H redistribution, thus promoting embrittlement. Detailed discussion about the influence of H on the cyclic loading behavior can be found in Section 2.4.3.

**Figure 9:**
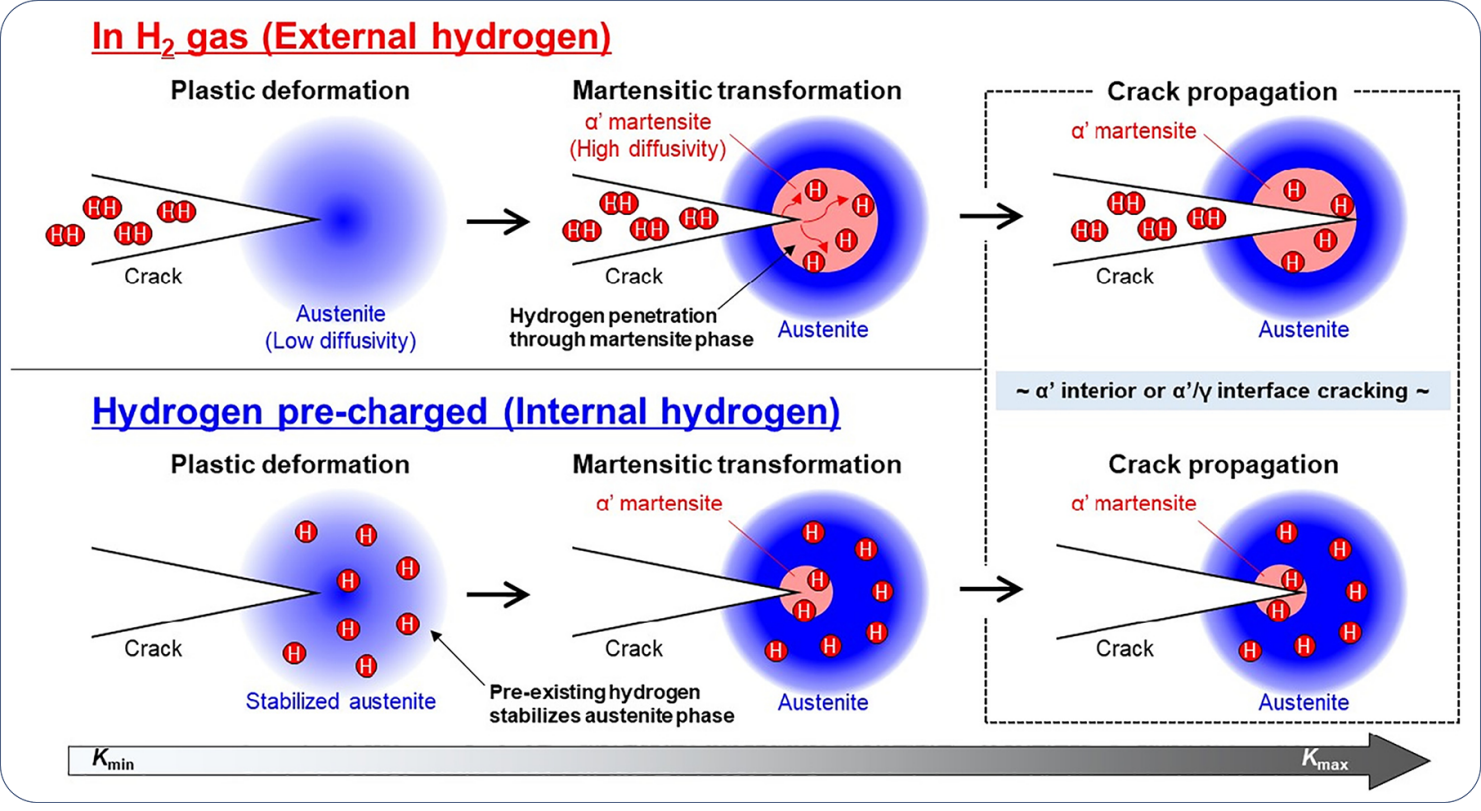
External H versus pre-charged H. The interplay between H and martensitic transformation is crucial to explain the difference between pre-charged and in situ (H_2_) conditions. Pre-charged H stabilizes the austenite phase and therefore embrittlement is suppressed despite the higher concentration. On the other hand, external H diffuses through the strain-induced martensite phase and promotes HE even at low concentrations. Reprinted with permission from ref (205). Copyright 2018 Elsevier.

As mentioned, *in situ* H environment (external H) usually results in a more severe mechanical degradation, and Ogawa et al.^205^ confirmed this in cyclic loading test in austenitic stainless steel. The specimens with internal H showed lower FCG acceleration rates than those in the external H case, while the amount of H was much higher in the former because of the low diffusivity of H in the material. This was interpreted in terms of internal H-modified deformation character, i.e., planer dislocation gliding or deformation twinning, and the resultant stabilization of the austenite phase. Such a positive effect of H was not achieved under external H conditions since α′ transformation in the crack tip zone already took place before H penetrated the austenite phases. This is depicted in Figure 9. Therefore, the internal and external H may exert quite different influence on embrittlement, which is highly dependent on microstructural characteristics of a material.

For metastable austenitic stainless steels, H transport by moving dislocations can promote embrittlement due to external H,^206^ but this effect is hard to decouple from strain-induced martensite formation. It should be noted that H diffusion and trapping at GBs play a critical role in HE of nickel alloys and therefore the difference between external and internal H is expected to be substantial. For external H condition, Wada et al.^207^ found that short-circuit diffusion and H transport by moving dislocations were required to trigger embrittlement, whereas these processes were less important for internal H condition.

##### 2.2.2.5 Influence of Phase Composition

H diffusion is markedly influenced by the lattice structure; thus, the phase composition and morphology of an alloy play pivotal roles in H transport. This is particularly evident in dual-phase alloys, as demonstrated by duplex stainless steels where the diffusivities in ferrite and austenite phases are significantly different. Ferrite serves as an expedited diffusion pathway, while austenite creates a hindrance to H mobility. Consequently, the phase fraction and their interconnectivity are crucial in determining H accumulation and subsequent embrittlement phenomena.^208^ In these steels, the directionality of H diffusion can also be affected by how H gets trapped at the interfaces between austenite and ferrite, as well as the patterning of these phases.^209^ Similar dynamics are seen in ferrite/pearlite microstructures, where pearlite acts as a diffusion barrier.^210^ Similarly, ferrite can act as fast H diffusion path in comparison to martensite, notably, this effect is not attributed to a slower H lattice diffusion in martensite but to H trapping in lath boundaries and dislocations.^211^ The increase in martensite fraction will therefore produce more ferrite/martensite interfaces and enhance H trapping.^137^

Packet boundaries and lath interfaces in specific phases, and interfaces between the constituent phases in dual- and multi-phase alloys are favorable H trapping sites, but the trapping characteristics of these interfaces are difficult to measure experimentally.^212^ Nevertheless, ferrite/cementite interfaces in a pearlitic microstructure have been identified as weak traps^213^ in comparison to ferrite/pearlite interfaces.^214^ Retained austenite interface is believed to trap H,^215^ but it is not easy to assure whether trapping occurs at the phase interface^216^ or the austenite phase itself acts as a sink for H due to its low diffusivity.^217^

##### 2.2.2.6 Modeling of H Transport

Fick’s first law of diffusion asserts that the diffusive flux, *J*, is directly proportional to the negative gradient of concentration, ∇*C*, such that *J* = −*D*∇*C*. This law encapsulates the principle that diffusion proceeds from regions of higher concentration to those of lower concentration, driven by the concentration gradient. The proportionality constant, *D*, represents the diffusion coefficient, which quantifies the diffusion rate subject to the medium’s properties and temperature. Fick’s second law of diffusion, also known as the diffusion equation, describes the time-dependent evolution of concentration within a medium, deriving from Fick’s first law and the principle of mass conservation in the absence of chemical reactions. Diffusion modeling of H in a perfect lattice is based on Fick’s second law. To account for the scenario under mechanical loading, two modifications are introduced: to incorporate the influence of mechanical stress on diffusion and to modify diffusivity to account for H trapping with microstructural defects.

A modified Fick’s second law considering the influence of hydrostatic stresses, *σ*_h_, is widely adopted for the purpose:^218,219^
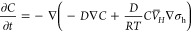
17where *C* can represent total H or more generally, diffusible H that contributes to embrittlement. The implementation of this equation is highly practical in commercial software like Abaqus, where mechanical analysis has been sequentially coupled to mass diffusion analysis as an imbedded function. However, a fully coupled scheme is not readily available. Nonetheless, this can be realized by utilizing user defined subroutines in the software, such as UMATHT, by making an analogy between mass diffusion and heat transfer in thermal analysis.

The diffusion eq 17 is regarded as a one-level model because total concentration *C* is considered. Stress-assisted diffusion is included, but a distinction between H in lattice and trapping sites is not made. Trapping effects can be incorporated to eq 17 if the diffusion coefficient *D* is replaced by experimental values of apparent diffusivities *D*_app_. Apparent diffusivities are commonly found in permeation tests. However, this one-level model does not reflect the concentration-dependent trapping phenomenon. To consider the phenomenon, it is necessary to explicitly consider a trapping concentration term and dividing the total H into two species, lattice *C_L_* and trapped *C_T_*, respectively.
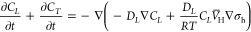
18This two-level continuum modeling approach, including the hydrostatic stress term, was firstly used by Sofronis and McMeeking^220^ and since then has been extensively used to reproduce trapping effects near a crack tip.

Oriani^221^ assessed the validity of thermodynamic equilibrium between lattice and trapped H, and this framework has been extensively utilized for its simplicity, without having to consider the trapping and detrapping frequency and energy. The relation between lattice and trapped occupancies is only a function of the binding energy:
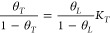
19where *θ_L_* and *θ_T_* are the occupancy of lattice and trapping sites, respectively, and the trapping constant *K*_T_ can be expressed as a function of a binding energy, *K*_T_ = exp(−*E*_b_/*RT*). Given the number of lattice and trapping sites available in a material, the occupancies are readily linked to lattice and trapped H concentration, *C_L_* and *C_T_*.

Taking Oriani’s equilibrium between lattice and trapped H, eq 18 is significantly simplified by reducing the unknown degrees of freedom to *C_L_* solely. Details of implementation can be found in the original reference^220^ which can be regarded as a starting point for continuum modeling of H transport near a crack tip. When Oriani’s equilibrium is assumed, only five material parameters are needed: *D_L_*, *N_L_* describing the ideal lattice, *E_B_*, *N_T_* for trap quantification, and *V̅_H_* to account for hydrostatic effects, discussed in Section 2.2.2.7. Several efforts have been made to develop this two-level modeling approach. Table 1 summarizes a number of important improvements.

**Table 1:** Improvements Made on the Two-Level H Transport Modeling Approach Originally Proposed by Sofronis and McMeeking^220^

Authors (year)	Contributions
Sofronis and McMeeking (1989)^220^	Introduced a reference framework by particularizing the classical Fick’s laws for H transport near a crack tip. This includes considering the partition of H in lattice and traps, the effect of hydrostatic stress on flux, and the impact of plastic deformation on trap density.
	
Krom et al. (1999)^222^	Extended the *∂C_T_*/*∂t* term to account for plastic strain rate, preventing artificial creation or loss of H and capturing the depletion of lattice sites during high plastic strain rates.
	
Lufrano et al. (1998)^223^	Explored coupled effects such as H-induced dilatation crucial for hydride-forming metals and H-induced softening, relevant to the HELP mechanism.
Sofronis et al. (2001)^224^	
Taha and Sofronis (2001)^225^	
	
Dadfarnia et al. (2011)^226^	Accounted for multiple trapping sites to include dislocations, carbides, and GBs, extending the trapping term.
	
Di Leo and Anand (2013)^104^	Developed a formulation based on the chemical potential to naturally account for stress-dependent H uptake and eliminate the need for computing stress gradients.
	
Dadfarnia et al. (2014)^227^	Introduced a mechanistic flux term associated with H transport by dislocations.
	
Turnbull et al. (1996)^108^	Proposed generalized boundary conditions to account for electrochemical inputs at crack tips, translating the imbalance between adsorption and absorption into a flux boundary condition.
Martínez-Pañeda et al. (2020)^110^	
	
Kanayama et al. (2009)^228^	Considered kinetic trapping based on McNabb and Foster’s formulation instead of Oriani’s equilibrium, introducing an additional independent degree of freedom, *C_T_*.
Martínez-Pañeda et al. (2020)^110^	
Charles et al. (2021)^229^	

Complex modeling approaches are elucidated in the framework by Toribio and Kharin,^230^ which introduced a generalized model for H diffusion in metals incorporating multiple trap types. When discrete trapping sites fail to represent the microstructure, e.g., in disordered alloys, a Gaussian distribution for the density and energy of sites can be considered.^231^ Continuum modeling of H transport in resolved polycrystalline structures has also been advanced, employing sophisticated treatments of grains and GBs.^232,233^ This includes the adoption of a diffusivity tensor and consideration of texture effects on H transport,^234^ implementing the impact of GB characteristics and misorientation on macroscopic diffusivity^232,235,236^ and the role of dislocation pile-up at boundaries,^237,238^ as well as integrating transport equations with crystal plasticity formulations.^239^

The explicit consideration of trapping in numerical models, e.g., the two-level models, requires trapping density and binding energy as input parameters. Experimental calibration of the trapping parameters is under constant development. Common experimental methods include H permeation and TDS, based on which two simplified fitting strategies are extended to characterise traps:

- Detrapping energy can be estimated based on Kissinger’s reaction equation^240^ from TDS profiles at different heating rates, which gives the so-called Choo-Lee plots.^241^ However, this simplified method is only strictly valid for detrapping-controlled desorption, generalized assumptions are needed for diffusion-controlled conditions.^242,243^ Additionally, conventional trapping modeling assuming Oriani’s equilibrium is governed by the binding energy, not by the detrapping energy determined by Kinssinger’s fitting of TDS.
- Trap density *N_T_* can be determined from the analytical solution of permeation derived by McNabb and Foster^244^ and applied by Kumnick and Johnson.^245^ However, if the binding energy is not known a priori, *N_T_* can only be derived assuming a saturated state, which sometimes is overlooked. To overcome this limitation, Raina et al.^246^ proposed a general framework to determine both *N_T_* and *E_b_* with permeation test at different concentration levels, i.e., covering a wide range of charging current densities. This strategy has been followed by Peral et al.^158^ using different charging conditions.

Sometimes, it is convenient to apply merely the modified Fick’s second law to model H transport, without explicitly accounting for H trapping. In this case, apparent diffusivity applies which is defined as a phenomenological quantity and expressed as a function of concentration to represent both diluted and saturated trapping scenarios. This approach aligns with permeation standards (ASTM G148 and ISO 17081:2014) and is widely utilized in electrochemical analyses. The use of apparent diffusivity (*D*_app_) facilitates analytical closed-form solutions for fitting permeation transients. Analytical solutions have also been applied to fit decaying H concentrations in samples exposed to ambient conditions, a method followed in several studies.^186,247^ A comparison of testing methods for calibrating diffusivities was performed by Zafra et al.^248^ These fitting procedures were found to be reliable only when H trapping is not pronounced. The diffusion coefficient derived from the Fick’s second law without trapping modification is sometimes referred to as effective diffusivity,^249^ but this could cause confusion with the so-called operational diffusivity *D*_eff_ derived from the two-level models that consider trapping effect.^226^

##### 2.2.2.7 Influence of Stress and Strain

In a similar manner to that described in Section 2.2.1 for H adsorption from a gaseous phase, the chemical potential can be the governing factor for H diffusion in the bulk. The fundamental framework follows Onsager’s transport theory for irreversible processes that occur simultaneously.^250,251^ The driving force for mass transport is the gradient of chemical potential of lattice H. If diffusion flux is only considered from the motion of atoms through interstitial sites, which is reasonable if traps are isolated, the flux vector *J_L_* is proportional to the gradient of *μ_L_*:
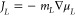
20where *m_L_* = *D_L_C_L_*/*RT* is a mobility factor that links Onsager, Einstein, and Teorell formulae,^251−253^ bridging random motion and thermodynamic continuum diffusion. Note that the flux is a function of lattice concentration in this formulation. The main advantage of this approach over classical Fick’s laws is that all effects, e.g., stress or temperature, that drift ideal diffusion can be captured by only considering their influence on the chemical potential. To account for the effect of mechanical stress on the chemical potential of H in lattice sites, the most common and simplest expression is a linear reduction, derived by McLellan^254^ from thermodynamics arguments:
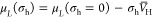
21However, certain assumptions that are sometimes overlooked were made to derive this formula, where the second-order stress terms are neglected: H-induced lattice expansion is isotropic and stress values are much lower than the Young’s modulus. Asymmetrical strain field produced by solute H, which was not considered in the equation above, was implemented by Hirth and Kirchheim.^135,255^

Either eq 17 or eq 21 is able to account for the effect of hydrostatic stress *σ*_h_ on H accumulation, reflecting the observation that HE often initiates near cracks and notches; however, they cannot capture the H enrichment under torsional loading, i.e., in a shear stress field. In contrast, H trapping at dislocations has been demonstrated to depend highly on shear stresses.^256^ Moreover, atomistic results showed a weak hydrostatic stress effect,^257,258^ which conflicted with the large enhancement factor predicted by those equations at high stress triaxialities, e.g., at a crack tip.

Hydrostatic stresses *σ*_h_ in the equations are often obtained based on classical J2 plasticity model, whereas it was pointed out that much higher *σ*_h_ values can be obtained if strain gradient effects are considered, and the implications for HE interpretation are significant.^259^ This is not only because of the higher stresses but also due to a very different stress and hence H distribution over a short range ahead of the crack tip. Within strain gradient plasticity formulation, the role of geometrically necessary dislocations (GNDs) in H uptake and diffusion must better understood, but GNDs also have been demonstrated to govern trapping and H diffusion along GBs,^260^ which may further complicate the modeling framework.

**Figure 10:**
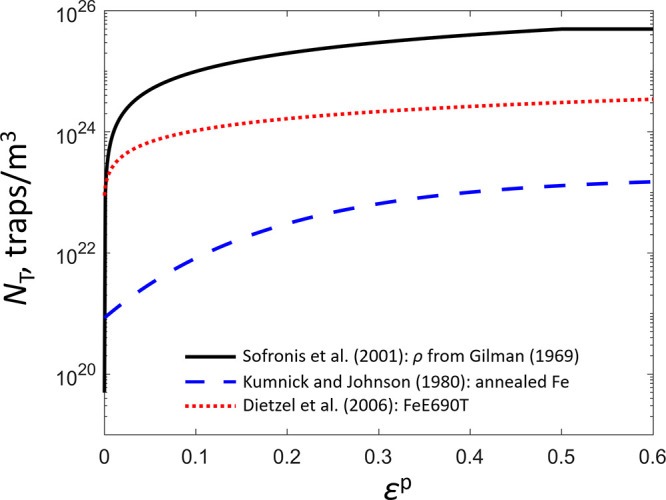
Huge differences in trap density as a function of plastic strain for iron utilized in the three empirical relations used in the literature.

In continuum modeling of H diffusion, increasing plastic strain can be related to an increased number of traps associated with dislocation multiplication. Phenomenological expressions of the trap density *N_T_* as a function of equivalent plastic strain are often employed, and the expressions can be fitted from permeation tests at different pre-strain levels.^261−265^ The expressions typically feature a steep increase in trap densities in early loading stage, followed by a plateau with intensive plastic deformation, as illustrated in Figure 10 showing results of Kumnick and Johnson^245^ and Dietzel et al.^266^ for pure iron.

##### 2.2.2.8 Key Challenges in H Transport

Due to the complexity of H trapping at different microstructural sites, advanced characterization and multiscale modeling are required to accurately assess H transport. As detailed in Section 2.2.3, there are still significant challenges in achieving precise H mapping across different resolution scales, which also hinders H trapping characterization. Therefore, multiscale modeling procedures are crucial to elucidate local transport and accumulation of H, as well as the corresponding delay in mesoscopic diffusion. A promising direction involves the integration of diffusivities and binding energies derived from ab initio calculations into continuum modeling frameworks, aiming to reduce reliance on phenomenological assumptions. However, this approach mandates the development of novel strategies to bridge the substantial gap in length and time scales, inherent between atomistic simulations and continuum models.

Furthermore, the evaluation of H transport at mesoscales necessitates an appropriate approach to encapsulate trapping phenomena into a manageable set of parameters that accurately represent local H accumulation and its transient effects. Techniques commonly employed for characterizing mesoscale H transport, such as permeation test and thermal desorption analysis, are categorized as indirect methods because diffusion and trapping are characterized by the analysis of output flux or degassing. Nevertheless, the extraction of trapping parameters through these techniques is contingent upon certain assumptions, which hold validity only under specific conditions. A comprehensive understanding of trapping mechanisms must also account for factors such as change in concentration, deviation from thermodynamic equilibrium between trapping and lattice sites, and interaction among various trapping sites. Previous studies have critiqued the limitations inherent in traditional fitting methodologies for TDS spectra,^242,243,267^ advocating for a more generalized approach that is backed by direct experimental evidence and adequately addresses the difference among surface-controlled, detrapping-controlled, and diffusion-controlled desorption processes.

Despite the advancing understanding of trapping phenomena, especially for steels and nickel alloys, a persistent discrepancy exists between theoretically calculated H diffusion distances and the experimentally observed range of embrittlement. This gap may be ascribed to a variety of underlying mechanisms that are contingent upon the specific material, loading, and environmental conditions. Notably, there is evidence suggesting that subcritical cracking and non-lattice H transport mechanisms, such as H transport via dislocations or through GBs by short circuit diffusion, play significant roles in this deviation. Beyond the theoretical-experimental mismatch, the numerical prediction of H transport across multiple phases and in alloys susceptible to phase transformations poses considerable challenges. Addressing these complexities necessitates a multiscale and multiphysics-based modeling approach. Despite the extensive modeling effort to predict H accumulation, the experimental mapping of H distribution remains an indispensable component of H analysis, providing essential validation and calibration for simulations.

#### 2.2.3 Experimental Mapping of H

Because of the high mobility of H atoms and versatile nature of H traps as a result of different metallurgical, fabrication, and mechanical treatment approaches, it is extremely difficult to detect the position of H in a free-standing material, let alone combining with different loading conditions. Nevertheless, the advancement of experimental techniques in the past decades has enabled the determination of H diffusivity, bulk H concentration, and binding energies of H trapping sites with a high resolution down to nanometer scale. Furthermore, direct observation of H trapping at defects was realized through APT at a cryogenic condition (cryo-APT).^268^ In this section, experimental approaches for quantitative and qualitative H measurement are summarized, including electrochemical permeation, TDS, cryo-APT, scanning kelvin probe force microscopy (SKPFM), and secondary ion mass spectrometry (SIMS), which fall into four categories: electrochemistry, spectroscopy, microscopy, and tomography-based techniques. The advantages and limitations of these techniques are discussed.

##### 2.2.3.1 Electrochemistry-Based Techniques

**Figure 11:**
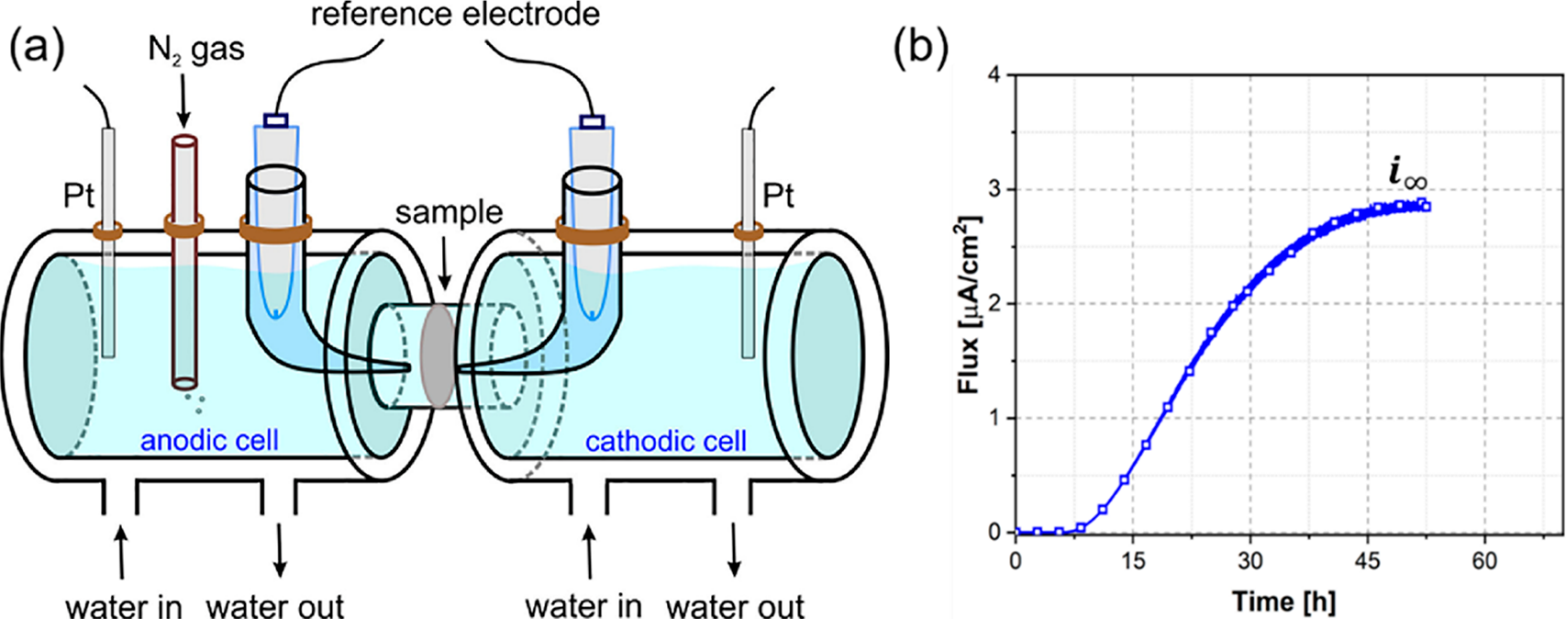
(a) Schematic of electrochemical permeation cell. (b) An example of permeation curve. Reprinted with permission from ref (272). Copyright 2022 Elsevier under [CC BY 4.0 DEED] [https://creativecommons.org/licenses/by/4.0/].

*Electrochemical Permeation*. Electrochemical permeation is widely adopted for measuring H permeation behavior in a broad range of metallic materials.^269−273^ The experimental setup, also referred to as the Devanathan-Stachurski device, consists of two compartment cells, i.e., H charging and oxidation cells. Each cell consists of a reference electrode and a counter electrode, e.g., a Pt wire. The test sample is inserted between the two cells and serves as a working electrode, as illustrated in Figure 11a. For electrochemical H charging in an electrolyte (acidic, alkaline or neutral), either galvanostatic or potentiostatic electrochemical charging is applied. On the detection side, i.e., oxidation side, a constant potential is polarized on the sample surface. As discussed in Section 2.2.1, the permeation of H through a sample consists of complicated physicochemical processes including dissociation of molecular H and adsorption on the sample surface, absorption and diffusion of H toward the oxidation side, and recombination and desorption of H molecules from the sample surface.^274^ In international standards, e.g., G148-97(2018)^275^ and ISO 17081:2004,^276^ one-dimensional diffusion perpendicular to the permeation surface is usually assumed, which is reasonable under the condition that the permeation surface is sufficiently large relative to the thickness. In this case, the permeation rate of H atom towards the oxidation side can simply be expressed by Fick’s second law. As a result, H concentration on the detection side increases with permeation time and finally reaches a steady state, see Figure 11b. Either the so-called time-breakthrough or time-lag method can be employed to calculate H diffusivity. The term time of breakthrough refers to the time needed to detect H for the first time on the oxidation side, while the time of lag is taken as the time elapsed when H flux reaches 0.63-times the steady-state flux.^275^ Once the trapping behavior of H is considered and demonstrated by the permeation transients, we term the obtained H diffusivity as “apparent diffusivity”. It is possible to achieve a state that all traps are filled by a succession of transients, in this way H diffusion through lattice can be obtained. After reaching the steady-state condition, the desorption of H, also called the decay process, can be used to measure H trapping properties.^198^

The approach of electrochemical permeation to measuring H diffusivity is popular because of its flexibility, accessibility, and safety. H charging conditions can be easily adjusted upon need. Further, plastic deformation can be coupled with permeation test to investigate the effect of microstructure evolution, for example, dislocation multiplication, on the permeability.^112,277−279^ In such manner, H diffusivity and trapping property can be correlated by performing tests under different charging conditions. However, there are still challenges with the test and data analysis. For instance, surface impedance being an H permeation barrier, i.e., oxidation layer, is typically observed on the detection side of steels, which poses an obstacle to the measurement of the decay process. To overcome this problem, Pt coating can be applied to promote H absorption, which is necessary for reliable exploitation of the data.^270,280,281^ However, extra coating could deviate the laboratory test from real applications. In addition, permeation test on materials with a low H diffusivity, i.e., *fcc* and *hcp* materials, are considerably difficult in terms of sample preparation and the extremely long testing time. To reduce the testing time, a very thin sample with a thickness of several hundred micrometers has to be used for these materials. Even with such a thin sample, it can take tens of hours before reaching the steady-state flux.^272,273^ Further, it is not feasible to distinguish different types of traps with a permeation test. On top of that, permeation data can be of poor reproducibility as the test is very sensitive to the variation of testing environment, i.e., humidity, temperature, stability of potential, and current. In most cases, repetitive tests are necessary to obtain reliable data.

**Figure 12:**
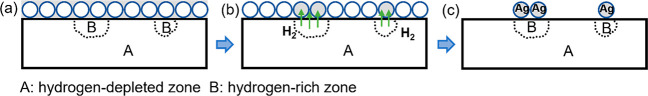
Schematic of H microprint technique to identify H-rich zones. (a) AgBr in emulsion applied on sample surface, (b) Ag^+^ ions locally reduced by H desorbed from H-rich zones, (c) reduced Ag particles left on the surface of H-rich zones. Adapted with permission from ref (284). Copyright 2019 Elsevier.

*H Microprint Technique*. H microprint technique is an H visualization technique developed in the early 1980s.^282,283^ It is a simple, cost-effective method with a relatively high accuracy to reveal the H diffusion path on a sample surface,^284^ see Figure 12. The basic principle is the reduction effect of H atom released from the sample surface, where a layer of photographic emulsion containing silver bromide (AgBr) is prepared before H releasing. The Ag ions (Ag^+^) are reduced to Ag followed by the equation:^285,286^
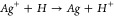
22In this case, the microstructures with higher H emission activities and the distribution of H flux can be visualized as Ag deposits under scanning electron microscope (SEM).^285^ This technique has been combined with heat treatments to successfully study the H trapping behavior of defects, for instance, dislocations and vacancies.^287^ Such experimental method is convenient and safe; however, it can only provide qualitative results of H flux. For quantitative measurement on H desorption, it is more difficult and requires a precise measurement of the reduction products.

##### 2.2.3.2 Spectroscopy-Based Techniques

*Thermal Desorption Spectroscopy*. TDS is an accurate and sensitive technique to measure H uptake, diffusion, and trapping properties of metallic materials. There are two main techniques used in the TDS equipment: the ultrahigh-vacuum and the carrier gas method.^288^ In the former, the sample is subjected to ultrahigh-vacuum and the H particles released from the sample are ionized and accelerated toward the mass spectrometer. In the latter, an inert gas, e.g., nitrogen, is used to carry H gas to the detector at ambient pressure. In the majority of the cases, TDS is conducted on a hydrogenated sample from room temperature to a higher temperature (∼900 °C) at a given heating rate. As the temperature increases, H atoms release from the sample and are collected by the detector.

**Figure 13:**
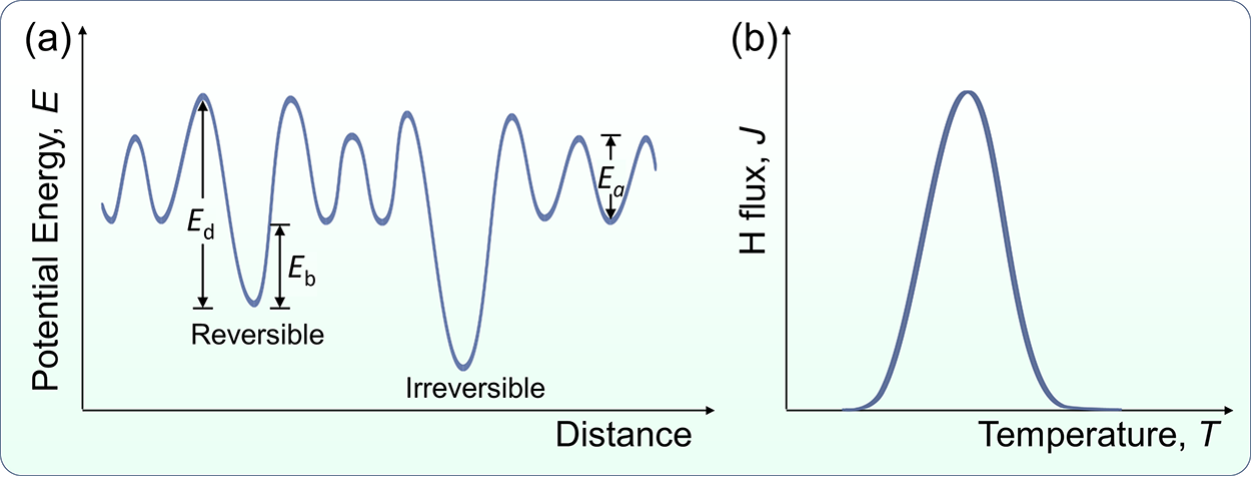
Schematic of (a) potential energy associated with interstitial diffusion sites with activation energy *E*_a_, trapping sites with binding energy *E*_b_ and detrapping energy *E*_d_. The lattice activation energy is equivalent to the solution energy *E*_s_. Note that the definitions of *E*_b_ and *E*_d_ in the original reference were slightly different, and adaptations were made accordingly in the present figure. Redrawn with permission based on ref (289). Copyright 2012 Elsevier. (b) Representative H desorption curve from TDS.

Compared with the electrochemical methods, the resultant TDS profile provides quantitative data not only for the bulk material but also the activation energies for different trapping sites. Specifically, the appearance of several desorption peaks indicates the emission of H from specific trapping sites, and the desorption peak temperature is highly dependent on the binding energy of H with corresponding trapping sites^289^ (Figure 13). In addition, the trapping characteristics of defects are determined by the binding energies for H, i.e., reversible trapping sites have much lower binding energies compared with irreversible trapping sites.^290,291^ In another word, reversibly trapped H can receive sufficient activation energy to escape from trapping sites at higher temperatures, whereas irreversibly trapped H cannot be released because of a high binding energy (> 60 kJ/mol).^93,226^ Likewise, the binding energy for H lies on the nature of defects. Specifically, vacancies, dislocations, GBs, and phase boundaries are considered as reversible trapping sites.^290,291^ Carbides and microvoids are typical irreversible trapping sites.^292−294^ In addition to trapping information, diffusible H can be captured on some low H diffusivity metals at temperature below approximately 300 °C.^292^

There are different ways to analyze TDS data to retrieve the binding energy. A commonly used approach is the analytical regression based on desorption profiles at several heating rates.^93,295^ Kissinger’s diffusion reaction equation^240^ is applied, which for temperature *T*, gas constant *R*, and time *t* reads:
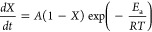
23where *X* is the fraction of released H, *A* is a frequency factor, *E*_a_ is the activation energy for H detrapping. In this context, the detrapping energy of H from trapping sites can be unambiguously calculated from the slope of linear regression of inverse of peak temperature, , as a function of , where *φ* is the heating rate.

Despite its versatility and popularity in probing diffusion and trapping behavior of H, there still exist some challenges in using TDS to acquire desired information. One clear challenge is how to deconvolute and interpret the TDS data and correlate it to the microstructure. Because there is no specific boundary and definition in terms of the binding energies of defects, the peak deconvolution can be somewhat arbitrary and debatable.^267^ In most cases, several traps show similar binding energies, and the interpretation of data requires rigorous characterization and interpretation of the microstructure. Another challenge is associated with the microstructures. Specifically, for *fcc* metals, the diffusion activation energy of H is close to the detrapping energy of H from some reversible traps with approximately 50 kJ/mol.^296,297^ For *fcc* metals that are not hydrogenated close to a saturation level, the interpretation of energies can be misleading. Also, the testing of *bcc* metals is in general difficult as H diffusivity is high (∼10^–8^–10^–11^ m^2^/s).^298^ H released from the lattice cannot be exactly captured, in particular for the testing at ultrahigh vacuum condition, where a certain amount of H can diffuse out during stabilization in the vacuum chamber.

**Figure 14:**
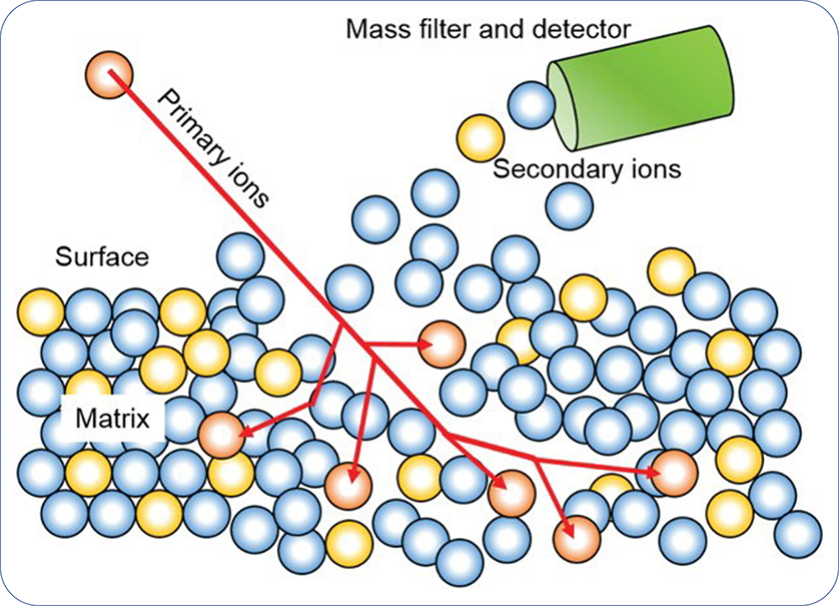
Schematic of the SIMS technique showing the process of sample surface bombardment by primary ion, and secondary ions ejected from the surface collected by detector. Reprinted with permission from ref (304). Copyright 2017 Sage under [CC BY 4.0 DEED] [https://creativecommons.org/licenses/by/4.0/].

*Secondary Ion Mass Spectrometry*. SIMS is a desorption mass spectrometry technique that offers great lateral resolution compared with permeation and TDS tests. Through bombarding the sample surface using an energetic primary ion, secondary ions (positively or negatively charged) can be ejected from the surface and collected by the spectrometry detector (Figure 14). Such mass spectrometry technique has been used to identify the location of H and its isotopes because it can be determined by the mass-to-charge ratio.^299^ It allows a direct observation of H distribution and provides important basis for studying H diffusion and trapping.^300^ To analyze a relatively wide mass range, a time-of-flight (TOF) analyzer is used, and the instrument is thus called TOF-SIMS with a lateral resolution of approximately 150 nm. With the advancement of nanoscale SIMS, the spatial resolution can reach about 50 nm, which enables local microstructural characterization. Typically, deuterium instead of H is used as an H tracer because deuterium is assumed to induce higher strain in the crystal lattice and improve the SIMS analysis detection limits.^301^ The reason is to eliminate the background H signal. The results obtained from SIMS are usually cross correlated with that from transmission electron microscopy (TEM), SEM, and electron backscatter diffraction (EBSD) to reveal the H distribution at a variety of microstructures, for instance implantation defects,^301^ phase, and phase boundaries.^15^ It was also exploited to analyze H-induced microstructure change.^300,302^ In addition, SIMS was used for mapping and characterizing surface H distribution of cylindrical samples of steel, giving a good resolution.^303^ However, quantitative mapping of H using SIMS technique remains challenging because of the strong matrix effect and the difficulty in finding reference materials. Improvements are still needed regarding the spatial resolution and signal anti-jamming. Furthermore, SIMS is a destructive analytical technique in which material is removed from the bulk and it is difficult to replicate results.

##### 2.2.3.3 Microscopy-Based Techniques

*Scanning Kelvin Probe Force Microscopy*. SKPFM is a non-destructive and novel tool to measure H distribution, diffusion, and segregation in materials with high spatial resolution and sensitivity down to the scale of a single GB.^305^ The principle of SKPFM is a comparison of contact potential difference (CPD) between the sample surface and the probe. In an H environment, the CPD fluctuates as a function of localized H concentration in materials. The calculation of CPD, written as *V*_CPD_, is expressed as^306^
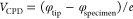
24where φ_tip_ and φ_specimen_ represent work functions of the tip and sample surface, respectively. *e* is the value of elementary charge (1.602 × 10^–19^*C*). The egression of H lowers the work function of the specimen and thus increases *V*_CPD_ when *φ*_tip_ is constant.

**Figure 15:**
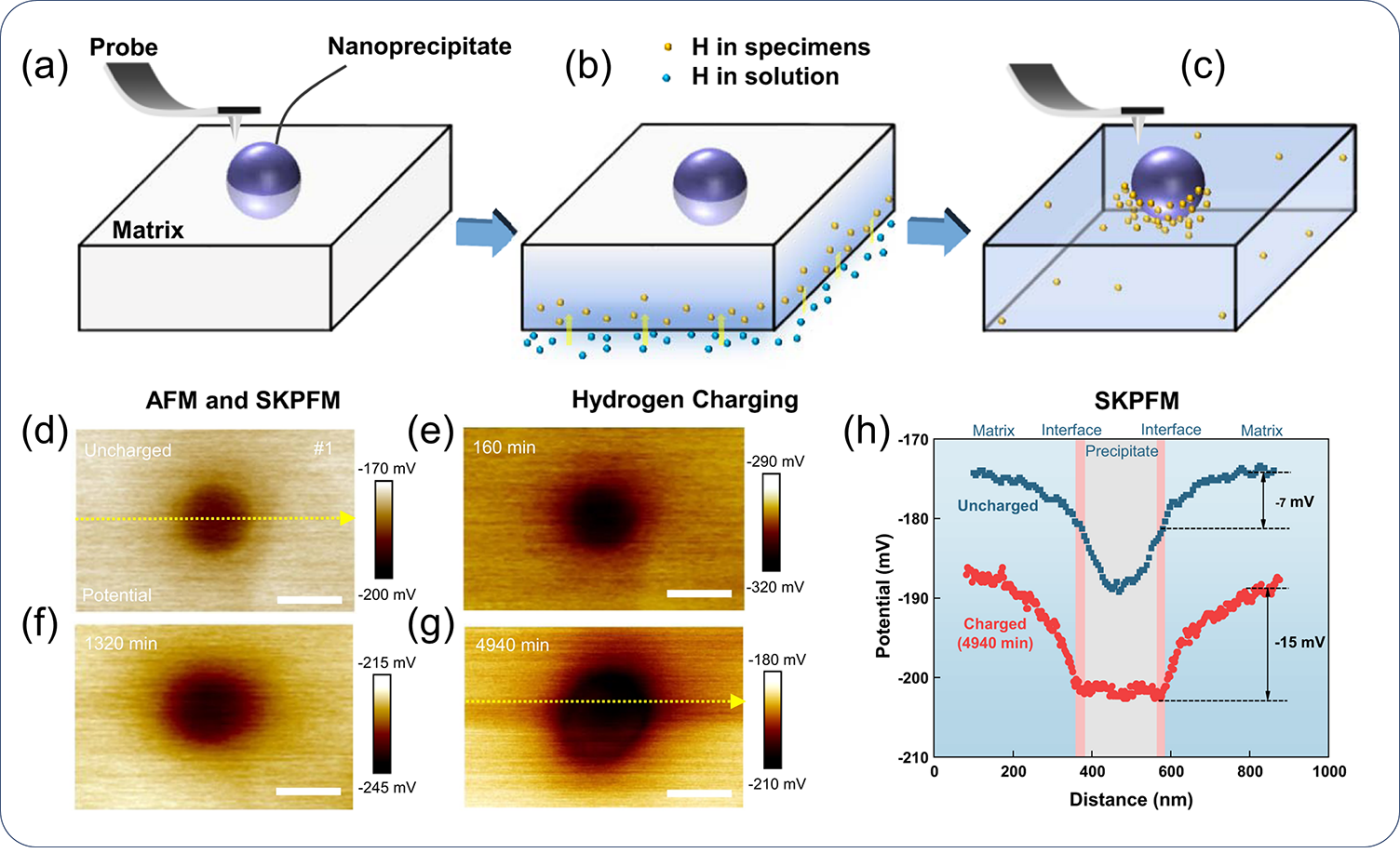
(a–c) Illustration of experimental processes for using SKPFM to detect H at nanoprecipitates interfaces. (d–g) Potential maps of a nanoprecipitate at H-free and H-charged states at *t* = 160 min, 1320 min, and 4940 min. (h) *V*_CPD_ profiles along the yellow dotted lines in (d) and (g). All scale bars, 200 nm. Adapted with permission from ref (313). Copyright 2022 Springer Nature under [CC BY 4.0 DEED] [https://creativecommons.org/licenses/by/4.0/].

Because of its capability of both directly and dynamically mapping H distribution with high lateral resolution, SKPFM has been adopted to probe H signal at GBs and twin boundaries.^305,307^ An example of employing SKPFM for probing H trapping and exclusion at incoherent nanoprecipitates is shown in Figure 15. By recording the change of *V*_CPD_, dynamic observation of H egression on the front surface can be achieved. As demonstrated in Figure 15, after the completion of H desorption, potential of the interface between nanoprecipitate and the martensite matrix is the lowest. In addition, the potential drops more steeply at the interface compared to that inside the matrix and the precipitate, indicating that the interface traps more H than the matrix. So far, SKPFM has been successfully used to determine H distribution in stainless steel,^308−310^ nickel,^305^ aluminum alloy,^16^ and palladium.^308^ However, full quantification of H is still challenging because the calibration of potential is difficult^304^ and the surface condition of the sample plays a crucial role. In many cases, SKPFM is combined with other characterization techniques, for instance, TDS,^310−312^ glow discharge optical emission spectroscopy,^310^ and SIMS,^306^ to robustly reveal H distribution and trapping related to microstructural defects. Nevertheless, SKPFM has been primarily applied on *fcc* metals after H pre-charging because of the slow H diffusivity. Still, *in situ* H charging with scanning designated for *bcc* metals is lacking.

##### 2.2.3.4 Tomography-Based Techniques

**Figure 16:**
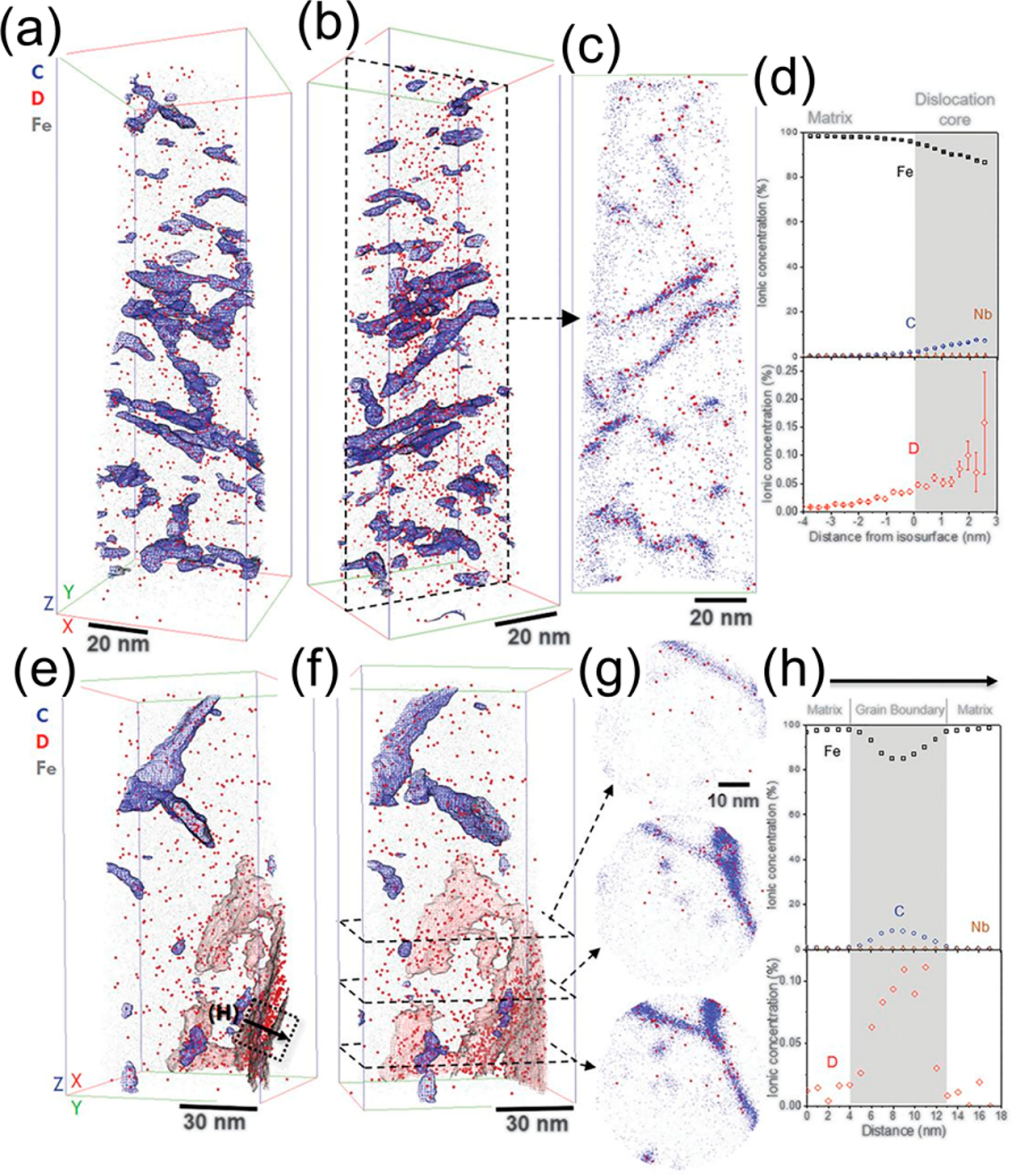
H trapping at GBs and dislocations in martensitic steel samples by cryo-APT: (a–c) three different views showing deuterium segregation at carbon-decorated dislocations. (d) Collective proxigram analysis of dislocations and carbon isosurfaces showing an enrichment of carbon and deuterium at dislocation cores. (e–g) Different views containing a GB region highlighted by a transparent red isosurface, and deuterium rich zone therein. (h) 1D composition profile across the GB marked in (e). Reprinted with permission from ref (174). Copyright 2020 Science.

*Atom Probe Tomography*. The above-mentioned characterization techniques are widely adopted for H mapping with respective spatial resolution. However, direct observation of H trapping at nano-scale precipitates, for instance, TiC and VC, and dislocations is difficult.^268^ To overcome the challenge, three-dimensional APT was developed as an excellent tool to visualize H distribution with a high spatial resolution down to atomic-scale level.^187,314−316^ The principle of APT is bombarding a sharp tip (typically 80 × 80 × 200 nm^3^) using a pulsed laser or voltage to evaporate surface atoms. Because of the high resolution of approximately 1 nm, a three-dimensional image, structure information, and chemical composition of the tip can be visualized after the reconstruction of the evaporated atoms. Clear challenges pertaining to this technique are on the one aspect the high mobility of H atom that can diffuse out in a short time, and on the other aspect the inevitable H signal from background.^268^ To tackle these problems, deuterium with natural abundance of 0.015% was charged to the tip sample to make a distinction from the background H.^314,315,317,318^ Further, cryogenic sample transfer protocols were developed to reduce H loss.^268^ In this manner, H trapping at V-Mo-Nb carbides,^10^ nano-sized NbC, dislocations, and GBs^174^ was revealed (see Figure 16). With the above benefits from cryo-APT, limitations are still expected in regard to (1) the extremely small size of the tested sample tip that cannot reflect bulk property, (2) the destructive nature of the method makes it impossible to have a statistical analysis of trapping properties, and (3) the complication of sample fabrication and low accessibility of the testing equipment.

Other H mapping techniques that are relatively less applied in HE study include neutron tomography^319^ and atomic force microscopy.^320^ Neutron is very sensitivity to light elements, and it raises strong contrast between H-rich and H-clean regions as a result of the strong interaction between neutron and H proton. A three-dimensional view of H trapping at cracks and blisters can be visualized but with a limited spatial resolution with neutron tomography. Atomic force microscopy has been used to study H adsorption and desorption on thin films.^321^

##### 2.2.3.5 Summary of H Mapping Techniques and Key Challenges

A summary of the H mapping techniques discussed above is shown in Table 2. Main advantages and challenges are listed to provide reference for selecting appropriate techniques for relevant HE study. Because each technique has pros and cons, it is not unusual to combine several of them to draw a complete view of H uptake, diffusion, and trapping. A critical limitation inherent to the aforementioned methodologies lies in their incapacity for real-time visualization of H distribution during mechanical loading, while such a dynamic mapping is key to elucidating H-induced fracture mechanism in a given material.

**Table 2:** Summary of the Available H Mapping Techniques

H mapping technique	Application	Spatial resolution	Main advantage	Main challenge
Electrochemical permeation	Bulk material	None, dependent on the electrochemical signal.	Simple setup, adaptable to temperature control and mechanical loading equipment.	Encounters issues with surface impedance; difficult to test on samples with low H diffusivity; inability to distinguish between trap types; highly sensitive to the testing environment.
H microprint	Bulk material	Sub-micron.	Simple setup, capable of mapping individual defects.	Quantitative analysis of H desorption is problematic.
TDS	Bulk material	None, contingent on sample size.	Can elucidate H diffusion and trapping related to specific microstructures.	Difficulties in capturing diffusible H in *bcc* metals without cryogenic setups; interpretation of TDS spectra necessitates extensive microstructure characterization; for *fcc* metals, distinguishing energies between diffusible and trapped H demands meticulous experimental planning.
SIMS	Bulk material	Tens of nanometers to micrometer.	Facilitates direct observation of H distribution across specific microstructures, compatible with microscopy techniques.	Quantitative mapping of H is intricate due to the pronounced matrix effect; destructive nature of tests complicates result replication.
SKPFM	Bulk material	Tens of nanometers.	Direct observation of H distribution within specific microstructures, supports kinetic H evolution analysis.	Full quantification of H is difficult due to the complexity of potential calibration; susceptible to surface conditions; measuring on *bcc* metals poses challenges due to their high H diffusivity, necessitating in situ setups.
Cryo-APT	APT tip	nanometer.	Enables 3D mapping of structural information and chemical composition, with high spatial resolution.	Requires sophisticated testing setups and sample preparation; cryogenic conditions are required, or deuterium is substituted to mitigate background H interference; the small probe tip does not accurately represent bulk properties; destructive testing complicates statistical analysis of trapping behavior.

#### 2.2.4 Atomistic Modeling of H Distribution

Despite the advancement in experimental techniques for H characterization, the observation of individual H atoms at specific microstructural locations, especially under loading conditions, remains beyond current capabilities. Furthermore, experimentally determining the movement trajectories of H atoms as they shift between sites is not feasible due to the difficulty to access the required temporal and spatial scales. As a result, the predominant method for studying the accommodation and migration of individual H atoms continues to be atomistic simulation. Three energy terms are frequently applied when evaluating the tendency of H accommodation, the solution energy and binding energy. Solution energy refers to the energy associated with dissolving H atoms into the metal lattice. It is the energy change when an H atom is introduced into the host metal without being trapped or bound to specific defects. The H solution energy is evaluated as , where *E*_i+H_, *E*_i_, and *E*_H_2__ are the energies of the system containing H, the system without H, and the energy of isolated H molecules. It describes the tendency of H residing at a single site by breaking down the H molecules, e.g., in the lattice or at a trapping site. The stability of the H-site combination in materials is inversely proportional to the solution energy: the lower the solution energy, the more stable the combination, and, consequently, the more likely it is for H to reside at that site. It is crucial to consider the sign of the solution energy in this evaluation. For instance, the propensity for H to reside at a site increases as the solution energy decreases, such as when moving from 0.5 eV to 0.1 eV, or further to −0.1 eV. Binding energy *E*_b_ refers to the energy required to remove an H atom from a specific site, such as a GB, dislocation, or vacancy, to which it is bound. It is a measure of the strength of the interaction between H and these microstructural features. The H segregation energy to a lattice site can be related to the solution energy. Among the various methodologies for calculating the energetics, DFT is considered the most precise. DFT calculations provide detailed insights into the electronic structures of materials, thereby enabling a more accurate prediction of H accommodation in various microstructural sites.^322,323^ The simulations are mainly performed in a ground state corresponding to 0 K; thus, the zero-point energy correction is necessarily considered, especially for H atoms.

##### 2.2.4.1 H Solution in the Lattice

Atomistic calculations have been applied to evaluate the solution energy of H at various trapping sites in lattice or various crystalline defects. In the bulk of *bcc* iron and *fcc* nickel, two possible interstitial sites for H dissolution are tetrahedral and octahedral ones. H solution energies in these sites calculated with DFT are 0.19–0.27 eV for tetrahedral and 0.26–0.35 eV for octahedral in *bcc* iron^324−327^ and 0.29–0.38 eV for tetrahedral and 0.05–0.19 eV for octahedral in *fcc* nickel.^294,328−331^ As such, dissolution of H into the bulk of a metal is endothermic. Another indication is that H prefers the tetrahedral sites to the octahedral sites in *bcc* iron while it does the opposite in nickel. Thus, it is the tetrahedral sites in *bcc* iron and the octahedral sites in nickel that take the main responsibility of accommodating H in the bulk. Further, the corresponding solution energy of octahedral sites in nickel is much lower than tetrahedral sites in iron, which results in the higher solubility of H in nickel compared to *bcc* iron. Even with some fluctuations, the DFT calculation results are comparable to the experimentally measured solution energies that are 0.18–0.3 eV in *bcc* iron^101,255,332^ and 0.13–0.22 eV in nickel.^333−336^ The experimental values are measured in an average sense, as current techniques lack the precision to distinguish H in different types of sites. This results in a generalized representation of H location rather than a specific, site-by-site analysis.

##### 2.2.4.2 H Diffusion in the Lattice

Besides the preferred occupation sites, the kinetics of H in the bulk can also be probed by DFT-based simulations.^82,116,164,337,338^ The diffusion of H in material observed macroscopically is essentially a collective behavior of H atoms doing random walks between neighboring interstitial sites. Using nudged elastic band (NEB) method, Jiang et al.^337^ found that H movement in the *bcc* lattice of iron deviates from a linear trajectory, occurring instead via hops between tetrahedral sites along a curved path. This observation refutes earlier theories of H passing the octahedral sites as saddle points. The key barrier to H diffusion is the energy differential between the minimum and maximum points in the energy profile, identified as 0.088 eV, indicative of high H mobility in iron. In contrast, H diffusion involves movement between octahedral sites in nickel, with a significantly higher diffusion barrier of 0.37 eV, calculated via DFT.^164^ This explains the low diffusion coefficient in nickel which is 4–8 orders of magnitude smaller than that in *bcc* iron.

##### 2.2.4.3 H Trapping at Defects

When the H atom travels inside the bulk, it encounters numerous microstructural impurities, such as vacancies,^325,326,339−343^ dislocations,^42,344−347^ GBs,^348−350^ free surface (FS),^349,351−353^ phase interface,^354,355^ precipitates,^325,356−358^ or combinations of these. Although defects occupy only a small fraction of the material’s volume; they act as trapping sites and retain a substantial quantity of H. This trapping effect significantly alters the mobility of H in the vicinity, resulting in a notably heterogeneous distribution of H within the material. The local behavior of H at these defect sites is key to understanding the interaction between H and the microstructure. The trap binding energy, or binding energy, is defined as the difference between the detrapping energy at a defect and the activation energy at lattice sites. More specifically, *E*_b_ = *E*_d_ – *E*_a_, where *E*_d_ and *E*_a_ are the detrapping energy at defects and activation energy in the lattice, respectively. For *bcc* iron, *E*_a_ is evaluated at the tetrahedral site. For nickel, *E*_a_ is evaluated at the octahedral site. The binding energy quantifies the capacity of a defect to attract and capture H from the lattice. This concept is synonymous with the binding energy of traps used in experimental contexts. Notably, binding energy is characterized by a negative value, meaning that H always prefers a trap to a lattice site. This aligns with the earlier discussion about H solution energy, namely, the smaller the binding energy a site possesses, the more preferable it is for H. Therefore, a smaller binding energy indicates a stronger trapping effect. For example, a binding energy of −0.50 eV signifies an intensified trapping effect compared to a binding energy of −0.30 eV.

**Figure 17:**
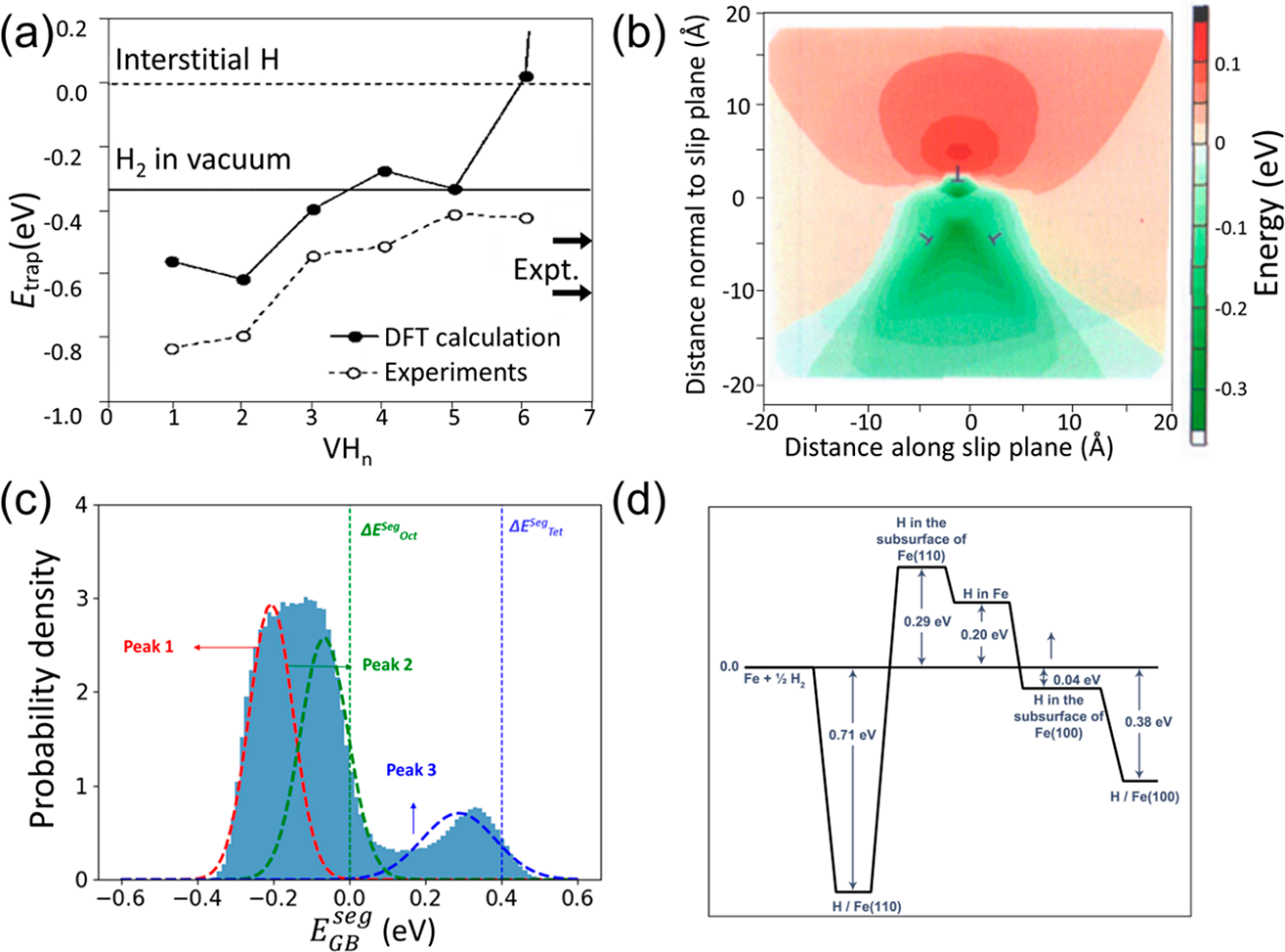
(a) H binding energies as a function of the number of trapped H atoms for a vacancy in α-iron. VH_*n*_ denotes the monovacancy with *n* trapped H, and *E*_b_ is the binding energy for the *n*th absorbed H atom. Reprinted with permission from ref (339). Copyright 2003 American Physical Society. (b) Binding energy of H near the a/6[110] Lomer dislocation in nickel. The Lomer dislocation has dissociated into a locked configuration. The partials that have moved off the (100) glide plane are indicated by the inclined T symbols and core of the a/6[110] dislocation is indicated by the inverted T. Reprinted with permission from ref (360). Copyright 1995 Institute of Physics. (c) Probability density distribution of H binding energies at GBs of polycrystalline nickel, with the data collected from numerous local GB configurations. The spectrum is decomposed into three Gaussian distributions denoted by red, green, and blue dash curves, respectively. The segregation energies of octahedral and tetrahedral sites in the lattice are indicated by the vertical dashed line. Reprinted with permission from ref (365). Copyright 2023 Elsevier under [CC BY 4.0 DEED] [https://creativecommons.org/licenses/by/4.0/]. (d) Binding energy landscape for H in bulk Fe, and the surfaces and subsurfaces of Fe(110) and Fe(100). Zero-point energies are excluded. The energy of a thick slab of Fe (labeled here simply as Fe with either (110) or (100) surfaces plus one-half of an isolated H_2_ is set as zero. Redrawn with permission from ref (337). Copyright 2004 American Physical Society.

Starting with vacancies, a simple type of point defect, Tateyama et al.^339^ investigated the stability of vacancy–H complexes (VHs) in *bcc* iron using DFT, as depicted in Figure 17a. They determined that the binding energies for VH and VH_2_ formations are approximately −0.60 eV, lower than those for VH_3_ and VH_6_, which are around −0.4 eV. Therefore, VH and VH_2_ are more stable and energetically favorable. Notably, these DFT results align closely with experimental measurements, which are approximately −0.63 eV. They concluded that VH_2_ is the predominant complex under ambient conditions, correcting the earlier hypothesis that VH_6_ is more common. Counts et al.^325^ showed that in iron, a vacancy with a binding energy of −0.57 eV is a more effective H trapping site than other point defects, such as solute atoms with a similar binding energy. In nickel, Connétable et al.^359^ reported binding energies of −0.26 eV and −0.4 eV for monovacancy and divacancy, respectively. Beyond monovacancy and divacancy, H can also accumulate significantly in vacancy clusters, eventually forming a nanovoid. Geng et al.^343^ found that H_2_ begins to form in a 9-vacancy void in *bcc* iron once sufficient H is adsorbed on the void surface, and a H-saturated nanovoid exhibits a stronger attraction to vacancies than a H-free nanovoid, due to the H–vacancy interaction.

Relative to vacancies, dislocations exhibit a comparatively weaker trapping capability for H. Using DFT, Itakura et al.^346^ assessed the binding energy of H at various positions and dislocation configurations in iron, particularly focusing on screw dislocations. Their findings revealed that the minimal binding energy for H at a stable screw dislocation configuration is around −0.26 eV. By combining DFT and experimental data, Angelo et al.^360^ applied the EAM approach to model H interactions with edge, screw, and Lomer dislocation junctions in nickel, as illustrated in Figure 17b. They identified that the lowest trapping site energy for both edge and screw dislocations is approximately −0.1 eV, occurring 3–4 Å from the dislocation core under tensile stress. For the Lomer dislocation, the minimal binding energy was found to be −0.33 eV, located at the core of the a/6(110) dislocation.

GBs in materials differ significantly from vacancies and dislocations due to their virtually unlimited potential configurations, reflected by varying misorientation angles and GB planes. As a result, H binding energy at GBs can vary extensively based on the specific GB type and local geometry. In the literature, GBs are predominantly modeled in bi-crystals using the coincident site lattice theory (CSL),^361^ with several Σ-GBs examined as representative cases. Early work by Freeman and co-workers focused on comparing H binding energy of iron^352^ or nickel^353^ GBs to energy at corresponding separated surfaces, aiming to characterize the embrittling effect at GBs. These studies are further detailed in Section 2.3.5 as supporting evidence for the HEDE mechanism. Using DFT, Du et al.^362^ studied H interactions with Σ3[1–10](112) and Σ5[001](310) GBs in *bcc* iron and found the lowest binding energy for both cases were −0.43 eV. Additionally, they noted that these GBs acted as H traps without providing rapid diffusion pathways. Yamaguchi^363^ also reported a similar binding energy of −0.45 eV for the Σ3[1–10](112) incoherent twin GB in iron. While experimentally measured segregation energies of H at iron GBs are not precisely known (ranging from −0.10 to −0.61 eV), they are larger than the free surface energy (e.g., −0.91 eV). In nickel, the behavior of H trapping and diffusion at GBs can differ markedly. Stefano et al.^329^ analyzed two types of nickel GBs: Σ3[1–10](111) with a close-packed interface structure and Σ5[001](210) with more open structural units. They found that the Σ3 GB, with a binding energy of −0.01 eV, neither traps H nor facilitates its diffusion, whereas the Σ5 GB, with a binding energy of −0.23 eV, offers fast diffusion channels along the GB plane. Zhou et al^350^ concluded that low-angle GBs tend to hinder H diffusion, whereas high-angle GBs promote it.

Although DFT calculation has been instrumental in studying H trapping at GBs, its application has predominantly been confined to a limited number of GB types. Recently, empirical potentials, which balance the aspects of cost and accuracy, have provided a possibility for investigating H–GB interactions at multiple GBs^364^ and even in a polycrystalline GB network. Utilizing the EAM potential^360^ and a thermal-equilibrium polycrystal model, Ding et al.^365^ computed a spectrum of H binding energies in polycrystalline nickel, illustrated in Figure 17c. This spectrum revealed three distinct peaks, each corresponding to different structural characteristics. Notably, the first peak, with a binding energy of −0.205 eV, signifies the crucial H trapping sites at the GB core, which is in close agreement with the experimental value of −0.214 eV.^366^

Free surfaces, which may form due to the separation of a GB or the formation of a microcrack, are known to be strong trapping sites for H. Jiang et al.^337^ utilized DFT to study H adsorption and absorption on (100) and (110) surfaces in *bcc* iron, as shown in Figure 17d. They found that H prefers to stay on the iron surface rather than in the subsurface or bulk, with the lowest binding energies identified as −0.58 eV and −0.91 eV for the (100) and (110) surfaces, respectively. Ferrin et al.^82^ expanded this analysis to include the interaction of H with various facets of 17 transition metals, noting a significant difference in binding energies between close-packed and more open (100) facets within the same material. For example, the binding energies for (100) and (111) surfaces in nickel were −0.60 eV and −0.73 eV, respectively. Additionally, the interaction of H with free surfaces created by GB separation has been studied.^352,353^ While the binding energy varies across different types of free surfaces, it is generally lower than that of intact GBs or the bulk. Therefore, free surfaces are likely the most favorable sites for H accumulation.

Precipitates are well-established trapping sites for H and have been extensively studied with atomistic simulations. Counts^325^ employed DFT to study the interaction of H with various substitutional solutes in iron, including Mg, Al, Sc, Ti, V, Ni, Cu, Zn, Y, Nb, and Cd. These solutes were found to trap H with negative binding energies, some as low as −0.25 eV. Conversely, for Si, Cr, Mn, Co, and Mo, the lowest binding energies were zero or positive, indicating a lack of interaction or even repulsion with H. The interaction between H and these substitutional solutes appears to be roughly correlated with the electronegativity and size of the solutes. Ito et al.^367^ showed that a small amount of Mo segregation to α-Fe GBs significantly reduced the concentration of H, owing to the repulsion between Mo and H. A similar trend was observed in nickel by He et al.,^294^ where the concentration of H near Mo solutes was lower compared to the bulk.

Moreover, external loading can significantly affect H trapping properties. With DFT, Drexler et al.^368^ estimated that in nickel, a volumetric strain of approximately 20% near a crack tip can effectively create a binding energy of around −0.37 eV. This value is comparable to the trapping strength exhibited by vacancies.

##### 2.2.4.4 Summary of H Mapping through Atomistic Simulations

Quantum mechanics-based calculations, predominantly DFT and empirical potential-based approaches, have facilitated a quantitative assessment of the tendency of H binding to the bulk and various microstructural defects. Summarizing the findings, the preference of H for different accommodation sites can be ranked as follows: free surfaces > vacancies and GBs with open structures > dislocations, GBs with compact structures, and precipitates > bulk. These atomistic calculations are instrumental in interpreting experimental results or directly determining the solution energy of specific sites that are challenging to measure experimentally.

However, a major drawback of these calculations is their high computational cost, limiting simulation sizes to just a few nanometers, which is insufficient to capture the complex interactions between H and realistic microstructures. Another concern is the simplifications and approximations inherent in these simulations, which can lead to deviations from actual microstructures and raise accuracy issues. Even with the precision of DFT, calculated binding energies for the same microstructural defect can differ by as much as 0.4 eV,^352^ a significant variance for a quantum particle like H. Therefore, the configurations of an atomistic model should be aligned with experimental calibration or validation to avoid oversimplifying real-world materials. More about the verification of an atomistic simulation is discussed in Section 3.5.

#### 2.2.5 Laboratory H Charging Methods

Laboratory testing of H induced fracture is indispensable for mechanistic understanding and engineering failure assessment of HE. Any laboratory test of H-induced fracture consists of two phases, H charging and mechanical testing. H charging is less understood and more difficult to control and is therefore often the limiting factor. Electrochemical charging and gaseous charging are the two methods of introducing H into a test specimen. As elaborated in Section 2.2.1, H ions are supplied in an electrolyte in the former method, while H atoms are generated by dissociation of H_2_ in the latter. While gaseous charging is more relevant under the context of H gas transport pipelines, electrochemical charging is more efficient, much cheaper and faces less regulatory barriers (safety). Therefore, industrial practice favors electrochemical charging as a tool for routine testing or preparatory testing before industrial level applications. In most cases, lab work cannot directly represent the real application because of the discrepancy in the H source, charging parameters, sub-surface H concentration, surface condition, stress distribution, nature of defects, microstructures, etc. In addition, the transferability from laboratory testing to large scale components can be challenging, which requires systematic investigation and validation. In this section, challenges brought by different lab H charging methods and the transferability to real application are discussed.

##### 2.2.5.1 Overview of the Two H Charging Methods

As discussed above, the majority of lab charging uses electrochemical H charging cells, which consist of a working electrode (the specimen), a counter electrode (Pt wire), and a reference electrode in an electrolyte. The electrolyte with different pH values in acidic (H_2_SO_4_, H_3_PO_4_, pH ≈ 1), alkaline (NaOH, pH ≈ 13), and neutral (NaCl, pH ≈ 7) solutions can be used.^58,118,369^ Typically, HE is more severe in the acidic electrolyte,^369^ where H evolution reaction is promoted by decreasing the pH value.^370^ Unlike gaseous H charging, electrochemical charging can change the microstructure and alter the diffusivity and solubility in a specimen. For instance, cathodic H charging induced hydride formation and martensitic phase transformation were reported on Ti alloys,^18,371^ Zr alloys,^372^ and high-entropy alloys.^373^ The difference in H diffusivity between the new phases and matrix accelerates H absorption and is deemed to potentially alter the HE mechanism. Further, cathodic H charging can induce surface cracking in *fcc* metals as a consequence of internal stresses associated with H concentration gradient.^200,201,374,375^ The internal stresses can alter the subsurface lattice arrangement and possibly influence H dissolution and diffusion. H-induced blisters with high-pressure H gas inside are often observed in *bcc* metals.^369,376^

Compared to gaseous H charging, electrochemical charging can also deteriorate the oxide film on the specimen and promote redox reactions.^377^ These microstructure changes can effectively influence H uptake, diffusion, and trapping, which causes complexity to the interpretation of HE mechanisms and raises questions regarding the transferability from electrochemical charging to gaseous H environment which is more relevant to engineering applications.

Gaseous H charging is an important yet less widely applied approach to study HE of a material. Due to safety issues and complicated setup, only a few labs have the capability to conduct high pressure H gas test, e.g., *in situ* slow strain rate tensile (SSRT) test and fatigue test. The testing pressure typically ranges from several MPa up to 100 MPa, with the temperature varying from −80 °C to several hundred degrees. As an alternative to high pressure H gas test, hollow specimen tests were developed to quantify material ductility loss from both external and internal H sources, which is elaborated in Section 2.2.5.3. Compared to high pressure H gas condition, hollow specimen requires less amount of H up to 20 MPa, which is safer and more cost efficient. However, how to precisely control the inner surface condition remains a critical issue when analyzing the results.

##### 2.2.5.2 Gap between Laboratory Charging and in-Service Conditions

Accurate measurement of H diffusion rate at ambient and low temperatures can be challenging, especially for *fcc* metals with extremely low H diffusivity (10^–13^–10^–15^ at 23 °C).^272,291^ For instance, it may take several weeks to obtain a single permeation curve at room temperature on a nickel foil with only 100 μm thickness, while this can be done within several days at a higher temperature and with a larger current density since H uptake can be remarkably promoted at elevated temperature^272^ and current density.^272,378^ Therefore, most of laboratory H charging tests are conducted with harsher charging conditions than in-service ones in order to accelerate the tests to be done within a reasonable time. Then the test data have to be transferred from the harsher conditions to the conditions of practical interest in order to be exploited. This leaves a gap between lab test data and engineering applications, which has to do with a wide range of H charging parameters, including temperature, loading condition, current density, metal surface condition, and possible corrosion product as inhibitor or accelerator to H uptake.

While laboratory H charging is usually conducted on virgin specimens without external load,^50,198,260^ the harsh electrochemical H charging in acidic electrolyte can induce a microstructural change close to the H–metal interface, such as phase transformation or even surface crack; this has an impact on H adsorption and absorption kinetics and diffusion properties, leading to uncertainties of experimental measurement. Furthermore, a structural component in engineering application is inevitably subjected to stresses induced by complex service load, which triggers microstructural changes, e.g., through the multiplication of dislocations and formation of vacancies, and leads to a substantial alteration of H diffusivity.^142,273,278^ The uncertainties with microstructural changes in the laboratory test severely undermine the engineering transferability to real applications.

In service, H is continuously supplied by the working environment over a long period. While laboratory charging is conducted within a relatively short time, either *ex situ* or *in situ*. H diffusion pathways can be quite distinct between very slow uptake and fast charging, and between *ex situ* and *in situ* charging. Some engineering components are subjected to dynamic service load, which makes the H uptake and diffusion more difficult to track and deviates more from the laboratory charging conditions. In addition, the formation of corrosion product on the surface of structural components in subsea application further complicates the situation.^379,380^

At a macroscopic scale, the presence of stress concentrators introduced during the manufacturing and machining of engineering components, e.g., cracks, thread roots, or weldments sharp edges can accumulate and trap H and cause inhomogeneous H distribution. While in the laboratory, only a small piece of material is tested after careful surface preparation. It is questionable if the models developed and the parameters calibrated for H uptake and diffusion in the laboratory can be applied to engineering failure assessment of real structures.

The aforementioned factors are always intertwined in engineering application, which makes it extremely challenging to distinguish the main parameters to control in the laboratory to reproduce the in-service conditions for H uptake and transport. To fix the gap between laboratory charging and engineering applications, it is necessary to develop a test protocol detailing the laboratory H charging procedure and assessing the transferability of the lab test data to components under service conditions.

##### 2.2.5.3 Correlation between Electrochemical and Gaseous Charging

To apply electrochemical charging to “mimic” gaseous charging and further apply the test results in engineering components, e.g., H pipelines, a mapping between the two charging methods must be drawn. Establishing this mapping allows for accurate prediction and comparison of material behaviors under different H exposure conditions, thus facilitating the cross-application of research findings among different H-related areas. This involves finding the right electrochemical charging current density or overpotential that introduces the same amount of H into a specimen as a given gaseous H pressure. The comparison is not just about matching quantities of H; it’s also about understanding how each method affects the material. For instance, does electrochemical charging alter the microstructure differently than gaseous charging? This is a key consideration since changes in microstructure can influence how materials react to H, potentially affecting their performance in real-world applications.

The concept of fugacity acts as a critical bridge in establishing correlation between gaseous and electrochemical H charging methods. It functions as an effective activity level indicator of H within a system, offering a quantifiable means of correlating macroscopic charging parameters with the microscopic behavior of H atoms in the metal lattice. When two different charging methods result in the same H fugacity within a material, they can be considered equivalent in terms of their potential to induce material changes, such as embrittlement. This correlation is quantitatively established through the correlation of gaseous H pressure *p*_H_2__ and electrochemical overpotential:
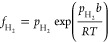
25
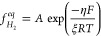
26where *b*, *F*, *ξ*, *A*, and *R* are constants, and *T* is temperature. Eq 25 gives the relation between fugacity and H gas pressure; eq 26 defines a so-called equivalent H fugacity, *f*_H_2__^eq^, during electrochemical charging, and correlates it to the overpotential. This equation is crucial for establishing the correlation between the two H charging methods.

H permeation testing and TDS are the two experimental approaches employed to determine *f*_H_2__^eq^. Permeation tests provide a means to determine *f*_H_2__^eq^ by comparing the permeability coefficients obtained with the two charging methods;^381^ these tests can also be applied to determine *f*_H_2__^eq^ directly based on eq 26 in conjunction with thermodynamic calculations.^114^ Recently, a correlation between gaseous and electrochemical charging on a X65 pipeline steel was established using this methodology.^118,382^ Alternatively, the equivalent H fugacity can be determined with TDS by comparing the H concentration in gas-charged and electrochemically charged samples.^383,384^

Several challenges must be noted in the experimental calibration of such a correlation between electrochemical and gaseous charging. A critical issue is to maintain stable surface conditions during charging, which is crucial for accurate assessment of H uptake and diffusivity via permeation techniques. During electrochemical charging, factors such as the presence and alteration of the oxide layer, the formation of corrosion products, and the excessive evolution of H bubbles can substantially affect the accuracy of measurement. Additionally, aggressive conditions in electrochemical H charging, for instance, high overpotential or elevated current density, can induce damage such as blisters and cracks. The specimen used for gas permeation is often electroplated with Pd on the charging side to overcome the surface impedance which can be caused by an oxide layer, but the influence of the Pd coating on H uptake and diffusion has not been adequately evaluated. Considering the sub-surface lattice H concentration as a reflection of charging severity offers a way to compare uptake from different H sources. However, accurate measurement of the sub-surface concentration is challenging because of H trapping which affects both H uptake and diffusivity. Despite these challenges, the development of a correlation between gaseous and electrochemical H charging is an emerging and significant area of HE research.

#### 2.2.6 Accommodation of H in Engineering Alloys

Enormous studies have been conducted to characterize the accommodation of H at different microstructural sites in steel and nickel alloy, providing important reference for the mechanistic understanding, prediction, and mitigation of HE.

The mechanical property of a steel is highly dependent on the microstructural phases it possesses, e.g., austenite, ferrite, martensite, bainite, etc. The behavior of H in these phases varies to a large extent. H diffusivity can range from 10^–10^ to 10^–15^ m^2^/s in these phases, being very small in *fcc* phases and relatively large in *bcc* phases. The solubility of H, on the contrary, is small in *bcc* phases and very high in *fcc* phases. Therefore, the fraction and distribution of the phases have a significant influence on the distribution of H in steel.

In addition, grain size, characteristics of GBs, and precipitation all influence H trapping and diffusion properties, as detailed in Section 2.2.2.7. Alloying elements in steel, such as N, B, P, Zr, Cr, Cu, and Mn, typically reduce the effective H diffusivity (*D*_eff_) and increase H solubility.^385−389^ N at a concentration higher than 0.38 % in an austenitic steel decreases H diffusivity because N can be an effective H trap with a binding energy of about 25 kJ/mol.^387,390^ B, Zr, and Cr have a similar effect as that of N.^385^ B and P segregation to GBs increases the H binding energy to an extent that *D*_eff_ is reduced.^386^ The addition of Cu in a low carbon low alloy steel was reported to increase the content of retained austenite and retard H diffusion.^387^ In addition, Mn, Mo, and C were demonstrated to slow down H uptake because the energy barrier of H diffusion from surface toward the bulk was increased.^391^ The role of Ni as an alloying element in steel is debatable. Ni was reported to increase the apparent H diffusivity,^392^ while a recent study on a quenched and tempered Ni-containing steel demonstrated that 5 wt. % addition of Ni decreased *D*_eff_ by a factor of two;^393^ Ni was reported to reduce *D*_eff_ in ferritic/pearlitic low alloy steels in another study.^394^

Through modification, or engineering, of grains and GBs, H diffusivity can be tuned in steels. An Fe-25Ni-15Cr austenitic steel with ultrafine grain size of about 100 nm, produced by high-pressure torsion, showed increased H diffusivity and permeation rate through short-circuit diffusion facilitated by the increased number of GBs.^395,396^ At elevated temperature, however, H diffusivity does not depend on the grain size.^397^ There seems to be a grain size limit below which H diffusivity does not increase further. For X70 pipeline steel, the value is ∼46 μm as suggested by a two-dimensional diffusion model.^398^ For materials with nanosized grains (∼10 nm), caution should be paid to the trapping effect of triple junctions, while this is negligible for coarse grains.^399^ Special GBs such as Σ5, Σ9, and high angle symmetric tilt GB are preferential trapping sites for H.^397^ In this scenario, there is a competition between short-circuit diffusion that tends to increase H diffusivity and GB trapping that tends to do the opposite. Moshtaghi et al.^400^ observed in the Ni-added steel that H diffusivity decreased as grain size decreased, since H trapping at GBs dominated over the short-circuit diffusion mechanism.

Some alloying elements promote the formation of secondary phase precipitates, which can be effective H traps and consequently reduce *D*_eff_ drastically. Fine copper precipitates (∼10 nm),^401,402^ vanadium carbide particles (<60 nm),^181^ TiC carbide (5–10 nm),^403^ coherent η-Ni_3_Ti precipitates,^404^ and nano-sized (Nb, Ti)C precipitates^405^ can trap H and lower H diffusivity in steels. Meanwhile, it should be noted that the H trapping capacity of a specific type of precipitate depends on its structure, size, and coherency. MC (M representing V, Mo, Nb) carbides can trap H, while M_6_C (M representing Cr, Mn, Mo) cannot because of the low affinity of Cr for H in M_6_C.^406^ In comparison, GB carbides are obstacles to fast diffusion of H, thereby reducing H absorption.^407^

Similar to precipitates, micropores and nano-vacancies can be strong H traps with a binding energy of 40–70 kJ/mol.^325,408−410^ However, it has been shown that a high concentration of vacancy is necessary to have a remarkable effect on H diffusion.^411^ To control H content under a critical value, both trapping and permeation energies of H in the constituent phases should be accounted for.^213^

Nickel and its alloys have an *fcc* structure and very different H solubility and diffusivity compared to *bcc* materials. H solubility in α-Fe is much lower than that in pure Ni, while H diffusivity in α-Fe can be five orders of magnitude higher than that in pure Ni.^271^ Due to the extremely low diffusivity of H in the lattice of nickel and its alloys, the effect of GB on H diffusion stands out. The role of GBs during H transport in nickel alloys is two-fold. Oudriss et al.^260,291^ conducted permeation test and EBSD measurement, and showed GBs with low misorientation act as H traps, due to the defect accommodation. Meanwhile, random GBs, i.e., high-angle boundaries, were shown to accelerate H diffusion by acting as short circuits. Whether a GB acts as a trap or fast diffusion pathway for H depends on its type and feature. To make it more complex, a high-angle GB in nickel can act not only as a diffusion highway but also as an H trap at the same time, as demonstrated by DFT calculations.^164^ A Σ5 type GB was shown to provide favorable trapping sites for H within its open structural units as well as easy migration pathways for H along the GB plane. The close-packed Σ3 GB, on the other hand, was found neither to trap H nor enhance its diffusion, but instead acted as a two-dimensional diffusion barrier to H. Therefore, it is crucial to give an adequate account of the size, type, and energy status of GBs when assessing their effects on H diffusion and trapping in nickel and its alloys.

Similar to the effect of secondary phases in steels, precipitation by heat treatment typically enhances H trapping and suppresses H diffusion in nickel alloys. Such precipitates include coherent γ′ (Ni_3_Al), δ, σ, and carbides.^171,412,413^ TDS analysis can be applied to measure the binding energies of these phases; a permeation test can help to assess the influence of these phases on the diffusion of H.^296,414^ These trapping sites at precipitation phases in nickel are usually classified as reversible trapping sites in comparison to the high binding energies of carbides.^13^ However, NanoSIMS observations by Zhang et al.^15^ showed that *δ* phase adsorbs more H than carbides and nitrides despite its possibly lower binding energy. Therefore, the binding energy may not be the sole parameter to distinguish a reversible and an irreversible trap. These authors also demonstrated that H is trapped within the precipitates rather than at the interfaces between them and the matrix.

As is evident from the above discussions, H transport behavior in steels and nickel alloys is strongly dependent on the nature of the material, in particular, microstructure. *fcc* alloys possess a high H solubility but a low diffusivity; on the contrary, *bcc* alloys exhibit a high H mobility and a low solubility, rendering these two categories of material quite different in H accommodation properties. A summary of the trapping capacity of various H trapping sites in steels and nickel alloys is shown in Table 3. Permeability, diffusivity, and solubility parameters calibrated for a number of steels and nickel alloys are summarized in Table 4.

**Table 3:** Summary of Typical H Trapping Sites and the Binding Capacity in Iron and Steels, and Nickel and Its Alloys

Trapping sites	Detrapping/binding energy (*E*_d_/*E*_b_, kJ/mol)	Detection method	Ref
*bcc* Iron and Steels			
GB	17.20–19.68 (*E*_d_)	TDS	(241, 415)
Dislocation	26.04 (*E*_d_)	TDS	(241)
Elastic field around dislocation	0–20 (*E*_d_)	TDS	(416)
Dislocation core	58.60 (*E*_d_)	TDS	(416)
Iron oxide (Fe_2_O_3_, Fe_3_O_4_)	50.60 (*E*_d_)	TDS	(417)
	47.20 (*E*_d_)	TDS	(418)
Gangue inclusion (SiO_2_)	112.10 (*E*_d_)	TDS	(417)
Microvoid	35.20 (*E*_d_)	TDS	(241)
	48.3 (*E*_d_)	TDS	(419)
Fe_3_C interface	19 (*E*_d_)	TDS	(416)
TiC (incoherent)	68–116 (*E*_d_)	TDS	(420)
	145 (*E*_d_)	TDS	(421)
TiC (semi-coherent)	55.8 (*E*_d_)	TDS	(420)
NbC (incoherent)	63–68 (*E*_d_)	TDS	(422)
NbC (semi-incoherent)	28–56 (*E*_d_)	TDS	(423)
Ferrite-cementite interface	18.4 (*E*_d_)	TDS, permeation	(424)
MnS interface	72.30 (*E*_d_)	TDS	(419)
Al_2_O_3_ interface	79 (*E*_d_)	TDS	(415)
Austenite-ferrite interface	52 (*E*_b_)	Permeation	(425)
			
Nickel and Nickel Alloys			
Elastic field around edge dislocation	33.77 (*E*_d_)	TDS	(291)
Dislocation core	54.99 (*E*_d_)	TDS	(291)
GB	22.19–29.91	DFT, tilt ∑5(012)	(294, 353, 426)
		TDS, permeation	(272, 296)
	15.44 (*E*_b_)	DFT, twist ∑5(001)	(293)
	20.26 (*E*_b_)	TDS, general GB	(366)
γ'	30.87–36.66 (*E*_b_)	Permeation	(413)
	19.30 (*E*_b_)	NRA	(427)
γ′′	23.15–27.01 (*E*_b_)	Permeation	(413)
δ	29.90 (*E*_b_)	Permeation	(413)
M_23_C_6_	19.29–27.98 (*E*_d_)	TDS	(428)
σ	69.47 (*E*_b_)	TDS	(171)
Microvoid, H at surface	57.89 (*E*_b_)	DFT, (012) free surface	(294)
	55.96 (*E*_b_)	DFT, (001) free surface	(293)

**Table 4:** Summary of Recommended Permeability, Diffusivity, and Solubility Relations for Pure Iron, Austenitic Stainless Steels, and Nickel Alloys^a^

	Permeability		Diffusivity		Solubility		
Material	Φ_0_	*H*_Φ_	*D*_0_	*H*_D_	*S*_0_	Δ*H*_S_	Ref
Pure iron	5.35 × 10^–5^	33.6	6.20 × 10^–8^	10.5	863	23.1	(429)
Austenitic stainless steels (301, 302, 304, 310)	5.35 × 10^–3^	56.1	2.01 × 10^–7^	49.3	266	6.9	(430)
316L stainless steel	8.80 × 10^–4^	66.5	1.30 × 10^–6^	54.0	714	12.5	(431)
Ni	7.08 × 10^–4^	54.8	7.43 × 10^–7^	44.1	953	10.7	(432)
Inconel 600	9.50 × 10^–4^	66.2	1.36 × 10^–7^	37.7	1980	28.5	(432)
Hastelloy X	8.92 × 10^–4^	64.6	4.90 × 10^–7^	43.4	1830	21.2	(431)

a Note: Φ_0_, *D*_0_, *S*_0_ and *H*_Φ_, *H*_D_, Δ*H*_S_ are pre-factors and activation energies for calculating permeability, diffusivity, and solubility. Φ ≡ *DS*. Units: .

The H trapping and diffusivity properties in these engineering alloys can be controlled by tuning the microstructure of the material, through the following techniques as a brief summary: heat and mechanical treatment, microalloying, GB engineering, and surface treatment, etc. The aim of these techniques is to lower H absorption, slow down H diffusion, and enhance H trapping capacity. This reduces the amount of diffusible H and increases the resistance to HE. More details about mitigation strategies for HE are presented in Section 3.3.

### 2.3 HE Mechanisms

The history of the development of the HE mechanisms targeted in this Review has been briefly introduced in Section 2.1.1. Over the past decades, the debate on the HE mechanisms has never ended, but there has been a clear shift in the emphasis. Until the 2000s, the debate had been about which mechanism is dominant, and there seemed to be a presumption that there existed a unified theory that could conveniently rationalize most of HE phenomena. Since the 2010s, it has become widely accepted that HE is so complex that it cannot be accounted for with a single theory, and several mechanisms can operate at the same time, depending on the material, environmental, and loading conditions. A seminal work in this regard is the introduction of the so-called H-enhanced and plasticity-mediated decohesion mechanism. The proposal is based on a series of experimental studies in a variety of materials revealing that H-enhanced plasticity is a necessary precursor to the HEDE mechanism. FIB lift-out and high-resolution SEM were the enabling techniques for this discovery. Nowadays, the debate is still ongoing but zooms into the influence of H on very specific microstructural features or processes. For example, instead of putting the influence of H on a generic term of dislocation activity as the HELP mechanism does, atomistic investigations into the influence of H on elementary processes like the movement of a dislocation segment (mobility), the activation of an FR source (multiplication), and the emission of a dislocation from a crack tip were carried out. Investigations dedicated to the cases of a pure edge dislocation and a pure screw dislocation were also conducted. With the study becoming more sophisticated and case-specific, it is natural to expect new or even conflicting insights to be revealed, which poses a challenge to existing HE mechanisms. We anticipate that HE mechanisms will evolve along with new research findings to become more precise and rigorous, which is beneficial for the categorization and practical application.

Following the above overview, we organize this section as follows. Before delving into the mechanisms of HE, we first make a clarification of the terminology frequently encountered in this section. Then we will present a comparative study among the HE mechanisms, with a clear focus on the controversial and unresolved issues. This comparative study includes original reflections and strives to present objective, and where necessary, critical comments. Further, we categorize the theories with respect to material systems, mainly steels and nickel alloys, and give a state-of-the-art review on the key experimental techniques that help to advance the theoretical development in this field. Atomistic insights that contributed to deepening the theoretical understanding on specific effects exerted by H will also be presented, followed by a section dedicated to mesoscale modeling techniques that enable the understanding of HE mechanisms from a scale closer to practical applications.

#### 2.3.1 Clarification of Terminology

##### 2.3.1.1 Dislocation Activity

The interaction between H and plasticity is a key aspect of HE. Except for HEDE, all the mechanisms to be reviewed subsequently have to do with the influence of H on dislocation activity, emphasizing on different aspects of the interaction including dislocation nucleation or emission, dislocation multiplication, dislocation mobility, and dislocation entanglement.

A dislocation can be created from a perfect bulk material or free surface, by producing a disorder in the arrangement of atomic lattice, and this is termed dislocation nucleation. A dislocation that is nucleated can readily move away from the nucleation site under applied load, and the combined process of the nucleation and the movement is termed dislocation emission. Dislocation emission is employed in the AIDE mechanism specifically for the combined process happening at a free surface, while the term dislocation nucleation is frequently used for the bulk case such as in a nanoindentation experiment. However, there are a few cases where the term dislocation emission is also adopted for the bulk case.

A dislocation can also generate from a short dislocation segment that already exists. Upon loading, such a segment can bow out, elongate, pinch off, and bow out again, thus repeatedly generating more dislocations; this process is called dislocation multiplication and the original segment a dislocation source. A dislocation source is usually pinned at its ends or has one end connected to a free surface, such that the source itself is not easily annihilated during the multiplication. In the former scenario, it is called a Frank-Read (FR) source and in the latter, it is called a surface source. The critical event for dislocation multiplication is the bow-out of a dislocation source, usually if the source is able to bow out beyond a certain extent, the multiplication is activated and can go on for several rounds. This event is therefore termed the activation of a dislocation source, and the main resistance to this activation is the line tension of the dislocation which is proportional to the core energy of the dislocation.

The term dislocation mobility describes the readiness of a dislocation to move and the speed at which a dislocation moves. Assuming an edge dislocation that is arrested in a stress field, one can calculate at what external load it can break away and continue to move; after it starts to move, one can also estimate its speed. The same applies to a screw dislocation or a mixed dislocation. Dislocation mobility can be easily confused with the activation of a dislocation source, but these are fundamentally different. Dislocation mobility describes the readiness of a dislocation segment to move, this dislocation is free from pinning at either end, and it keeps its original characteristic angle (angle between the dislocation line and its burgers vector) length even after it starts to move. The activation of a dislocation source is about the readiness of a dislocation segment to bow out, this dislocation (e.g., an FR source) is pinned at both ends, and it elongates and develops new segments with various characteristic angles once it starts to bow out. Obviously, activation of a dislocation source and dislocation mobility are both factors influencing dislocation multiplication.

Finally, dislocations belonging to different slip systems can encounter each other and form a dislocation junction which is immobile. Dislocation junctions then anchor more dislocations and form dislocation entanglement, which can be an embryo of dislocation substructure. Activation of a dislocation source and dislocation mobility are factors influencing the formation of dislocation entanglements.

##### 2.3.1.2 HE Fractography

There is a general consensus about metallic materials that a fracture surface produced at room temperature and in the absence of H displays ductile features, such as obvious dimples and noticeable plastic tearing ridges. It is also widely accepted that the presence of H makes the fracture surface appear more brittle. However, there exists a substantial diversity in the description of experimentally observed morphology of fracture surfaces in the presence of H. The terms that have been employed to describe an “H embrittled” fractography include TG fracture, IG fracture, cleavage, quasi-cleavage, and “flat featureless surface”. These terms are to a certain extent overlapping and are sometimes used interchangeably even erroneously, which adds unnecessary complexity to the study of HE. Therefore, it is helpful to make a clarification on these terms before unravelling further the complex nature of HE.

As mentioned, the fractography of ductile fracture is characterized by plastic tearing ridges and/or dimples associated with microvoids. The discussion of these features usually does not concern GBs, as ductile fracture of most metallic materials relevant for H application is TG at room temperature. Failure is initiated from the interior of a grain, a microcrack then propagates across a grain, possibly transmitting a GB and then propagating across another grain. If omitting secondary local events of GB transmission of the microcrack, ductile fracture can be analyzed with the same continuum approach, such as the Gurson model, no matter the sample is a single crystal or polycrystal. In this sense, when talking of a TG fracture, the focus is on what happens to the atomic lattice of the interior of a grain, or a single crystal.

Strictly speaking, the fractography of brittle fracture is generated by a different failure mechanism and is therefore free from ductile features such as plastic tearing or dimples, which in the ideal situation, manifests as a “flat featureless surface”. A typical case of brittle fracture with such a surface in metallic materials is cleavage which occurs at a sufficiently low temperature. Cleavage is defined as a brittle TG fracture by separation across well-defined crystallographic planes. In *bcc* materials, such as iron and ferritic steel, brittle fracture usually occurs on the {001} planes at low temperatures, which is a typical example of cleavage, {110} and {112} planes are also potential cleavage planes for *bcc* iron^433^ but are less frequently seen. The cleavage plane of a crystal is known a priori, the direction and angular relationships between cleavages give valuable hints about atomic arrangement of crystals. Obviously, cleavage is defined in a single crystal, but in a polycrystalline metallic material, such as ferritic steel, it can also occur, only on the cleavage plane of each individual grain. Two remarks can be made on cleavage, that it is a TG fracture, and that it has to occur on specific crystallographic plane(s).

Quasi-cleavage is a term defined to depict a type of TG fracture surface in the presence of H. Such a fracture surface has much less dimples or tearing ridges compared to that without H, it can even appear featureless under microscopy at a low magnification. This type of TG fracture surface is similar to cleavage in morphology, however, it is essentially different from cleavage, in that it does not occur on any of the crystallographic planes that define cleavage. Although widely adopted, quasi-cleavage is perhaps not an appropriate term for H embrittled TG fracture surface. Quasi-cleavage cannot approach cleavage, no matter how H concentration and the level of embrittlement increase, because the two failure modes are fundamentally distinct. This aspect has been highlighted in the literature; as a matter of fact, Merson et al.^433^ used the term “true cleavage” to highlight the difference between cleavage and quasi-cleavage in their work.

It is important to note that the fracture modes discussed so far are all TG fracture. In some materials and when the concentration of H is sufficiently high, IG fracture can occur. In this scenario, fracture initiates and propagates along GBs in a crystalline material. An IG fracture surface usually appears more brittle than quasi-cleavage. The fracture surface can display as featureless flat facets under low to medium magnification microscopy.

#### 2.3.2 A Comparative Study of HE Mechanisms

##### 2.3.2.1 HEDE Mechanism

The HEDE mechanism assumes that H absorbed in metallic materials reduces the cohesive strength of crystalline lattice. It was first postulated by Pfeil and then developed by Troiano, Oriani, and Gerberich.^26^ This consequently leads to premature material separation before reaching the critical load for an H-free material. Fracture and more specifically, brittle fracture, is a natural outcome of the operation of HEDE; therefore, this mechanism is frequently invoked in phenomenological modeling as detailed in Section 2.3.6.1.

The HEDE mechanism is supported by a number of theoretical and simulation studies. Hoagland and Heinisch^434^ adopted a single H interstitial model to investigate crack propagation in nickel. When the model is oriented such that dislocation emission is difficult, H was observed to facilitate crack propagation by modifying the hydrostatic component of the dilatational stress field of the crack. Jiang and Carter^324^ conducted first principles calculation of ideal fracture energy of in the presence of H, using iron and Al as model materials. They found that the ideal fracture energies decreased almost linearly with increasing H coverage, dropping by almost a half at a coverage of 0.5. It is worth noting that H coverage was employed to quantify the amount of H, so the study investigated the influence of adsorbed H at an interface. According to the Langmuir–McLean isotherm, a coverage of 0.5 corresponds to a very high bulk H concentration that is absorbed in the lattice. Katzarov and Paxton^435^ combined the thermodynamic–kinetic continuum scale cohesive zone model and the quantum-mechanical magnetic tight-binding approximation to interatomic forces, and studied the decohesion process in time as traction was applied to an H charged crystal. Decohesion was found to occur between two (111) crystal planes in iron. This allowed for a direct investigation on the cohesive strength of the interface. The cohesive strength was found to be reduced from 33 GPa to 22 GPa due to the presence of dissolved H. The reduction was unaffected by a change in temperature from 300 K to 200 K and was independent of the bulk H concentration in the range of 0.1 appm to 10 appm. It should be noted that the cohesive strength predicted for an otherwise perfect lattice in this study is extremely high.

The HEDE mechanism is often questioned in two aspects. It is questionable whether the very high lattice concentration needed to trigger decohesion is attainable in reality.^436^ Another question is about the very high cohesive strength of a perfect lattice populated with H. It is suspected that the material could have failed by another process with a lower threshold, before such a high cohesive stress is actually built up.^23^ Consequently, it is often assumed that the HEDE mechanism acts upon impurity interfaces instead of a perfect lattice in a material, such as GBs or phase interfaces, where the cohesive strength is reasonably low and the concentration of H can be sufficiently high due to H trapping.

##### 2.3.2.2 AIDE Mechanism

The AIDE mechanism deals specifically with dislocation emission from a free surface. It claims that H adsorbed onto a free surface, which can be the free surface of a sharp crack or the inner surface of a nanovoid, facilitates the emission of dislocations from the surface. This involves the simultaneous formation of a dislocation core and surface step by cooperative shearing of atoms (breaking and reforming of interatomic bonds) over several atomic distances, which is facilitated by adsorbed H through weakening interatomic bonds. The operation of the AIDE mechanism naturally leads to the advancement of a crack front. Compared to direct decohesion, it is usually easier to emit a dislocation, so the threshold for the operation of the AIDE mechanism is lower than that for the HEDE mechanism. In the model developed by Hoagland and Heinisch,^434^ an H interstitial was observed to assist dislocation emission instead of decohesion when the model is oriented to allow for easy emission. While based on enhanced dislocation activity, the AIDE mechanism predicts a decreased level of plasticity in front of a crack tip, as compared to the ductile crack growth with crack tip blunting in the absence of H. According to Lynch,^436^ ductile crack growth occurs predominantly by the multiplication of dislocations from sources in the region ahead of a crack, with little or no emission of dislocations occurring at the crack tip. In this scenario, most of the dislocations move and contribute to the plasticity ahead of the crack tip, while few of them intersect exactly with the crack tip and cause a deformation step. With the AIDE mechanism, a larger number of dislocations are emitted exactly at the crack tip, and a greater proportion of dislocation activity results in crack growth because dislocation emission on suitably inclined slip planes produces crack advance as well as crack opening. This decreases the plastic strain near the crack tip and limits the size of plastic zone.^22^

The AIDE mechanism phenomenologically resembles the HEDE mechanism. A common misunderstanding of the AIDE mechanism is that H increases local plasticity ahead of a crack tip because of the more dislocations emitted; this arises because of confusion of crack tip dislocation emission with dislocation nucleation/emission in the bulk. Dislocations emitted in the bulk induce plastic strain as they move, increasing plastic dissipation ahead of a crack tip. The contribution to plasticity ends as soon as a dislocation exits from a free surface and leave a deformation step there. Therefore, H enhances local plasticity ahead of a crack tip only if it facilitates dislocation multiplication and movement in the bulk, which is not considered by the AIDE mechanism.

##### 2.3.2.3 HESIV Mechanism

The HESIV mechanism states that absorbed H atoms facilitate the formation of nanovoids induced by plastic straining, which is possibly achieved by H stabilizing vacancy clusters. Failure can be induced by the coalescence of these voids or the decohesion of the ligament between the voids.

The HESIV mechanism involves two aspects, that plastic straining induces the formation of vacancies and that H facilitates the process. In 1999, Nagumo et al.^437^ illustrated the existence of deformation induced defects in iron by using TDS of H (tritium). Tensile straining was applied to test specimens at 20 °C and −50 °C to various strains up to 25%; tritium was cathodically charged into the deformed specimens; the amount of tritium in the specimens was then measured with TDS. It was found that under the same charging condition and plastic straining, a larger amount of absorbed tritium was observed at the lower temperature of −50 °C. This finding suggests the existence of other types of defects that trap H, apart from dislocations. These defects are more stable at a lower temperature. In another series of tests, the specimens strained to 25% at −50 °C were aging treated at various temperatures up to 600 °C, and tritium was then cathodically charged, followed by TDS measurement. It was found that H absorption decreased significantly from a relatively low aging temperature of about 200 °C. This temperature is too low to anneal any other types of defects such as dislocations or precipitates; it was therefore reasonable to assume that lattice point defects that trap H were annealed, with the point defects presumably being vacancies. Note that in all experiments reported in that work, H (tritium) was charged into the specimens after pre-straining and thermal treatment. Therefore, this work did not focus on H induced fracture or plasticity; it demonstrated the existence of strain-induced vacancies and their capacity to trap a considerable amount of H, and it also showed that TDS with H as a tracer was a viable approach to measuring defect density. However, whether H influences the formation of these vacancies was not investigated.

Later, Nagumo et al.^438^ studied the influence of H on the formation of strain-induced vacancies. H charging of samples was conducted in three scenarios: unloaded specimen, pre-strained specimen, and *in situ* loaded and charged specimen. The specimens were then subjected to TDS measurement and the amount of H was determined. H concentration in pre-strained specimen was found to be higher than that in the unloaded specimen, and the concentration in the *in situ* charged and loaded specimen was the highest. Under the presumption that vacancies are the major H trapping sites, as revealed in the aforementioned work,^437^ it was concluded that H facilitates the process of strain-induced vacancy formation. In 2004, Nagumo^34^ summarized their previous findings and proposed a new mechanism for H induced failure, i.e., the H increased creation of vacancies on straining, and claimed that “the function of H is ascribed to an increase in the density of vacancies and their agglomeration, rather than H itself, through interactions between vacancies and H.” In 2006, Sakai et al.^439^ used the phrase “H stabilizes strain-induced vacancies” to describe the process; Fuchigami et al.^440^ mentioned “the role of H in enhancing strain-induced creation of vacancies”. In 2008, Takai et al.^266^ used the term “H-enhanced strain-induced vacancy model”, and this is to our knowledge the first time that the term HESIV was proposed.

**Figure 18:**
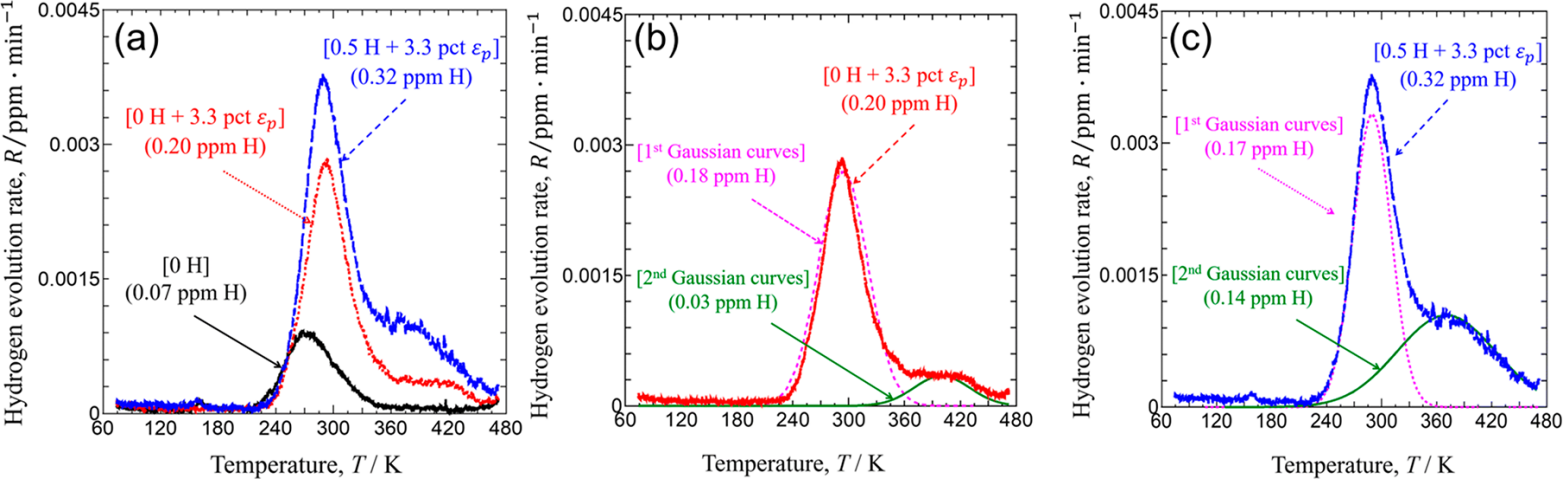
(a) Tracer H desorption spectra and the amount of tracer H corresponding to the amounts of lattice defects of [0 H], [0 H + 3.3 pct εp], and [0.5 H + 3.3 pct εp] specimens. (b, c) Comparison of the H desorption spectra between experiment and fitting using Gaussian function. Reprinted with permission from ref (441). Copyright 2019 Springer Nature.

The HESIV mechanism was further verified and developed with the advancement in H measurement. The conventional TDS technique is not capable of distinguishing the types of lattice defects that trap H in a low temperature range below, e.g., 500 K. Consequently, only one H desorption peak from thick specimens is obtained due to the diffusion-controlled, rather than the de-trapping-controlled process, particularly for *fcc* structures, although H desorbs from various lattice defects in this temperature range, such as dislocations and point defects. Therefore, it is not possible to distinguish vacancy trapping from dislocation trapping with the conventional TDS technique. TDS from a low temperature (L-TDS) can detect H from a temperature as low as 73 K and is therefore suitable for quantitative analysis of vacancy trapping versus dislocation trapping. With the L-TDS technique, Saito et al.^441^ was able to extract a desorption peak of H de-trapping from lattice point defects. Three specimens from tempered martensitic steel were prepared: (1) an H-free specimen without straining, denoted as the [0H] specimen in the original work; (2) an H-free specimen strained to 3.3%, denoted as the [0H + 3.3 pct ε_p_] specimen; (3) an H-pre-charged specimen strained to 3.3%, denoted as the [0.5H + 3.3 pct ε_p_] specimen. The pre-charged specimen was subjected to degassing at room temperature for 48 h, to get depleted of H. All the specimens were then charged with H under the same condition and measured with L-TDS. As illustrated in Figure 18a, the desorbed H was the least in the [0 H] specimen, and the highest in the [0.5H + 3.3 pct ε_p_] specimen, this indicated that straining induced the generation of H traps, which was enhanced by H. The conclusion is consistent with that drawn based on TDS by Nagumo et al.^438^ As shown in Figure 18b and c, H desorption at a peak temperature of approximately 293 K was commonly observed in the strained specimens and the amount was almost equal whether in the presence or absence of H. In contrast, H desorption corresponding to the second peak, about 371 K, was significantly more in the [0.5H + 3.3 pct ε_p_] specimen (0.14 ppm) than in the [0 H + 3.3 pct ε_p_] specimen (0.03 ppm). A reasonable assumption is that the first peak is attributed to H de-trapping from dislocations and the second to the de-trapping from point lattice defects that are induced by the straining. And H apparently enhances the formation of strain-induced point defects. It is worth noting that the pre-charged H in this experiment served as an agent to promote the process of strain-induced vacancy generation, while H charged after the tensile tests and before the L-TDS measurement functioned as a tracer to detect the amount of trapping at various sites. Further, positron annihilation spectroscopy (PAS) was applied in this work, showing elongated positron lifetime in specimens with straining and H, adding evidence that the strain-induced point defects were of vacancy-type, i.e., mono-vacancies and vacancy clusters.

So far, the timeline of experimental investigation on the HESIV mechanism has been summarized by elaborating three important works along the timeline. Except for these works, enormous efforts have been made on this topic, which provided important theoretical support and evidence, and helped advance the HESIV mechanism. A summary of theoretical insights in this respect revealed by atomistic calculations is presented in Section 2.3.5.

##### 2.3.2.4 HELP Mechanism

The HELP mechanism assumes that absorbed H enhances the mobility and generation of dislocations, thereby leading to increased amount of localized plasticity. The mechanism has been under consistent development, endowing it with an increasingly wide scope encompassing a range of scenarios where local plasticity is intensified in the presence of H. The fundamental assumption of the HELP mechanism is that H enhances the mobility of dislocations. The enhanced mobility can be attributed to several causes. In the early version of the HELP mechanism, H was postulated to exert a so-called elastic shielding effect, that is, H atoms accumulating in a region exert an elastic stress field which counteracts that exerted by microstructural defects such as dislocations themselves and precipitates, the result is to facilitate the motion of dislocations in the region since the stress field that arrested the dislocations is weakened.^41^

A work by Yu et al.^442^ with dislocation dynamics simulation demonstrated that H elastic shielding reduces the strength of a Lomer-Cottrell dislocation junction in a *fcc* material, which tends to destruct the junction and restore it to a pair of mobile dislocations. This provides support for the elastic shielding concept. Meanwhile, it should be noted that the concept is built upon linear elasticity, so it fails to account for the influence of H atoms residing in the dislocation core where the stress field is highly nonlinear. In order for elastic shielding to make a noticeable contribution, an extremely high bulk H concentration is required, which is typically in the order of 10^4^–10^5^ appm.^44^ The nonlinear part of contribution due to H in the dislocation core can be considered by implementing a solute (H) interaction term which acts upon the dislocation core energy and hence influences the line tension of a dislocation.^443,444^ It was shown that solute interaction in the core region dominates over H elastic shielding effect, when it comes to H promoted activation of an FR source.^444^ This enables the HELP mechanism to operate at a reasonably low concentration of H in the bulk. Solute interaction in the core region is a short-range effect, so its influence on the interplay between a dislocation and another type of defect such as a precipitate may not be as pronounced as H elastic shielding. This needs further investigation.

**Figure 19:**
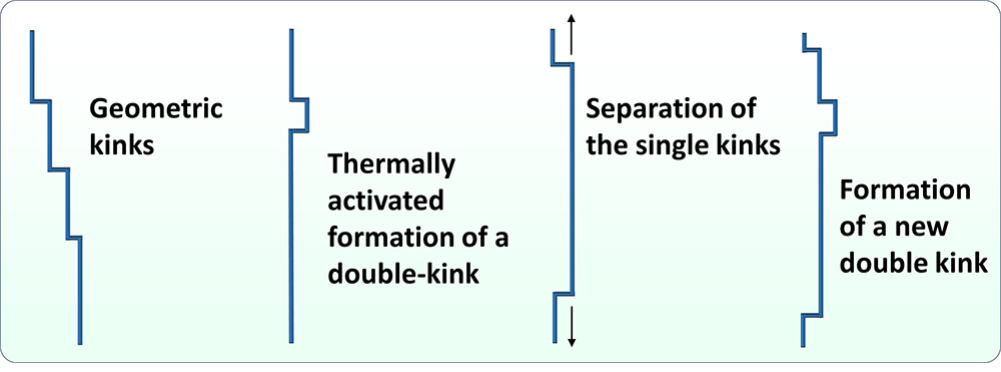
Illustration of the motion of a screw dislocation. A pair of kinks along a screw dislocation line are firstly formed by thermal activation, a jog forms at the same time which, in this example, is to the right of the screw dislocation line; the kinks then move along the dislocation line in opposite directions; the result of this process is that the screw dislocation “moves” in the same direction of the jog, i.e., to the right in this example. Note that a large number of kinks pairs will form along the dislocation line upon thermal activation, and the movement of a screw dislocation is a result of the collective behavior of the kink pairs. Reproduced with permission based on web course material published by Föll,^446^ Kiel University.

H enhanced dislocation mobility is supported by a number of atomistic calculations in the case of a screw dislocation. In *bcc* material, e.g., iron, a screw dislocation moves through the formation and migration of kink pairs. As illustrated in the Figure 19 below, a screw dislocation can be modelled as a piece-wise straight line stretched in the vertical direction. While the dislocation has, on average, a screw orientation, it consists of edge dislocation segments which forms links along the dislocation line. As the edge segments of a single move in opposite directions, the screw dislocation advances in the horizontal direction. Apparently, the mobility of the screw dislocation is higher with a higher frequency of kink pair generation and with a higher mobility of the kinks, and the mobility of a kink is essentially the mobility of a short edge segment. Itakura et al.^346^ and Katzarov et al.^445^ demonstrated that the effect of H on the mobility of a screw dislocation has two folds. It facilitates the generation of kink pairs but hinders the motion of the kinks, depending on a combination of temperature, H concentration, and load. Both studies predicted enhanced mobility of a screw dislocation at room temperature and at a small concentration of H which is often seen in *bcc* material.

In their atomistic calculations, Katzarov et al.^445^ calibrated a relation of H enhanced dislocation mobility which can be used as an input to larger scale models. Yu et al.^44^ implemented the relation in discrete dislocation dynamics (DDD) simulation, and demonstrated that dislocation core-bound H plays a dominant role in H enhanced dislocation mobility, in the *bcc* case.

While mainly focusing on H enhanced dislocation mobility, another aspect of the HELP mechanism is H enhanced slip planarity, which is observed experimentally in a number of *fcc* materials, including aluminium,^447^ nickel,^448^ nickel alloy,^449^ and duplex stainless steel.^297^ The phenomenon is usually attributed to H-suppressed shrinkage of extended dislocations and cross-slip of screw dislocations. In materials with high stacking fault energy (SFE), the H effect on cross-slip of screw dislocations plays an important role in the slip planarity.^450^ Cross-slip is an important mode of motion for a screw dislocation, where the dislocation moves out of its original habit plane and continues to glide on a new one. This facilitates the formation of complex dislocation entanglement and is also an important step in the formation of dislocation loops in particle-strengthened materials.^451^ It is a general consensus that H reduces the tendency of cross-slip, as evidenced by a number of atomistic calculations in aluminum^452^ and nickel.^453−456^ Interestingly, similar H effect on cross-slip has also been reported in pure iron.^457,458^ According to Martin et al.,^21^ a viable explanation for the suppressed cross-slip is that the binding energy of H to an edge partial dislocation is higher than that to a perfect screw dislocation. Therefore, cross-slip, which elongates screw dislocations, requires extra energy consumption to “pump” H atoms out of the energy wells of edge dislocations to allow them to reorient to a screw-type. It is difficult to judge if the reduced cross-slip increases or decreases dislocation mobility. On the one hand, the degree of freedom of screw dislocations seems to be limited; on the other hand, reduced cross-slip should decrease the amount of dislocation entanglement and therefore decrease the number of obstacles to dislocation motion. H enhanced slip planarity should perhaps be viewed as another aspect of the HELP mechanism, which promotes the transformation of homogeneous macroscopic plastic deformation into intense shear but is not directly related to H enhanced dislocation mobility.

Unlike the mechanisms introduced earlier, the HELP mechanism does not directly address fracture that is an indispensable and critical stage of HE. Assuming the HELP mechanism is manifested as a decrease in the flow stress of a continuum body, Sofronis et al.^224^ showed that the inhomogeneous stress-driven redistribution of H and the consequent local softening induces a plastic instability, which signifies a loss of loading bearing capacity, i.e., material failure. However, such a formulation indicates a substantial softening in the global loading curve, which conflicts with many experimental observations as discussed in Section 2.4.1. Neither does the formulation reflect the interactions among dislocations such as dislocation entanglement.

Perhaps the closest link established so far between H enhanced plasticity itself and fracture is the H promoted formation of dislocation substructures, such as dislocation cells, observed in a number of experiments. The cell walls, sometimes referred to as sub-GBs, are potential fracture initiation sites. Gong et al.^459^ observed that cracks were initiated at dislocation substructures that formed as a consequence of H–dislocation interactions in a nanoprecipitation-strengthened steel. They further attributed the actual crack initiation to the dislocation substructure and its associated strain partitioning, rather than debonding, void formation, or crack tip dislocation emission. While the sub-GBs are strictly speaking still precursors to fracture, as they do not directly represent material separation, this work marks an important step towards bridging H enhanced plasticity and fracture, as it showed a possibility to rationalize fracture under the sole theory of H enhanced plasticity, without invoking the HEDE, HESIV, or AIDE mechanism. Based on these findings, it may be possible to simulate H induced fracture with a single HELP mechanism by implementing a failure criterion based on critical dislocation density or misorientation in the cell walls. To achieve this, field methods allowing to map the dislocation density distributions over a sample appear to be mandatory, while defining an average dislocation density over a representative volume element is hardly sufficient.^460^

##### 2.3.2.5 Defactant Concept

The Defactant concept proposed by Kirchheim^35,45,461^ is formulated based on a thermodynamics framework, claiming that solute atoms, in this case H, trapped in defects lower the total energy of the system. In other words, the formation energy of these defects is lowered in the presence of H atoms. By taking different types of defects into the framework, the H promoted formation of a number of defects can be explained, such as dislocations, vacancies, and even microcracks.

Similar to the HESIV mechanism, the Defactant concept also supports H enhanced generation of vacancies. With regard to dislocation activity, the Defactant concept predicts H enhanced plasticity, and it provides a new insight that is not within the scope of the HELP mechanism. In the framework of the Defactant concept, the influence of H atoms residing in dislocation core region is naturally considered. According to the theory, the binding of H to the core reduces dislocation core energy; therefore, the dislocation core energy can be conveniently expressed as a decreasing function of local H concentration. Since dislocation line tension is proportional to the magnitude of dislocation core energy, it follows that H decreases dislocation line tension. Now recall the activation of a dislocation source as discussed earlier. With H decreasing the line tension, the bow-out of a dislocation source, e.g., an FR source, should be eased, and hence the activation of the source, and hence, the multiplication of dislocations is promoted by H. It is noted that the HELP mechanism cannot explain this aspect of dislocation activity. Assuming that H enhances the mobility of dislocations, the HELP mechanism only partially explains the enhanced multiplication of dislocations and local plasticity. We find it helpful to define the scopes of the HE mechanisms and clarify their boundaries. A clear boundary between the AIDE mechanism and the HELP mechanism was drawn by Lynch,^22^ but the boundary between the HELP mechanism and the Defactant concept has been obscure so far. Another plasticity related phenomenon that is difficult to rationalize with the HELP mechanism is the nucleation of dislocations as defined earlier. Simply viewing dislocation as a microstructural defect, the Defactant concept anticipates a decreased formation energy of dislocations in the presence of H; hence, nucleation of dislocations is eased by H. As a matter of fact, the Defactant theory has been employed to rationalize a number of nanoindentation experiments where dislocation nucleation is facilitated in the presence of H.^462,463^

While being distinct, the Defactant theory and the HELP mechanism are not without overlapping. It can be claimed that the Defactant theory provides a rationale for the H enhanced mobility of a screw dislocation as mentioned earlier. Viewing a pair of kinks as defects along a perfect screw dislocation line, the accumulation of H along the dislocation will reduce the formation energy of the kink-pair and therefore increase the kink-pair forming rate, according to the Defactant theory. However, we suggest to leave the H enhanced dislocation mobility to the scope of the HELP mechanism, in order not to complicate the theoretical discussion of HE. Similarly and as indicated at the beginning of this section, the Defactant theory can cover a large part of the HESIV mechanism, but we suggest not to confuse these two theories, either. There may be a period when the H community are interested in a unified theory of HE, “one theory to explain them all”. But it is gradually realized that such a theory will have to be quite generic and so is difficult to implement practically in HE which is quite material- and case-specific. Therefore, we try to limit the scope of the Defactant theory in our discussion, and relate it specifically to the case of dislocation nucleation and activation of a dislocation source, although it has a potential to become a unified generic theory. In doing so, we leave the subjects of vacancy generation and dislocation mobility to the scopes of the theories that were established earlier, i.e., HESIV and HELP.

According to the Defactant theory, H is expected to facilitate the activation of an FR source, whether the source is of edge or screw type. However, there is an opinion that the situation may be more complex, since the binding energies of H to different segments of a bowing dislocation can be different, which is called H segregation. New segments are created as a dislocation source bows out, which possibly leads to a scenario where H is forced to move from a segment with a higher binding energy to a segment with a lower binding energy (in terms of magnitude). H is reluctant to do so and therefore may obstruct the activation of certain types of dislocation sources. This is an important argument for viewpoint of H suppressed plasticity.

##### 2.3.2.6 H Suppressed Plasticity

The HELP mechanism and the Defactant theory had great success in rationalizing the phenomena of HE. H enhanced plasticity is supported by a great number of experiments conducted at the microscopic scale. With the advancement in experimental and numerical techniques over the past decade, there has been an endeavor to zoom into an elementary dislocation activity and study how exactly it is influenced by H. A typical example is the very recent work by Huang et al.,^46^ which experimentally studied the bow-out of a screw dislocation segment in iron, using *in situ* nanopillar testing method. It was confirmed that H enhances the motion of a screw dislocation in iron, which is a support to the H enhanced plasticity theories. Another powerful tool is atomistic modeling which enables the analysis of a single dislocation segment or an elementary dislocation activity such as the activation of an FR source. It is noted that the atomistic modeling approach is applied in a wide range of HE scenarios beyond the study of a few dislocations, which is elaborated in Section 2.3.5. The discussion in this section is limited mainly to dislocation and plasticity.

Research, especially atomistic study, in the past decade has raised viewpoints inconsistent with the H enhanced plasticity theories. This is not surprising. While the HELP mechanism and the Defactant theory are undoubtedly well established and supported by a large body of evidence, some statements in the theories were deduced from post mortem evidence and some were made in a generic manner. For example, “H enhanced dislocation mobility” did not specify the type of dislocation concerned in the statement. Inconsistent findings are therefore an expected outcome of a research that zooms into details not explicitly addressed by the existing theories. The findings provide a valuable reference to sophisticate existing theories.

Based on MD simulation of Ni–H system, Song and Curtin^464^ proposed that H accumulation around a microcrack tip prevents crack-tip dislocation emission and thus suppresses crack-tip blunting and ductile fracture, which promotes cleavage fracture. This is not consistent with the AIDE mechanism. But interestingly, the two seemingly contradictory opinions both point to a suppressed plasticity zone ahead of the crack tip, thus a sharper and more easily extending crack due to H, which is consistent with the observation of “embrittlement”. In other words, both the AIDE mechanism and the viewpoint of H suppressed dislocation emission support H suppressed localized plasticity in the bulk. Now recall that the HEDE mechanism is suspected to be more difficult to operate than the AIDE mechanism. The H suppressed dislocation emission anticipates a decrease in plastic dissipation and an increase in the hydrostatic stress field thus in the concentration of H, which probably helps establish the condition for HEDE to take place. The same authors^47^ later demonstrated that H suppressed dislocation emission applies also to *bcc* material, which was demonstrated by MD simulation of Fe–H system. The mechanism was further connected to material states and loading conditions through a kinetic model for H delivery to the crack-tip region. Based on the simulation, prediction was made of embrittlement thresholds in steels which were found in good agreement with experiments. It should be mentioned that H enhanced dislocation emission was observed in the MD simulation conducted by Taketomi et al.^465^ The discrepancy could be attributed to the difference in the empirical potential or the different method for loading the system.^21^

There exists a contradictory opinion to the Defactant theory regarding the activation of an FR source. In the MD simulation conducted by Tehranchi and Curtin,^23^ the redistribution of H atoms along an elongating dislocation line during the activation of an edge-type FR source was taken into account, and H was observed to obstruct the activation of the source. This was attributed to the fact that H prefers to bind to an edge segment rather than a screw segment of a dislocation line.^23^ When the edge-type source is activated, screw segments are created and H is forced from an edge site to a screw binding site, which is energetically unfavorable. Following this rationale, the activation of a screw-type dislocation source should be facilitated by H because edge segments will be generated during the bow-out, which is energetically favorable for H segregation. Regarding a screw-type dislocation source, however, the MD approach is less employed because the screw dislocation–H interactions predicted with a widely cited interatomic potential exhibited some unphysical local effects.^42^

The atomistically revealed viewpoints of H suppressed plasticity provide a valuable reference for the improvement and evolution of existing HE theories. Meanwhile, it should be noted that some of these viewpoints are still controversial because of the discrepancy in the atomistic simulations. As pointed out by Tehranchi and Curtin^23^ and Martin et al.,^21^ it is crucial to select a proper empirical potential and loading algorithm in an MD simulation to correctly capture H–dislocation interaction at the atomic scale. Ideally, before an MD setup is applied to study a specific H related problem, the setup should be verified against some benchmark cases to ensure that no obvious artefact is produced with the setup. An option is to compare the energetics of H in the model system calculated with the selected potential to that obtained with ab initio calculations.^466^ The practice adopted by Song and Curtin^42^ is a good example in this regard, where they did not continue with MD simulation of a screw dislocation because unreasonable local event was observed in that case with the selected interatomic potential.

##### 2.3.2.7 Synergistic Action of HE Mechanisms

It is a general consensus that HE is a multifaceted phenomenon that cannot be fully explained by a single mechanism, and more than one HE mechanism can coexist, contributing to the final fracture. This may be referred to as the synergistic action of HE mechanisms, literarily meaning that more than one HE mechanisms cooperate and the combined effect is greater than their individual impacts. To our knowledge, the term “synergistic action of HE mechanisms” was first introduced by Novak et al.^467^ to describe the cooperation between HELP and HEDE mechanisms. This concept was further elaborated by Djukic et al.^40^ Broadening the scope, this term may also encompass the interplay of several mechanisms, such as the HESIV mechanism and Defactant concept.

In Section 2.3.2.4, the feasibility of rationalizing fracture solely based on the HELP mechanism is discussed, attributing to extreme strain partitioning associated with the formation of dislocation substructures. Quite frequently, fracture initiates at other types of impurities in a material, such as the lath boundary of a high strength steel^37^ and the GB in nickel.^36^ It is probable that H induced fracture is a consequence of the synergistic action of the HELP mechanism and other mechanisms such as the HEDE mechanism, the AIDE mechanism or the HESIV mechanism. In this scenario, the HELP mechanism is a crucial precursor that “prepares for” fracture by establishing locally critical conditions to trigger one of the mechanisms. For example, through the operation of the HELP mechanism, more dislocations are generated in a material, and these dislocations tend to accumulate at a material interface like a lath boundary or GB, this at the same time leads to the accumulation of H at the interface, because a dislocation may transport H with it. The accumulation of dislocations and H causes an increase of local stress and a decrease of fracture resistance at the interface, which simultaneously triggers the HEDE mechanism. This example actually reiterates the key concept of the so-called H-enhanced and plasticity-mediated decohesion mechanism.^37^ This serves as a bridge between the HELP mechanism and the critical event of fracture and most importantly, it reflects the reality of HE, as evidenced by a series of experiments.^36−38,468^ A similar pattern may be adopted to synergize the HELP mechanism and the HESIV mechanism, as indicated by Martin et al.^21^ and Nagumo and Takai.^20^

It is important to note that the HELP mechanism may not always be a prerequisite for other mechanisms to operate, as pointed out by Djukic et al.^40^ and which aligns with the discussion in Section 2.3.2.6. They proposed that the HEDE and HELP mechanisms may operate simultaneously but more independently. Typically, HELP predominates at lower H concentrations, while HEDE becomes more dominant at higher H concentrations. In this way, the two mechanisms still act synergistically, in the sense that they both add to fracture. Meanwhile, they act competitively, as one mechanism may overshadow the other. This concept differs from the H-enhanced plasticity-mediated decohesion mechanism and is termed the “HELP+HEDE” model. For more details of the concept and a schematic illustration of the HELP+HEDE model versus the H-enhanced plasticity-mediated decohesion mechanism, readers are directed to the review article by Djukic et al.^40^

The concept of synergistic action among various HE mechanisms holds significant value, particularly for predictive modeling as detailed in Section 2.3.6. To effectively apply this synergistic action, two key prerequisites must be met. Firstly, a comprehensive understanding and quantification of each individual HE mechanism is essential. Secondly, a precise mapping of these mechanisms to specific H conditions and material characteristics is required. This mapping should accurately define the threshold conditions that mark the transition between different mechanisms. Fulfilling these prerequisites allow for a practical implementation of the synergistic action of multiple mechanisms, thereby capturing the complete HE processes more effectively.

#### 2.3.3 HE Mechanisms Identified by Material Categories

**Figure 20:**
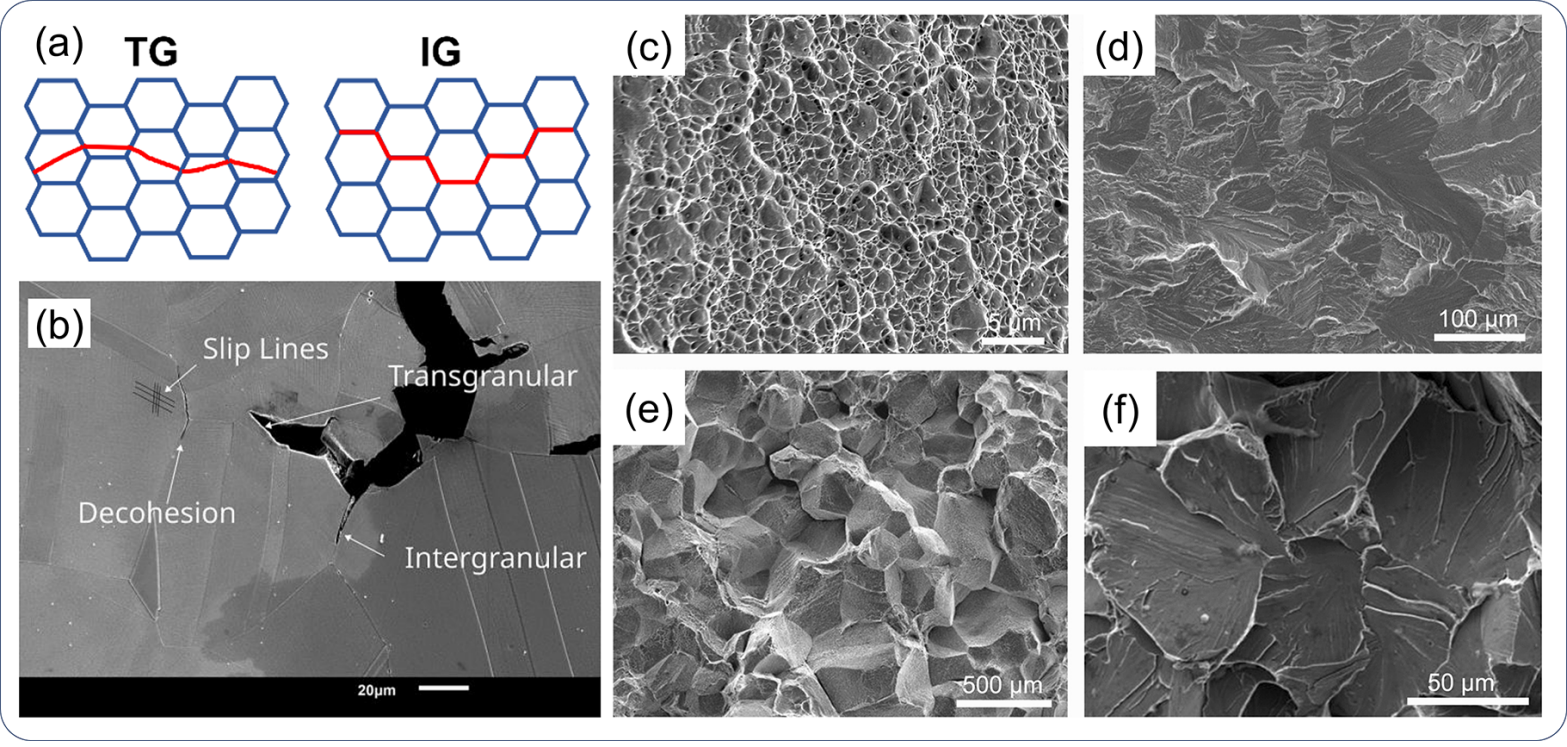
(a) Schematic of H-assisted TG and IG fracture. (b) Surface cracks showing different failure modes in conventional Inconel 718. Reprinted with permission from ref (469). Copyright 2023 MDPI under [CC BY 4.0 DEED] [https://creativecommons.org/licenses/by/4.0/]. (c–f) Fractography of steel samples failed in H-free and H-charged conditions showing (c) MVC-induced dimple, (d) quasi-cleavage fracture, (e) IG fracture, (f) cleavage fracture. Reprinted with permission from refs (24 and 470). Copyright 2023 Springer Nature. Copyright 2019 Elsevier.

Because H is affinitive to and brings irreversible mechanical degradation to practically all types of metals and alloys, it is crucial to identify the appropriate failure mechanisms for each material category, based on the HE mechanisms discussed in Section 2.3.2. Materials sharing similar characteristics typically exhibit analogous failure processes under comparable loading and H charging scenarios. Therefore, rule of thumb is a thorough characterization and understanding of the microstructural elements influencing cracking and plasticity behaviors, specifically crystallographic structure (*fcc*/*bcc*/*hcp*), phase distribution (single/multiple), grain size, GB character (low/high angle), precipitates (distribution/fraction), phase transformation, etc. This approach facilitates the correlation of H-assisted fracture morphologies with established failure mechanisms. Different from ductile failure featured by dimples in H-free samples (Figure 20c), H-assisted brittle fracture showcases either TG or IG fracture mode or a combination of both (Figure 20a,b). Fractography of TG fracture reveals flat brittle features within the grain matrix, presenting as cleavage facets (fracture along cleavage plane), or quasi-cleavage (fracture along non-cleavage plane) features such as tear ridges, river patterns, and striations, see Figure 20d and f. A mix of these brittle features and dimpled fracture surfaces may be observed when H concentration is not sufficiently high. TG cracking initiates from areas with high stress and strain concentrations, including slip band intersections, defects in the matrix and GBs, and it propagates across the grain interior. This involves significant plastic deformation enabled by dislocation activities related to microstructural features near the crack tip, often explained within the framework of HELP, AIDE, and HESIV mechanisms. IG fracture refers to the debonding of GB planes due to the reduction of cohesive energy in the presence of H and appears as flat facets with suppressed plasticity (Figure 20e). Typically, H-assisted IG brittle fracture results from a combined effect of H atoms diffusing along or trapped at GBs and other embrittling elements segregating therein. HEDE mechanism and Defactant theory are often used to explain the failure of GBs. Several examples of TG and IG fracture are shown in Figure 20.

Given the complexity and diversity of mechanisms reviewed in Section 2.3.1, definitive conclusions regarding the predominance of one mechanism over another remain elusive. Often, interpreting embrittlement behavior necessitates invoking a combination of mechanisms. This section aims to encapsulate HE mechanisms pertinent to critical material categories, including *bcc* iron and steels, *fcc* nickel alloys, steels exhibiting transformation-induced plasticity (TRIP), twinning-induced plasticity (TWIP), and multi-phase steels. HE mechanisms in aluminum alloys, high entropy alloys (HEAs), and additively manufactured materials are also reviewed. Efforts are made to cross-correlate H-assisted crack initiation and propagation paths with microstructural characteristics, thereby linking fracture observations to specific HE mechanisms. Criteria essential for drawing such conclusions encompass: (a) the diffusivity and solubility of H within the microstructure, (b) identification of microstructural features highly susceptible to H-assisted cracking, and (c) the manner in which plastic deformation unfolds under certain loading conditions in the presence of H.

##### 2.3.3.1 *bcc* Iron and Steels

**Figure 21:**
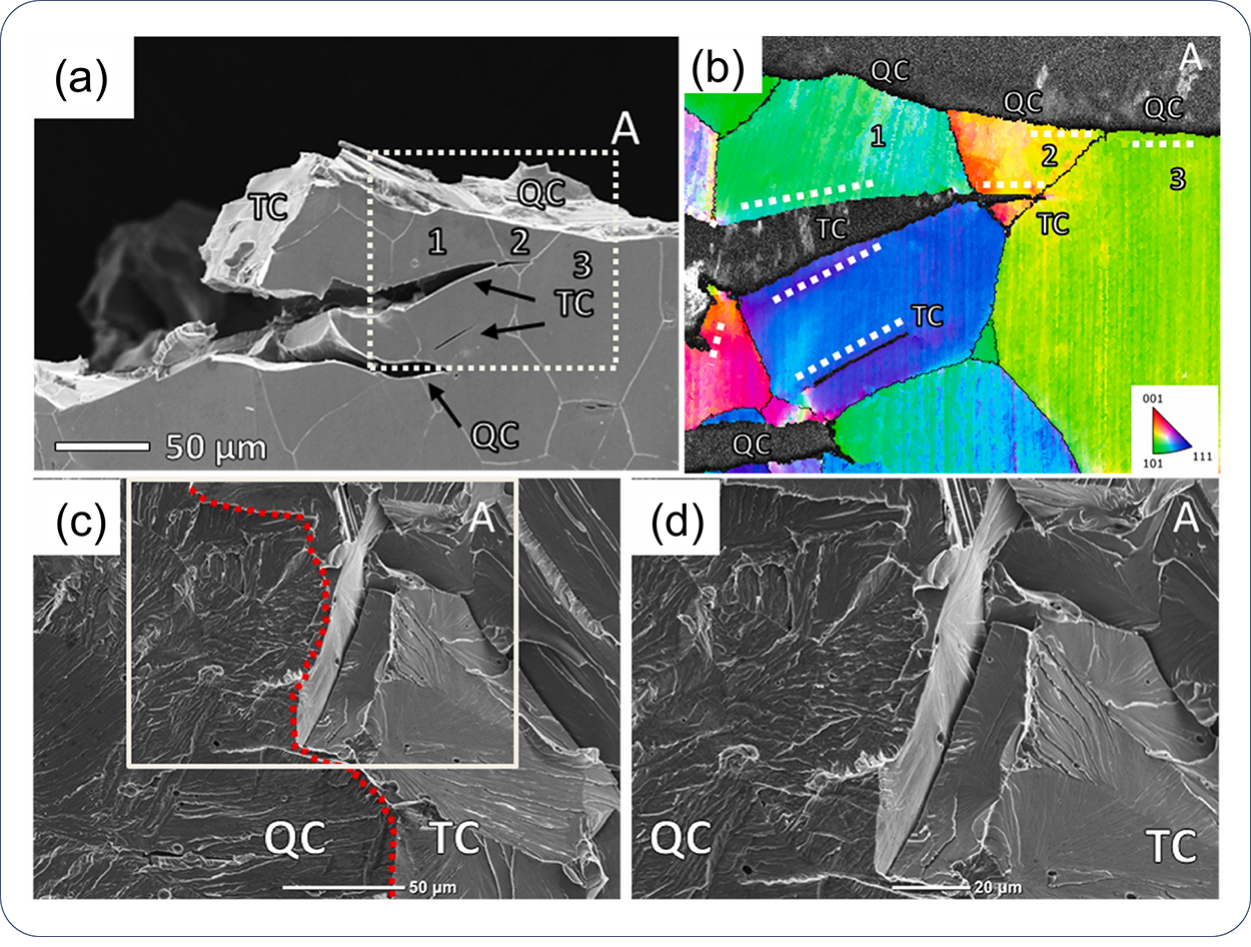
(a) SEM image showing true cleavage (TC) and quasi-cleavage (QC) cracks on the Fe-2.5%Si alloy specimen surface. (b) Corresponding EBSD inverse pole figure map, white dotted lines indicate {100} plane traces. (c, d) Fractographies of both TC and QC fracture features. Adapted with permission from ref (433). Copyright 2021 Elsevier.

α-Fe (ferrite) and *bcc* steels (e.g., carbon steel, martensitic steel, pearlitic steel, etc.) are representative metallic materials for *bcc* metals. As reviewed in Section 2.2, *ab initio* calculations have shown that H atoms primarily occupy tetrahedral sites in *bcc* iron; H diffusivity in *bcc* lattice is high, while the solubility is low. However, the measured H content is normally higher than that predicted by the theoretical solubility due to the existence of various traps in the microstructure, such as dislocations, GBs, precipitates, and possible vacancies. Without external loading, cathodic H charging can cause extensive planar defects in either grain matrix or GBs due to lattice distortion by H.^471^ Under applied stress, HE in the α-Fe manifests as IG and TG fracture. Figure 21 shows H-assisted TG fracture in *bcc* Fe-2.5%Si alloy along the {100} cleavage plane for true cleavage crack, and quasi-cleavage deviates by approximately 30° from the {100} plane.^433^ In most cases, true cleavage is rare, quasi-cleavage is commonly observed. In *bcc* steels with partly or fully ferritic phase, quasi-cleavage fracture was also observed under monotonic or cyclic loading.^472^ In comparison, at a low stress intensity range, H-induced IG fracture was observed in a fatigue test.^473^ More about HE under cyclic loading is presented in Section 2.4.3. When a sufficient amount of H is present in iron, the evolution of dislocation microstructure can be accelerated under the framework of the HELP mechanism; in this way, dislocation cell structure can be formed beneath the IG facets.^38^ Generally, H effect on the failure mechanism of *bcc* iron lies in the following aspects: (1) H atoms lower the cleavage stress of the system, through the HEDE mechanism and Defactant theory; (b) H atoms lower the kink pair formation energy of screw dislocations upon introducing H, thus increasing screw dislocation mobility, which is linked to the HELP mechanism; (c) H atoms segregate and change dislocation core structure, and reduce the strength and extent of elastic interactions among dislocations, i.e., the HELP mechanism and Defactant theory; (d) H accelerates dislocation cell evolution and the formation of microvoids at GBs, invoking the H-enhanced and plasticity-mediated decohesion mechanism and HESIV.

In *bcc* steels such as martensitic and pearlitic steels, where various phase boundaries and phase morphologies exist, HE mechanisms are more complex compared to those in pure iron. Various types of fracture modes have been reported for *bcc* steels. For instance, HE in a lath martensitic steel includes cleavage along {100} martensite (α′) planes, quasi-cleavage and decohesion at lath and block martensite boundaries.^37,474^ The appearance of flat surface feature on the fractography was induced by the debonding along the prior austenite GB.^37^ Upon further characterization, intense slip bands were observed beneath the fracture surface. The mixed-mode fracture can be attributed to the H-enhanced and plasticity-mediated decohesion at the boundaries. In a carbon steel under fatigue loading in a gaseous H environment, Ogawa^472^ reported the formation of planar and wide striations, which was explained by the localized slip deformation near the hydrogenated crack tip based on the HELP mechanism.

Non-metallic inclusions in steels can act as the initiation sites for secondary surface cracks. *In situ* electrochemical charging and tensile test of a ferrite-bainite X65 steel have shown that the majority of surface cracks initiated at non-metallic MnS-Al_2_O_3_ inclusions due to the synergistic action of elevated stress and trapped H at the interfaces.^202^ TG cracks propagated along {110} slip planes, which indicate that higher subsurface H concentration promotes highly localized slip bands on the {110} planes and facilitates the decohesion as per the HELP and HEDE mechanisms. Similarly, Al, Si, Ti-rich oxides were demonstrated as preferred crack initiation sites in X65, X80, and X100 steels.^475−477^

##### 2.3.3.2 Nickel Alloys

**Figure 22:**
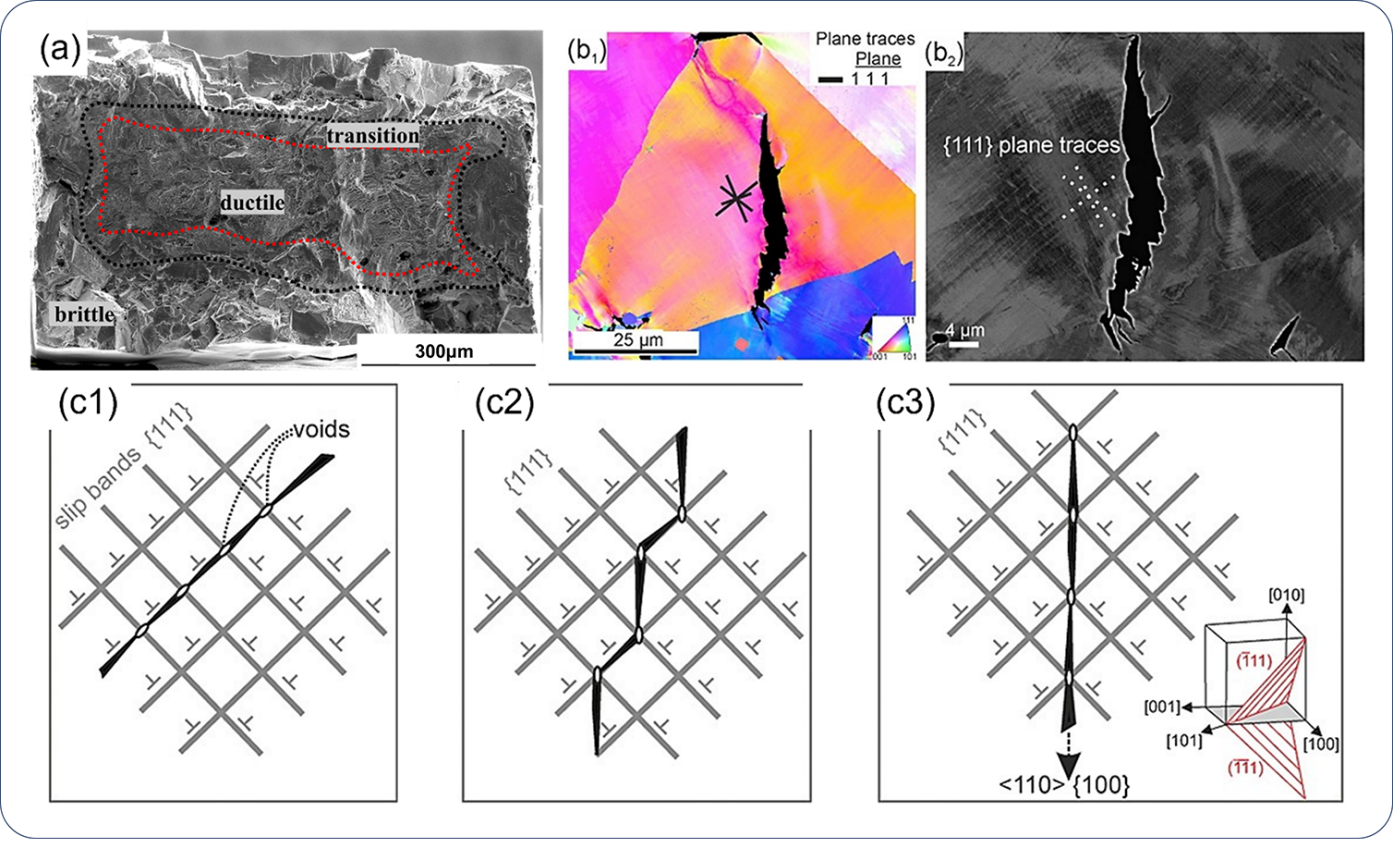
(a) SEM image showing a typical fractography of nickel alloy failed in three H-containing fracture zones. Reprinted with permission from ref (200). Copyright 2019 Elsevier. (b1, b2) Inverse pole figure mapping and backscattered diffraction image of H-induced TG cracks along alternating {111} slip planes. Schematics showing localized deformation resulting in cracks along (c1) slip band, (c2) alternating slip planes, (c3) {100} plane following <110> direction. Reprinted with permission from ref (480). Copyright 2017 Elsevier.

For *fcc* nickel and its alloys, H diffusivity is orders of magnitude lower compared to *bcc* iron. Because of the low diffusivity, H penetration depth for nickel alloys is normally several to tens of micrometers. The fracture surface of a tensile specimen typically exhibits three different zones, a brittle zone in the exterior, a ductile zone in the interior, and a transition zone in between. With respect to fracture mode in H environment, both TG and IG fracture have been reported in the literature (Figure 22a).

TG fracture was reported in a number of precipitation-hardened nickel alloys. The appearance of quasi-cleavage failure on fracture surfaces indicated that such failure occurs along dislocation slip lines, attributed to H-enhanced plasticity causing shear localization along slip planes. TG cracks can appear as straight crack along single {111} plane,^184,478,479^ as zig-zag crack along alternating {111} planes (Figure 22b1,b2) or as cracks with growth directions on {100} planes near <110> crystallographic directions (Figure 22c1–c3). The localized plastic deformation caused by the presence of H can be attributed to the following processes:^14,200,480^ (a) H stabilizes the edge component of dislocations and promotes planar slip; (b) H reduces SFE, suppresses cross-slip, and thereby facilitates slip planarity; (c) stress concentration is markedly increased at slip band intersections, promoting microvoid nucleation; (d) crack deviation from {111} slip planes happens if unequal amount of dislocation slip arises on each cracking side.

**Figure 23:**
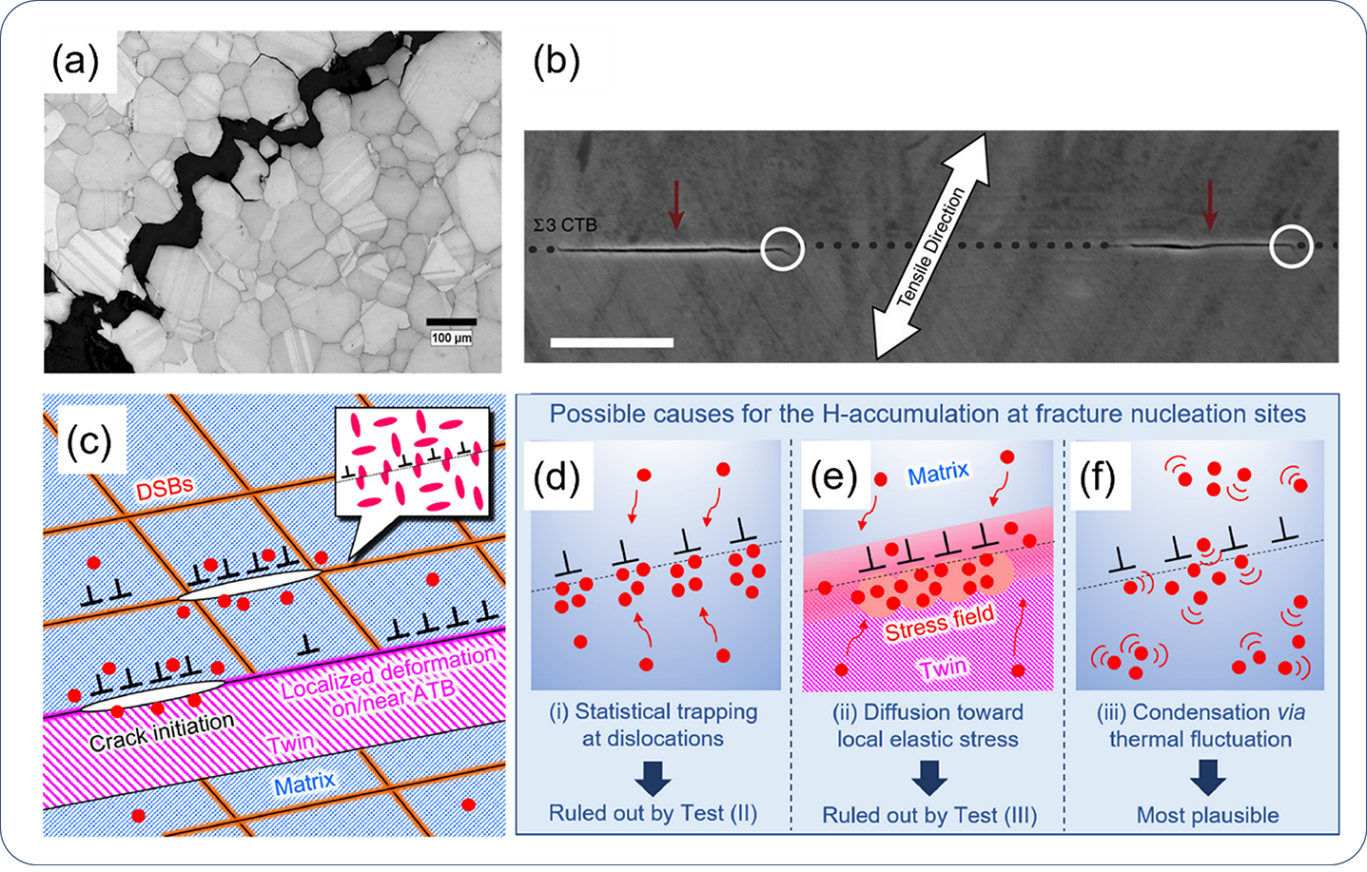
(a) Overview of IG fracture of a nickel alloy failed in H environment. Reprinted with permission from ref (486). Copyright 2017 Springer Nature under [CC BY 4.0 DEED] [https://creativecommons.org/licenses/by/4.0/]. (b) SEM image showing the duality of coherent twin boundary (dotted line). Two cracks initiate along coherent twin boundary were observed, but both terminate in short segments. Reprinted with permission from ref (483). Copyright 2015 Springer Nature. (c–f) Schematic illustration of possible mechanisms for crack initiation along annealing twin boundary and slip plane due to the accumulation of H and dislocation causing lattice decohesion. Reprinted with permission from ref (485). Copyright 2022 Elsevier.

Considering that H diffusion through Ni lattice is slow, GBs as potential short-circuit diffusion pathways become critical when assessing HE of *fcc* metals.^207^ The significance of GBs is underscored in nickel alloys, where IG fracture is prevalently documented (Figure 23a). Specifically, IG cracking is associated with the purity of GB and plastic incompatibility with respect to crystallographic orientation. Atomistic simulation has revealed the connection between the mechanochemistry of H adsorption and GB decohesion for a variety of symmetric tilt grains in Ni, and described the GB strength as a decreasing function of H content occupying polyhedral interstitial sites.^481^ Segregation and accumulation of H are in turn dependent on the GB characteristics, with coherent and low-energy GBs exhibiting reduced affinity for H segregation compared to random GBs.^482^ Localization of plasticity under H occlusion at random GBs and triple junctions is believed to increase stress concentration and facilitate crack initiation. Seita et al.^483^ found in H-charged Inconel 725 that coherent twin boundaries are susceptible to crack initiation, which may have to do with the enhanced dislocation activity at these boundaries, for instance, H-enhanced dislocation-mediated GB sliding, H-enhanced and plasticity-mediated decohesion, and slip transmission (Figure 23b). Further study highlighted the important role of boundaries with low-index planes in deflecting cracks, which strengthens the material and improves HE resistance.^484^ However, a recent work^485^ on nickel superalloy 718 claimed that dynamic H–dislocation interaction may not be an important factor for the onset of H-assisted crack nucleation at annealing twin boundaries; instead, the crack nucleation is governed by the H atoms initially segregated in localized dislocation slip bands along the GBs prior to the onset of mechanical loading. A high H concentration can be achieved because of the high dislocation density in these regions; cracks are then nucleated there upon loading, due to the reduced lattice coherency induced by the presence of dislocations and H. This process is schematically illustrated in Figure 23c–f. Djukic et al.^40^ pointed out that the HEDE mechanism can dominate over the HELP mechanism upon local reaching of a high H concentration close to or above a critical value, while these mechanisms often act synergistically. This helps explain the above process.

**Figure 24:**
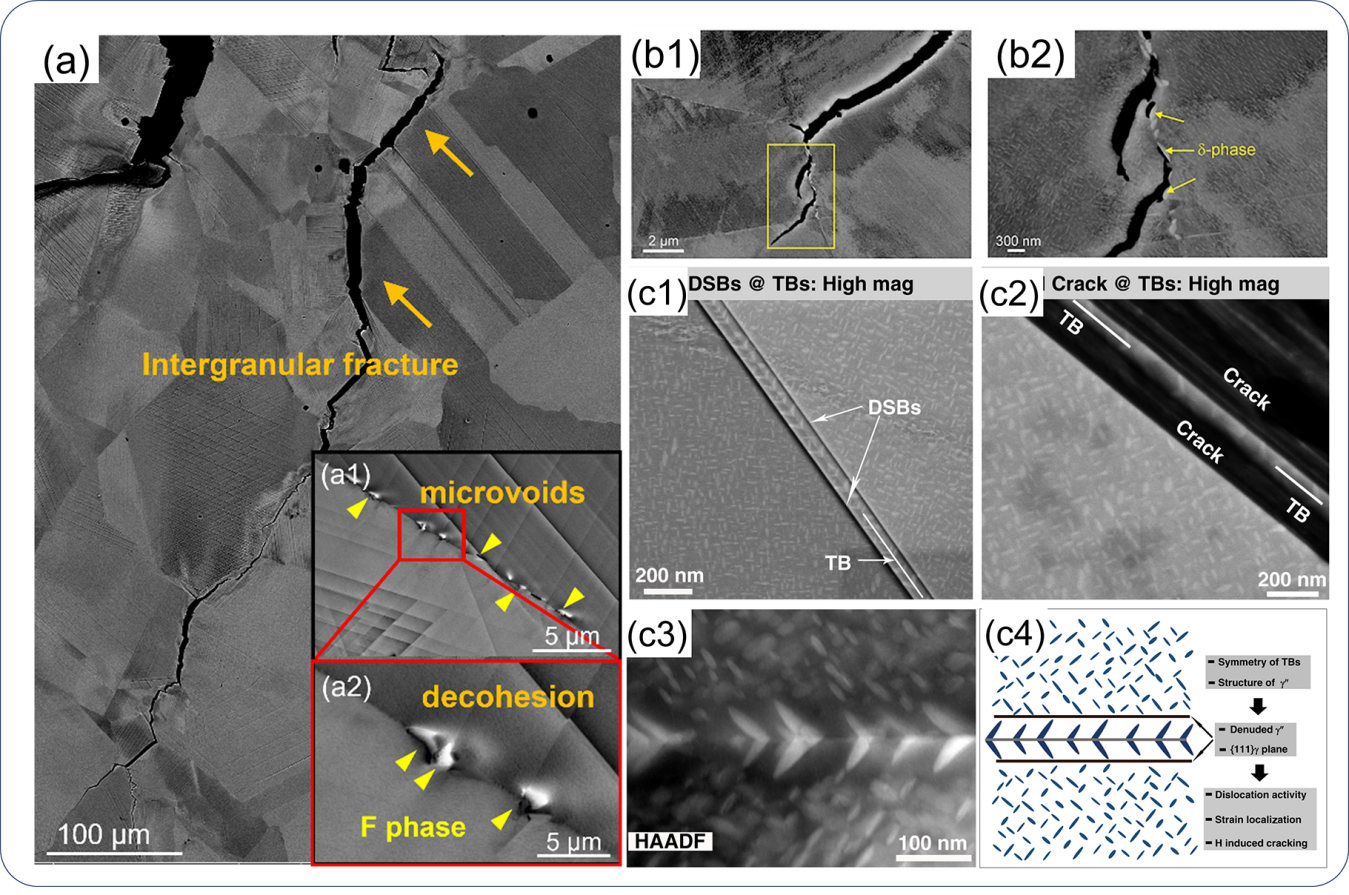
(a–a2) SEM images showing H-assisted IG cracking initiated from F phase/Ni matrix interface in a precipitation-hardened Alloy 725. Reprinted with permission from ref (491). Copyright 2023 Elsevier under [CC BY 4.0 DEED] [https://creativecommons.org/licenses/by/4.0/]. (b1, b2) H-assisted cracking along GB δ phase in Alloy 718. Reprinted with permission from ref (480). Copyright 2017 Elsevier. (c1–c4) H-induced cracks associated with twin boundary due to strain localization around abnormal γ′′ precipitates in Alloy 945X. Reprinted with permission from ref (490). Copyright 2020 Springer Nature under [CC BY 4.0 DEED] [https://creativecommons.org/licenses/by/4.0/].

Besides the strong impact of GB characteristics, the presence of GB precipitates (carbides, δ, F phase) and embrittling elements (S, P) have been demonstrated to promote H-assisted IG failure under the framework of the HELP and HEDE mechanisms and the Defactant theory.^13,480,487−489^Figure 24 shows the detrimental effect of GB precipitation F phase (Figure 24a–a2) and δ phase (Figure 24b1,b2) facilitating H-induced microvoid initiation and crack propagation.^480^ H lowers the cohesive energy of the precipitate and matrix interface. Upon loading, dislocations pile against GBs and cause high strain localization. The deformation process also establishes high H concentration at GBs, thereby facilitating GB and interfacial decohesion. In addition, it has been shown that abnormal γ′′ precipitation at twin boundaries in a Ni-based superalloy 945X facilitated dislocation activities, induced strain localization and finally led to crack initiation at twin boundaries during mechanical loading (Figure 24c1–c4).^490^ In addition, the transition between TG and IG fracture modes is dependent on H concentration. It was reported in nickel that the HELP mechanism plays a critical role in premature IG fracture with a bulk H concentration of 400 appm, whereas the HEDE becomes the dominant embrittlement mechanism when H content increases to 1200 appm.^481^ This aligns with the summary made by Djukic et al.^40^ about the possible synergy and competition between the HELP and HEDE mechanisms.

##### 2.3.3.3 TRIP Steels

TRIP-assisted steels are advanced high strength steels, which are widely used to make components in the automotive and construction industries because of their high strength and light weight.^492^ TRIP steels are designed with a minimum 5% volume fraction of metastable retained austenite. Such steels possess high work hardening rate due to the formation of hard martensite (α′) during deformation. The effect of H on the mechanical degradation of TRIP and TRIP-aided steels is an important aspect in the engineering application.

**Figure 25:**
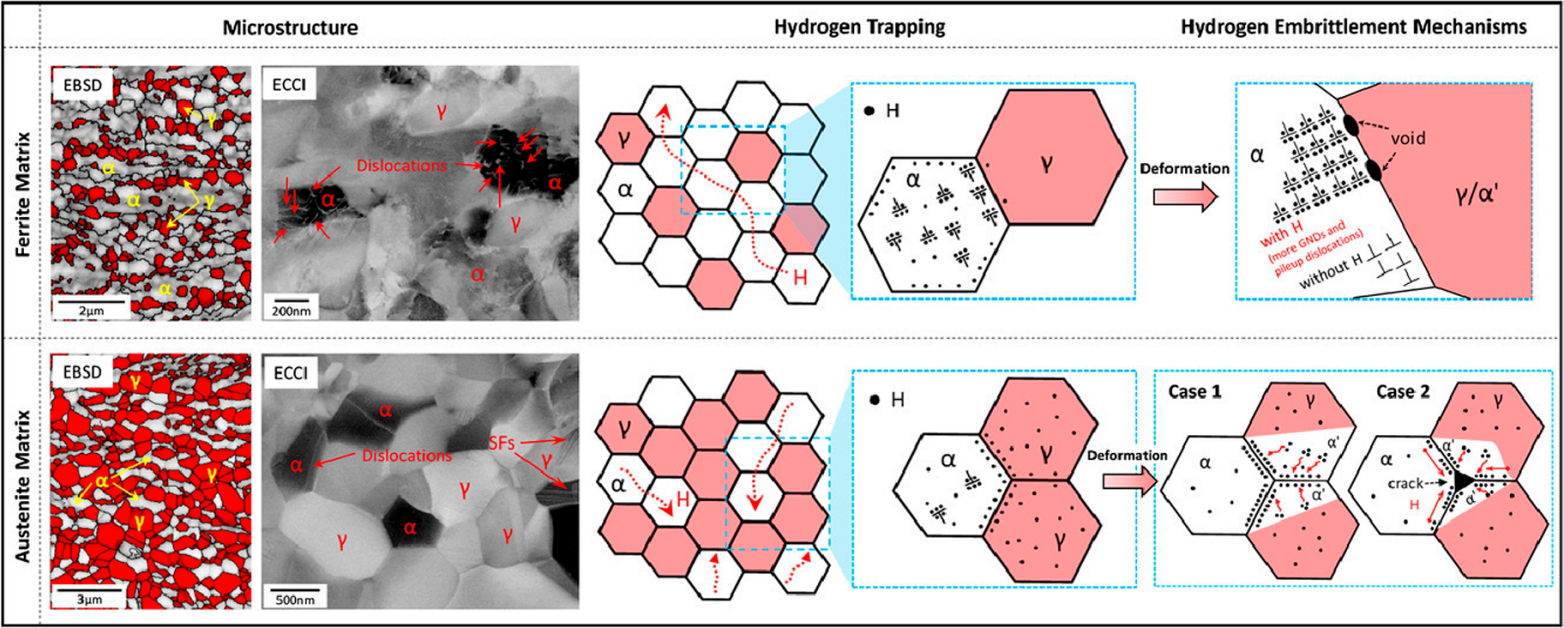
Schematic describing different HE mechanisms in medium Mn steels containing different ferrite and austenite fraction. Reprinted with permission from ref (495). Copyright 2020 Elsevier. For ferrite matrix steel, the HELP mechanism is responsible for the strain incompatibility between ferrite and adjacent γ/α′-martensite interfaces. For austenite matrix steel, the HEDE mechanism and H accumulation at phase boundary are responsible for the IG fracture.

In TRIP-780 steel and FeCCrNiBSi TRIP steel, H was reported to trigger cracking in the fresh martensite phases transformed from unstable austenite, and facilitate crack propagation along γ/α′ interfaces or through ferrite (α) grains.^492,493^ In this case, both IG and TG fracture modes were observed. H lowers the SFE of austenite, which promotes the formation of ε martensite in the slip bands and finally transforms it to α′-martensite. The larger the α′-martensite fraction, the more susceptible the material is to HE. IG cracking is attributed to the sudden change in H diffusivity and solubility from γ to α′-martensite, which results in high H concentration and stress localization at the γ/α′ interfaces and facilitates fracture. This process is related to the HEDE mechanism. TG cracking manifests as quasi-cleavage fracture primarily associated with void formation at dislocation cell boundaries as per the HELP and HESIV mechanisms.^459^ TRIP-aided medium Mn steel has recently gained attention because of their excellent combination of strength and ductility. H-assisted cracking typically occurs at α′-martensite-associated interfaces, such as α/α′-martensite phase boundaries.^494^ The initial fraction of ferrite and austenite phases has been demonstrated to be an important factor determining the underlying HE mechanism that operates during deformation. The fracture mechanism in a medium Mn steel with a higher ferrite fraction (∼74 vol. %) is attributed to the HELP mechanism in ferrite and the enhanced strain incompatibility between ferrite and adjacent austenite or transformation-induced α′-martensite phases,^495^ while the HEDE mechanism becomes dominant in the decohesion fracture along phase boundaries and GBs in medium Mn steel with a large fraction of austenite (∼59 vol. %).^495^ A schematic illustration of these two scenarios is shown in Figure 25. Non-metallic inclusions have also been demonstrated to be preferential initiation sites for H-induced cracks.^496^ Because H solubility is high in austenite, the overall H content depends on the retained austenite fraction. Higher retained austenite fraction typically results in higher HE susceptibility.^496^ For TRIP steel with existing ε martensite, TG fracture was reported to initiate preferably in ε-martensite with <0001>||RD microscopic crystallographic orientation.^497^ Kumai et al.^498^ reported that pre-straining can influence the cracking mechanism of a TRIP-aided bainitic ferritic steel. The pre-strain-induced dislocations trapped more H in the grain interior, which induced quasi-cleavage fracture through H enhanced multiplication and pile-up of dislocations.^498^ At a high pre-strain level, local accumulation of dislocations near microstructure boundaries caused highly heterogeneous stress fields and H distribution, thus inducing HE.^498^

##### 2.3.3.4 TWIP Steels

**Figure 26:**
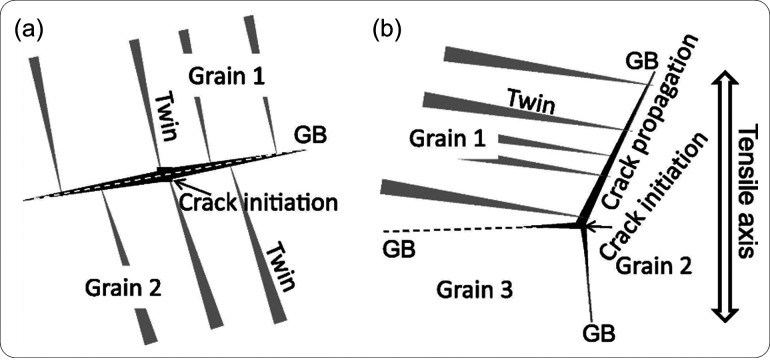
Schematics showing: (a) the crack initiation by intercepting deformation twinning; (b) triple junction cracking at GBs in an H-charged TWIP steel. Reprinted with permission from ref (504). Copyright 2013 Elsevier.

TWIP steels are the second-generation advanced high strength steels composed of *fcc* austenite phase. The TWIP effect imparts an excellent combination of strength and ductility to the steels; however, it also results in significant susceptibility to HE. Under H pre-charging and tensile testing, the primary fracture mode of high Mn TWIP steels (i.e., Fe-22Mn-0.6C, Fe-18Mn-0.6C) is IG fracture.^499−502^ This is caused by the H effects on the SFE, deformation twinning, and dislocation activity. H has been shown to decrease the SFE as per the Defactant theory, thereby promoting early nucleation of stacking faults and deformation twins.^499^ H simultaneously decreases the twin thickness but increases the twin density in individual twin bundles.^503^ It was demonstrated that high angle GBs and grains with tensile axis orientations close to <111>//RD and <112>//RD were more susceptible to H-induced IG cracking.^499,500^ The interaction between twins and GBs caused strain localization and promoted microvoid formation.^500^ Koyama highlighted that H-assisted cracking can also initiate at deformation twins.^504^ A schematic describing twin evolution associated with IG cracking is shown in Figure 26a and b.^504^

By elevating the SFE, the detrimental effect of twinning-induced stress concentration can be remarkably mitigated.^505^ It was demonstrated that the addition of Al can effectively suppress H-induced IG fracture, by increasing the SFE and postponing the nucleation of twins, thus reducing the stress level at GBs.^501,506,507^ The effect of the addition of Mn has been under debate: Zhou et al.^508^ found that an increase of Mn content reduced the H diffusion coefficient and H solubility in TWIP steel, which tends to improve the HE resistance of TWIP steel. On the contrary, Claeys et al.^502^ claimed that Mn had a detrimental effect since it enhanced H diffusivity in TWIP steel. Koyama et al.^509^ demonstrated that Mn decreased GB cohesive energy and promoted IG fracture in an H environment; therefore, high Mn content should be avoided. In addition, grain refinement is another mitigation strategy to improve H resistance of TWIP steels by delaying twin formation.^510^

##### 2.3.3.5 Multiphase Steels

High-strength steels, which consist of multiple microstructural phases such as martensite, austenite, ferrite, bainite, etc., are particularly susceptible to HE. The presence of various phases raises complications not only in H diffusion and trapping but also in crack initiation and propagation under H occlusion. For instance, austenite phases impede H diffusion and make the diffusion path tortuous and meandering.^208,511^ Because of different H diffusion and trapping properties, H concentration gradient can easily build up at phase boundaries and facilitate the debonding of interfaces by the assistance of dislocation interactions, through, e.g., H-enhanced and plasticity-mediated decohesion mechanism. During plastic deformation, different phases have to deform and harmonize to achieve a compatibility. However, H atoms alter the deformation mechanism in each phase that can easily create stress concentrations and lead to crack initiation. A single HE mechanism is often not sufficient to explain all the phenomena considering the interactions between H and different microstructural features. Therefore, the evaluation of HE mechanism becomes extremely complex and challenging.

A study on a 17% Cr martensitic-ferritic steel showed that both cleavage with small dimples and IG cracking were observed on fractography of a quenched sample, indicating that both the HELP and HEDE mechanisms operated in the fracture process.^512^ In comparison, when IG phases precipitated along the GBs after double tempering treatment, IG fracture became the predominant failure mode, which is dominated by the HEDE mechanism.^512^ X65 pipeline steel is a typical multiphase steel consisting of ferrite, bainite, and cementite. In an *in situ* electrochemical charging condition, abundant surface secondary cracks initiated at MnS-Al_2_O_3_ inclusions were observed during tensile testing.^202^ The crack initiation attributes to the accumulation of H atoms at the interfaces, reducing the cohesive energy as per the HEDE mechanism. TG cracks propagated along {110} plane traces and manifested as quasi-cleavage fracture, where the HELP and HESIV mechanisms can be applied regarding the restricted cross-slips and enhanced localized stress concentration.^503^ However, there exists an argument that inclusions are not critical during fatigue cracking in gaseous H condition, but pearlite morphology and its orientation with respect to the cracking direction are more relevant for FCG.^513^

Some TRIP-assisted steels also contain multiphases like ferrite, bainite, and metastable retained austenite; the latter can partially or fully transform to α′-martensite during mechanical loading.^514^ SSRT testing under an *in situ* electrochemical H charging environment resulted in cracks initiating primarily in or along martensite phases, while few cracks were seen in the ferrite and bainite grains.^514^ The cause of crack initiation in martensite is the supersaturation of H after phase transformation, as phase transformation-induced stress results in a significant number of defects near the phase boundaries, which act as weak sites for cracking.^514^ After forming in the martensite, the H-induced microcracks were observed to propagate into the ferrite, while in the H-free condition, microcracks were found in martensite without further propagation into the surrounding ferrite. Therefore, ferrite is better able to arrest cracks in this steel in the absence of H, possibly due to its higher capability of energy dissipation by plastic deformation without H.^514^

##### 2.3.3.6 Aluminum Alloys

Compared with steel and nickel alloys, HE in aluminum alloys is less investigated. Aluminum alloys manifest several advantageous attributes: lightweightness, high strength, corrosion resistance, and recyclability, positioning them as suitable candidates for myriad applications within the H value chain, encompassing production, storage, and transport. In H production, aluminum-water electrolysis, a methodology employing aluminum as a sacrificial anode, presents a clean and efficient avenue for H extraction from water. This process involves the electrochemical reduction of aluminum, generating H as a byproduct. Aluminum alloys are also considered promising for H storage applications, accommodating both gaseous and liquid H. Particularly, the weight-sensitive domain of aviation applications underscores the suitability of aluminum alloys for cryogenic H storage tanks in aircraft. Nonetheless, in all these applications, HE remains a significant threat to structural integrity. With aluminum alloys emerging as key materials in the development of a sustainable H economy, research interest on the topic of HE in these alloys has seen an escalation in recent years. This surge can be attributed to the fact that, historically, aluminum alloys were predominantly utilized in aerospace and construction engineering sectors, where HE might not have been perceived as the most immediate and critical safety concern.

The lattice diffusivity of H in aluminum is low, typical of other *fcc* materials such as nickel, while the perfect lattice solubility of H in solid aluminum alloys is small relative to other solid *fcc* materials.^515^ Except for residing in the lattice, H can also be trapped at dislocations and vacancies in pure aluminum^516^ and at second-phase particles and GBs in aluminum alloys.^318^ HE arises from the interaction of H with these microstructural defects.

Ferreira et al.^447^ investigated the influence of H on dislocation characters in pure aluminum with in-situ TEM and found that H promoted slip planarity by suppressing cross-slip of dislocations. This was attributed to H stabilizing edge dislocations. Lu et al.^452^ studied the influence with *ab initio* calculations and concluded that H enhanced dislocation mobility while inhibiting dislocation cross-slip, which is consistent with the observation made by Ferreira et al.^447^ This is an indication of the HELP mechanism. In another atomistic study, Lu et al.^517^ highlighted the crucial role of vacancies in HE of aluminum, claiming that H-induced superabundant vacancies and vacancy clustering on certain planes led to the formation of microvoids and microcracks. This conforms with the HESIV mechanism. Barnoush and Vehoff^518^ found that H promoted dislocation activities in aluminum via an *in situ* electrochemical nanoindentation approach. They further claimed that either the HELP mechanism or the HEDE mechanism may operate in H-induced fracture of the material. It should be mentioned that Xie et al.^466^ conducted *in situ* TEM observations on H-charged pure aluminum under micropillar compression and found that H impeded the motion of dislocations, and attributed the phenomenon to superabundant hydrogenated vacancies. Later, Xie et al.^519^ conducted *in situ* pre-notched cantilever bending experiment on single crystalline aluminum. They claimed that the H-promoted TG fracture was caused by H promoted formation of sub-GBs which then fractured due to the HEDE mechanism. In this model, they postulated that H promoted the nucleation of dislocations from the notch surface, in a way similar as the AIDE mechanism. Most recently, Li et al.^520^ conducted atomistic calculation in aluminum and revealed that vacancies are H traps and the trapping strength can be greatly enhanced by forming vacancy–solute complexes with addition of certain alloying element. A reduction in lattice strength was revealed associated with H trapping, which is an indication of the HEDE mechanism. It should be noted that this work did not touch upon the HESIV mechanism, the vacancies in the calculation were pre-existing and vacancy generation was not considered. While discrepancies still exist, it has been agreed upon that H suppresses cross-slip and promotes slip planarity of dislocations and that hydrogenated vacancies play a crucial role in aluminum. Notably, the above review covers virtually all experimental studies regarding the impact of H on the mechanical properties of pure aluminum, which is relatively scant compared to the abundant research on H in pure nickel. Because of this limited amount of research, it is hard to determine whether H in aluminum shifts the type of fracture from TG to IG.

Driven by practical needs, profound insights into HE in 7xxx series aluminum alloys have been achieved. Bond et al.^521^ observed H-enhanced dislocation mobility and induced softening of flow stress in age-hardened 7050 and 7075 Al-Zn-Mg alloys. Nguyen et al.^522^ studied a heat-treated 7050 alloy and claimed that embrittlement susceptibility correlated well with the size and type of matrix precipitates, while the role played by GB precipitates was relatively small. These studies highlighted the role of H–dislocation interactions in HE, relevant to the HELP mechanism. Takano^523^ reported both IG and TG fractures in H-charged 7050 alloy, attributing the former to IG H accumulation and the latter to H–dislocation interactions within grains.

**Figure 27:**
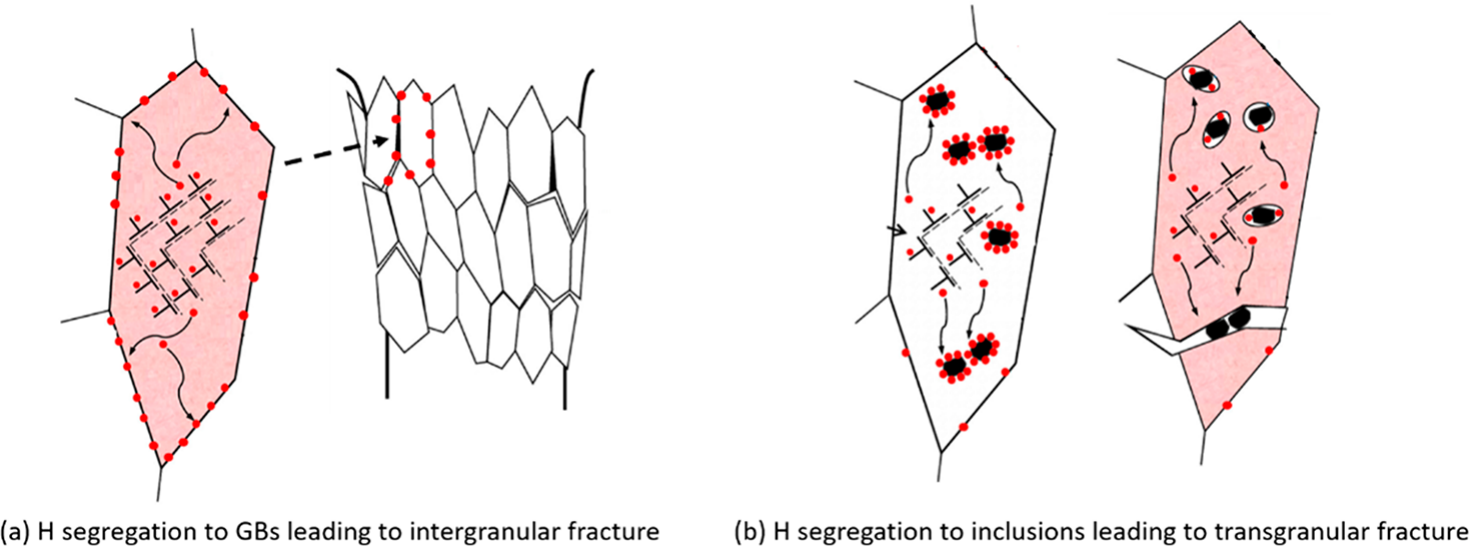
Schematic illustration of the HE mechanisms for IG and TG fracture in a 7xxx series aluminium alloy. Figure adapted with permission from ref (526). Copyright 2021 Elsevier.

The growing interest in green H and recent technological advancements, particularly the adoption of APT for precise mapping of H distribution in material microstructures, have enhanced the understanding of HE mechanisms in 7xxx series aluminum alloys. López Freixes et al.^524^ reported that H segregated to planar arrays of dislocations and to GBs in a 7449-T7651 aluminum alloy, with the former supporting the HELP mechanism and the latter the HEDE mechanism. Note that the synergistic action between the two mechanisms was not mentioned. Wang et al.^525^ investigated a 7xxx series aluminum alloy and observed H enrichment at both dislocations and GBs, suggesting that both the HELP mechanism and the HEDE mechanism were operational. The H-enhanced decohesion at GBs was claimed to be mediated by the HELP mechanism. Safyari et al.^526^ found that the fracture mode of a cold-rolled 7xxx aluminum alloy was dependent on the solution treatment condition. They attributed the IG fracture to the HEDE mechanism operating at GBs and the TG fracture to the decohesion of interfaces between the matrix and second phase particles inside the grains. Further, they emphasized the influence of particle coherency, interface, size, volume, and crystal structure on interfacial fracture.^527^ They claimed that the H-enhanced GB decohesion or interfacial separation at particles inside the grains were mediated by the HELP mechanism via dislocations transporting H atoms to the fracture sites. A schematic illustration of the governing HE mechanisms in these two scenarios was made by the authors and adapted in Figure 27. Zhao et al.^318^ utilized cryo-APT to map H and Mg distribution in a 7xxx aluminum alloy, finding H accumulation in intermetallic phases, Al3Zr dispersoids, and to a lesser extent, GBs. However, fracture occurred predominantly by decohesion of GBs, which was induced by the co-segregation of Mg and H in that region. The fracture mechanism can therefore be partly attributed to HEDE. In contrast, H enrichment at second-phase particles was found not to exert clear embrittlement effects, and the role of plasticity was not highlighted in that work. It is worth noting that the 7xxx alloys employed in the aforementioned studies possessed distinct chemical compositions.

Like other engineering alloys, the way aluminum alloys fracture due to HE is largely influenced by their chemical composition. Nevertheless, a consensus seems to have emerged from recent studies that decohesion induced by H segregation at GBs is an important factor contributing to HE in 7xxx series alloys.^318,524−526^ Therefore, the resistance of an aluminum alloy to HE can be improved by reducing the amount of H that segregates to GBs. This can be achieved by populating precipitates or second phase particles within the grains that can trap H more effectively, thereby facilitating a favorable repartition of H between the GBs and the grain interior. For instance, Wang et al.^525^ demonstrated that switching nanoprecipitates from the η phase to the T phase without changing the overall chemical composition effectively mitigated HE in a 7xxx aluminum alloy, attributed to the superior H trapping capacity of the T phase. Xu et al.^528^ reported that introducing Mn-rich intermetallic compound particles as strong H traps in a high strength aluminum alloy reduced HE susceptibility. Safyari et al.^529^ found that the addition of Zr to a 7xxx series aluminum alloy enhanced the material’s resistance to HE due to the high H trapping capacity of Al_3_Zr dispersoids.

##### 2.3.3.7 High Entropy Alloys

HEAs are novel alloys composed of multiple principal elements in near-equal atomic proportions. This unique composition creates a high degree of atomic disorder, leading to a stabilized solid solution structure with high entropy. Research interest on HEAs emerged recently, in 2004.^530,531^ Compared with conventional alloys, HEAs possess a number of superior mechanical properties under extreme conditions, such as high toughness at cryogenic temperatures, which makes them promising candidates for cryogenic H transport and storage applications, such as liquid H storage tanks.^532^ In such applications, the issue of HE needs to be carefully analyzed. Another promising application of HEAs is solid-state H storage,^533^ on which several review articles have been published over recent years.^534−536^ At present, the primary focus of the research is the capacity and kinetics of H absorption. Consequently, HEAs are often studied in powder form for their high surface area, and HE seems irrelevant. Yet, understanding HE is important for improving the efficiency and durability of solid-state H storage. For instance, the presence of local lattice distortions and dislocations in HEAs affects how much H they can store and release.^536,537^ These defects, which can form during hydrogenation cycles, impact the long-term performance of the materials.^538^ By studying these interactions, researchers can design better HEAs for H storage. Moreover, HEAs have the potential to act as both structural materials and H carriers. This dual functionality is particularly appealing in designing lightweight, efficient storage systems like conformal H tanks for aircraft. As these applications develop, understanding and managing HE in HEAs will be key to maximizing their performance and safety.

The exploration of HE in HEAs started gaining attention in 2017,^539,540^ with approximately 30 journal articles published thereafter. Li and Raabe’s review^531^ underscored the importance of studying H-induced degradation in HEAs. In the same year, Luo et al.^539^ discovered that H alloying in appropriate concentrations in an equiatomic CoCrFeMnNi HEA with *fcc* structure improved strength and ductility due to a reduction in SFE and enhanced nano-twinning. In their subsequent research, Luo et al.^541^ revealed that the through-thickness H diffusion gradient translated into a nano-twin gradient and effectively mitigated H-enhanced localized plasticity in the same material. Concurrent research by Zhao et al.,^540,542^ Kwon et al.,^543^ and Pu et al.^544^ found that CoCrFeMnNi HEA exhibited better resistance to H-induced fractures compared to conventional stainless and pipeline steels in various H environments. Rather than focusing on the effects of twinning, these studies linked the notable HE resistance of the material to fewer H trapping sites and lower H enrichment compared to traditional alloys. In other words, reaching the threshold H concentration necessary to initiate the HELP or HEDE mechanism is more challenging in the HEA. However, Luo et al.^545^ observed different effects when CrMnFeCoNi HEA was alloyed with interstitial carbon, where H charging notably reduced tensile ductility, resulting in a combination of IG, TG fractures, and MVC. In this scenario, QC and IG fracture facets were evident, the former attributed to H-induced slip localization on primary slip planes and the latter to HELP and HEDE-related GB fracture at very high H concentrations.

Recent advancements in research since 2019 have shed new insights on the susceptibility and underlying mechanisms of HE in HEAs. Yang et al.^546^ conducted nanoindentation experiments on H-charged CoCrFeMnNi HEA, revealing that H facilitates dislocation nucleation, in line with the Defactant concept. Contrasting findings emerged from other nanoindentation studies, which indicated that H impairs dislocation mobility in the same HEA,^547,548^ likely due to an increase in lattice friction caused by H atoms. Bertsch et al.^549^ observed H-induced IG failure in FeNiCoCrMn HEA. Their findings suggest that H assists in forming dislocation substructures like dislocation cells and increases deformation band density via the HELP mechanism. This dislocation substructure caused strain incompatibilities across GBs by hindering their rotation, leading to IG fracture. Initially, the material lacked dislocation substructure, allowing H to segregate to GBs via dislocation transport during early and intermediate loading stages. The fractures were concluded to result from H-enhanced and plasticity-mediated decohesion.

Koyama et al.^550^ found that grain refinement enhanced the ultimate strength of H-charged CoCrFeMnNi HEA without further reducing the elongation, as compared to the H-charged untreated HEA. The work highlighted the role of dislocation impingement at GBs and H transport from grain interior to GBs with dislocation motion, indicating a synergistic action of the HELP and HEDE mechanisms. Grain refinement was thought to alleviate stress concentration at GBs, thus reducing H-assisted IG fracture. Furthermore, a grain-refined CoCrFeNi HEA pre-charged with 100 MPa H gas showed ductile fracture after necking and higher elongation than that of H-charged grain-refined CoCrFeMnNi HEA, which is perhaps owing to the removal of disadvantageous Mn effect on GB strength.^551^ Fu et al.^552^ investigated HE in a CoCrFeMnNi HEA fabricated via selective laser melting (SLM). SLM processing produced cellular dislocation structures with high density, leading to GB cracking when proximal to GBs. Controlled annealing retained these structures while significantly lowering dislocation density, enhancing HE resistance. This improvement was linked to the formation of nano-twins in “clean” cellular structures and restricted dislocation movement within these structures, reducing dislocation impingement and H segregation at GBs. Fu et al.’s^552^ clean cellular structure, interpreted as grain refinement through additive manufacturing (AM) and annealing, aligns with Koyama et al.’s^550^ concept that grain refinement helps improve HE resistance of HEAs. Notably, Koyama et al.^550^ also emphasized on optimizing annealing conditions for effective grain refinement, because σ phase forms by annealing at low temperatures (e.g., 700 °C for the CrCoFeMnNi HEA) and the interface between the σ phase and matrix acts as a preferential site for H-related cracking.

As a brief summary, studies on HE in HEAs have predominantly centered on CoCrFeMnNi HEA, demonstrating IG fracture as the primary fracture mode under H influence. Dominant HE mechanisms identified include HELP and H-enhanced and plasticity-mediated decohesion. Strategies to augment HE resistance involve reducing dislocation impingement and H segregation at GBs through grain refinement or dislocation cellular structure engineering. Additionally, H’s role in reducing SFE and promoting deformation twinning in HEAs, particularly in CoCrFeMnNi, is crucial for enhancing HE resistance, as discussed in recent literature.^553−555^ A handful of studies have been performed on other types of HEAs. Zhang et al.^556^ investigated an Al0.25CoCrFeNi HEA with an *fcc* structure, claiming immunity of the material to HE due to slow H uptake and H promoted planar dislocation slip. However, H only reached a shallow depth in the test specimen in this work, the performance of the material at high H concentrations needs to be examined. Cheng et al.^557^ studied a (FeCoNi)_86_Al_7_Ti_7_ HEA, and attributed H induced IG fracture to the HELP mechanism and the HEDE mechanism. Chen et al.^558^ studied a precipitation strengthened FeCoCrNi HEA and reported good resistance of the material to HE, because of nano-sized NbC precipitates that suppress dislocation transport of H and facilitate the formation of nano-twins. Interestingly, VNbMoTaW HEA’s *bcc* structure was exploited in producing fine powders via H environment annealing, leveraging HE-induced brittleness, though the HEDE mechanism was suggested but not empirically confirmed.^559^ For more information on H-induced failure of HEAs, the reader is referred to a review article by Li et al.^560^

##### 2.3.3.8 Additively Manufactured Materials

In recent years, AM techniques have been increasingly utilized for component fabrication in H technologies, attributed to AM’s inherent capability to produce complex geometries. An example is the manufacturing of bipolar plates for proton exchange membrane technology relevant to H applications. However, a significant challenge in deploying AM for this technology is the susceptibility of components, notably bipolar plates, to HE.^561^ Moreover, the application of AM extends to producing components for H-fuelled gas turbines, such as turbine blades, which also face H-related failures under a combination of environmental factors.^562^ Another promising application of AM is in the development of storage tanks with intricate structural designs, such as the conformable H storage tanks mentioned earlier. All these applications necessitate a robust understanding and mitigation of HE in AMed alloys.

As AMed materials possess distinctive microstructural and surface characteristics from conventionally produced materials; this section categorizes materials based on their manufacturing approach rather than the predominant mechanical composition. This section encompasses several prevalent AMed alloys, including austenitic stainless steel, precipitation-hardened stainless steel, maraging steel, nickel-based superalloy, titanium alloy, and HEA. Although the present Review excludes the discussion of hydride-based HE mechanisms, findings related to the widely used AMed Ti-6Al-4V alloy are revisited in this section, given the extensive research data available on that alloy. It is acknowledged that a comprehensive review categorizing AMed alloys based on their primary chemical composition holds significance, and this has been done by Yao et al.^563^ The present section seeks to avoid repetition of that review, instead focusing on the discussion of microstructural phases and patterns, anisotropy, phase transformation, residual stress, and surface finish and defects in relation to HE.

*Microstructural Phases*. AM processes are distinguished by their rapid cooling rates, which can significantly alter the resultant microstructures compared to those of conventionally manufactured counterparts. For example, duplex stainless steels consist of a fully ferritic microstructure in the as-built condition in laser powder bed fusion (L-PBF).^564^ It must be noted that the altered phase balance in certain AMed alloys is not solely due to rapid cooling. Other factors, including partial segregation or even the evaporation of specific alloying elements during the printing process, also play a crucial role in determining the final microstructure.^565^

The Ti-6Al-4V alloys consist of an α/β microstructure in its wrought condition and usually after electron beam melting (EBM). However, when produced by L-PBF, these alloys manifest a predominantly acircular α' martensite microstructure.^566^ Kong et al.^567^ analyzed a L-PBFed Ti-6Al-4V in the as-built condition but with a stress-relief treatment at 300 °C for 2 h. This martensitic microstructure did not show any sign of hydride formation or HE, which is explained by the low solubility and diffusivity of H in the absence of β phase. On the other hand, after a heat treatment producing a Widmanstätten α/β structure, H uptake was enhanced, hydrides were formed at the α/β interface, and HE was triggered. This exemplifies how a brittle and hard microstructure (α') may be less prone to HE. Nevertheless, contrasting results were obtained by Kacenka et al.^568^ where the as-built L-PFBed product suffered a strong HE effect after annealing, though the involvement of β phases and their influence on hydride formation were not detailed in that work. The presence of defects and internal stresses was mentioned as a possible cause of the high HE susceptibility, but the exact mechanisms remain unclear.

Low-carbon steels can develop a dual-phase microstructure following conventional manufacturing processes, e.g., ferritic-pearlitic. In steels fabricated with AM, a predominantly ferritic structure is observed. This is the case for the Fe-(1.8–2.1)Mn-(0.7–1.0)Si-(0.05–0.11)C low-carbon steel, assessed in the cast condition and manufactured by EBM.^569^ The cast steel was ferritic-pearlitic, whereas the material produced by EBM was fully ferritic and possessed a better HE resistance. The high HE susceptibility of the cast variant was attributed to the platelet structure of pearlite and H trapping at carbides, leading to increased H concentration. This observation seemingly contrasts with other research indicating a protective role of carbides as strong traps mitigating HE, as suggested by other authors.^570,571^

The 17–4 precipitation-hardened stainless steel fabricated via L-PBF exhibits a microstructural divergence from its conventionally produced counterpart, too. Typically, wrought 17–4 precipitation-hardened stainless steel has a martensitic microstructure. However, the rapid cooling rates associated with L-PBF inhibit austenite formation and consequently the martensitic transformation, resulting in a δ ferritic microstructure.^572^ Despite that the L-PBFed microstructure is ferritic, H permeation in L-PBFed 17–4 precipitation-hardened stainless steel is expedited due to the lower density of GBs compared to the wrought version, possibly contributing to its increased HE susceptibility. This higher susceptibility is also attributed to the coarser grain size, which corresponds to a lower cleavage stress. HE takes place as IG fracture in the martensitic wrought microstructure, with crack propagating along prior austenite GBs, but as TG fracture in the ferritic L-PBFed version.

In AM, as-built materials frequently necessitate further treatment to optimize mechanical properties and relieve residual stress, making heat treatment a common post-processing practice. The interaction between precipitates and H trapping, as well as crack initiation, is particularly important. Mei et al. studied H trapping in L-PBFed 18Ni-300 maraging steel after various aging treatments.^573^ Precipitates formed at higher aging temperatures were found to have a higher H binding energy, as characterized by TDS. However, the precipitate size thus the interfacial area of matrix–precipitate was reduced at higher aging temperatures, resulting in lower trap densities. This trap density reduction might be attributed to the decomposition of dislocation cell walls. In addition, the over-aged condition, with its reverted austenite, tended to be more ductile, but this effect was counterbalanced by the high diffusivity due to the dissolution of dislocation cell walls and the consequently higher H concentrations. In a GH4169 nickel-based alloy produced by L-PBF, precipitates were also found to play a crucial role in HE. Xu et al. characterized δ/γ interfaces as H trapping sites with a high energy and density.^574^ They suggest that diffusible H moves along dislocation slip bands, encouraging dislocation accumulation, breaking δ precipitates, and ultimately leading to microcrack initiation and propagation. Since δ precipitation occurred within the grain as well as at GBs, both TG and IG cracks were observed. Additionally, the significance of Laves phases in trapping H and acting as preferred cracking sites has been documented for nickel alloys produced by L-PBF^575,576^ and by wire arc additive manufacturing (WAAM).^577^

*Microstructural Patterns*. One of the most interesting features of AMed microstructures is the appearance of sub-grain cellular structures or dislocation cells. Typically, these cell walls are constituted of dislocation tangles or occasionally segregated elements.^578^ The impact of such substructures on HE mechanisms has been explored across various materials. Solidification and element segregation also result in melt pool boundaries (MPBs), which mark the fusion line during layering in AM processes. The influence of MPBs on pitting corrosion is documented,^579^ and in some instances, segregation of corrosion-resistant elements at these boundaries can enhance corrosion resistance.^580^ However, research on the specific effects of these microstructural features on HE is still limited. Furthermore, the epitaxial growth of columnar dendrites, another signature of AM processes, also has an influence on HE, which is discussed later along with the overall role of anisotropy.

The role of cellular structures on H diffusion and trapping is not clear. Claeys et al.^581^ found in a L-PBFed 316L that heat treatment at 1066 °C for 1 h reduced the dislocation density and led to an increase in H diffusivity, despite the retaining cellular structure. This suggests that cell walls might act as rapid channels for H movement, a stark contrast to the behavior seen in cold-rolled and annealed materials. In that study, the heat treatment was observed to remove MPBs from the initial build, but their potential effects on HE were left unexplored. In an L-PBFed Inconel 718,^582^ H-induced fracture was observed to occur both by decohesion of MPBs and by layer delamination at the H-charged surfaces. Other studies suggest that while MPBs may serve as initiation sites for H-assisted cracking, they are not deemed the dominant factor of HE.^583,584^

**Figure 28:**
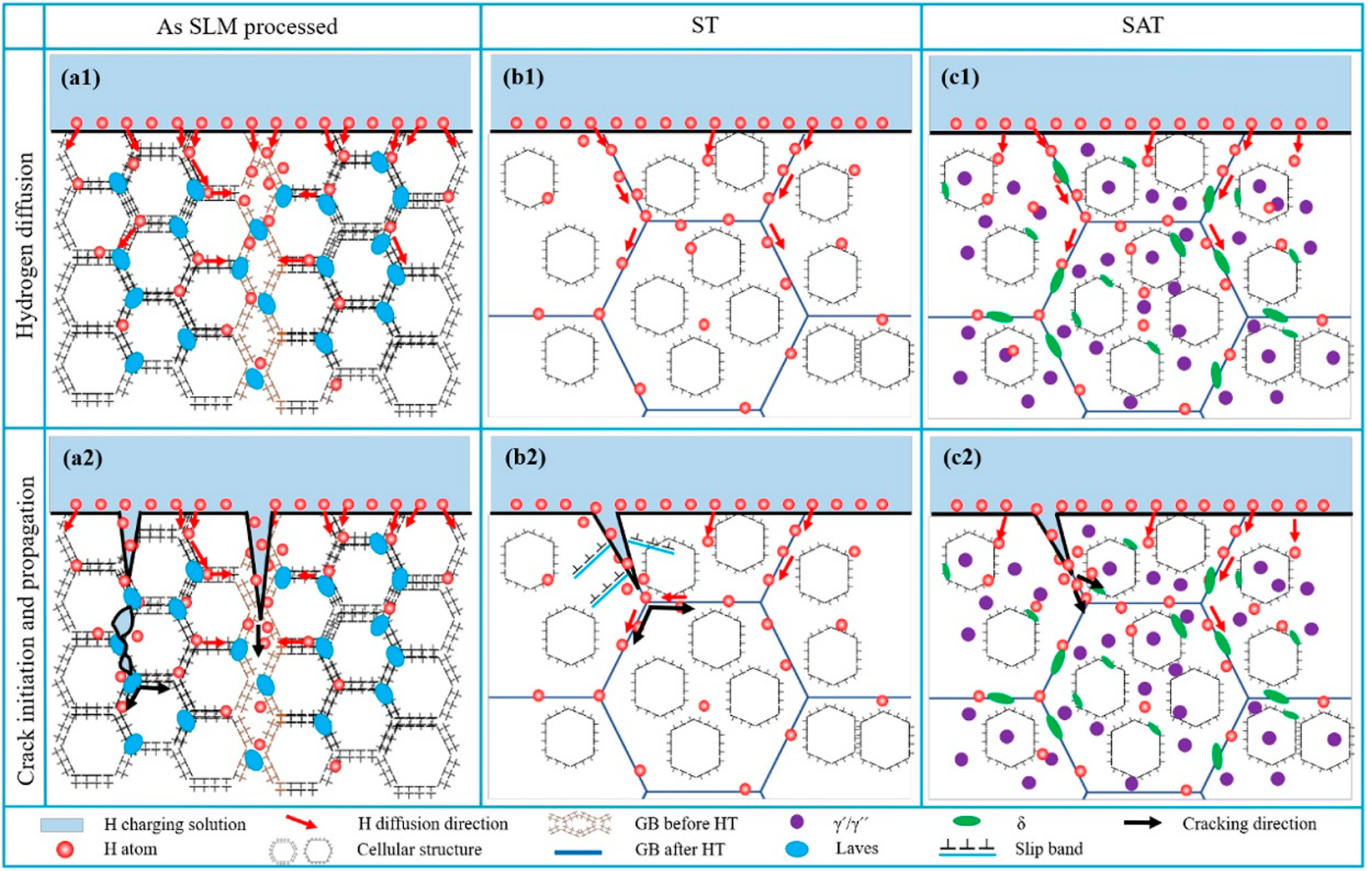
Diffusion and HE mechanisms in an Inconel 718 for the as-built condition (as SLM processed), and after two heat treatments: solution treatment and solution aging treatment. Adapted with permission from ref (575). Copyright 2023 Elsevier under [CC BY 4.0 DEED] [https://creativecommons.org/licenses/by/4.0/].

The role of sub-grain structures as fast diffusion paths were also noted by Lin et al.^585^ in L-PBFed 316L steel. However, the permeation tests in that work showed some noise and it was questionable whether a steady state was reached. Short-circuit diffusion through cell walls was also noted for AMed nickel-based alloys^575^ and for HEAs.^552^ The effects of heat treatment on H transport and H-assisted cracking were assessed by Xu et al.^575^ for an Inconel 718 produced by L-PBF. As shown in Figure 28, the HE susceptibility of the as-built material is caused by the cell structure which has a high density of dislocations and Laves phases. An enhanced diffusion along these cell boundaries is expected. On the other hand, solution treatment reduced both dislocations and Laves phases. Hence, HE is governed by GB diffusion and cracking in that situation. Finally, precipitates after aging produce H accumulation in both GBs and dislocation cells and lead to a combination of IG and TG fracture.

The role of cell walls as fast H diffusion channels is particularly important for low-H diffusivity alloys. However, the presence of dislocations and precipitates within these cell walls might concurrently act as H retention sites, counteracting the role as rapid diffusion channels. Metalnikov et al.^584^ compared TDS profiles between a 316L steel produced via L-PBF and cold-rolling, noting dual peaks in the L-PBFed specimen. The lower energy peak was associated with elastic stress fields or dislocation cores, while the higher energy peak (62 kJ/mol) was linked to dislocation cell walls, a feature less prominent in cold-rolled specimens. In contrast, binding energy at cell walls was characterized by TDS to be around 27 kJ/mol for a L-PBFed 18Ni(300) maraging steel.^573^ In that study, cell walls were formed of dislocation tangles and Ni segregation, which promoted reversed austenite near the cell walls. Zhou et al.^586^ reported that heat treatment on L-PBFed 18Ni(300) maraging steel, which removed cellular structures, and consequently enhanced H diffusion by reducing trapping and increased HE susceptibility. In addition, the formation of some precipitates acting as reversible traps increased the amount of diffusible H. Direct aging treatment led to austenite formation near the molten pool boundaries due to the segregation of nitrogen, affecting H distribution and cracking at austenite/martensite interfaces. Lee et al.^587^ noted a similar trend in L-PBFed Inconel 718, where direct aging led to increased HE susceptibility due to H accumulation at GBs rich in precipitates. The cellular structure acted as failure initiation sites due to the cracking of precipitates at these cell boundaries, while their influence on H uptake and diffusion was considered negligible.

The multifaceted dynamics between H and cellular structures have been further elucidated by Hong et al.,^588^ who examined the role of deformation twinning within the sub-grain cellular structure in L-PBFed 316L stainless steel. Strain-induced martensites were observed at the intersection between twins and cell walls, which contributed to H accumulation and embrittlement. Additionally, their study attributed HE in a precharged specimen to the combined effect of HELP and H transport by dislocations, which enhanced both H and dislocation accumulation at twin boundaries, eventually leading to interfacial decohesion via the HEDE mechanism.

It is important to recognize that dislocation cells can manifest without the segregation of alloying elements, as demonstrated by He et al.^589^ in pure nickel fabricated by L-PBF. Notably, these cells dissipated following an annealing treatment at 550 °C for 3 hours. The as-L-PBFed material with dislocation cells had a higher HE susceptibility compared to the annealed specimen and the wrought material. Another important note is that the cellular structure varies between different AM approaches. Bertsch et al.^590^ compared two AM methods for a 316L stainless steel, L-PBF and direct energy deposition (DED). One of the most interesting findings is that the cellular structure is different in DED than in L-PBF. H was found to stabilize dislocation cells and impede their reorganization in the material produced with DED, resulting in a higher HE susceptibility. This is attributed to the smaller size and equiaxed nature of dislocation cells in DED, in contrast to the elongated dislocation cells in L-PBF. This work emphasizes the influence of dislocation arrangement, depending on their size and morphology, on HE susceptibility.

*Anisotropy and Directionality*. Few studies have systematically evaluated the effect of printing orientation on HE. Nonetheless, it is recognized that AM processes typically foster the epitaxial growth of columnar grains along the build direction, leading to anisotropy and directionality which have an influence on H diffusion and HE. Álvarez et al.^591^ conducted an extensive study on the impact of printing orientation on HE susceptibility in 316L stainless steel fabricated through L-PBF. They specifically looked at the material in its as-built state and after various thermomechanical post-processes. The findings indicated that samples printed vertically, where the load direction aligns with the building direction, exhibited increased ductility and softer mechanical response. A pronounced orientation influence on HE susceptibility was evident in the as-built condition, diminishing significantly after treatments like annealing or hot isostatic pressing (HIP). This observation is likely due to the orientation of grains along the build direction in the as-built material and the interaction of H with GBs and oriented dislocation patterns.

In an Inconel 718 produced by laser direct forming, the anisotropic nature of HE is manifested in two aspects: first, the presence of columnar dendrites that hinder H diffusion, leading to lower H concentrations in vertically oriented samples, which are parallel to the deposition direction; second, an anisotropic HE response attributed to the alignment of Laves phases, influencing the dominant cracking mechanism. Specifically, H-assisted cracking begins in the γ matrix and takes a zigzag path in vertical orientations, while cracking is initiated by the decohesion between the Laves phases and the matrix in horizontal orientations. Hesketh et al.^592^ claimed that H diffusion in horizontally printed samples is facilitated by elongated grains, persisting even post-heat treatment. It is also important to note that horizontal specimens exhibit inherent brittleness even in the absence of H, which is attributed to the orientation-specific cooling process and resultant precipitate distribution. Feng et al.^577^ observed similar columnar dendrites in Inconel 718 produced via WAAM, which markedly slowed down H diffusion perpendicular to the building direction. Mohandas et al.^469^ tested hollow specimens of L-PBFed Inconel 718 in H gas at 150 bar. They noted a superior resistance in the L-PBF material, loaded parallel to the building direction, compared to the wrought condition, which is attributed to the microstructural anisotropy in the as-built material. The elongated grains along the building direction influence crack propagation parallel to the load, thereby delaying cross-sectional failure.

Orientation effects have been studied for Ti-6Al-4V produced by L-PBF. Deconinck et al.^593^ observed an increase in H diffusion and uptake in horizontally built orientations. This phenomenon was ascribed to the improved H transport through boundaries between prior β grains, despite a microstructure predominantly comprising acicular martensite. They also noted that variations in porosity and dislocation density, stemming from different cooling rates in various building directions, complicate the identification of a unique HE mechanism. Wu et al.^594^ found that HE susceptibility was accentuated when the tensile sample’s thickness aligned with the building direction, correlating this with increased hydride formation. Interestingly, this orientation also exhibited inferior mechanical properties in air, attributable to interlayer defects which likely exacerbate HE. The print direction not only influences hydride formation but also the H trapping mechanism and energy, as indicated by XRD and TDS analyses in the study by Silverstein and Eliezer.^595^ In that work, the orientation with higher HE susceptibility was explained by the lower binding energy and thus the higher amount of diffusible H.

For dual-phase alloys produced by AM, a stronger influence of orientation is expected. Wan et al.^596^ studied an AlCoCrFeNi2.1 eutectic HEA produced by laser engineered net shaping (LENS). They found that the local orientation of *bcc*/*fcc* layers relative to the loading direction has a significant influence on crack initiation and propagation in H-charged specimens.

*Phase Transformation and Composition*. As discussed earlier, the HE susceptibility of austenitic stainless steels is closely linked to the formation of strain-induced martensites. Therefore, a change caused by AM in the sensitivity to this transformation is expected to modify HE resistance.

The different sensitivity of AMed austenitic stainless steels to strain-induced martensite transformation is typically ascribed to two factors, the altered composition of the powder compared to conventionally manufactured materials, and the unique dislocation cell structure. For instance, L-PBFed 316L steel examined by Claeys et al.^581^ had a slightly different composition than the reference cold-rolled and annealed condition, resulting in a higher austenite stability, i.e., less prone to martensitic transformations. This enhances the resistiance of the AMed 316L steel to HE. The austenite stability towards martensitic transformation is usually assessed by three parameters,^584^ namely an austenite stability factor, the Md30 temperature and the SFE, all of which are composition-dependent. For example, increasing Ni content in 316L powder significantly improved the HE resistance of an L-PBFed stainless steel, which was attributed to the effect of Ni as a strong austenite stabilizer.^583^ Álvarez et al.^591^ observed that strain-induced martensite transformation was suppressed in a L-PFBed 316L stainless steel as compared to the wrought version. This difference in austenite stability between AMed and wrought specimens is attributed to their distinct compositions, as evidenced by the lower Md30 temperature in the former. It is noted that strain-induced martensites still occurred during hydrogenation, as revealed by the XRD peaks related to α′ and ε martensite, but this transformation was less pronounced in the AMed material. Kong et al.^597^ pointed out that the high density of dislocations and the fine cellular pattern in L-PBFed 316L increased the stress required for twinning, thus reducing martensitic transformation and enhancing HE resistance. Other factors than dislocation and composition may contribute to the suppressed formation of SIM. Lee et al.^598^ found that α' martensites were larger in size than the sub-grain structures in an AMed 304L austenitic stainless steel, implying that dislocations might not play a crtical role in the strain-induced martensite formation. Moreover, the chemical composition of the AMed 304L steel closely matched that of conventionally manufactured material, reflected in comparable Md30 temperatures. Consequently, the enhanced austenite stability noted in AMed specimens is not attributed to compositional differences. The authors ascribed this stability to a more uniform distribution of alloying elements in AMed samples, facilitated by the rapid cooling rate in AM processes that suppresses δ ferrite precipitation. This is in contrast to conventional casting, where retained δ ferrite depletes nickel from nearby austenite and increases its propensity to form strain-induced martensite.

In contrast to AMed austenitic steels, AMed maraging steels typically exhibit greater HE susceptibility. Li et al.^599^ suggested that H decreases the critical stress needed for martensitic transformation of retained austenite in L-PBFed 300 steel, leading to premature phase transformation and higher HE susceptibility. Similarly, Strakosova et al.^600^ reported substantial HE in L-PBFed X3NiCoMoTi 18-9-5 maraging steel with a fully martensitic microstructure after heat treatment, H trapping at defects was suspected to be the main cause. HE of a 18Ni-300 maraging steel produced by SLM was also attributed to H trapping,^601^ in particular due to retained austenite. It was postulated that γ/α' interfaces acted as high H concentration and crack initiation sites in the as-built condition. Solution treatment was found beneficial for high HE resistance by lowering γ content and dislocation density.

*Residual Stresses*. The role of residual stresses in affecting H uptake and embrittlement in AMed alloys is a critical factor that is often underexamined, despite that they are very likely to exist due to rapid cooling and thermal gradients during fabrication. Residual stresses are posited to influence HE susceptibility, as noted by Li et al.,^599^ but comprehensive measurements are seldom reported in literature. Heat treatment enhancing HE resistance in AMed components can be partly attributed to the mitigation of the residual stresses, as found by Claeys et al.^581^ in a L-PBFed 316L steel. Nevertheless, quantifying the contribution of residual stress reduction is challenging as heat treatment simultaneously alters dislocations. Moreover, post-processing techniques like ultrasonic nanocrystal surface modification (UNSM) have been observed to increase HE resistance in L-PBFed Inconel 625.^602^ The authors claim that compressive residual stresses could reduce H diffusion, although this effect might be produced by trapping at dislocations due to deformation as well.

Feng et al.^603^ measured a reduction in residual stresses after annealing and especially after HIP for a L-PBFed CoCrFeNiMn HEA. The residual stress relief is one of the causes for a lower HE susceptibility, while the authors noted that the reduction in dislocation density and defects (pores and microcracks) could also contribute to the HE mitigation. Heat treatment was also found to improve fatigue resistance in H due to the stress relief.^576^ H-accelerated FCG in a L-PBFed Inconel 718 was explained by H-induced suppression of dislocation activity and a shift to IG fracture.

While heat treatment is effective in alleviating macro-stresses, residual stresses particularly along GBs may persist due to incomplete recrystallization, potentially escalating HE.^604^ Large residual stresses can also be associated to the cellular structures near fusion boundaries, as noted by Fu et al.^552^ in a L-PBFed CoCrFeMnNi HEA. The dislocation cell walls were considered as weak traps for H, potentially acting as fast diffusion channels and crack initiation sites. Similarly, Cheng et al.^557^ obtained the local misorientation map in an L-PBFed CoCrFeNiMn alloy as an indicator of residual stresses. The residual stress distribution was found higher near the GBs and fusion boundaries, which explains the observed interfacial cracking along these boundaries.

*Surface Finish and Defects*. Parts produced via AM in the as-built condition are characterized by a substantially high roughness. However, the influence of surface finish on H uptake in AMed alloys remains under-explored. Claeys et al.^581^ found for a L-PBFed 316L that polished surfaces increase HE susceptibility. This is attributed to the lower H concentration in as-built samples where the rough surface offers an expanded area, resulting in a diminished effective charging current. However, the increased surface roughness and consequently, effective area of as-built L-PBF samples, lead to an augmented H uptake in Ti-6Al-4V.^593^

For specimens printed with a small thickness, the influence of surface defects is especially worthy of consideration. Khedr et al.^605^ analyzed HE of auxetic lattice structures printed in 316L stainless steel and found an increasing resistance for smaller strut thicknesses, which was attributed to the higher amount of surface flaws that acted as irreversible traps. In thinner printed structures, expedited cooling leads to lattice shrinkage, engendering microcracking and surface defects. Such structures also exhibit increased surface roughness. Conversely, thicker structures are distinguished by a rise in H-induced mechanical twinning, with consequent cracking along twin boundaries, thereby exacerbating HE susceptibility.

The influence of porosity, a typical outcome of AM processes, on H trapping and embrittlement needs further exploration. A critical role of porosity was found in crack initiation and propagation in L-PBFed Ti-6Al-4V.^606^ These voids were hypothesized to act not only as stress concentrators but also as H traps. Particularly, the accumulation of H in pores near the surface was deemed critical in facilitating crack initiation and consequently promoting HE. The reduction of porosity by HIP post-processing has a significant role in improving fatigue behavior, but its influence on HE is not fully established. Álvarez et al.^591^ found that while HIP enhanced HE resistance more than annealing, it was difficult to isolate the effects of porosity reduction from the broader microstructural changes HIP induced. Due to the longer exposure to 1100 °C, HIP resulted in a more evenly distributed and coarser grained structure compared to annealing. This structural change may also contribute to better HE resistance.

##### 2.3.3.9 Summary of the Mechanisms and Materials

In summary, we have demonstrated H-assisted fracture across a spectrum of critical metallic materials: *bcc* iron and steels, *fcc* nickel and its alloys, along with advanced steels exhibiting phase transformation and twinning, multi-phase steels, aluminum alloys, and HEAs. We have highlighted the typical fracture patterns and the main HE mechanisms for each material type. It is important to note that these examples are illustrative of the HE mechanisms we’ve discussed, and not an exhaustive list of all engineering alloys. In particular, the category of steels, due to its extensive diversity, presents a formidable challenge for a complete review that interlinks all material types with experimental conditions and HE mechanisms within the scope of a general review like this. For in-depth exploration of HE in specific steel types, readers are directed to specialized review articles, for instance, on pipeline steel and its welds,^607−610^ austentic steel,^611−613^ ferritic steel in general,^614^ and advanced high strength steel.^7,615^ Note that there can be overlapping among these steel categories, which again, underscores the complexity inherent to steels.

TG and IG fracture modes, while prevalent across practically all metals and engineering alloys, exhibit case-specific mechanisms influenced by distinct microstructural attributes, loading condition, and H environment. For a quantitative assessment of HE, it is crucial to apply criteria tailored to the material in question. These criteria should encompass: (a) the H distribution profile both before and under mechanical loading; (b) critical microstructures with diverse crystallographic orientations and mechanical properties, prone to stress and strain localization; (c) microstructures susceptible to phase transformation, enhancing lattice misfit and local H concentration; (d) the dynamic interplay between H diffusion and dislocation activities under specific loading scenarios. The cornerstone for such criteria is a complete database that correlates HE mechanisms with material type, microstructure, loading conditions, and H environment. A table summarizing steels and relevant HE mechanisms was provided in a review by Djukic et al.,^40^ which is a good start towards a more comprehensive mapping of HE mechanisms to these factors, thereby facilitating material-wise criteria for HE evaluation.

#### 2.3.4 Probing HE Mechanisms by Experimental Approaches

The investigation of HE encompasses multiple spatial scales, extending down to the atomic level, and spans a broad temporal range, potentially reaching years. This complexity has necessitated the development and employment of a diverse array of multiscale experimental methodologies aimed at elucidating the embrittlement mechanisms. The intricate nature of HE has consistently driven the demand for innovative experimental techniques, contributing significantly to advancements in the field. A typical example is the application of environmental TEM for the *in situ* probing of dislocation motion in the presence of H.^616,617^ Such experimental practice, which dates back to the 1980s, has revealed a number of important observations that later served as key evidences for the operation of the HELP mechanism.^616,617^ Traditional and still the most widely used experimental approaches for studying HE mechanism rely on post-mortem characterization on samples that are deformed or fractured in the presence of internal or external H.^495,618,619^ This can provide important insights on the process of H-induced defects and damage evolution. Nonetheless, the dynamic nature of H interaction with defects and subsequent damage progression underscores the significance of *in situ* characterization techniques. These methods facilitate real-time observation of microstructural alterations under H exposure and mechanical loading. Key challenges include establishing a controlled H atmosphere during microstructural examination, often under simultaneous loading conditions. Techniques that have been developed by far include *in situ* environmental TEM, *in situ* (environmental) SEM, *in situ* electrochemical small-scale mechanical testing, and *in situ* synchrotron high-energy X-ray diffraction. In this section, these *in situ* approaches and their associated findings in the field of HE will be systematically reviewed. Their advantages and disadvantages in terms of unraveling HE mechanisms will also be discussed.

##### 2.3.4.1 *In Situ* Environmental TEM

**Figure 29:**
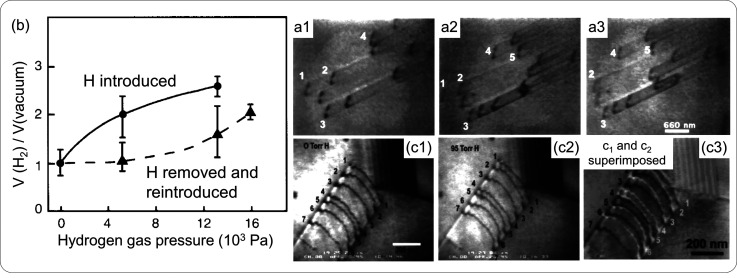
The behavior of dislocations in a constantly strained α-Ti (Ti-4 wt. % Al) under (a_1_) vacuum and (a_2_, a_3_) ∼13 kPa H_2_ gas (the numbers identify the same dislocation). Reprinted with permission from refs (617 and 623). Copyright 1988, 2012 Elsevier. (b) The influence of introduced H gas pressure on the dislocation velocity (*V* (H_2_)) in the same material. Reprinted with permission from refs (617 and 623). Copyright 1988, 2012 Elsevier. (c) The influence of H on the separation distance between pile-up dislocations in a 310S stainless steel (the sample was loaded to certain stage and the deformation was kept constant during the introduction of H and imaging; (c_1_) pile-up dislocations under vacuum, (c_2_) pile-up dislocations under ∼12 kPa (95 Torr) H_2_ gas, (c_3_) superimposed image made from c_1_ and negative c_2_ (white dislocations)). Reprinted with permission from ref (629). Copyright 1998 Elsevier.

Environmental TEM (ETEM), which was developed in the 1950s,^620^ provides an opportunity to observe deformation-driven evolution of dislocations and damage under the presence of H atmosphere. The differential pumping scheme attached to the ETEM allows a gaseous environment with a pressure up to the order of ∼10^4^ Pa.^617,621^ The dissociation and ionization of the gas molecules by high-energy electron beam enables a much higher H (MPa level) fugacity close to the sample surface.^622,623^ First studies using such technique for HE research were reported in the 1980s and 1990s. In those studies, the influence of H on a variety of model metals/alloys including pure Fe^624^ and austenitic stainless steels,^625^ Ni and Ni-based alloys,^626^ Al and Al alloys,^627^ Ti alloys,^623^ and Ni_3_Al alloys^628^ was probed. One of the most intriguing findings from these works is the effect of H on dislocation motion, as exemplified in Figure 29. It was observed in *hcp* α-Ti that during constant displacement loading, dislocations started to move upon the introduction of H gas (pressure ∼13 kPa, Figure 29a_1_–a_3_) and stopped moving when the gas was removed from the cell. Further quantitative measurement revealed a promoting role of H on dislocation mobility (Figure 29b). Interestingly, such promoting effect was the most pronounced for the case when H was firstly introduced and became weaker when H was removed and reintroduced (Figure 29b). In austenitic stainless steels, it was also found that the separation distance between pile-up dislocations was reduced (i.e., dislocations were closer) due to the introduction of ∼12 kPa H_2_ gas (Figure 29c). These experiments provided direct supporting evidence for the HELP mechanism which proposes that H reduces the repulsive force acting between dislocations and other obstacles and thus accelerates dislocation motion.^41^

**Figure 30:**
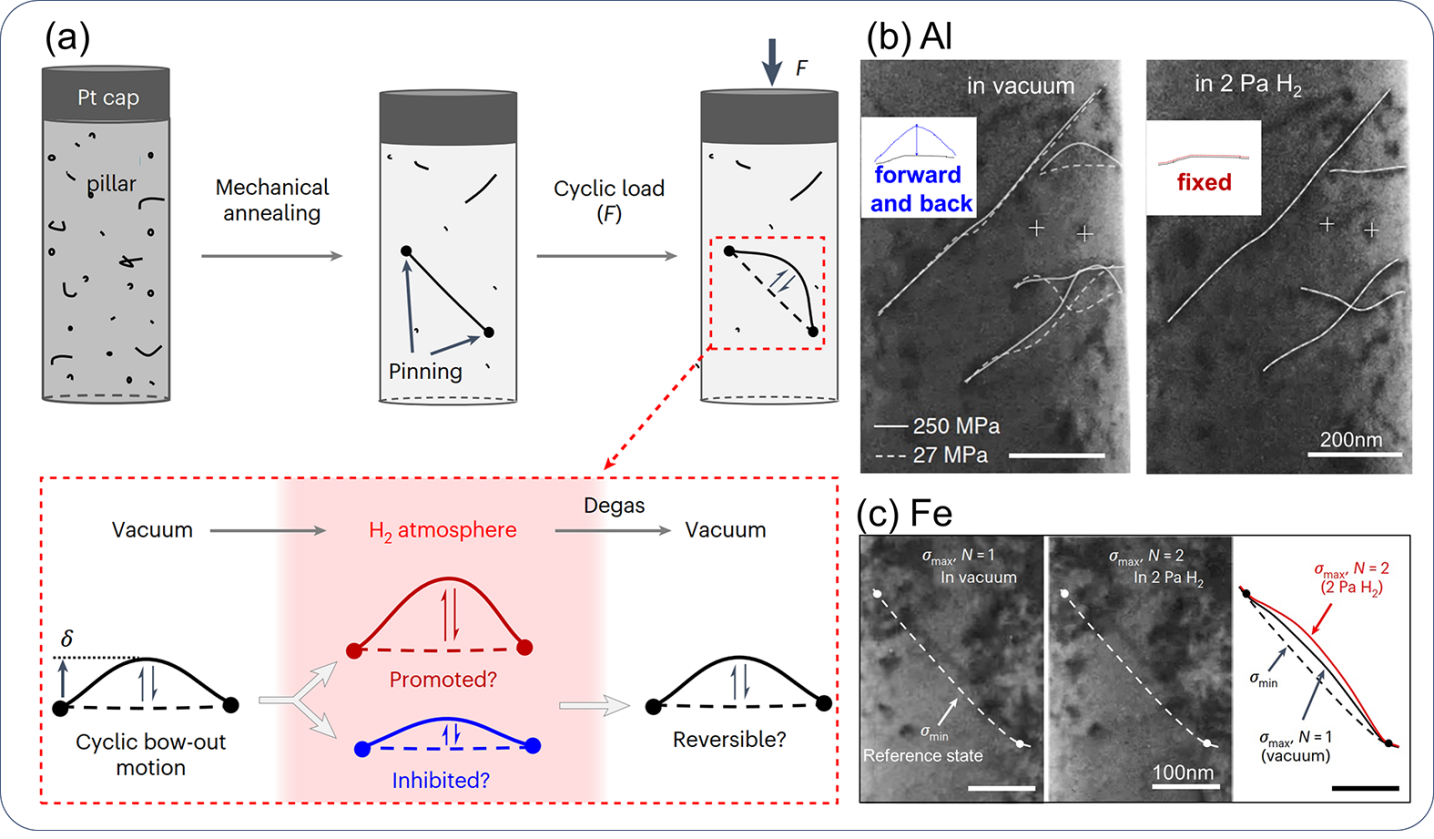
(a) Schematic diagram of the *in situ* ETEM setup used in ref (46) for revealing the effect of H on the motion of single dislocation. Adapted with permission from ref (46). Copyright 2023 Springer Nature. The influence of H on the behavior of dislocation bow-out in (b) pure Al^466^ and (c) pure iron^46^ (white solid/dash lines indicate the position of probed dislocations at corresponding conditions marked in the images). Reprinted with permission from refs (46 and 466). Copyright 2016, 2023 Springer Nature under [CC BY 4.0 DEED] [https://creativecommons.org/licenses/by/4.0/].

However, open questions exist for the previous *in situ* ETEM setup, especially pertaining to whether the acquired results can be affected by some sorts of artifacts (e.g., the bending stresses in the TEM foil introduced from the gas pressure or from the high H fugacity produced by the electron beam dissociating and ionizing the H).^22,46,466^ Indeed, some atomistic calculations have shown rather a decreasing effect of H on dislocation mobility (i.e., a strengthening effect).^23,42,47^ Moreover, it is difficult for previous ETEM setup to quantitatively evaluate the behavior of a single dislocation which can be affected by surrounding dislocations (and their associated stress fields) and by the pinning effect from the thin foil surfaces.^46^ In view of these, Xie et al.^46,466^ have applied a better controlled *in situ* ETEM mechanical testing protocol to revisit H–dislocation interactions at the scale of a single dislocation. The experimental procedure is illustrated in Figure 30a.^46^ In contrast to the conventional setup where a thin foil material containing a number of mobile dislocations was used,^617^ Xie et al.^46,466^ prepared submicron-sized free-standing pillar-typed specimens (Figure 30a). Before the introduction of H, most of the pre-existing dislocations were eliminated by cyclic loading, retaining only a few individually isolated dislocations that are pinned at two ends. The reversible bow-out motion of these dislocations subjected to varying cyclic stresses and atmosphere can be well accessed, which allows to quantitatively reveal the effect of H on single dislocation motion. When applying such experiment in single-crystal Al,^466^ it was observed that all the probed dislocations remained fixed after hydrogenation (i.e., they were locked by H), which was very different from the reversible motion of these dislocations cyclically loaded in vacuum (Figure 30b). Such locking effect was further attributed to the segregation of hydrogenated vacancies to the dislocation core, a more sluggish process than interstitial H diffusion. This finding contradicts with the results from Robertson and Birnbaum^625^ who observed H-enhanced dislocation motion in pure Al. In a more recent work, Huang et al.^46^ used the same setup to study the H–dislocation interaction in pure Fe, from which a completely different conclusion was drawn. In that study, the presence of H was found to enhance rather than suppress the screw dislocation motion in Fe at low H concentrations (Figure 30c), showing ∼27% reduction of the activation stress for the motion of one particular dislocation and ∼64% enhancement of its bow-out distance (in comparison to the behavior in vacuum under the same applied load).

The above brief review on recent *in situ* ETEM studies shows that the H effect is highly dependent on a variety of factors including material, dislocation type, H environment and concentration, vacancy concentration, and mechanical conditions. The enhancing role of H on dislocation motion is unlikely to be a universal rule regardless of intrinsic and extrinsic conditions. The boundary conditions controlling whether H promotes or suppresses dislocation motion and their respective extent need to be further clarified, which would require tremendous experimental efforts. In addition to the study of H–dislocation interactions, *in situ* ETEM has also been used to investigate the H-induced crack propagation behavior.^519,521,617,623,626−628^ The typical experimental procedure involves straining the thin-foil sample till crack propagates, after which H is introduced with either incremental straining or maintaining constant displacement.^617^ In the former case of incremental straining, the crack propagation usually occurs at a high speed which renders the capture and interpretation of the dislocation activity near the crack tip difficult. As such, the acquired knowledge from these studies does not show too much progress in comparison to that based on post-mortem crack analysis. Further, since the crack-tip stress state for the thin-foil sample (near the plane stress condition) differs substantially from that for a bulk material, whether such *in situ* ETEM technique is the most suitable tool for studying H-induced damage evolution remains an open question.

##### 2.3.4.2 *In Situ* SEM-Based Technique

**Figure 31:**
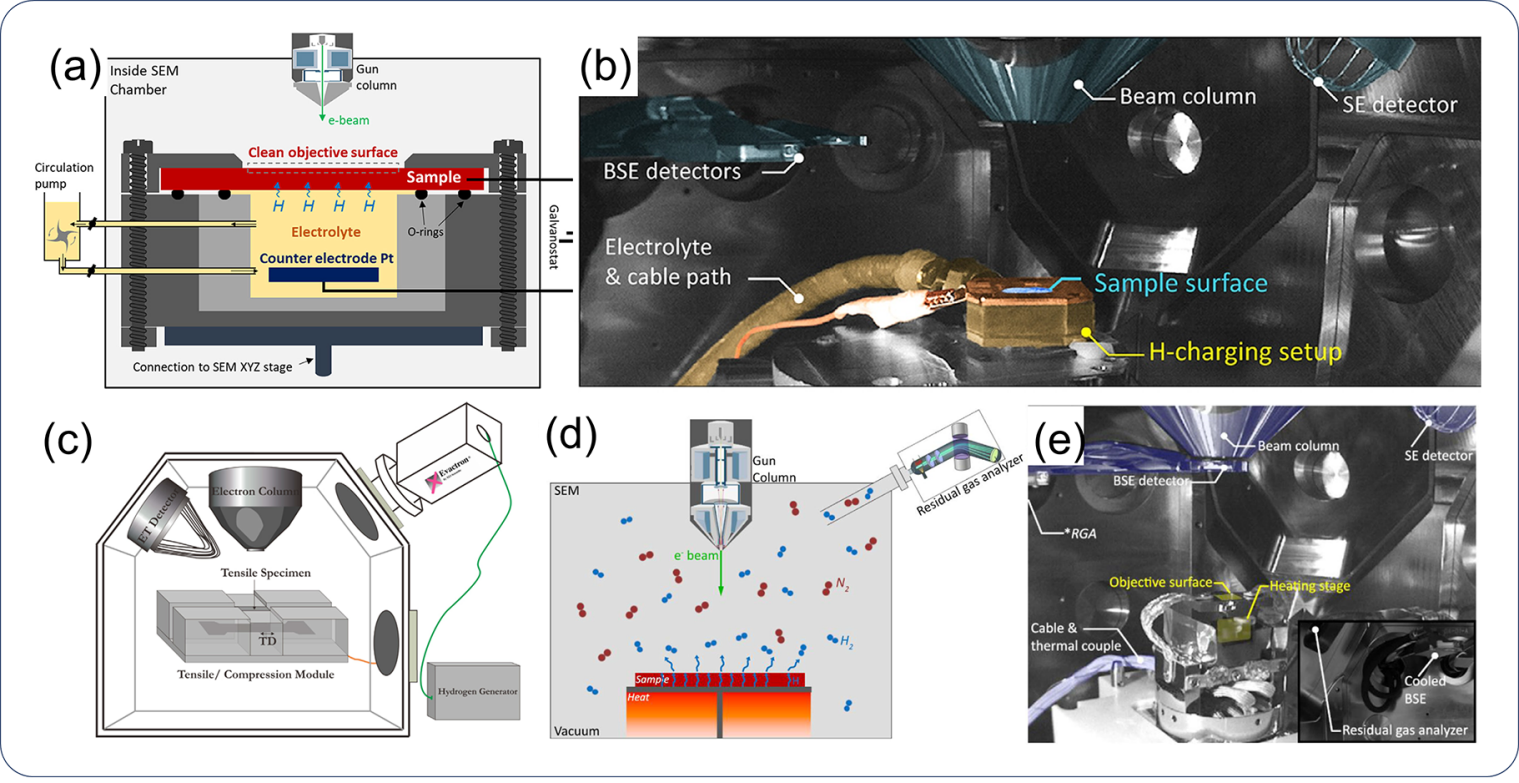
(a) Schematic diagram and (b) infrared image of the SEM-accommodated *in situ* electrochemical H-charging setup. Reprinted with permission from refs (634 and 635). Copyright 2019, 2020 Elsevier. (c) Schematic diagram of the SEM chamber equipped with H-plasma charging setup. (d) Schematic diagram and (e) infrared image of the SEM-TDS setup. Reprinted with permission from ref (639). Copyright 2022 Elsevier.

**Figure 32:**
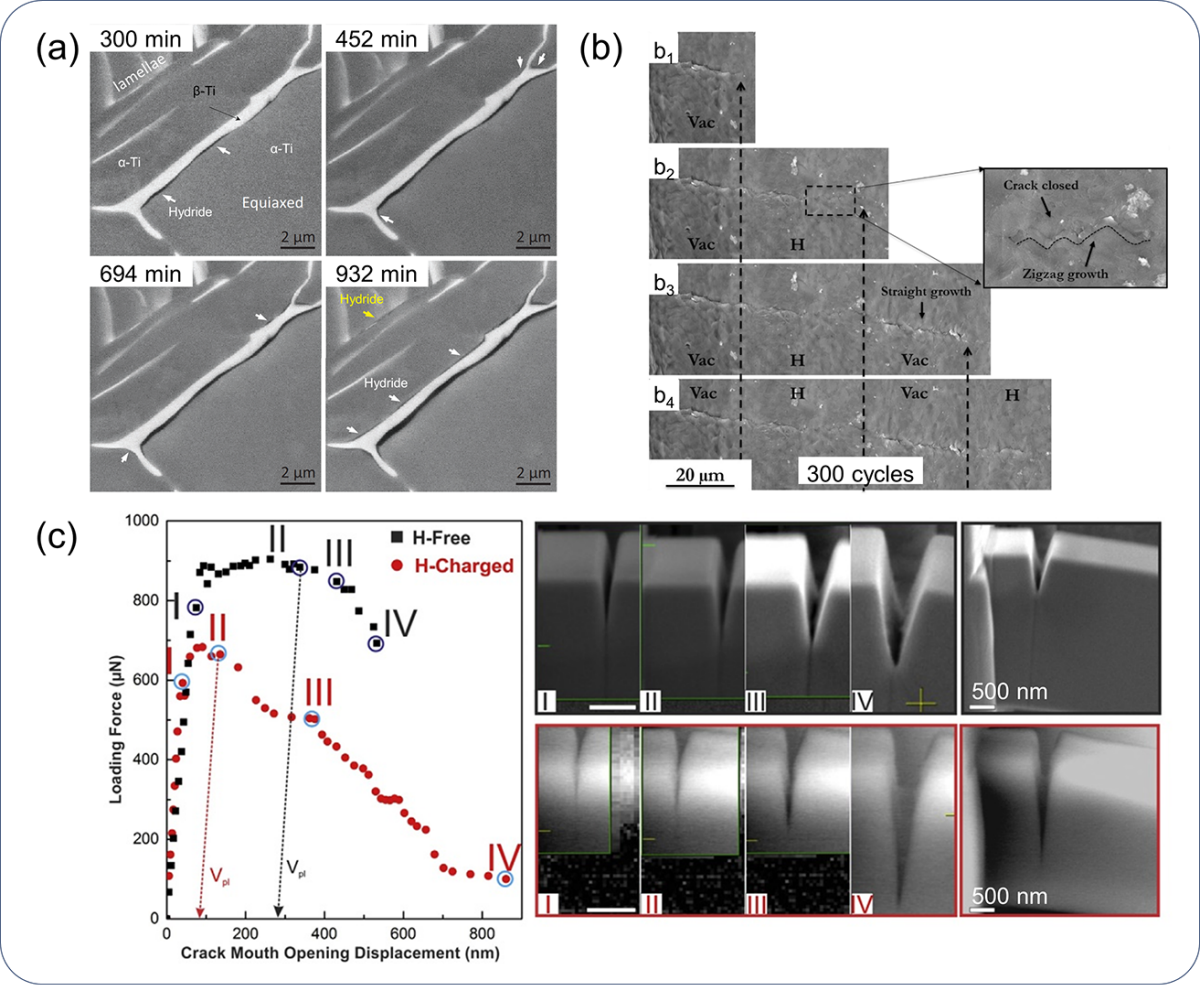
(a) Back-scattered electron (BSE) image of a Ti-6Al-4V alloy subjected to *in situ* electrochemical back-side charging inside the SEM chamber (the charging time was marked on top of each image). Reprinted with permission from ref (635). Copyright 2020 Elsevier. (b) The behavior of FCG of an austenite-ferrite two-phase steel subjected to alternative environment of high vacuum and H-plasma. Reprinted with permission from ref (638). Copyright 2021 Elsevier under [CC BY 4.0 DEED] [https://creativecommons.org/licenses/by/4.0/]. (c) Mechanical data of two FeAl micro-cantilevers bent under vacuum and water vapor (450 Pa), along with SEM micrographs showing characteristic crack propagation stages indicated in the load-crack mouth opening displacement (CMOD) curves. Reprinted with permission from ref (633). Copyright 2018 Elsevier.

In comparison to TEM which is constrained by the limitations of thin-foil samples, SEM-based techniques directly work on bulk materials and provide a larger field of view despite relatively poor spatial resolution. In combination with modern micro- to meso-scale deformation devices, a variety of deformation modes (tensile, compression, bending, etc.) performed on a wide range of sample scales (down to submicron level) can be realized inside a SEM chamber.^630−636^ The difficulty of utilizing such technique to study the HE processes *in situ* lies in the application of a well-controlled H atmosphere. Currently explored *in situ* H charging methods within SEM include (a) electrochemical H charging,^634,635^ (b) H plasma charging,^632,636^ and (c) water vapor reaction.^631,633^ The SEM-accommodated *in situ* electrochemical H charging setup (Figure 31a,b), developed by Kim and Tasan,^634,635^ possesses a similar working principle as that for conventional H charging in air. The main difference is that the H charging cell inside the SEM chamber has to be properly sealed to avoid any leakage of electrolyte as well as molecular H_2_ formed during charging. Unlike conventional electrochemical H charging setup where the whole sample is often immersed in the electrolyte, only one specimen surface can be in contact with the electrolyte for the SEM-accommodated setup and the other probing surface has to be clean, finely polished and directly interact with electron beam for imaging (Figure 31a,b). In this case, H is supplied by its absorption at the source-contacting surface and its continuous diffusion to the probing surface. Kim and Tasan^634,635^ have utilized this setup to study hydride formation and associated embrittlement in a Ti-6Al-4V (in wt. %) alloy. The real-time observation of the hydride formation is shown in Figure 32a, which revealed a prominent hydride formation at the α/β phase boundary in the equiaxed grain regions. These interphase hydrides subsequently act as nucleation sites of nanoscale cracks upon loading, which promoted the premature failure of the material. When combining this *in situ* H charging setup with SEM nanoindentation technique, the temporal evolution of H-induced hardness and modulus change can also be studied.^634^ However, *in situ* observation of H-induced damage evolution (especially through-thickness crack propagation) upon loading would be challenging using this setup due to the risk of electrolyte leakage and thus the contamination of the SEM chamber and the specimen.

In addition to isolated and sealed *in situ* H charging cell, H can also be provided inside environmental scanning electron microscopy (ESEM) through the introduction of H plasma,^632,636^ with the setup schematically shown in Figure 31c. In this setup, an H generator was connected to the working gas inlet of a commercial plasma cleaner attached to the SEM. H atoms and excited H gas molecules were thus produced and pumped into the ESEM chamber.^636^ The remote plasma mode was used to avoid the interaction between plasma and the material. The partial pressure of the plasma phase in this setup can reach up to a few tens of Pa,^637^ which is much lower in comparison to electrochemical cathodic charging (can reach tens of MPa of H fugacity^384^). Nevertheless, such low-pressure H plasma was shown to be sufficient to cause certain degradation of macroscopic mechanical properties (e.g., tensile ductility and FCG resistance) in certain ferritic and austenite-ferrite two-phase steels.^632,636,638^ One example is shown in Figure 32b,^638^ which demonstrated the FCG behavior of an austenite-ferrite medium Mn steel loaded under alternative vacuum and H-plasma condition. The growth of the fatigue crack was obviously faster under H atmosphere. Also, the produced plastic deformation seemed to be weaker when the crack grew under H atmosphere compared with that under vacuum, as shown from the less pronounced topographical change close to the cracking stage under H. It has to be noted that such crack observation was conducted after H-plasma was switched off and the chamber was pumped to high vacuum again. According to Wan et al.,^632,636,638^*in situ* SEM imaging under H-plasma environment in their setup was not possible due to technical limitations of the SEM detector and safety concerns. This means that the SEM probing can only be conducted *in situ* in position but not *in situ* in environment.

The chemical reaction between specific alloy systems (e.g., Al alloys) and water vapor has also been used to provide an H atmosphere inside ESEM. Deng et al.^631,633^ have performed *in situ* micro-cantilever bending test on a single crystalline FeAl alloy within an ESEM filled with 180–450 Pa water vapor. Atomic H was assumed to be produced by the reaction: 2Al + 3H_2_O → Al_2_O_3_ + 6H^+^ + 6e^–^. This setup allows a direct, real-time imaging of the H-induced cracking behavior under well-defined mechanical conditions at the microscale (Figure 32c). It was found that the crack propagation of the FeAl alloy under water vapor atmosphere occurred at a lower stress level, indicating a more facile crack propagation induced by H (Figure 32c). Further post-mortem transmission Kikuchi diffraction (TKD) and TEM analysis revealed a more confined plastic zone with highly localized geometrically necessary dislocation (GND) density in front of the crack tip for the sample loaded under water vapor in comparison to that deformed under vacuum. They attributed this phenomenon to the H-enhanced dislocation nucleation and H-reduced dislocation mobility, factors that suppress further dislocation emission from the crack tip thus promote crack propagation.

The aforementioned previous work mainly utilized *in situ* SEM-based techniques to probe hydride formation or H-induced cracking behavior under a well-defined microstructure and mechanical condition. *In situ* investigation on the H-induced change in dislocation activity should also be possible with the combination of electron channeling contrast imaging (ECCI) technique. Although *in situ* ECCI probing on materials under H exposure has yet to be performed to the best of authors’ knowledge, the work by Koyama et al.^640^ have shed some lights on this direction. In their study, they focused on H pre-charged specimens and have applied *in situ* ECCI to study the dislocation motion during the H desorption process. More missing information for these *in situ* techniques is the often unknown H concentration within the sample. This is particularly problematic for mesoscale samples with a low H diffusivity (e.g., *fcc* materials) throughout which H saturation and homogenization is difficult to reach (if not impossible). Combining SEM with the residual gas analyzer (for the detection of desorbed H) provides some solution to this problem (Figure 31d,e^639^), yet great research effort is still needed to advance such technique to better correlate the detected H signal with the observed local microstructure change and HE behavior.

##### 2.3.4.3 *In Situ* Electrochemical Small-Scale Mechanical Testing

**Figure 33:**
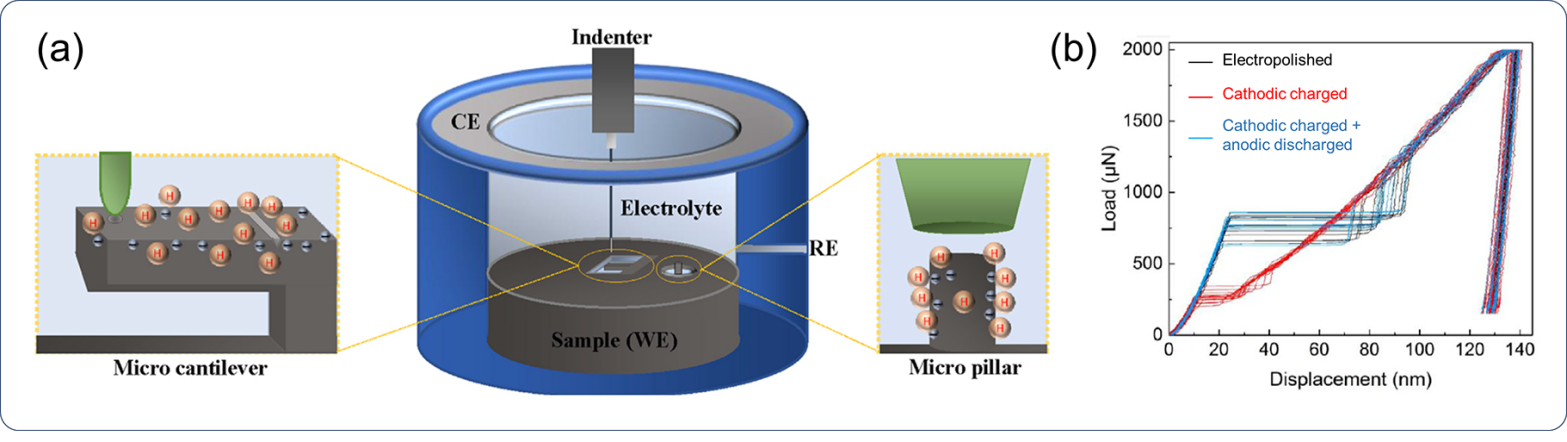
(a) Schematic diagram of the *in situ* electrochemical charging cell used for microscale mechanical testing. (b) Representative nano-indentation load-displacement curves of a Fe-3wt.% Si alloy at different *in situ* electrochemical charging states (reference H-free electropolished condition, cathodic H charging condition, and anodic H discharged condition). Reprinted with permission from ref (643). Copyright 2018 Elsevier.

Since the adoption of *in situ* H-charging cell is generally challenging inside a SEM chamber due to the space and environment restriction, some researchers developed an alternative approach of performing *in situ* small-scale mechanical testing in open, ambient environment with concurrent electrolytic charging.^462,630,637,641,642^. The continuous supply of H by *in situ* H charging is essential for retaining H within the small sample dimension used in micro- and nano-scale mechanical testing. During the electrochemical charging process, the sample can be entirely immersed in the electrolyte such that all the surfaces (including the measurement surface) are charged with H during micromechanical testing (Figure 33a). In this case, the electrolyte and the charging parameters must be carefully selected to minimize any corrosion or contamination of the sample surface that would otherwise cause significant artifacts to the measurement results. Hajilou et al.^643^ have introduced a glycerol-based solution, which was found to be effective in preserving surface integrity at nano-scale during H charging. Using this electrolyte and a nano-indenter device integrated with a miniaturized electrochemical cell, Barnoush and coworkers^462,463,641,643−646^ have performed a number of *in situ* nanoindentation,^462,463,643,645^ micro-pillar compression,^643,647^ and micro-cantilever bending^641,643,644^ tests on different materials including iron-based alloys,^463,641,643,645^ Ni and Ni-based alloys,^374,462,647^ Cu,^462^ and HEAs.^644^ These *in situ* tests, in combination with post-mortem characterization, allow to reveal a more detailed mechanism of H–dislocation interactions and the associated cracking behavior in comparison to traditional experiments on large-scale bulk materials. Take *in situ* nano-indentation tests as an example, an H-induced reduction in pop-in load (Figure 33b) has generally been observed in a variety of materials (e.g., steels, Ni and Ni-based superalloys and CoCrFeMnNi HEA), although the extent of such reduction is dependent on materials, microstructure, and grain orientation.^462^ Such phenomenon can be linked to the earlier onset of plasticity caused by H-promoted homogeneous dislocation nucleation,^648^ which was often explained with the Defactant theory as detailed in Section 2.3.2.5. It is proposed that H, acting as a defactant, can reduce the formation energy of defects (e.g., dislocations) in a manner analogous to the case that surfactants reduce the surface energy of liquids.^35,45,461^

In addition to the setup shown in Figure 33a, the *in situ* charging cell can also be designed in a way that only one sample surface is in contact with the electrolyte and the measurement is conducted on the other surface where H is supplied by through-thickness diffusion (similar to Figure 31a).^649^ Surface contamination can be completely avoided in this case, which ensures better measurement robustness. However, this “back-side” charging setup might not be suitable for metallic materials with a slow H diffusivity (like austenitic steels) due to the prolonged time required for H migration through the whole thickness. When using this setup to perform *in situ* pillar compression and microcantilever bending tests, additional concerns might also arise pertaining to whether H can be retained within the small sample volume with the unavoidable H outgassing from all the free surfaces.

##### 2.3.4.4 *In Situ* Synchrotron High-Energy X-ray Diffraction

The aforementioned *in situ* techniques all focus on localized small regions of a material, statistical information of H-induced microstructure change and H-defect interactions cannot be acquired. This issue can to some extent be solved by *in situ* synchrotron high-energy X-ray diffraction testing performed in H atmosphere. An *in situ* setup was recently developed by Connolly et al.^650^ in Argonne National Laboratory Advanced Photon Source (APS) 1-ID-E beamline. An H gas chamber was mounted on a servo-hydraulic load frame that existed at the beamline. High-pressure H_2_ gas (a few MPa) can be introduced during concurrent loading and X-ray diffraction or imaging, which allows to capture the influence of H on lattice strain fields, dislocation population, material phases, and crack tip morphology in a statistical manner.^650,651^ Connolly et al.^651^ used this setup to study the mechanisms of H-induced FCG in a AISI 4130 steel. They observed an enhancement of the elastic strain field near the fatigue crack grown in H_2_ compared with that in air, which indicated an increased effective stress intensity factor due to the presence of H. Such observation is consistent with the HEDE mechanism. Further, they found a reduced dislocation density at near-crack regions for the sample loaded in H_2_. This behavior was attributed to the HELP mechanism in terms of the H-facilitated movement of dislocations from grain interiors to GBs, which subsequently resulted in IG fracture via the HEDE mechanism. An H-promoted TG crack growth due to HEDE and the H-induced IG cracking due to HELP facilitated HEDE were thus concluded as the key HE mechanisms for this material.

##### 2.3.4.5 Summary and Key Challenges in Experimental Approaches

**Figure 34:**
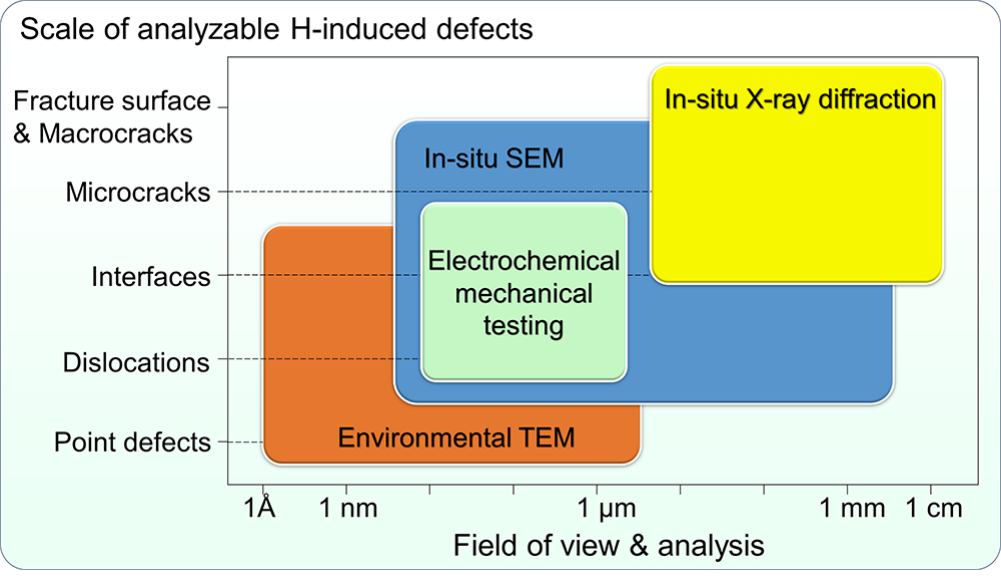
Comparison of *in situ* characterization techniques for HE study in terms of their field of view and the scale of analyzable H-induced defects. Redrawn with permission based on ref (634). Copyright 2019 Elsevier.

In summary, the development and application of various *in situ* characterization techniques under H atmosphere offers more direct observation on H-induced microstructure change and defect evolution, which advances our fundamental understanding of HE mechanisms. Naturally, the aforementioned different experimental techniques have certain limitations on the field of view and spatial and temporal resolution, which restricts the scale of microstructural features that can be analyzed. The capability and comparison of these techniques in terms of their field of view and the scale of analyzable H-induced defects are roughly summarized in Figure 34. A common issue in these *in situ* methods is the accurate control of the H source and quantification of H concertation within the sample. It should also be noted that the acquired knowledge from these small-scale characterization methods in only applicable for very specific microstructure, environmental and mechanical conditions. Caution must be taken when correlating such knowledge to the H-induced degradation of macroscopic properties that is essentially a collective result of H-induced defect and damage events across many different scales and boundary conditions.

#### 2.3.5 Exploring HE Mechanisms by Atomistic Simulations

Considering the current limitations in experimental techniques to detect atomic H and elucidate the H-participated microstructure evolution,^21,26^ atomistic simulations have emerged as a powerful tool for investigating the interactions between H and metals at the atomic/nanoscale. Based on quantum mechanics principles, these simulations can provide insights into the atomic-level mechanisms that govern HE, without requiring a priori knowledge of material properties.

DFT calculations and empirical interatomic potential-based MD simulations are among the most used methods for investigating atomic-scale HE. DFT allows for the calculation of electronic structure and properties of materials critical to understanding H–metal interactions, providing valuable information on the binding energy of H to specific microstructures, such as vacancies, dislocations, and GB as elaborated in Section 2.2.4. Importantly, this information also helps investigate their formation and stability in the presence of H, understand microstructure-correlated HE mechanisms, and develop up-scale modeling tools. However, DFT calculations are usually restricted to thousands of atoms due to computational capacity limitations, which is insufficient to elucidate certain key scenarios in HE, such as dislocation–obstacle interactions and plasticity activity near the crack tip. Hence, empirical interatomic potentials, developed by fitting potential energy functions from DFT calculations and experimental data, are used to study these collective behaviors instead. These potential-based simulations are faster and less computationally expensive than first-principles methods, capable of simulating millions or even billions of atoms. Depending on whether kinetics is introduced, they can be classified into molecular statics (MS) and MD. MS works like an extension of DFT to calculate the energy of minimized H-segregated microstructure at a larger scale. In contrast, MD calculates the trajectory of particles by solving Newton’s law, helping to demonstrate the H-participated microstructure evolution as a function of time. However, both of them sacrifice some accuracy when describing the entire physics of the system, and the simulation performance is highly dependent on the choice of interatomic potential. Critical opinions exist about the incoherent results obtained from simulations using different potentials. Despite those limitations, atomistic simulations combined with kinetic or thermodynamic models can provide a consistent map of multiscale HE mechanisms.

##### 2.3.5.1 H Reduced Cohesive Energy

Troiano^652^ postulated that the incorporation of 1s electron of H atom into the unfilled d-band of transition metals triggers a repulsive force. Oriani^653^ subsequently advanced a mechanistic model for the crack propagation velocity by considering the reduced cohesive force in the presence of H. Essentially, HEDE involves a process of electron transfer and cohesive energy reduction that results in the decrease of cohesive strength. This quantum-level electron behavior is hard to be directly proved by experiments, thus atomistic simulation has been a perfect tool to verify or even quantitatively develop the HEDE mechanism.

In line with the early surface energy model,^654^ Rice and Wang^655^ suggested that the segregation of impurity at the interface alters the ideal work of interfacial separation, i.e., the Griffith fracture energy required to separate the interface into two free surfaces. They further compared the experimental data on free surface (FS) and GB adsorption for carbon, phosphorus, tin, antimony, and sulfur segregation in iron with the data from IG fracture experiments. The most striking result is that the potency of a segregating solute in reducing the Griffith energy can be linearly determined by the difference between binding energies for that solute at the GB, *E*_b_^GB^ and at the free surface, *E*_b_^FS^. This highlights that the energy difference Δ*E* = *E*_b_^GB^ – *E*_b_^FS^ can work as a measure of embrittlement potency. A solute with a positive value of Δ*E* (i.e., *E*_b_^GB^ is smaller in magnitude) will work as a potential embrittler, while a solute with a negative Δ*E* can enhance GB cohesion.

In the 1990s, inspired by the Rice-Wang model, Freeman and co-workers^352,353,656,657^ were the pioneers to use DFT to calculate energy difference Δ*E* and predict the embrittlement potency at GBs in the presence of segregated impurities such as boron, carbon, nitrogen, phosphorus, and H. For example, Geng et al.^353^ first calculated the embrittlement potency of H, boron, and phosphorus on Σ5 (210)[100] nickel GB. The H binding energy difference between Σ5 GB and (210) free surface, Δ*E*, has a positive value of 0.27 eV, indicating that it’s a strong embritter, while boron with a negative value works as a GB cohesion enhancer. Besides, they also found the energy difference Δ*E* mainly originated from chemical contributions rather than mechanical contributions, i.e., H–metal interaction is dominated by electron transfer between H and metal instead of changes in the host–host interaction induced by the H. Using DFT, Zhong et al.^352^ further calculated the H-induced cohesion reduction at Σ3 (111)[1–10] iron GB. Even the calculated binding energies vary according to the different approximating methods in DFT, the Δ*E* are well located in the range of 0.26–0.33 eV, indicating H as a strong embrittler. Simply put, H-free surface interaction is stronger than H-GB interaction in nickel and iron.

**Figure 35:**
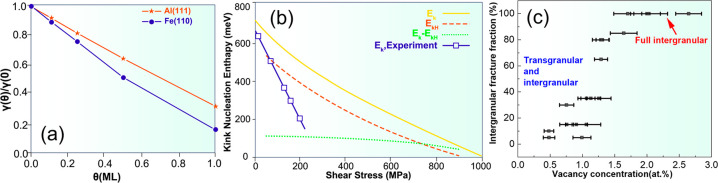
(a) Normalized facture energies (γ_θ_)/(0) for H-covered Al(111) and Fe(110) surfaces as a function of H coverage (γ_θ_). Redrawn with permission from ref (324). Copyright 2004 Elsevier. (b) Kink nucleation enthalpy with H (*E*_KH_) and without H (*E*_K_) for the screw dislocation in *bcc* iron as a function of shear stress, with a low value indicating enhanced dislocation mobility and softening effect. Redrawn with permission from ref (346). Copyright 2013 Elsevier. (c) IG fracture fraction as a function of vacancy concentration at GBs. The fully IG fracture happens as critical vacancy concentration (1.7% for Σ5 GB) is reached, i.e., all the samples are expected to fail by IG fracture when such vacancy concentration is reached. Redrawn with permission from ref (659). Copyright 2022 Elsevier under [CC BY 4.0 DEED] [https://creativecommons.org/licenses/by/4.0/].

The aforementioned positive or negative value of ΔE could only help to identify the embrittling and strengthening effect of solutes of different elements. To make quantitative prediction of embrittlement, it is necessary to obtain H concentration-dependent cohesion reduction. By envisioning a scenario where H could quickly segregate to the opening crack surface and reach equilibrium, Jiang et al.^324^ calculated the reduced normalized Griffith energy for iron as a function H coverage *θ_H_* which decreases almost linearly with the increase of H coverage, dropping by 47% at one-half monolayer and by 81% at full monolayer coverage (as shown in Figure 35a). Those DFT data have been taken as key inputs in the continuum-scale cohesive zone modeling to quantify the cohesion reduction in bulk iron as a function of H concentration. By assuming the two scenarios in the Rice-Wang model: fast fracture where H is immobile and its coverage stays constant, and slow fracture with mobile H corresponding to Jiang’s^324^ setup, Yamaguchi et al.^658^ found the cohesion reduction at a Fe Σ3(111) symmetrical tilt GB could be 70–80% during slow fracture in contrast to only 10–20% during fast fracture. Essentially, the difference in cohesion reduction between fast and slow fractures is caused by the dramatically different H coverage that participates in the decohesion and is left on the final separated surfaces. It is worth emphasizing that the reduction in cohesion due to H concentration is significantly influenced not only by H occupying the surface but also by H atoms present beneath the surface. In particular, Tehranchi and Curtin^349^ demonstrated that cleavage fracture along (111) planes in Ni originates from the establishment of three planar layers involving interstitial H occupancy positioned at a clearly defined crack tip. As cleavage progresses, sub-surface H atoms within the upper and lower layers rapidly diffuse toward the fracture surface, leading to a decrease in fracture energy. Consequently, this process facilitates the occurrence of brittle fracture.

Besides the H coverage at the surface, the GB type and local geometrical arrangement are other factors that influence the reduction of cohesion. Du et al.^362^ calculated the cohesion reduction to be 11%, 29%, and 33%, respectively, for *bcc* Σ5(310), *fcc* Σ11(113), and *bcc* Σ3(112) GBs in iron with the same H coverage, varying due to their intrinsically different structures. However, DFT calculations are still limited to several fixed GB types, empirical potential-based simulations are therefore utilized to explore the cohesion reduction at a wider variety of GBs. For example, Wang et al.^364^ used MS combined with an EAM potential^479^ to calculate the reduced Griffith energy for a number of iron GBs considering multiple trapping sites. They found the segregation energy in the Rice-Wang model is a macroscopic average based on the Langmuir-McLean law, while there is no unique value at the microscale as the energy is dependent on the specific trapping site. They further concluded that the cohesion reduction predicted by the model is insufficient to account for the experimentally observed level, microstructure evolution or plasticity must also play a role in the H-induced IG fracture. Using MS and an H-Ni potential,^360^ Tehranchi et al.^349^ elucidated the decohesion energy at several nickel GBs and GB cracks. The deformation behavior at the GBs is, however, generally complex and not as simple as cleavage or dislocation emission at a sharp crack tip, which is not unexpected due to the complexity of the GB structures.

Besides at GBs and in the bulk, H promoted decohesion can also take place at precipitate interfaces and phase boundaries. Using DFT, Geng et al.^660^ found that H significantly enhances decohesion between (110) planes both inside the martensite and along the martensite/ferrite interface, with the former being more significant than the latter. This can explain the H-initiated crack observed in the martensite region.

MD or semi-MD method could also provide deep insights into HEDE-related processes. Using MD combined with kinetic analysis, Song et al. observed an atomic H-controlled ductile to brittle transition in both nickel^42^ and iron.^47^ Driven by the stress field, H accumulation around a microcrack tip prevents crack-tip dislocation emission or absorption, and thus suppresses crack-tip blunting, which leads to brittle crack propagation. They also provided mechanism-based predictions of embrittlement thresholds over a range of environmental factors in comparison with experiments. More recently, Zhou et al.^661^ found that H atoms increase the unstable stacking energies for a variety of alloys, while significant reduction in fracture energies was revealed. This interesting phenomenon gives rise to a competition between dislocation emission and cleavage at a crack tip, ultimately triggering the ductile-to-brittle transition in the examined alloys.

##### 2.3.5.2 H–Dislocation Interactions

Due to the complexity of the material system and experimental setup, there exist contradictory conclusions on whether H facilitates dislocation mobility.^46,466,629^ By simplifying the system, atomistic simulations help to obtain some fundamental insights into the H–dislocation interactions. Many atomistic simulations support the enhanced mobility of dislocation in the presence of H. Combining DFT and the Peierls-Nabarro model, Lu et al.^452^ first reported a strong binding of H atoms to screw, edge, and mixed dislocation cores in Al which inhibits dislocation cross-slip and develops slip planarity. The reduction of Peierls stress of dislocations (i.e., the minimum shear stress required to move a single dislocation in a perfect crystal) by more than an order of magnitude in the presence of H strongly suggests enhanced mobility of dislocations in the presence of H. But different from the original HELP mechanism^41^ where enhanced mobility is caused by H distribution in the elastic field around a dislocation, Lu et al.^452^ attributed this mobility modification mainly to the H trapped at the dislocation core. Through MS combined with EAM potential^478,479^ and NEB method,^662^ Taketomi et al.^345^ and Wang et al.^663^ reported similar H–dislocation core interaction and reduced Peierls stress for edge and screw dislocation in *bcc* iron. However, Taketomi et al.^664^ further reported that dislocation mobility in the presence of H depends on the stress conditions, increasing at a lower applied stress and decreasing at a higher stress. Itakura et al.^346^ used DFT to calculate the H binding energy at various positions of a screw dislocation in *bcc* iron. The results were incorporated into a line tension model of a curved dislocation line to elucidate the effect of H on the dislocation migration process. Both the softening and hardening effect of H, caused by the reduction of kink nucleation enthalpy and kink trapping, respectively, were elucidated as shown in Figure 35b. Using EAM potential and NEB method, Wen et al.^665^ pointed out that both H-induced softening and hardening could be caused by H interaction with kink pairs depending on specific microstructure configuration and temperature. Using DFT and EAM methods, Pezold et al.^666^ further pointed out that the local H concentration at the dislocation core highly depends on the H–H interaction. Even a weak attractive interaction between interstitial H atoms in the host matrix could lead to the formation of local hydrides along the dislocation line, which induced a short-range shielding effect along the slip plane correlated with a reduced dislocation separation at dislocation pile-up tips.

However, different or even opposite modeling results have been reported, particularly when considering time-dependent kinetic processes, an aspect noticeably absent in the aforementioned studies that were inclined towards static analyses rooted in energy considerations. This dynamic factor, however, is taken into account in MD simulations. For example, by conducting a series of MD simulations directly recording the dislocation gliding process, Song et al.^42^ revealed that H Cottrell atmosphere provides no shielding effect between edge dislocations in iron but rather suppresses their motion consistent with solute drag theory. Furthermore, once dislocation motion stops and a pile-up is established, the H Cottrell atmosphere do not affect the equilibrium spacing of dislocations in the pile-up; thus, the H atmosphere provides no “shielding” of dislocation–dislocation interactions. Using MD, Bhatia at al.^667^ also reported an enhanced Peierls stress for edge dislocation in the presence of H for *bcc* iron, which highlights the pinning effect of H. Combining *in situ* TEM nano-compression tests and MD simulation in aluminum, Xie et al.^466^ revealed that both H and vacancy–H (VH) clusters could apply a pinning effect on the edge dislocation motion, with the latter being more significant than the former.

Not all MD studies challenge the concept of enhanced dislocation mobility. Huang et al.^46^ combined in situ TEM nano-compression tests, DFT and MD simulations to reveal that H can increase the mobility of screw dislocation in *bcc* iron by lowering the critical shear stress required. Contrasting with earlier findings,^466^ they further suggested that the interaction between H and dislocations, whether enhancing or decreasing mobility, varies with the material, dislocation type, and the concentrations of H and vacancies. In their MD study, Tehranchi et al.^43^ discovered that VH clusters in nickel facilitate the movement of dislocations, consistent with the HELP mechanism, in contrast to the pinning effect identified by Xie et al.^466^ in Al.

The discrepancy between these MD studies is likely due to different model treatments. In Xie et al.’s work,^466^ VH clusters were directly inserted into the dislocation core, creating jogs in the dislocation. However, in Tehranchi et al.’s study,^43^ VH clusters were distributed randomly away from the dislocation core, and the core region was purposefully left VH-free to avoid “unusual interactions with the dislocation core” and the formation of jogs, which act as strengthening obstacles. This underscores how the outcome of an MD simulation is highly dependent on the model’s treatment. Other factors, such as the selection of interatomic potentials and the application of boundary conditions, also significantly influence the results.

In addition to the aforementioned studies that directly comment on dislocation mobility, several atomistic studies have provided alternatives to account for the dislocation mobility from different views. Through Grand Canonical Monte Carlo (GCMC) simulations, Yu et al.^668^ found H brings minor effect on the stress field of either the edge or screw dislocation, but increases the core radii and decreases the core energy of dislocations, which is the only factor leading to the reduction of dislocation line energy by H. Based on those results, they claimed that the experimentally enhanced homogeneous dislocation nucleation^648^ is due to the effect of H on the dislocation core. Those results agree well with the Defactant concept,^35^ where H lowers the defect formation energy, including vacancy, GB, and dislocation. Through DFT calculations, Li et al.^669^ found that H could induce dislocation core reconstruction in *bcc* tungsten: At low concentrations of H, dislocation maintains the intrinsic easy-core structure and H atoms enhance dislocation motion; while at high concentrations, dislocation transforms into a hard-core and H hinders the dislocation motion. With MD, Ding et al.^670^ found that H could have a dual role in the mobility of an edge dislocation array-type GB. In the low temperature and high loading rate regime, where H diffusion is substantially slower than dislocation array motion, GB breaks away from the H atmosphere and transforms into a new stable phase with highly enhanced mobility. In the reverse regime, H atoms move along with the dislocation array, exerting a drag force on GB and decreasing its mobility. Those findings indicated the importance of considering the specific microstructures when modeling the H–dislocation interactions.

Besides focusing on the behaviors of single or several dislocations, atomistic simulation also provides information on the collective behavior of dislocations,^671^ especially at the crack tip. For example, Matsumoto et al.^672^ applied MD to simulate the mode I crack propagation in α-Fe single crystal with and without H under different conditions, they found that in the cases without dislocation emission, H has a negligible effect on crack propagation behavior, while in cases with dislocation emissions, H can transform the originally crack blunting into crack propagation along slip planes. These results are in line with the AIDE mechanism,^436^ highlighting the important role of dislocation emission in causing HE. Using MS, Taketomi^465^ found that H decreases the SFE of α-Fe, resulting in an enhancement of dislocation emission under mode II. They concluded that H trapped at the dislocation core reduces the energy barrier for dislocation motion and subsequent separation is connected among pile-up dislocations. This can also be viewed as supporting evidence for the AIDE mechanism.

The enhanced plasticity could also happen in the vicinity of GB, facilitating IG fracture as observed in experiments.^36,38^ Using MD, Wan et al.^673^ found that during deformation dislocation impingement and emission on the H-saturated GB can transform the GB into an activated state with a more disordered atomistic structure, and introduce a local stress concentration responsible for the subsequent decohesion in *bcc* iron. They claimed this dislocation-GB reaction as a key process in controlling IG facture and HE. Li et al.^674^ reported that H could transfer the dislocation-GB interactions including transmission, nucleation and reflection into dislocation absorption in nickel. Ding et al.^675^ further revealed a nanoscale H-induced TG to IG fracture transition in nickel, where GB-dislocation interaction facilitates vacancy generation and causes nanovoid nucleation at GB. Chen et al.^676^ found that H-inhibited GB migration and H-enhanced GB dislocation emission together lead to cleavage-like crack propagation along the GB.

##### 2.3.5.3 Role of Nanovacancies in HE

Details about the HESIV mechanism have been elaborated in Section 2.3.2. Atomistic simulations have contributed to the understanding of this mechanism in two aspects: investigating the stability of VHs and exploring their impact on mechanical response, particularly the fracture mechanism associated with nanovoid formation.

As elucidated in Section 2.2.4, a number of DFT calculations have been conducted to determine the energetics of H–vacancy interaction. These data are helpful for assessing the stability and distribution of the vacancies, especially when coupled with TDS or PAS data. By comparing the DFT-calculated energies of different VH configurations VHn with experimental data, Tateyama et al.^339^ found that VH_2_ is the major complex at an ambient H_2_ pressure. They suggested that H facilitates the formation of line-shaped and tabular vacancy clusters without the improbable accumulation. These anisotropic clusters can be closely associated with the fracture planes observed in H-charged steel. Using DFT, Nazarov et al.^340^ studied H–vacancy interactions in *fcc* iron and found that a monovacancy can trap up to six H atoms. Combined with a thermodynamics model, they were able to determine the equilibrium vacancy concentration as a function of H chemical potential and temperature. H can dramatically increase the vacancy concentration by more than seven orders of magnitude and thus result in the formation of superabundant vacancies as observed in experiments. Tanguy et al.^341^ further studied H interaction with monovacancy or divacancy in nickel by DFT, and obtained a binding energy of −0.27 eV and −0.41 eV for monovacancy and divacancy, respectively, which agreed well with the two characteristic peaks in TDS data. Combining with Monte Carlo simulations, they obtained the VHn cluster distribution as a function of lattice H concentration. The stability domain of VH_6_ clusters was found to overlap with the experimental conditions for embrittlement.

Experimental evidence^449^ has shown that vacancy accumulation in the vicinity of GBs is critical to H-induced GB fracture, and numerous efforts have devoted to understanding the vacancy generation at GBs. Considering interactions between H, vacancies, and Σ3 GB in α-Fe, Momida et al.^677^ searched for the most deleterious defect states by evaluating their influence on tensile strength under static tensile strain using DFT. It was shown that H and vacancies prefer to accumulate near GBs, thereby decreasing the strength of GBs. Using DFT, Zhou et al.^358^ investigated the effects of alloying elements on vacancies and VH clusters at coherent twin boundaries in nickel alloys. Although the alloying elements did not favor H accumulation, it can considerably reduce the formation energies of vacancies and VH clusters at coherent twin boundaries, which may facilitate crack initiation. Opposite results have also been reported. Using DFT and thermodynamics thoery, Polfus et al.^678^ found that vacancy cluster are stabilized by H at GBs in Pd, and the equilibrium vacancy concentrations are enhanced by several orders of magnitude. However, coalescence of vacancies into nanovoids was found to be thermodynamically unfavorable, meaning vacancy clusters do not directly cause the formation of nanovoids.

In most of the aforementioned DFT studies, it was proven that H stabilizes vacancies by forming VHs and leads to the formation of superabundant vacancy clusters. However, little is known about whether and how those vacancy clusters can cause nanovoid nucleation and actually promote fracture. As per the HESIV mechanism, straining is a premise for the formation of superabundant vacancies in the presence of H, the complex interactions among H, vacancy, dislocation, and GB should be considered to probe H- and vacancy-induced fracture mechanism. MD simulations with a time-dependent kinetic processes provide the perfect tool for such an investigation. Li et al.^679^ revealed that, unlike a lattice vacancy, a VH is not absorbed by dislocations sweeping through the lattice in *bcc* iron and has a lower probability to be annihilated by various lattice sinks. Consequently, an extremely high concentrations of VH can be achieved during plastic deformation in the presence of H. Under such high concentrations, these VHs prefer to aggregate by absorbing additional vacancies and act as nuclei for nanovoids. These findings help to link VHs at the atomic scale to macroscopic failure by nanovoid coalescence in the presence of H. Using GCMC and MD, Ding et al.^659^ proposed a vacancy-controlled GB nanovoiding mechanism to account for the TG to IG fracture transition in nickel. H was found to enhance strain-induced vacancy generation by up to ten times. This leads to the superabundant vacancy stockpiling at the GB, which further results in the formation of nanovoids at the GB and eventually causes IG fracture. Importantly, an “S”-shaped quantitative correlation between the proportion of IG fracture, i.e., the degree of embrittlement, and vacancy concentration, was derived to quantitatively describe the TG to IG fracture transition. A characteristic vacancy concentration was highlighted, beyond which the fracture mode will be completely IG (as shown in Figure 35c). This may provide clue for the “S” pattern observed in laboratory tensile tests in H induced degradation of fracture property. More about the pattern at a macroscopic scale is presented in Section 2.4.2.

##### 2.3.5.4 Key Challenges and Limitations

Atomistic simulation is undoubtedly a valuable tool for attaining a deep understanding of HE in metals and is an essential complement to experimental studies. Its utility lies in interpreting experimental observations and providing theoretical insights into the fundamental mechanisms of HE, offering details beyond the scope of current experimental techniques.

However, the effective use of atomistic simulation requires awareness of its limitations. DFT accurately describes the interaction energy of H with microstructural defects, making it a crucial benchmark tool for MD simulations. Yet, DFT’s applicability is limited to small systems of a few hundred atoms due to its high computational cost. This limitation renders DFT unsuitable for simulating H in complex and realistic material systems or for kinetic simulations like H diffusion.

The use of an empirical potential-based approach in atomistic simulations allows for the simulation of large material systems encompassing over a billion atoms and the incorporation of time-dependent processes, offering a more comprehensive understanding of HE mechanisms. Most atomistic simulations are based on potentials developed within the framework of the EAM, with parameters tailored to match experimentally measured properties and/or first-principles calculations. The reliability of these simulations heavily depends on the chosen potentials. However, existing EAM potentials are not without flaws, and only a few can accurately represent a wide range of material properties in the presence of H. Tehranchi and Curtin’s review paper^23^ highlighted the complexity in adding another element as a solute to a metal. Accurate interactions between metal–solute, solute–solute, and H–solute, in addition to metal–metal and metal–H interactions, are required. This complexity makes it extremely challenging to identify suitable interatomic potentials for alloys with H. Consequently, current MD simulations of H–solute/vacancy–dislocation interactions are more qualitative and conceptual, rather than quantitative and definitive.

As a result, when initiating a new study on H-related phenomena, it is crucial to determine the availability of a suitable potential tailored to the specific case. This is a significant challenge in the field. Machine learning (ML) offers a promising solution to this bottleneck by developing material-specific and reliable potentials, as to be discussed in Section 3.5.

#### 2.3.6 Mechanistic Modeling of HE

Except for atomistic scale modeling, mechanistic simulation is also an indispensable tool for the study of HE. Meso-scale discrete dislocation plasticity approach provides a powerful tool to interpret microscopic experimental observations. Continuum models are utilized to interpret the cracking phenomena and reproduce strength and fracture toughness tests at laboratory scale. These simulations are fundamentally different from the atomistic investigations reviewed above, in that they are all based on an existing mechanistic model and on the HE mechanisms. In this section, we distinguish the numerical methods used for studying the HE mechanisms and the models applied for predicting the macroscopic property degradation as a result of HE. We present a review of the numerical simulation approaches based on each of the existing HE mechanisms and discuss their roles in furthering the understanding of HE. Application of these approaches in predictive modeling is discussed in Section 3.4.

##### 2.3.6.1 HEDE Mechanism-Based Modeling

A critical review on the fundamental HE mechanisms was presented in Section 2.3.2. It was concluded that the majority of these fundamental mechanisms are associated with dislocations activities and are unable to directly predict the crack initiation and propagation, with one exception, the HEDE mechanism. HEDE has a clear link to crack formation. Simply, the critical energy release rate related to the creation of new surfaces, i.e., fracture toughness, is reduced in the presence of H. Thus, HE can be simulated with a fracture mechanics approach by describing the fracture toughness as a decreasing function of H concentration. Both the so-called cohesive zone model and phase field model have been developed for HEDE-based numerical simulation.

*H Informed Cohesive Zone Model*. In the cohesive zone model, a material interface is modelled as a layer of cohesive elements that widen and eventually break under loading. In this way, interfacial separation is explicitly simulated. The behavior of a cohesive element is governed by the so-called traction-separation law,^680^ which specifies the cohesive stress, σ, developed in the cohesive element as a function of the separation, δ, between the two surfaces of the element. Upon loading, δ increases, while the cohesive stress σ first increases then drops after reaching a critical value σ_C_. The element is deemed to have failed completely when a critical distance or separation, δ_C_, is reached, and two free surfaces are created at failure, since the cohesive stress σ has turned zero. In this way, crack initiation is simulated. The area under the traction-separation curve is termed the cohesive energy and is related to the critical energy release rate of fracture, i.e., fracture toughness. The influence of H is implemented by decreasing the area under this curve. In practice, this is usually achieved by setting the critical distance δ_C_ constant while decreasing the critical stress σ_C_ as a function of H concentration.

In 2004, Jiang and Carter^324^ conducted DFT calculations on iron and aluminium and calibrated the relation between the cohesive energy and H coverage. In the same year, Serebrinsky et al.^98^ converted H coverage to bulk concentration and applied the atomistically calibrated relation in a cohesive zone model. In 2008, Olden et al.^681^ adopted the same relation and conducted H informed cohesive zone simulation in an austenitic steel. They considered trapped H concentration as a function of plastic strain. With this or other similar modeling frameworks, a great amount of numerical simulation has been conducted. A comprehensive review was conducted by Jemblie et al.^25^ in this regard.

Obviously, the accuracy of H informed cohesive zone simulation is highly dependent on the degradation function of the cohesive energy with respect to H concentration. Most of cohesive zone studies so far have adopted the relation calibrated by Jiang and Carter,^324^ however, it is questionable whether a degradation function calibrated at the atomistic scale is directly transferable to a continuum-level simulation; it is also questionable if the relation calibrated for pure iron is applicable to a wide range of steels. In addition, the direct translation of a surface coverage-based function to the bulk concentration-based simulation is a rough estimation. Among the few studies that did not apply this function, Alvaro et al.^682^ calibrated an H degradation function based on first principles calculations and applied it in cohesive zone simulation. Scheider et al.^683^ adopted a trapezoidal traction–separation law and a linear H degradation function on the cohesive strength; the degradation parameter was determined by fitting experimental data. Raykar et al.^684^ assumed a quadratic H degradation function and determined the degradation parameters by iterative fitting of experimental data. Yu et al.^680^ proposed an approach to experimentally calibrate the H degradation function from tensile tests, which showed good accuracy in simulating delayed fracture experiments.

*H Informed Phase Field Model*. With the cohesive zone model, fracture can only be simulated along predefined crack paths inserted with cohesive elements. Therefore, this method is not capable of modeling the arbitrary nature of crack propagation. The shortcoming can be overcome with the phase field model. This model is based on energy minimization, i.e., the minimization of a free energy functional composed of the stored elastic bulk energy plus the fracture energy, and no assumption of predefined cracks is needed such that crack initiation, growth, and deviation can be automatically simulated.^685^

In modeling of fracture, the phase field resembles a damage field, with the value of ϕ = 0 representing intact regions and of ϕ = 1 representing fully cracked material points.^686^ A characteristic length-scale parameter *l* is introduced which relates to the size of fracture process zone, as *l* → 0, the Griffith’s fracture theory is retrieved. In practice, *l* usually takes a nonzero value and is related to the characteristic length of a material. In this respect, the constitutive behavior of an element in the phase field model becomes similar to that of a cohesive zone element; therefore, the influence of H can be implemented in a similar manner via an H degradation function while keeping the characteristic length-scale parameter *l* constant.

Several versions of phase field formulations for H assisted cracking were proposed in cases of linear elasticity^687^ and elastic-plastic response.^688^ The studies adopted the same H degradation function as that applied in cohesive zone modeling. The approach was benchmarked with a pre-cracked plate under tension, a constant load test to measure delayed fracture, and complex crack propagation arising from defects intrinsic to corrosive environments. Later, the approach was extended to the simulation of H assisted fatigue.^218^ However, the same problem of selecting an appropriate H degradation function, as encountered in cohesive zone modeling, pertains to the phase field fracture approach. Martínez-Pañeda et al.^687^ proposed a generic linear form for the H degradation function, with only one degradation parameter that can be estimated by fitting DFT data from the literature.

For both the cohesive zone and the phase field approach, a premise is to obtain an accurate account of local H distribution. A detailed review of stress driven H diffusion analysis is presented in Section 2.2.2. Obviously, a high local H concentration needs to be established in order to sufficiently decrease the fracture toughness of the material to trigger cracking. However, there was a concern that the local H concentration simulated with a conventional J2 plasticity theory was insufficient to trigger fracture. Martínez-Pañeda et al.^689^ showed that strain gradient plasticity could play a critical role in rationalizing HEDE-based arguments and capturing the transition to brittle fracture since a sufficiently high local H concentration is achievable with this model. Later, a phase field model for elastic-gradient-plastic solids undergoing HE^690^ was developed.

A phase field regularized cohesive zone model for the simulation of HE was recently proposed by Wu et al.,^691^ which has a potential to give a more physical representation of cohesive fracture compared to a conventional phase field model and able to simulate complex crack patterns compared to an explicit cohesive zone model. The model has currently been implemented with the HEDE mechanism only, but it is claimed able to incorporate the HELP mechanism simultaneously.

##### 2.3.6.2 HELP and Defactant Theory Based Modeling

For numerical simulations conducted at scales other than the atomistic scale, plasticity is a result of the collective behavior of the nucleation, multiplication, and motion of dislocations in the bulk. It is practically impossible to separate the HELP mechanism and the Defactant theory when it comes to modeling the influence of H on plasticity. We therefore review the numerical simulations under the theories of H enhanced plasticity in the same section. In the literature, this kind of numerical simulation is usually claimed to be based on the HELP mechanism, possibly because the term HELP explicitly describes the outcome of the simulation, and it was proposed with a theoretical derivation and was put into numerical simulation afterwards.^41,224^ In contrast, the Defactant theory is more generic and conceptual. Since the Defactant theory is related to H enhanced dislocation nucleation and activation of dislocation sources, it is an important ingredient of all the upper-scale H enhanced plasticity simulations.

Obviously, H will influence the plastic constitutive relation and fracture of a material. To implement the influence of H on the constitutive relation, the basis model most widely adopted is the classical J2 plasticity.^224^ This is an empirical model with only two plasticity related parameters, the yield strength and the strain hardening exponent. The consequence of the HELP mechanism is simplified as a softening in the flow stress and implemented by prescribing the yield strength in the model as a decreasing function of H concentration, while the strain hardening exponent is kept constant. The decreasing function is termed the H softening function and in most of the cases takes a simplest linear form due to the lack of accurate calibration.^692,693^ This has been the most popular treatment of H enhanced plasticity in modeling so far, because J2 plasticity is prevailing for modeling of metallic materials and the implementation of H is effortless. Such a treatment reflected only the role of H in enhancing the nucleation and multiplication of dislocations but neglected the enhanced entanglement of dislocations which is a possible product of H enhanced plasticity and is likely to promote plastic hardening. Therefore, H enhanced softening of the global loading curve is always an outcome. This leads to a major criticism to the H softening based simulations because there exists plenty of evidence where H negligibly influences the global loading curve or even exerts a hardening effect, which is elaborated in Section 2.4.1. Considering the influence of H on the strain hardening exponent in J2 plasticity can help enable the simulation of global hardening, but this is in the expense of the cleanliness of the model by introducing more fitting parameters.

##### 2.3.6.3 Crystal Plasticity Finite Element Models

An alternative is to adopt a physics-based plasticity model as a basis, which is expected to increase the universality of the model and to minimise the need of fitting parameters, since the model parameters and the influence of H can all be determined from first principles, in theory. In 2018, Castelluccio et al.^694^ implemented the effect of H in a crystal plasticity finite element (CPFE) model and was able to simulate the H induced hardening of loading curve observed in an H charged tensile experiment on single crystalline nickel as well as H induced softening observed in an H charged cyclic loading experiment. In that formulation, H was assumed to increase the drag stress opposing dislocation motion while reducing the barrier to dislocation multiplication. Yuan et al.^695^ implemented H reduced dislocation line tension and H reduced cross slip rate in a CPFE model and simulated H induced hardening effect. The hardening was attributed to H drag and reduced dislocation annihilation rate via cross slip. Zirkle et al.^696^ considered H solute drag on mobile dislocations as well as the role of dilute concentrations of VHs as obstacles to dislocation motion in a CPFE model, and simulated H induced hardening in single crystalline 316L stainless steel. In these works, H was assumed to reduce the line tension of dislocations and thus enhance the multiplication of dislocations, which is consistent with the Defactant theory. This was recognized as the dominant factor in the work of Yuan et al.^695^ but as a secondary effect by Zirkle et al.^696^ Castelluccio et al.^694^ and Zirkle et al.^696^ both assumed that H decreases dislocation mobility in their simulations, which is consistent with the H reduced mobility of edge dislocations discussed earlier.

While the application of these physics-based models as a basis is a promising way to simulate the H influenced plasticity, it is very far from being practical. Such an approach has significantly increased the number of fitting parameters in a HELP mechanism-based simulation, which caused more complexity and even uncertainty of the simulation. Existing models are to a large extent qualitative, and it remains a formidable task to calibrate any appropriate parameters from experiments or first principles calculations, while the latter seems an option that can become viable in the future. As reviewed earlier, it is already possible to study the influence of H on elementary dislocation activities with atomistic modeling, for instance, how and how much H influences the line tension, mobility, and nucleation of a dislocation. These are crucial information for the determination of CPFE parameters. CPFE models adopt a continuum description of dislocations, smearing dislocation aggregates as density fields. It is impossible to quantify the influence of H on dislocation density field parameters just by knowing its influence on a single dislocation line and on an elementary process. There exists a gap between atomistic simulation of a few dislocations and the CPFE model, which are different in length and time scales by several orders of magnitude. It would be helpful to start from the atomistic information of H effects on single dislocations and elementary dislocation activities as an input, simulate the multiplication and entanglement of a large number of dislocations, and calibrate the influence of H on the collective behavior of these dislocations. If such as simulation is carried out to the scale of large dislocation aggregates that get smeared as density fields in the CPFE model, the calibrated influence of H can reasonably be taken as input to the CPFE simulation. This is like establishing a bridge between the atomistic level simulation and the CPFE model at the continuum level, i.e., at a meso-scale, which enables the communication between the two levels with a minimal loss of information.

The so-called DDD approach provides a potential meso-scale bridge between atomistic simulation and continuum-level plasticity models. The approach considers dislocations explicitly as individual lines in three dimensions that move under applied stress and have mutual interaction. The influence of H on dislocation elastic stress field (elastic shielding), dislocation mobility, and dislocation core energy can be readily incorporated in this framework. H elastic shielding was implemented by Gu and El-Awady^697^ with H atoms taken as Eshelby inclusions, following the framework proposed by Sofronis^698^ and improved by Cai et al.^699^ It was demonstrated later by Yu et al.^44^ that H elastic shielding effect plays a secondary effect in *bcc* material because of the low bulk concentration. Instead, the influence of H on dislocation mobility makes a major contribution, which originates from the influence of H on the formation and migration of kink pairs in the screw case. Yu et al.^444^ further calibrated the influence of H on dislocation core energy or equivalently, on dislocation line tension, and implemented it in DDD simulation.

The simulation capacity of a small-scale modeling approach can be evaluated by the dimensions of the simulation box and the resulting dislocation density. To our knowledge, the simulation capacity of atomistic simulation is usually below 100 × 100 × 100 nm^3^, while the largest simulation that has been conducted with the DDD approach is 15 × 15 × 15 μm^3^,^700,701^ and the maximum dislocation density that has been reached is in the magnitude of 10^12^ m^–2^.^702^ DDD simulations in the presence of H are limited to a size of 12×3×3 μm^3^ and a density also in the magnitude of 10^12^ m^–2^.^444^ In contrast, the dislocation density in a CPFE simulation can easily reach the order of 10^14^ m^–2^.^703^ Therefore, the atomistic simulation and the continuum-level plasticity model such as CPFE are complementary approaches to simulating H enhanced plasticity, while the DDD approach provides a potential link between them. An H informed, physics-based plasticity modeling framework can possibly be established via the combination of these three approaches.

##### 2.3.6.4 H Facilitated Microvoid-Mediated Failure

Although the HELP mechanism does not have an explicit link to fracture, it is possible to rationalize the loss of load bearing capacity of a material subjected to H. A systematic investigation of H induced failure had been conducted by Sofronis and colleagues in the 2000s, assuming that H causes a plastic softening at a continuum level, i.e., applying an H softening function to the J2 plasticity model as discussed earlier. In 2001, Sofronis et al.^224^ introduced a linear softening function to the J2 plasticity model and coupled it to hydrostatic pressure-driven H redistribution in steady state. This essentially converted J2 plasticity model to a pressure-sensitive plasticity model. By analyzing such a model in a plane strain specimen, they concluded that H can promote localization of the homogeneous macroscopic plastic deformation into bands of intense shear. Later, Liang et al.^692^ furthered the investigation by simulating necking in a plane strain tensile specimen, which is another viable form of plastic instability, and they concluded that the presence of H can escalate necking which is the original form of failure in the absence of H. Whether H triggers shear localization as a new form of failure or escalating necking as the original form of failure depends on the form of defects in the specimen.

Although the process of H facilitated plastic localization does not explicitly involve material separation, it can be viewed as the initiation of H induced failure as it is accompanied by an obvious reduction in load bearing capacity. The ductile fracture of metallic materials in the absence of H has been well investigated, and it is widely accepted that the fracture can be rationalized as the consequence of a series of microvoid processes, including nucleation, growth and coalescence.^704^ Under loading, the nucleated voids grow, and plastic strain in the ligament between the voids accumulates. Koplik and Needleman^704^ demonstrated that a critical point exists during void growth, before which the volume of the void increases steadily under loading, but once reaching the critical point, the rate of void growth will become unstable. Obviously, the unstable void growth is accompanied by the “collapse” of the inter-void ligament and neighboring voids will quickly merge into one. The load bearing capacity of the ligament and consequently, of the material, is lost, and the material is separated, which leads to fracture. This critical event of ligament collapsing and voids merging is termed void coalescence. The beauty of Kolpik and Needleman’s work is that they demonstrated how plastic localization in the ligament actually caused void coalescence and fracture. This was realized by employing the so-called unit cell approach.^705^ A unit cell under this context is a single void-containing representative volume element with periodic boundary conditions, and the matrix material is assumed to obey J2 plasticity. By controlling the displacements applied at the boundaries of the unit cell, a constant stress triaxiality is enforced, and void growth and coalescence are simulated in every detail.

Now that H has been found to facilitate plastic localization in J2 plasticity and void coalescence is triggered by plastic localization of inter-void ligament, it is a natural next step to study its influence on microvoid processes. With the unit cell approach and applying a linear H softening function to J2 plasticity in the matrix material, Ahn et al.^706^ and Liang et al.^693^ investigated void growth and coalescence with different H softening and trapping characteristics. It was found that H enhances void coalescence by escalating plastic instability in the form of necking in the inter-void ligament. Interestingly, H was found to induce a shear band when the softening is severe and trapping is strong, which is a new form of plastic instability not observed in the absence of H. It was therefore suspected that void coalescence could occur by shear banding in the presence of H. Later, Yu et al.^707^ applied the same approach and furthered the study by implementing a failure criterion for void coalescence by shear banding and calibrating failure loci for materials with different H softening and trapping characteristics. It was shown that the critical void volume fraction remains quite small if failure occurs via shear banding, which could partly explain the H embrittled fracture surface compared to ductile fracture in the absence of H.

There exists a well-established predictive model for ductile fracture based on the microvoid processes, the so-called Gurson model^708^ which describes the constitutive relation of a material containing spherical voids and elastic–plastic matrix. With H-informed unit cell analysis, it is possible to implement the influence of H in the Gurson model as platform and develop a continuum model for considering the effect of H on void failure. Ahn et al.^709^ based on their unit cell simulation calibrated a traction–separation law with H and implemented it in a cohesive zone model to simulate crack propagation due to H-accelerated microvoid processes. Another option is to implement H directly in the Gurson model, which may be even more straightforward. In recent years, H informed Gurson model has attracted more attention, and several factors related to H, e.g., accelerated void nucleation,^710^ accelerated void growth,^711^ and reduced stress threshold for void coalescence,^712^ have been implemented. More about this model will be elaborated in Section 3.4, which focuses on predictive models of HE.

##### 2.3.6.5 H-Induced Fracture by Synergetic Effects of HELP and HEDE

As mentioned in Section 2.3.2.4, the concept of synergistic action of HE mechanisms is gaining popularity, and in particular, the synergy between the HELP mechanism and the HEDE mechanism has been verified experimentally. Barrera and Cocks^713^ adopted the unit cell approach to simulate H induced fracture at a carbide in a steel. The representative volume element contained a spherical particle at the centre, the matrix material was assigned J2 plasticity with a linear H softening function, and the interface between the matrix and the particle was modelled with H informed cohesive zone model with a linear H degradation function. H weakened the interface and promoted debonding of the particle from the matrix, which led to the formation of a void; the growth of the void was then accelerated by H induced plastic softening. In this way, both the HEDE and HELP mechanisms were considered in the same simulation. Huang and Gao^714^ applied the same concept in their simulation but adopted a different practical implementation of the HEDE mechanism. They employed the phase field fracture model and applied a linear H degradation function on the fracture toughness of the material; at the same time, they applied a linear H softening function to the yield strength of the material. The authors found that the model could capture the transition from ductile to brittle fracture induced by H, which was more significant when the two mechanisms were combined than when only one mechanism was considered. Lin et al.^715^ applied an H-informed Gurson model to account for the HELP mechanism, together with an H-informed cohesive zone model with a bilinear H degradation function in their simulation. The Gurson model was calibrated using the unit cell approach with J2 plasticity theory and a linear H softening function. A simplified approach to modeling the combined effect of HELP and HEDE is to run a H-accelerated ductile damage model and a H-weakened cleavage fracture model at the same time, and take whichever that occurs earlier as the actual failure.^716,717^ Recently, Lee et al.^718,719^ implemented the synergistic action of HELP and HEDE in steel based on the so-called unified mechanics theory. The theory quantifies material degradation by the total specific entropy production rate. Entropy generation terms due to H-enhanced micro-plasticity, H-enhanced decohesion and H-induced dilatation were derived and incorporated in the unified mechanics theory. In this manner, the HEDE and HELP mechanisms are treated consistently, which enables convenient comparison of the contribution of individual mechanisms.

A common feature of the simulations reviewed above is that H-induced plastic softening at a continuum level was adopted as the presumption of the HELP mechanism, and ductile fracture is considered as one of the viable fracture modes. When the concentration of H is high enough, ductile fracture mode transits to brittle fracture which is handled by the cohesive zone model or the phase field fracture model. On the one hand, the HELP mechanism helps establish certain conditions favorable for the HEDE mechanism to operate, for instance, increasing local H concentration because of increased localized plasticity hence trapping. On the other hand, these two mechanisms are somewhat in “competition”, for example, the HELP mechanism tends to reduce the local stress because of the plastic softening, which is not favorable for the HEDE mechanism to come into effect. As a matter of fact, all the authors mentioned a transition of fracture model in their studies, which is an indication of the “competition”.

It is not necessary to implement H induced plastic softening when simulating the synergistic action of the HELP and the HEDE mechanisms. In 2010, Novak et al.^467^ proposed a model based on a different microscopic perspective. They assumed that H-induced fracture occurs in a locally stress-controlled manner at grain-boundary carbides; the role of H is to enhance the multiplication and mobility of dislocations, and the dislocations in turn transport H with them until they are arrested at GB carbides; the consequence of these processes is to elevate the local stress at the carbides due to the impingement of dislocations, and to weaken the carbide interfaces because of the accumulation of H. The authors reasonably adopted the weakest-link theory^720^ for cleavage fracture to capture H induced fracture which usually takes place in a quasi-cleavage or IG manner. The effective work of decohesion of a GB was assumed to decrease as local H concentration increases; the local H concentration is calculated as the sum of the concentration of lattice H, carbide trapped H, GB trapped H, and dislocation trapped and transported H, and the last term increases with plastic strain. Obviously, this approach well fits the so-called H-enhanced and plasticity-mediated decohesion mechanism that was proposed two years later.^37^ Later, Nagao et al.^468^ employed the same approach to simulate HE in lath martensitic steels, and extended its application from H induced IG fracture to TG quasi-cleavage fracture. More about this approach is found in a review article by Dadfarnia et al.^721^

##### 2.3.6.6 Modeling H-Induced Failure with HESIV and HELP

It is very likely a scenario that strain-induced vacancies in a metallic material agglomerate and form microvoids, the HESIV mechanism therefore has a close link to the nucleation of microvoids. The Gurson model mentioned earlier can serve as a great platform to implement the HESIV mechanism, which naturally combines the HESIV and HELP mechanisms in a continuum-level simulation. The feasibility of such an approach was discussed by Nagumo et al.^722^ as early as in 2001, soon after the experimental evidence of the HESIV mechanism was reported. H was assumed to increase both the nucleation and growth rate of voids, by a somewhat arbitrary factor, and it was illustrated that a reduction in fracture toughness was captured by such a treatment. The study was qualitative, without a micromechanical calibration of the influence of H or coupling to H diffusion, so the practical applicability was limited. While the HESIV mechanism enjoyed great popularity over the past two decades, little follow-up study was conducted on H-informed Gurson modeling until 2019^711^ and the concept has increased in popularity since then.^710−712,723^

As mentioned in the previous section, an H-informed Gurson model is more about predictive modeling of HE than about mechanistic investigation, so the technical details of this approach will be presented in Section 3.4. We put here a brief discussion about aspects related to the basics of the mechanism. First, it is possible to identify a clear boundary between the HESIV mechanism and the HELP mechanism in a Gurson model-based simulation, although they inevitably overlap. Obviously, aspects about H promoted void growth and coalescence via plastic instability should be attributed to the HELP mechanism. Regarding void nucleation, the HESIV mechanism applies if it is presumed that void nucleation is dominated by the agglomeration of vacancies, which is likely the case in pure metals and should be strain-controlled, as discussed by Nagumo and Takai.^20^ However, it should be mentioned that void nucleation was usually attributed to the debonding of matrix from impurity particles in alloys,^724^ which can be either stress- or strain-controlled. If a stress-controlled void nucleation function is applied, then the HEDE mechanism which promotes the debonding may be applicable instead of the HESIV mechanism.

Second, the HESIV mechanism may operate beyond the void nucleation stage, or even skip this stage, and play a direct role in promoting fracture. As discussed in Section 2.3.5, H could induce the agglomeration of nano-vacancies at a GB in nickel; through a process similar to the HESIV mechanism, this significantly weakens the GB and facilitates IG fracture.^659^ In a sense, this process is similar to the GB carbide-mediated decohesion assumption as adopted by Novak et al.^467^ In such a scenario, the Gurson model-based platform may still be used but a new fracture mode, even a new HE mechanism such as HEDE, needs to be incorporated.

##### 2.3.6.7 Modeling H Induced Fracture with AIDE

Practical simulation of HE based on the AIDE mechanism is very challenging. This mechanism acts on an existing free surface, e.g., a microcrack; H that adsorbs on the surface facilitates dislocation emission; the emitted dislocations do not contribute to local plastic strain or stress field ahead of the crack tip; instead, they instantly move away and leave displacement steps on the surface. Due to the repeated dislocation emission, the displacement steps are so large at the crack tip as to advance the crack. As stated by Lynch,^22,436^ the AIDE mechanism leads to a reduced plasticity ahead of the crack tip, which is contrary to the H enhanced plasticity mechanism. This distinction implies that current dislocation-based plasticity and damage models that are compatible with H enhanced plasticity mechanism are likely not compatible with the AIDE mechanism. Since the AIDE mechanism predicts a sharp crack with a suppressed plasticity zone ahead of the crack tip, the stress intensity factor may seem higher than the case of the HELP mechanism, as a larger portion of the applied stress intensity can be passed to the crack tip due to the reduced plastic shielding. However, this may not actually be the case since the enhanced emission of dislocations and the movement of the dislocations away must consume an increased amount of energy, this part of energy may still be regarded as a form of plastic dissipation but does not increase the plastic strain ahead of the crack tip. Therefore, it is difficult to apply the AIDE mechanism in a boundary layer model^47^ which is frequently employed in fracture mechanics to quantify the amount of plastic dissipation during crack propagation.

The only option seems to describe the fracture toughness of the material directly as a decreasing function of the adsorbed H coverage, without explicitly simulating the process of H enhanced dislocation emission. This is then purely a phenomenological model that can be implemented following the same strategy as the HEDE mechanism-based modeling. This part of discussion is consistent with the statement in Section 2.3.2.6 that the AIDE mechanism and the HEDE mechanism are similar. The AIDE mechanism focuses on adsorbed H and reduced dislocation emission threshold which is usually related to resolved shear stresses, while the HEDE mechanism focuses on absorbed H and reduced interfacial separation threshold which is usually dependent on normal stresses. Under a phenomenological modeling framework, it is difficult to distinguish between these two, but HEDE mechanism is more frequently invoked, possibly because the absorbed H is more convenient to consider. Interestingly, acknowledging the difficulty with AIDE-based simulation and its similarity with HEDE-based simulation helps deepen our understanding of the nature of the AIDE mechanism.

The challenge of numerical simulation based on the AIDE mechanism does not undermine the significance of the theory. It shows that there lacks a model which is capable of capturing the crack advancement due to displacement steps associated with crack tip dislocation emission, and at the same time capable of simulating the motion of the emitted dislocations in the bulk. Another key aspect of AIDE mechanism-based modeling is the accurate account of adsorbed H at a free surface and within a small distance beneath the surface (the so-called sub-surface). This is fundamentally different from the prevailing H diffusion analysis approaches, e.g., that based on the Fick’s law and Oriani’s equilibrium, which are valid only for H in the bulk.

##### 2.3.6.8 Key Challenges with Mechanistic Modeling of HE

There remain unsolved issues with each of the modeling approaches reviewed earlier. These are reflections of three key challenges with mechanistic modeling of HE in general.

To begin with, first principles-based, multiscale modeling of HE remains a challenge. From the atomistic scale to the laboratory scale, the numerical investigation of HE spans over seven orders of magnitude in temporal and spatial scales. Ideally, HE phenomena at the laboratory scale should be predicted with a multiscale modeling approach which integrates the information obtained at various scales, starting from the atomistic scale and requiring little empirical fitting. However, the communication of information across scales is very challenging and may cause great inaccuracy. Existing methods either cover a limited number of length scales or lack sufficient resolution on intermediate scales. For instance, the H-informed DDD simulation only concerns the atomistic scale and sub-micron scale, e.g., size of a few dislocations, insufficient to predict H influenced plasticity properties at a continuum scale; in contrast, many H-informed cohesive zone simulations adopt an atomistically calibrated H degradation law directly to model the failure of a laboratory specimen, omitting several important length scales in between. A desirable multiscale modeling method should cover a wide span of length scales and seamlessly connect the intermediate ones, to minimize the loss of information when “climbing up the scales”.

Second, a direct link between H influenced plasticity and H-induced fracture is still lacking, as elaborated in Section 2.3.2.4. It is therefore not possible to simulate H-induced fracture in an initially perfect material, such as high purity single crystalline iron or nickel, while the influence of H on plasticity has been extensively studied with MD and DDD approaches. Linking H-enhanced plasticity and fracture in numerical simulations will be a significant breakthrough for the mechanistic understanding and predictive modeling of HE.

Finally, an accurate account of H uptake and transport in a material is necessary for the mechanistic modeling of HE but remains a challenge. In particular, an accurate analysis of the content of adsorbed H on a free surface in contact with H and its relation with the amount of H absorbed into the bulk are crucial for the interpretation of experiments; an appropriate mapping between the amount of adsorbed H at free surfaces inside a material, such as surfaces of an imbedded microcrack, and the concentration of H in the bulk, is a prerequisite for AIDE mechanism-based modeling as mentioned above; the transport of H via dislocations is a key ingredient of HE mechanisms emphasizing on the synergistic action of HELP and HEDE, but this is still yet to be accurately implemented in numerical models.

### 2.4 H-Induced Degradation of Mechanical Properties

Mechanical properties of a material determine the structural integrity of an engineering component, and H affects these properties profoundly. Recognizing and predicting the influence of H on mechanical properties is key to mitigating the detrimental effects of HE. The mechanical properties most relevant to green H applications and susceptible to H include the constitutive behavior, the fracture property and the fatigue property. The constitutive behavior refers to the relation between stress and strain both in the elasticity range and the plasticity range, the relation can also be categorized as a non-fracture property; an accurate description of this relation is the premise for the prediction of H induced failure. The fracture property here specifically refers to fracture under monotonic loading, to make a distinction from fatigue which refers to the damage and fracture under cyclic loading. The consideration is that the two loading scenarios are equally relevant to engineering applications of H and the phenomena of HE have been found to differ significantly between the two scenarios. As a matter of fact, fatigue itself has been established as a largely independent research area from classical fracture mechanics. Under the context of H, both the fracture property and the fatigue property concern the transition of fracture mode and the degradation of fracture resistance. The fracture mode transition is a change induced by H in the morphology of fracture surface, while the latter is a reduction in the threshold of fracture, for instance, the fracture toughness in monotonic loading and the frequency dependent crack growth rate in cyclic loading. In this section, we review the consequences of HE on the aforementioned mechanical properties. The influence of H on fracture mode has been elaborated in Section 2.3.2; therefore, we emphasize on the degradation of fracture resistance in this section.

#### 2.4.1 H-Induced Change of Non-fracture Properties

Depending on the type of load, loading rate, temperature, features of the metallurgy state, and the nature, distribution, and density of generated or pre-existing defects, H can exert a softening and/or hardening effect on the constitutive behavior of a material, and the influence extends to both elasticity and plasticity properties.

##### 2.4.1.1 Effect of H on Apparent Elasticity Properties

The elastic stiffness, or modulus tensor *C*_ijkl_, is a fourth-rank tensor describing the constitutive relation between the stress *σ_ij_* and strain *ε_ij_* in a linear elastic material. The elastic stiffness tensor can be obtained as the second derivative of the internal energy density with respect to the strain field:
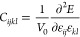
27where *V*_0_ is the volume of interest and *E* is the total internal energy of the volume as a function of the strain field. The elasticity tensor has 81 components in total; however, it has only 36 independent components due to symmetry properties. Adopting the so-called Voigt notation, the elastic stiffness matrix can be expressed as
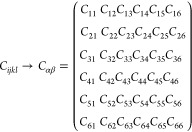
Any addition of solute or defect, such as H, is susceptible to modify the energy of the system and thus impact the elastic properties. There exist a few experiments that measure the impact of H on elastic properties under tension and nanoindentation.^448,725,726^ Both H-induced hardening and softening of elastic properties have been experimentally observed, and the apparent discrepancy has been partially understood with the help of numerical simulation. Recent atomistic calculations well illustrated the impact of solute and point defect on elastic properties.^448,727−729^ For a cubic structure, there exist only three independent components, *C*_11_, *C*_12_ and *C*_44_, due to symmetry. These constants were found to decrease as a function of the H content expressed in occupancy, θ, with a linear equation *C_ij_*(θ) = *C_ij_*–*n_ij_* × θ. The slope *n_ij_* depends on the orientation and material considered, with a value lower than 200 MPa. This leads to a moderate softening effect of H on elastic properties (2 GPa for 1 at % of H). The influence of H on elasticity associated with H solute can originate from two distinct aspects. One is a mechanical aspect, i.e., the addition of H solute induces volume expansion of the host lattice; the other is a chemical aspect, i.e., H solute alters the local atomic bonding in metals and/or has an electronic contribution.^727^ In each case of uniformly distributed H, the volumetric expansion induces a softening effect on elastic properties, while the chemical aspect induces a hardening effect. According to a study on pure metal, the elastic properties increase with the density of electron,^730^ which supports the fact that the electronic contribution of H induces hardening.

**Figure 36:**
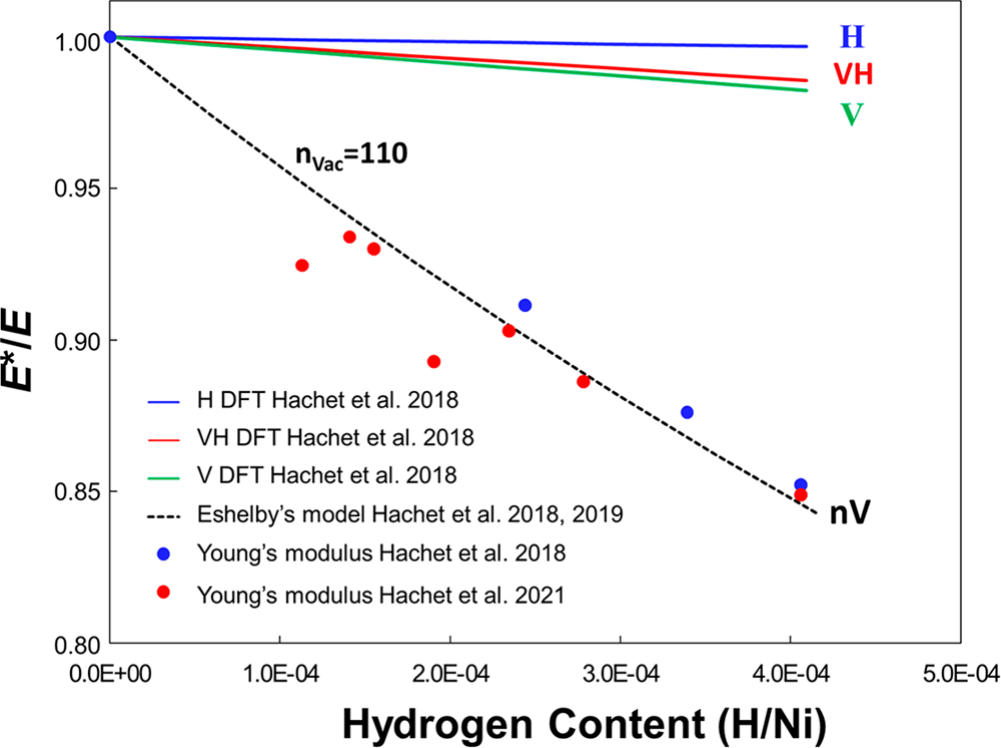
Evolution of the ratio E*/*E* between effective Young’s modulus E* (with H) and Young’s modulus *E* (without H) in <100> tensile direction as function with H content for a nickel single crystal. Adapted with permission from refs (727) and (736). Copyright 2018 and 2019 Elsevier. The dots represent experimental data, while the solid and dashed lines represent simulation results; H, V, and VH in the figure represent the case with H, the case with monovacancies, and the case with simple VHs; nV refers to the case with a large vacancy cluster, with the n representing the number of monovacancies in the cluster.

The influence of H on elastic properties originating from the volumetric expansion can be derived analytically. Describing the volume expansion contribution of H as the partial volume of H, *V̅_H_* in m^3^/at., the influence of the elastic stress/strain field on the chemical potential for solute can be treated with a thermodynamic framework:^135,448,699,731−734^
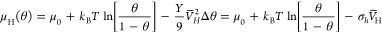
28where *Y* is an elastic parameter which depends on the orientation and components of the stiffness tensor,^699^ and *σ_h_* is the hydrostatic stress. This approach can be extended to any other centrosymmetric defects as vacancies or cluster of vacancies with the introduction of the notion of partial volume of defect *V̅*_d_. By using the equation of the chemical potential, the H diffusion equation (Fick laws) and the hypothesis that the H distribution is in equilibrium with the local stress gradients, the apparent compliance *S*_ijkl_* due to the incorporation of H or centrosymmetric defect can be expressed as follows:^135,448,732^
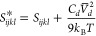
29The Young’s modulus in the <100> direction can be expressed as:
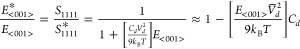
30The above equation is in good agreement with the atomistic calculations for H solute and vacancies.^727^ Recently, Hachet et al.^727^ showed that the impact of H on elastic stiffness is not consistent with experimental observation in pure <100> nickel single crystal. This indicated the need to consider the impact of vacancy clusters on elastic properties, e.g., with the so-called super abundant vacancies model initially proposed by Fukai.^735^ To improve the accuracy of the model when relating numerical simulation to experimental data, it is necessary to consider a vacancy cluster with a size of 2 nm, which was observed experimentally with TEM,^727^ see Figure 36. This indicates the influence of vacancy clusters is dominant over that of H on the elastic modulus.

##### 2.4.1.2 Antagonistic Effect: H-Induced Plastic Hardening and Softening

H influences the plasticity properties significantly, which may manifest as a softening or hardening effect on the global loading curve. Investigation on such an effect helps to deepen the mechanistic understanding of HE and to calibrate a constitutive relation of a material as a function of H, which is an indispensable ingredient in predictive modeling of H induced failure.

However, controversial experimental observations exist on the role of H on the global stress–strain curve, supporting both plastic softening and hardening induced by H. This is an important aspect of the diversity of HE phenomena as elaborated in Section 2.1.2. An example of the diversity is the series of experiments conducted by Matsui, Kimura, and Moriya in the 1980s on the influence of H on tensile loading curve of iron.^737−740^ Iron is one of the most important base materials for H applications and is a perfect subject of the study as the complexity with alloying elements is ruled out. The authors observed both H induced plastic softening and hardening in a series of tensile tests they had performed, and they depicted several factors influencing the outcome of a test. (i) Purity of iron: H-induced plastic softening is only observed when the content of carbon is below a critical value; otherwise, H induces plastic hardening; apparently, the purity of a material plays an important role. (ii) Temperature: H causes softening in high purity iron at temperatures between 200 K and 300 K but causes hardening at temperatures below 200 K. (iii) H concentration: softening is observed at a low concentration and hardening is observed when the concentration exceeds a critical value. (iv) Electrochemical charging condition: H-induced softening is observed if surface damage occurs due to improper charging condition, e.g., a very high current density.

**Figure 37:**
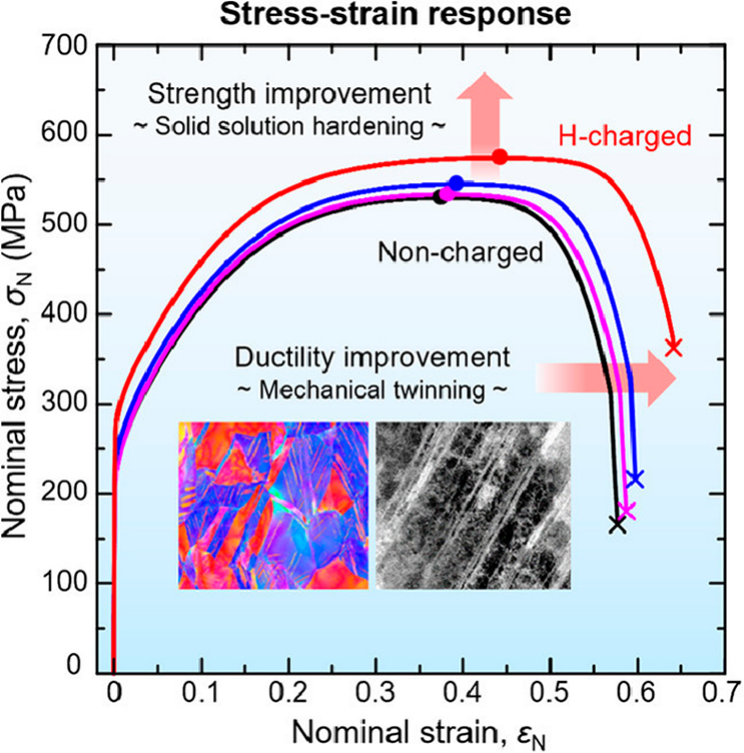
In stainless steel, H was observed to exert a significant plastic hardening effect; interestingly, the ductility of the material was enhanced by H charging, indicating that the material is resistant to HE. Reprinted with permission from ref (745). Copyright 2020 Elsevier under [CC BY 4.0 DEED] [https://creativecommons.org/licenses/by/4.0/].

In recent years, the antagonistic character of the effect of H on plasticity has been well recognized, and it has become a consensus that whether H induces softening or hardening is dependent on a combination of complex factors, such as material microstructure, H content, H diffusivity, and loading condition.^11,640,736,741,742^ Consequently, the investigation of H on plastic softening or hardening is conducted with more sophisticated small-scale mechanical testing and analysis methods that allow for a more accurate probing of local event of H–dislocation interaction, and the studies are more case-specific. The most important features are on the one hand to differentiate the direct effects of H from those induced by the formation of point defects and on the other hand to consider the effects of H on the dislocation pattern and the interaction of the slip bands with GBs. The H-induced hardening effect at the early stage of the deformation has been observed in pure nickel,^481^ in ferritic stainless steel,^640^ austenitic stainless steel,^11^ and nickel alloys.^742,743^ The solute accumulation around the edge components of a dislocation loop or dipole can cause a pinning effect similar as a Cottrell atmosphere. This pinning then caused an increase in the constraint to move the dislocation. This effect was observed during tensile test of single crystalline nickel, in fatigue of single crystalline nickel, and the *in situ* nano-indentation tests.^481,742^ According to Girardin and Delafosse,^742^ this constraint was evaluated through experimental measurements of the solute drag with dynamic strain ageing. Gu and El-Awady^697^ explained this hardening based on DDD simulations and Hachet et al.^744^ evaluated the pinning effect of edge dislocation using atomistic calculation and Mott-Nabarro-Labusch model. Ogawa et al.^745^ also observed significant H-induced hardening effect in an austenitic stainless steel, as shown in Figure 37. The authors interpreted the phenomenon as H-induced resistance to dislocation movement and attributed the hardening to a solid-solution strengthening mechanism with H being an alloying element, while the dragging force exerted by H on dislocations was deemed secondary. It is interesting to note that H not only induced hardening but also increased the ductility in the experiment, i.e., both the load bearing and deformation capacity of the material increased after H charging. The authors ascribed this to the increased density of mechanical twins in the presence of H. This study indicated that H could act as an alloying element and simultaneously increase the strength and ductility of a material. This could be valuable information for the design of materials against HE.

**Figure 38:**
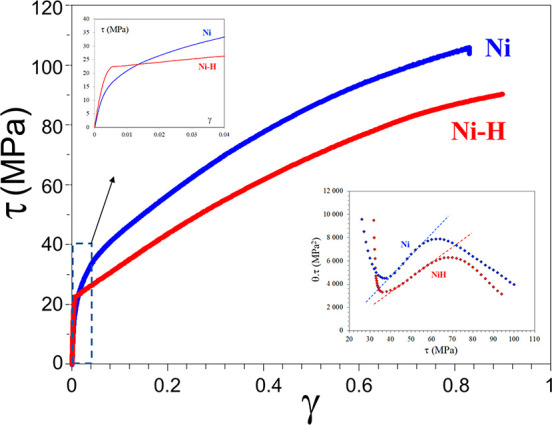
Tensile stress vs strain curve of nickel (100) single crystal without H (blue curve) and with H (red curve, 7 wppm) at room temperature (strain rate of 10^–3^ s^–1^). Adapted with permission from ref (56). Copyright 2019 Springer Nature under [CC BY 4.0 DEED] [https://creativecommons.org/licenses/by/4.0/]. H induces a hardening at the early stage of the deformation (lower than 0.01) and a softening for larges deformations (illustrated by a zoom in picture). Kock and Mecking representation vs allows to illustrate a softening effect of H on the hardening rate θ (slope of the dotted curves).

The H–dislocation interactions and their effects on the different stages in the stress–strain curve of *fcc* single crystals oriented for easy glide and multiple slip have been extensively studied.^56,733,742^ These studies highlighted an H-induced hardening effect in stage-I due to the viscous drag of Cottrell atmosphere, and a softening effect in stage-II due to shielding effect of H and delayed cross-slip event; the studies also reported a distinct increase or decrease of hardening rate correlated with the impact of H on dislocation patterning (Figure 38).^56^ Studies of the formation of dislocation cells in the presence of H, both in single crystals and polycrystals, highlighted the decrease of inter-wall distance and consequently the length of dislocation mean free path, which rationalized the increase of hardening rate as a function of H concentration.^741^ Ghermaoui et al.^56^ investigated the effect of H on the tensile stress–strain behavior of nickel oriented for multiple-slips <001> using multi-scale approaches (Figure 38), and revealed a contradicting phenomenon of H induced decrease of hardening rate, which is correlated with the decrease of dislocation density in cell walls resulting from the shielding effect at different scales. Hachet et al.^744,746^ observed similar correlation between H and the length of dislocation mean free path for the dislocation pattern produced under cyclic loading; dipolar walls spacing increased with the addition of H, which increased the length of dislocation mean free path and consequently induced a softening effect. Moreover, the authors claimed that the clusters of superabundant vacancies accelerate the H-induced softening process more than a direct impact of H. The fact that H enhances the generation of vacancies during the deformation is supported by several recent studies^175,659^ and can be a rationale for H-induced IG fracture.

Technological advancement in the past decade has provided the opportunity to clarify some antagonistic roles of H on the elementary processes of elasticity and plasticity. Still, many incomprehensive results persist mainly in relation with the dynamics of the systems studied. The antagonistic effect of the chemical and mechanical contribution of H on the elastic properties offers new possibilities for designing new alloys by considering the possible contribution of the interaction of H with other solutes. The competition between the direct impact of H on plasticity and the H enhanced formation of vacancies need to be clarified as a function of strain rate. Furthermore, the interaction of H with other solutes and precipitates along with the consequence on plasticity remains an important topic for future investigation.

#### 2.4.2 H-Induced Degradation of Fracture Property

##### 2.4.2.1 H-Induced Ductility Loss for Specimens without a Pre-crack

A prominent feature of HE is the H-induced loss of ductility. Ductility refers to the ability of a material to deform plastically under tension without fracturing. However, there are different ways for calculating ductility. In the case of a smooth specimen without initial stress concentration, the ductility is measured by determining the percentage of elongation or the percentage of area reduction. When stress concentrators exist, such as a notch in a tensile specimen, ductility can be calculated as the average fracture strain, e.g., calculated based on the dimensional change of the minimum cross section. While H may induce either hardening or softening of the loading curve, there is a general consensus that H decreases the ductility of metallic material, except for the few studies which reported an increase of ductility.^745^

A widely applied experimental approach to evaluate the loss of ductility due to H is H-charged tensile test. Smooth or notched tensile specimens are subjected to H charging and tensile loading. By comparing the loading curves recorded during the tests, the reduction of ductility is readily demonstrated. H-charged tensile tests can be carried out *in situ* or *ex situ*. In the former scenario, tensile load is applied to the specimen while H charging is ongoing; in the latter, tensile test is conducted on specimens pre-charged with H, i.e., after H charging. To allow maximum H uptake during charging and sufficient redistribution of H during loading, thus being conservative, SSRT test is often applied.^193^

**Figure 39:**
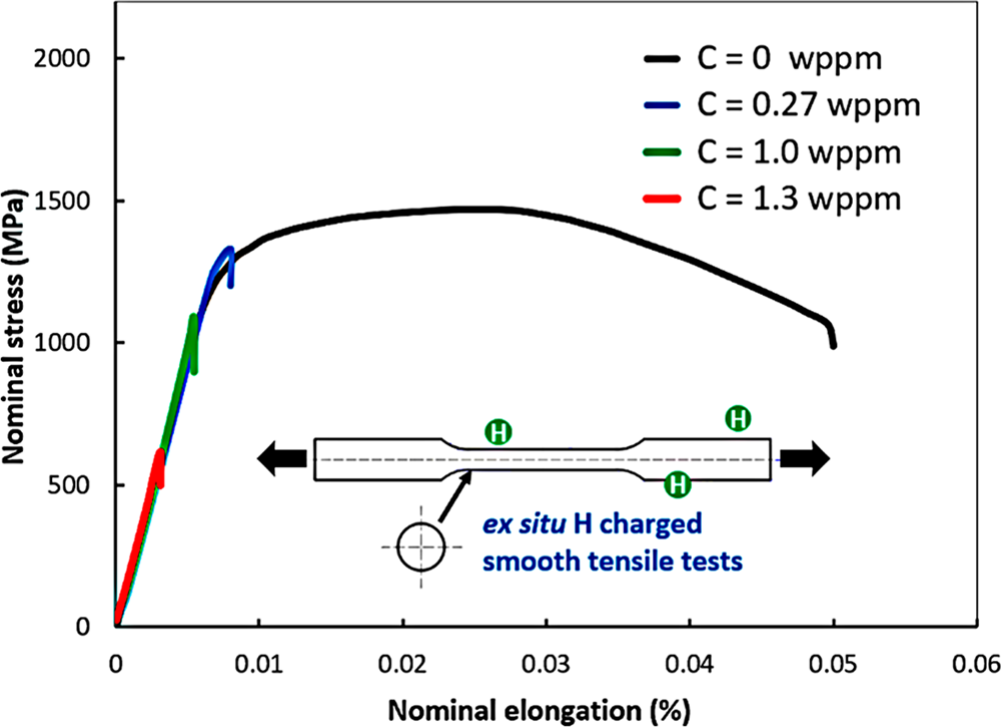
H-induced loss of ductility as manifested by the nominal stress vs elongation curves recorded in *ex situ* SSRT tests. The tests were conducted on smooth specimens with different diffusible H contents. H-induced loss of ductility escalates with the increase of H content. Redrawn with permission based on ref (193). Copyright 2007 Elsevier.

Significant loss of ductility in the presence of H has been reported both in *in situ* and *ex situ* tests. Wang et al.^193^ performed a series of *ex situ* SSRT experiments on smooth and notched tensile bars made of a high strength steel, the specimens were pre-charged with H for different durations, thus possessing different contents of H. The fracture strain, or ductility, was found to decrease severely with the increase of H content, a reduction of about 90% was observed in a smooth tensile bar charged with an H concentration of 1.3 wppm, as illustrated in Figure 39. A significant loss of ductility due to H was also observed in pipeline steel,^747^ austenitic steel^12^ and nickel alloy.^13^ Similar observations were obtained in *in situ* experiments.^235,501,748^

While H causes a reduction of ductility in general, the exact degree of reduction depends on a combination of material, geometric constraint, and H concentration. For pipeline steels, there is a consensus that the susceptibility to ductility loss increases with the strength of the material. For instance, Nanninga et al.^749^ compared the HE susceptibility of three API grades of pipeline steels, namely, X52, X65, and X100, under similar H and loading conditions, and they found that the susceptibility to HE increased with the steel grade, i.e., the strength. The HE susceptibility of austenitic steel is highly dependent on its microstructure, for instance, austenitic steels with appropriate deformation twinning were found to be less susceptible. In fact, an interesting phenomenon of the so-called H-induced ductilization, where the material becomes even more ductile in the presence of H, was recently reported.^505,745^ The studies indicated that appropriate twinability engineering could be a promising path toward mitigation of HE, as discussed in Section 3.3. The susceptibility to H-induced loss of ductility was found to increase with the level of geometric constraint. In the tensile experiments on a X70 pipeline steel conducted by Depraetere et al.,^747^ the relative reduction in ductility was found to decrease as the notch radius of the tensile specimen reduced.

**Figure 40:**
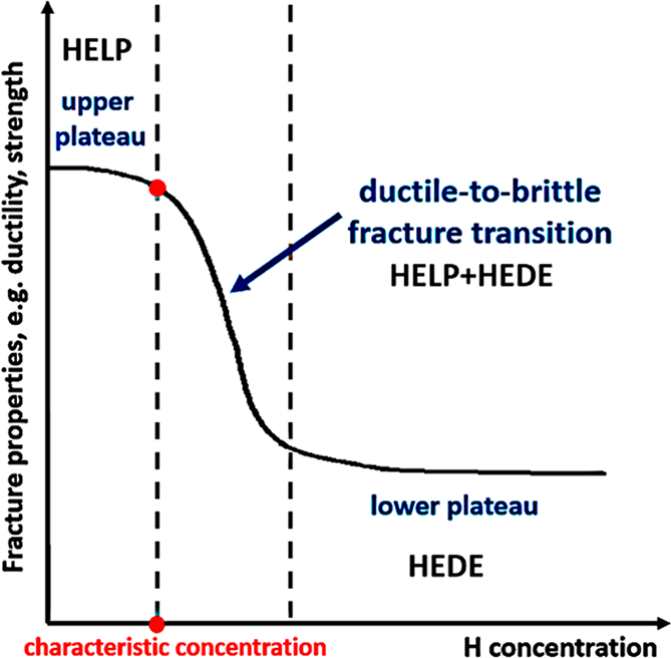
Illustration of “S”-shaped ductile-to-brittle transition curve in the presence of H. The fracture properties decrease mildly at low concentrations of H, but significantly and rapidly when the concentration became sufficiently high; after a narrow window of ductile-to-brittle fracture transition, a lower plateau is reached where the fracture properties decrease only mildly with further increase of H concentration. For engineering applications, the point where the sudden decrease occurs is critical, and a characteristic H concentration is defined there. Adapted with permission from ref (40). Copyright 2019 Elsevier. Note that the characteristic concentration is defined at the beginning of the transition zone here, while it was defined at the end of the transition zone by Djukic et al.^40^

While the degree of H-induced loss of ductility increases with H concentration, the relationship between these two is not proportional. Dmytrakh et al.^750^ observed that the extent of H-induced ductility loss escalates with increasing H concentration, yet this relationship is not linear. In their tensile tests on H-pre-charged specimens, ductility, gauged by failure strain, diminished mildly at low H levels but drastically at high concentrations. They proposed the existence of a specific H concentration threshold, or characteristic H concentration, marking a shift in HE mechanism. A similar trend was reported by Liu et al.^751^ The existence of such a characteristic H concentration becomes more evident when plotting the variation of critical stress versus the concentration, as illustrated by Dmytrakh et al.^750^ The critical stress decreases only mildly within a range of low H concentrations until the characteristic value; beyond the value, there’s a sharp drop in strength within a narrow concentration range. This strength degradation stabilizes at a lower limit with further concentration increase. This behavior delineates three stages: upper plateau (low concentrations), lower plateau (high concentrations), and a steep transition connecting them, forming an “S” shape, with the characteristic H concentration identified at the onset of the steep drop. An illustration of such a curve is presented in Figure 40. The “S”-shaped ductile-to-brittle transition curve in the presence of H was discussed in detail by Djukic et al.^40^ and Lin et al.^715^ Note that the H concentration refers to the global concentration in this figure. HE-related fracture is a manifestation of H–dislocation interactions (HELP for instance) and “local HEDE micro-incidents”^752^ related to decohesion and microcracking. The latter occurs when the local H concentration is sufficiently high. Upon the first appearance of a few local HEDE micro-incidents, the global H concentration remains low although local H concentration is high at the few spots, so the upper plateau in Figure 40 still applies. For HEDE to predominantly cause macroscopic damage, leading to a significant reduction of strength, ductility, and fracture toughness, a large volume must be affected by numerous local HEDE micro-incidents. Under such widespread HEDE influence, the role of HELP becomes less significant, particularly as the global H content approaches or exceeds the characteristic concentration, which manifests as the lower plateau in Figure 40. In some steels, the characteristic H concentration can be very low, resulting in a short upper plateau on the transition curve that can be difficult to distinguish. For instance, this was observed in a high strength steel tested by Wang et al.^753^ and later analyzed by Ayas et al.^754^

The “S”-shaped ductile-to-brittle transition in the presence of H is also supported by fractography analysis, which reveals a gradual transition of fracture mode as the H concentration increases. The fracture surface in the absence of H has a dimpled feature produced by a microvoid process as discussed in Section 2.3.2.4, while quasi-cleavage or IG fracture is usually observed when the H concentration is sufficiently high. In the aforementioned experiment, Dmytrakh et al.^750^ found that dimples still remain the dominant feature of fracture surface when H concentration is low; quasi-cleavage was observed only when the concentration of H was high enough. In an *in situ* H charging tensile experiment on a tensile specimen made of pipeline steel,^755^ H-induced quasi-cleavage was observed in the region close to the notch surface; moving away from the notch root to the centre of the specimen, small dimples became visible even under low magnification; ductile fracture features with large dimples were observed at the centre of the specimen. It is hypothesized that the transition is related to the nonhomogeneous distribution of H. The concentration of H is the maximum in the region close to the notch root and decreases towards the center. The observation of fracture mode transition is consistent with the observations made in *ex situ* tensile experiments.

A single damage process and HE mechanism may not be sufficient to account for the fracture mode transition and the S-shaped degradation pattern. A viable explanation for the phenomena is a transition from the microvoid process when H is absent, to the HELP mechanism in the range of low concentrations and to the HEDE mechanism when the concentration is high enough. The HELP mechanism doesn’t change the nature of fracture but accelerates inter-ligament necking and slightly decreases the critical fracture strain of microvoids; the HEDE mechanism could cause the direct decohesion of the inter-ligament between voids by decreasing the cohesive strength, which leads to fracture much earlier and most importantly, with the voids remaining small. The feasibility of such a concept has been demonstrated by Lin et al.^712,715^ through a series of studies. By coupling with H, the authors were able to develop a predictive model unifying H enhanced plasticity and decohesion. With the model, the S-shaped HE pattern was produced numerically.

##### 2.4.2.2 H-Induced Toughness Reduction for Specimens with a Pre-crack

Fracture mechanics approach applies when there is a pre-existing crack. According to fracture mechanics, cracking is controlled by the stress intensity factor, energy release rate or crack tip opening displacement.^757^ The crack will propagate when the crack tip driving force exceeds a critical value. This critical value is termed fracture toughness, both the critical stress intensity factor and the critical energy release rate have been used as the measures of fracture toughness.

**Figure 41:**
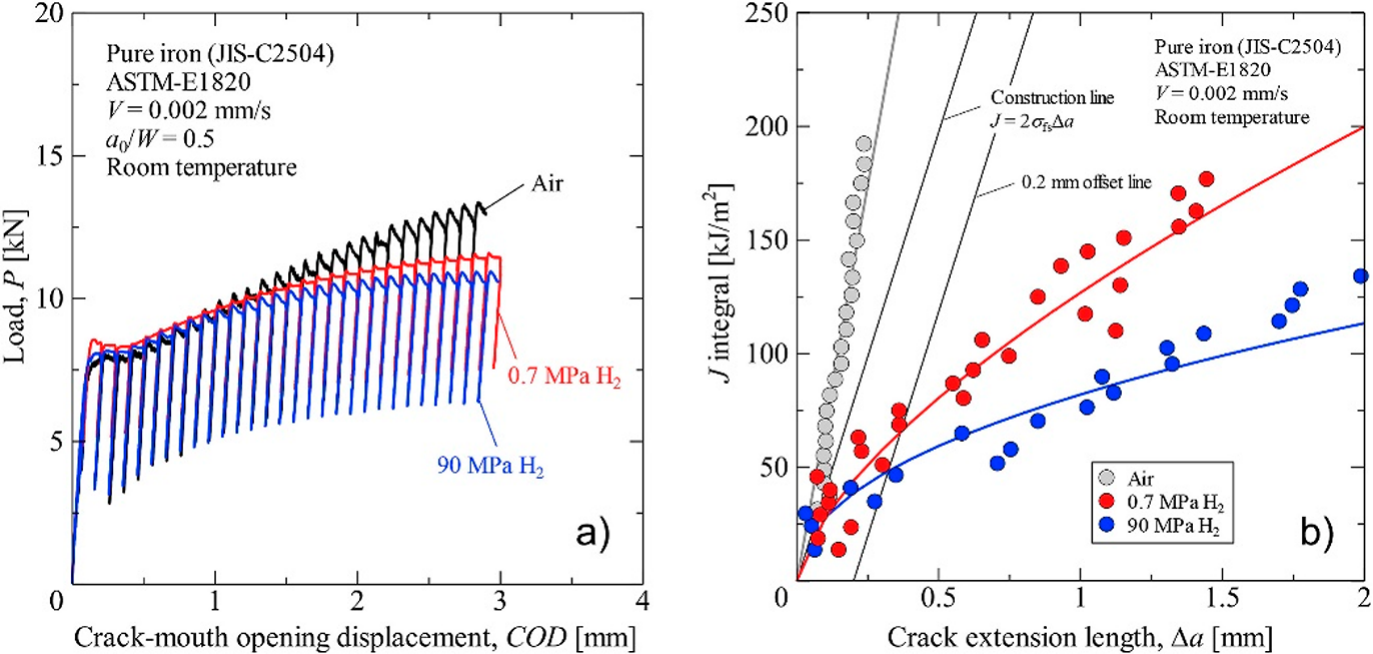
H-assisted crack propagation in α-iron during elastoplastic fracture toughness tests. (a) Relationship between the load and the crack-tip opening displacement and (b) the corresponding crack-growth resistance curve (J–R curve) for specimens tested in air and in 0.7 MPa- and 90 MPa-H gas at room temperature. Reprinted with permission from ref (756). Copyright 2019 Elsevier.

Fracture toughness tests, e.g., three-point bending test and compact tension test, have been adopted by a number of researchers to experimentally measure the H-promoted fracture. Álvarez et al.^758^ conducted three-point bending test on gaseous H pre-charged specimens and found that internal H significantly reduced the fracture toughness of a structural steel. Birenis et al.^756^ conducted *in situ* compact tension test on iron in gaseous H. Samples tested in air possessed large crack-opening and blunted crack-tips, while specimens tested in H_2_ had much smaller crack-openings and substantially sharper tips, as shown in Figure 41a; specimens tested in H_2_ were found to have a significantly lower fracture toughness as shown in Figure 41b. Halilović et al.^759^ conducted three-point bending test under *in situ* H charging condition and found that H reduced the fracture toughness of a high strength steel. In their experiments, the fracture surface still displayed considerable amount of dimpled feature when the concentration of H was low; the fractography became IG at a high concentration. The fracture toughness was found to decrease only mildly over a range of small concentration, but a sudden drop was observed when the concentration was sufficiently large. Presumably, the H induced degradation of fracture toughness also follows an S-shaped pattern as discussed in the previous subsection. Li et al.^760^ combined single edge notch tension test and electrochemical H pre-charging and reported H-reduced fracture toughness in an X90 grade pipeline steel. Note that the authors employed crack tip opening displacement which is an equivalent measure of fracture toughness.^757^

The so-called modified boundary layer model is a classical tool to apply fracture mechanics. A widely adopted version of the model is a circular disc containing a crack at the center,^761^ assuming elasticity and prescribing displacements to the remote circular boundary of the disc, a target stress intensity can be enforced at the crack.^762^ With the approach, Ahn et al.^709^ simulated the reduction of fracture toughness due to H promoted MVC. Song and Curtin^47^ conducted MD simulation of such a circular modified boundary layer model, and they applied stress intensity to the remote circular boundary using the formulation of anisotropic elasticity. As elaborated in Section 2.3.2, H was found to suppress the emission of dislocations and reduce plastic dissipation close to the crack tip; a larger portion of the applied stress intensity is therefore available to drive crack propagation. This can be a viable explanation to the H reduced fracture toughness in steel.

##### 2.4.2.3 Combined Effects of Crack Tip Constraint and H on Fracture

When a fracture mechanics approach is utilized to characterize HE resistance, the geometry of the chosen specimen will exert an influence on the measured results, which poses a challenge to transferring lab tests to real full size components under service conditions.

**Figure 42:**
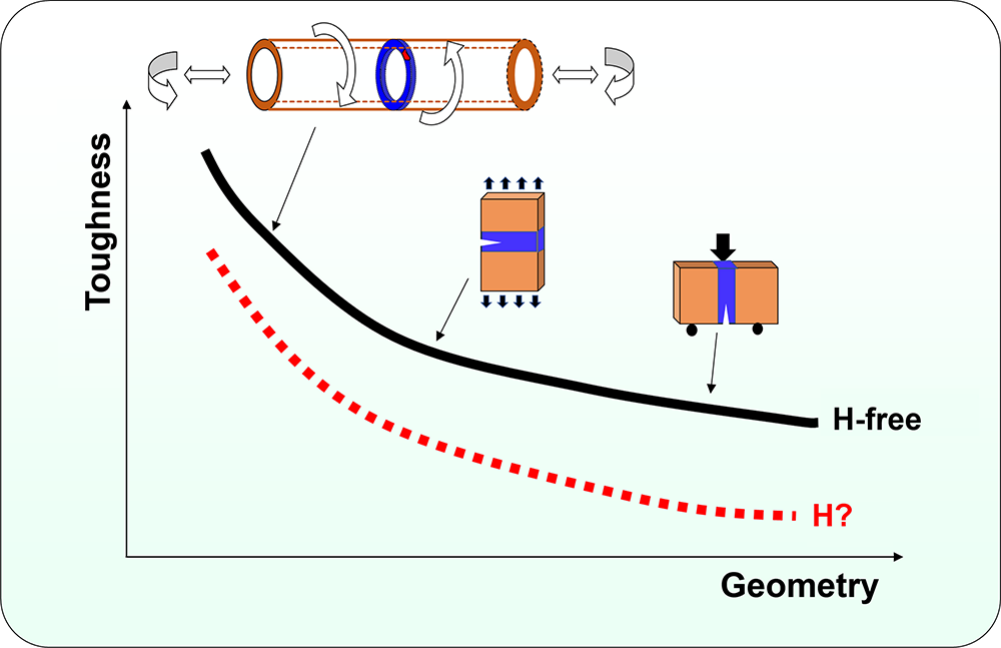
Schematic illustrating the effect of geometric constraint on fracture toughness. Fracture toughness decreases with the increase of crack tip constraint in air (solid curve). The influence of constraint in the presence of H depends on multiple factors, such H concentration and the embrittlement mechanism.

In classical fracture mechanics, fracture toughness is regarded as a unique material parameter, but research in the last three decades has clearly shown that fracture toughness of a material is not fixed, and crack tip constraint of a specimen strongly influences the measured fracture toughness. Crack tip constraint, which reflects the level of hydrostatic stress in relationship to equivalent stress, is a function of specimen geometry, loading condition, and crack size. For example, for the same geometry and loading condition, crack tip constraint increases with the increase of crack size. For the same crack size, a bending specimen yields higher crack tip constraint than a single notched tension specimen. In an H-free case, it is well understood that the fracture toughness of a ductile material in general decreases with the increase of crack tip constraint, e.g., the fracture roughness measured using a specimen under bending is lower than that using the same specimen under tension. Figure 42 shows schematically the effect of crack tip constraint on the measured fracture toughness.

Few studies have been carried out on the combined effect of H and geometric constraint on fracture toughness. In a numerical investigation, Liang et al.^763^ applied the modified boundary layer model and assumed a microvoid process-based crack propagation; they implemented *T*-stress^757^ to control the crack tip constraint, with a positive *T*-stress representing a high constraint and a negative *T*-stress representing a low constraint; H induced plastic softening and lattice dilatation were considered. The authors demonstrated that a smaller fracture toughness applied in the scenario with a high level of constraint, in the absence of H. With H, plastic softening, lattice dilatation, and T-stress were found to act synergistically to enhance void growth, and H could reduce the fracture toughness considerably even when the level of constraint was low. The authors warned that “the common assumption that deep-notch toughness data lead simply to a conservative defect assessment of low constraint structural components may not always hold in the presence of H.”

Elazzizi et al.^764^ noted in an X52 grade steel a decrease in fracture toughness both in the absence and presence of H as the level of geometric constraint increases. The authors suggested that fracture was undertaken by the microvoid process, and the mode of void failure changes when the level of constraint varies. With low constraint, voids were elongated in the tensile and shear direction, which resulted in zigzag crack extension; with high constraint, voids were sheared but not enlarged, and crack path was linear along a direction close to pure mode II bifurcation angle.

Li et al.^760^ conducted single edge notch tension tests on H pre-charged specimens with different thickness. A thicker specimen had a higher level of constraint in this experiment, and CTOD was selected as the measure of fracture toughness. A specimen with a higher constraint was found to possess a smaller fracture toughness; H was found to decrease the fracture toughness in all the specimens; and importantly, the reduction of fracture toughness due to H was found to be significantly larger with higher constraint. With TEM, the authors observed decreased dislocation density and therefore suppressed plasticity in a specimen with a higher constraint, which indicated that a higher concentration of H was built up at the crack tip. The authors then postulated that H-induced fracture occurred via the HEDE mechanism, and the severe reduction of fracture toughness in a highly constrained specimen was attributed to the suppressed plasticity. They suggested to take the constraint effect into account when assessing H-degraded fracture toughness, to maximize the transferability from fracture specimens in laboratory testing to the practical structures.

Recently, Halilović et al.^759^ explored the impact of pre-crack length ratio, which is a measure of geometric constraint, on the H-reduced fracture toughness in single edge notch bend tests of high strength steel. H was introduced using *in situ* electrochemical charging method, and a larger length ratio represented a higher level of constraint. In general, a specimen with a higher constraint level was observed to be more sensitive to HE, which was attributed to the suppressed plasticity and therefore higher hydrostatic stress built up at the crack tip. The conclusion is consistent with that reported by Li et al.^760^. Meanwhile, inconsistency existed in that a lower sensitivity to HE in a thicker specimen, which should also possess a higher level of constraint, was reported by Halilović et al.^759^ Further investigation is needed to probe the discrepancy. In all the above-reviewed studies, the influence of geometric constraint on HE was attributed to the interplay between H, plasticity, and constraint.

##### 2.4.2.4 Predictive Models and Key Challenges

While experimental observation of fractography is relatively easy, predictive modeling of fracture mode transition is very challenging. First, the underlying mechanism beneath the transition of fracture mode is highly dependent on the combination of material, environmental and loading conditions. Quasi-cleavage is often observed in *bcc* materials, while *fcc* materials usually displays an IG fracture mode; at low temperature, H-induced cleavage could apply. Furthermore, the transition of fracture mode takes place gradually as the concentration of H increases. At a low-to-medium H concentration, there is a mixture of ductile-like dimpled feature and brittle-like quasi-cleavage feature, which should be adequately accounted for by a predictive model. In this regard, an accurate account of local H concentration is a prerequisite for predictive modeling.

Mechanism-based modeling is highly desirable for its sound physical basis and fewer fitting parameters, however, many underlying processes of HE, although qualitatively understood, are still yet to be implemented quantitatively. For instance, H-induced IG fracture, decorated with nano-sized voids, may be better described with a so-called H-enhanced, strain-induced vacancy stockpiling model,^659^ but it is still not possible to simulate this process numerically. While H-enhanced dislocation activity is believed to be a key mechanism for quasi-cleavage with tearing ridges on the surface, it is still not possible to simulate the morphology of such a quasi-cleavage surface with those microscopic features.

Except for numerical modeling itself, quantitative experimental characterization technique is highly desirable. Accurate measurement of local plastic strain field over the entire loading history with and without H will greatly facilitate model development, providing a reference for the calibration and verification of models. Similarly, there needs a way to follow the growth of voids in three dimensions and measure the variation of void volume fraction over time, so that a microvoid process-based model can be validated.

As will be discussed in detail in Section 2.4.4, the ultimate purpose of the laboratory tests is to move from material screening to parameter identification and enable lifetime prediction of H energy systems. Currently, simple stress-based or strain-based failure criteria incorporating empirical functions to account for the influence of H are commonly employed to simulate experimental results.^193,754^ More advanced numerical approaches, e.g., CZM and phase field model, have also been put into application, which enhances the predictive capacity. However, two key challenges remain in predictive modeling of HE.

- The first concerns the transferability of the numerical models across different loading modes and geometric constraints. This is a known challenge for fracture mechanics in the cases without H, and it becomes even more problematic in the presence of H because the extent of degradation due to H is also dependent on the geometric constraint.
- The other is transferability between laboratory testing and in-service conditions. An engineering component under service is subjected to a complex combination of loads, highly dynamic aggressive environments, and aging of material. It is obviously impractical to reproduce exactly the in-service conditions in the lab. The challenge is then how to set laboratory testing conditions that are representative of the engineering applications in real life. This is crucial for the application of a laboratory-calibrated HE model to engineering failure assessment.

To tackle these challenges, mechanism-based modeling can be a powerful tool to limit the number of fitting parameters and ensure the accuracy of prediction. In addition, developing physics-guided, data-driven numerical approaches can be a promising strategy to achieve a balance between accuracy and computational efficiency. To apply these numerical approaches, it is necessary to develop reliable testing methods which adequately account for the multiphysics of HE and promise transferability of the calibrated material parameters.

Finally, material inhomogeneity is yet to be considered in predictive models. The issue is closely related to engineering applications of green H. For instance, H transport pipelines are joined through welding; due to the local heat input and high temperature gradient during welding, the microstructure of weld metal and the adjacent heat-affected zone is highly inhomogeneous and differs significantly from that of the base metal. In addition, both weld metal and the heat-affected zone are sensitive to manufacturing defects and consequently more prone to failure. Not only are these regions mechanically weaker, but they may also exhibit increased susceptibility to HE compared to the base metal. Unfortunately, very limited knowledge is available regarding the H uptake, H–metal interactions, and resulting property modifications caused by HE in these regions. Understanding and addressing these aspects are essential for ensuring the safe and efficient operation of H pipelines and optimizing their performance for the future.

#### 2.4.3 Cyclic Loading and Fatigue

##### 2.4.3.1 H-Accelerated Fatigue Crack Growth

**Figure 43:**
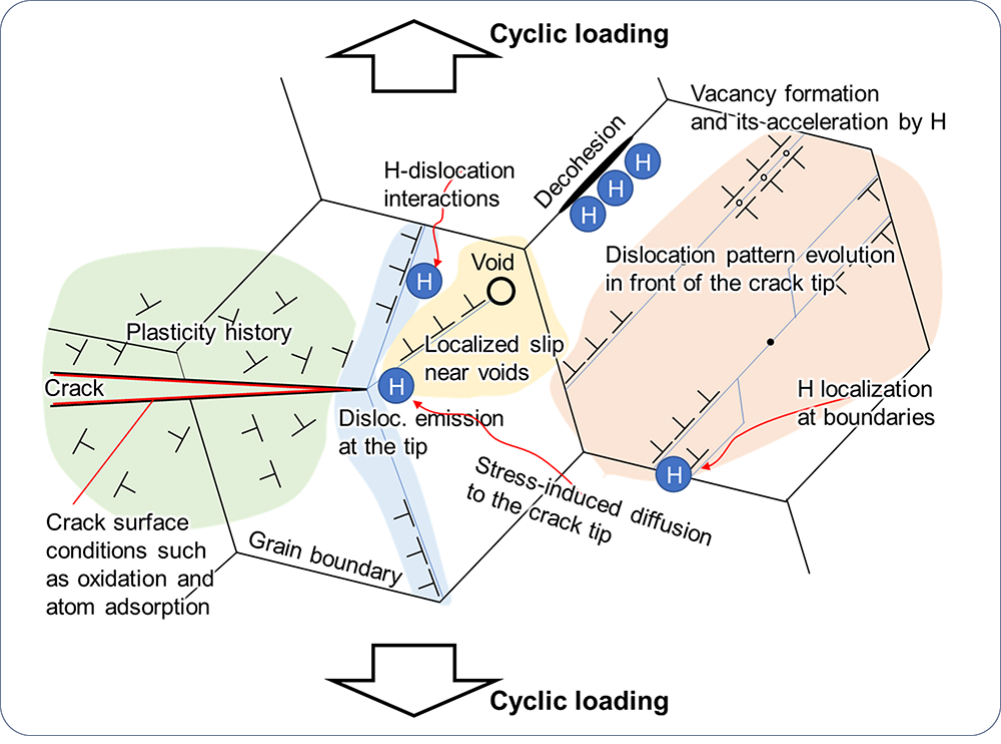
Schematic illustration showing the influential factors in H-promoted FCG.

Fatigue properties are crucial for structural design due to their influence on failure at stress levels below yield strength. In H environments, such as pressure vessels for H storage, H significantly accelerates fatigue crack initiation and growth.^765^ Fatigue crack initiation is commonly observed at sites of micro and macro-stress concentrations, like inclusions or small scratches. The FCG behavior is extensively analyzed to estimate the fatigue life of structures. Specifically in H atmospheres, FCG rates vary based on several factors such as plasticity history, crack surface conditions, and H localization at high hydrostatic stress areas and microstructural interfaces, as illustrated in Figure 43. The combined effects of plasticity and H lead to a reduced crack closure effect,^766^ increased dislocation emission at the crack tip,^767^ microscopic void/crack formation ahead of the crack tip,^767−769^ and vacancy formation in the evolved dislocation patterns.^770^ Consequently, the extent of H-accelerated FCG depends on variables like H content/pressure,^771^ test frequency,^470,771^ and stress ratio.^126,766,772^ Material systems also influence H-related FCG behavior, for instance, type 316L austenitic stainless steels exhibit notable FCG resistance even under 115-MPa H gas pressure.^773^ However, for cost and strength considerations, ferritic and tempered martensitic steels are preferred for H-related structures. Therefore, this section primarily discusses H effects on FCG in these steels and also briefly addresses the impact of crystal structure on H-related fatigue behavior in steels and Ni alloys.

##### 2.4.3.2 H Pressure and Frequency Effects

**Figure 44:**
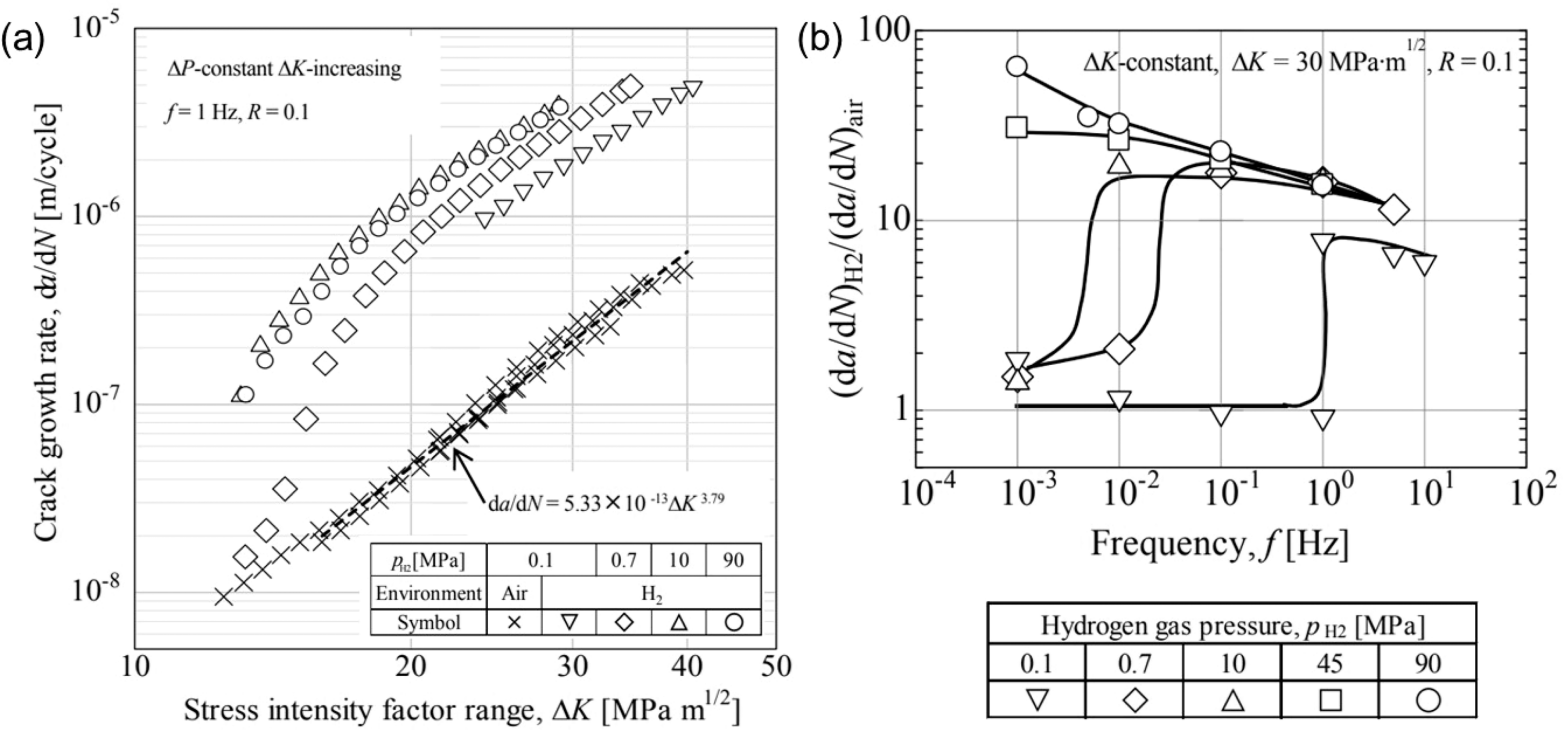
CT test results in air and H atmospheres with (a) different H gas pressures and (b) frequencies in a low carbon steel (SM490B). The tensile strength was 540 MPa. Reprinted with permission from ref (776). Copyright 2014 Japan Society of Mechanical Engineers.

Figure 44 presents FCG test results for ferrite–pearlite low carbon steel under varying H pressures and frequencies, using compact tension (CT) testing. The results illustrate a clear acceleration in FCG, measured as the ratio, (da/dN)_H2_/(da/dN)_air_, with rising H gas pressure (Figure 44a). Furthermore, lower testing frequencies enhance FCG acceleration, although this effect strongly depends on the stress intensity factor range Δ*K* (Figure 44b). Below 10 MPa H gas pressure, the (da/dN)_H2_/(da/dN)_air_ ratio gradually rises with decreasing frequency before experiencing a sudden drop. This pattern is commonly observed in various steel types, including tempered martensitic steel,^774^ cast-iron, and austenitic steel.^775^ Notably, the peak of the (da/dN)_H2_/(da/dN)_air_ ratio shifts towards lower frequencies with an increase in H gas pressure. At and above 45 MPa H gas pressure, the (da/dN)_H2_/(da/dN)_air_ ratio does not show the sudden decrease at low frequencies. In particular, at 45 MPa H gas pressure, the ratio stabilizes around 30. However, at 90 MPa H gas pressure, the (da/dN)_H2_/(da/dN)_air_ ratio continuously rises with decreasing frequency down to 10^–3^ Hz. This ongoing increase in (da/dN)_H2_/(dadN)_air_ with decreasing frequency poses a significant challenge for the use of steels in H environments in actual structures, complicating conservative structural design and necessitating tests at frequencies below 10^–3^ Hz.

**Figure 45:**
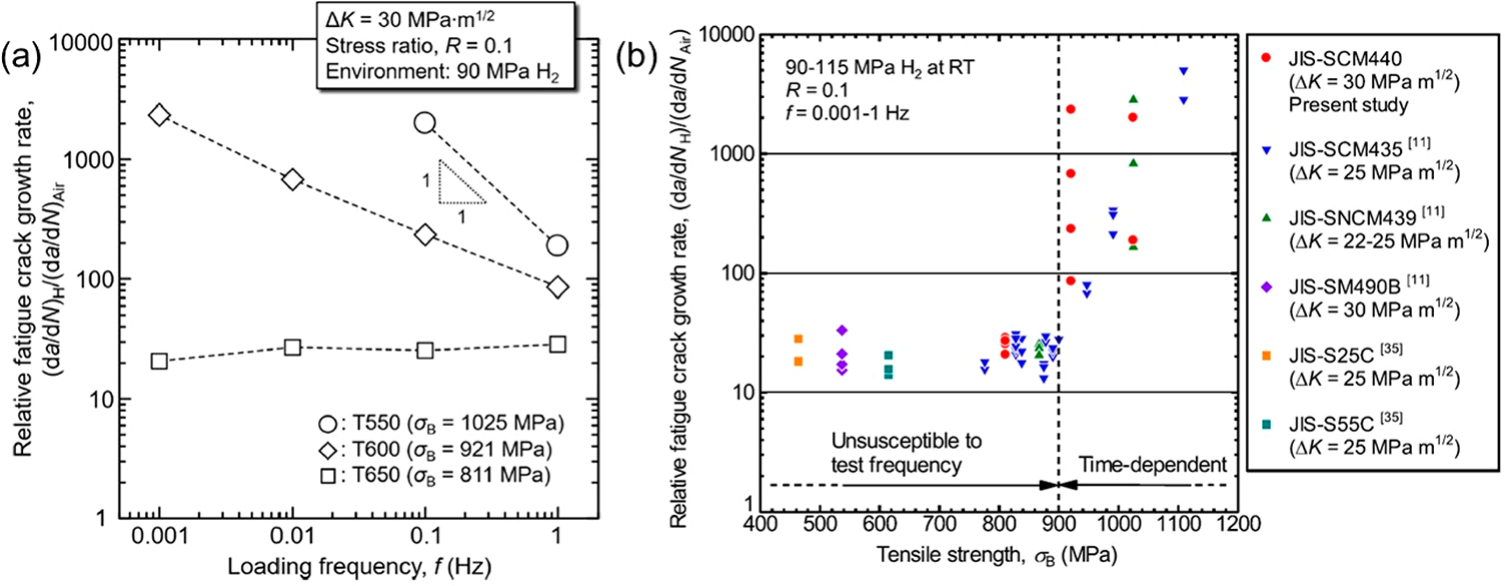
(a) Loading frequency effect on FCG acceleration in tempered martensitic steels with different tensile strengths. (b) Critical strength level for the drastic acceleration of FCG with a significant frequency dependence in various ferritic and tempered martensitic steels. Reprinted with permission from ref (777). Copyright 2022 Elsevier under [CC BY 4.0 DEED] [https://creativecommons.org/licenses/by/4.0/].

In contrast to ferritic steels, tempered martensitic steels, known for being ductile high-strength steels, exhibit different FCG behavior. As depicted in Figure 44b, at the same stress intensity factor range (Δ*K*), a tempered martensitic steel with a tensile strength of 811 MPa shows minimal frequency dependence of the ratio (da/dN)_H2_/(da/dN)_air_ until 10^–3^ Hz at 90 MPa H gas pressure (Figure 45a). This negligible frequency dependence suggests that FCG acceleration by H is primarily cycle-dependent, not time-dependent per loading cycle. This characteristic is termed cycle-dependent (da/dN)_H2_/(da/dN)_air_. The minimal frequency impact on (da/dN)_H2_/(da/dN)_air_ eases the mechanical design of structures with this material. However, in tempered martensitic steels with tensile strengths over 900 MPa, a notable frequency dependence of (da/dN)_H2_/(da/dN)_air_ emerges, more pronounced than in ferritic steels. In particular, tempered martensitic steels with 1 GPa tensile strength exhibit a tenfold increase in (da/dN)_H2_/(da/dN)_air_ when frequency decreases tenfold, indicating a highly time-dependent (da/dN)_H2_/(da/dN)_air_ behavior. This transition from cycle-dependent to time-dependent H-accelerated FCG occurs at the critical tensile strength of 900 MPa in ferritic steels as well as tempered martensitic steels, as shown in Figure 45b. An important feature of time-dependent H-accelerated FCG is the occurrence of IG FCG, likely due to decohesion. Suppression of the H-related decohesion of prior austenite GBs ahead of the fatigue crack tip is crucial for mitigating time-dependent FCG in tempered martensitic steels.

##### 2.4.3.3 Effects of Impurity Gases

**Figure 46:**
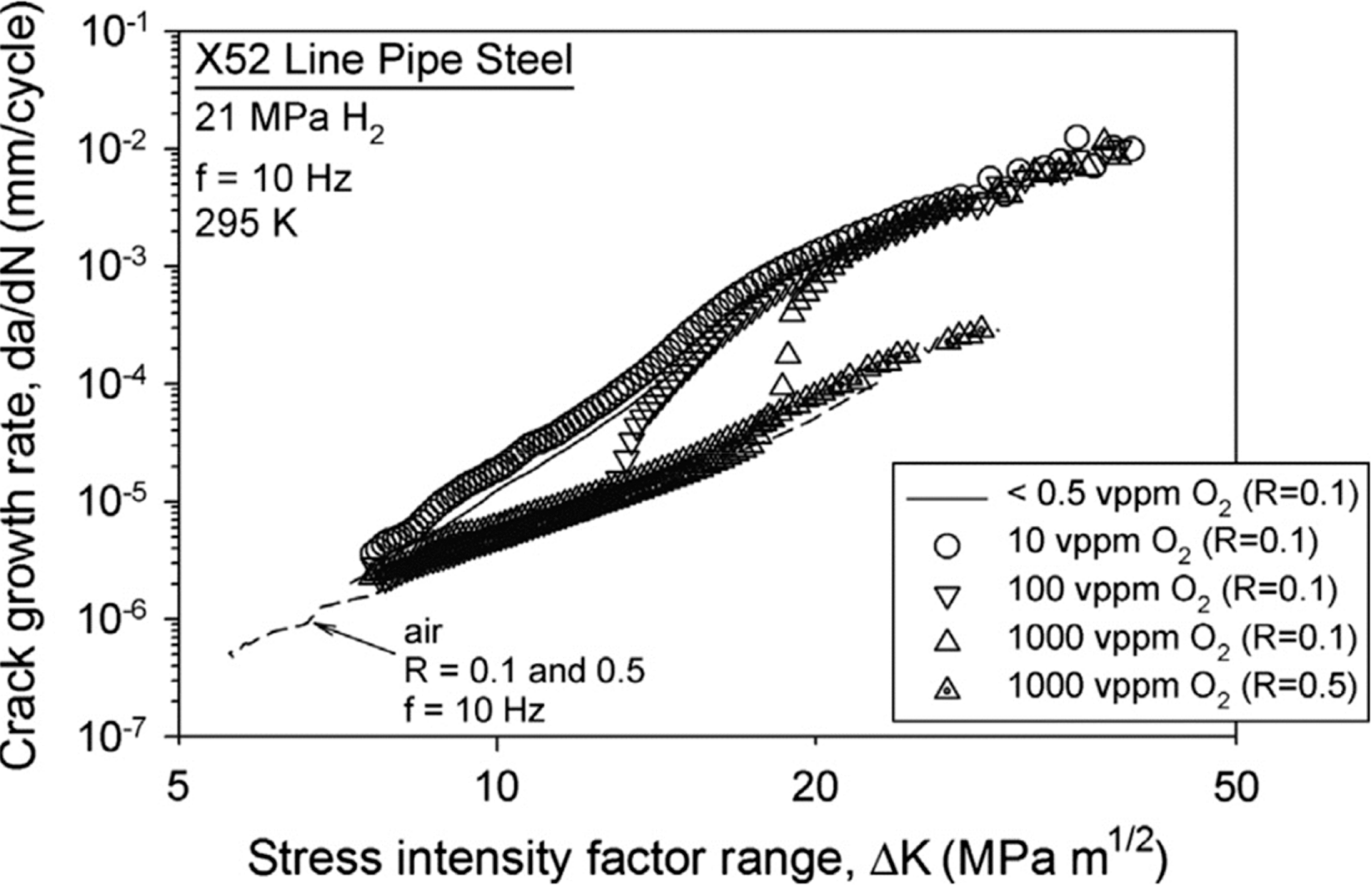
FCG rates with different impurity oxygen concentrations in the H gas. The tested material was ferrite–pearlite steel (X52 line pipe steel) with the tensile strength of 493 MPa. Reprinted with permission from ref (126). Copyright 2013 Elsevier.

An additional critical factor in the context of FCG is the role of impurity gases like oxygen and CO in high purity H gas. These impurities are known to inhibit H uptake and consequently suppress H-induced acceleration of FCG in ferrite–pearlite steel.^126,129^Figure 46 illustrates the effect of different oxygen impurity concentrations in H gas on FCG rates. Similar to how H pressure influences frequency dependence at lower H pressures, the oxygen impurity concentration affects the point at which accelerated crack growth begins. With an oxygen concentration of 10 vppm, the FCG rate is nearly the same as that in pure H. However, increasing the oxygen concentration to 100 vppm or more shifts the transition point of FCG acceleration to a higher Δ*K*. At these elevated oxygen levels, the rates of H-accelerated crack growth around the transition point rise more steeply with Δ*K* compared to lower oxygen concentrations and pure H. This is due to the competition between oxygen and H adsorption on iron surfaces.^123^ Thus, the interaction between crack growth rate and oxygen transport on the newly formed crack surface leads to the observed oxygen concentration dependence in H-related FCG.^126^

##### 2.4.3.4 Transgranular FCG

**Figure 47:**
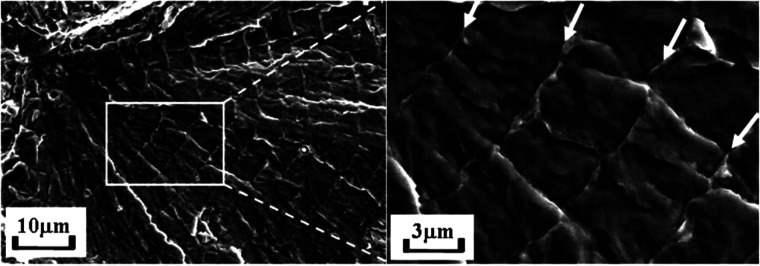
An example of H-related brittle-like striations on a fatigue fracture surface. Reprinted with permission from ref (768). Copyright 2011 Japan Society of Mechanical Engineers.

**Figure 48:**
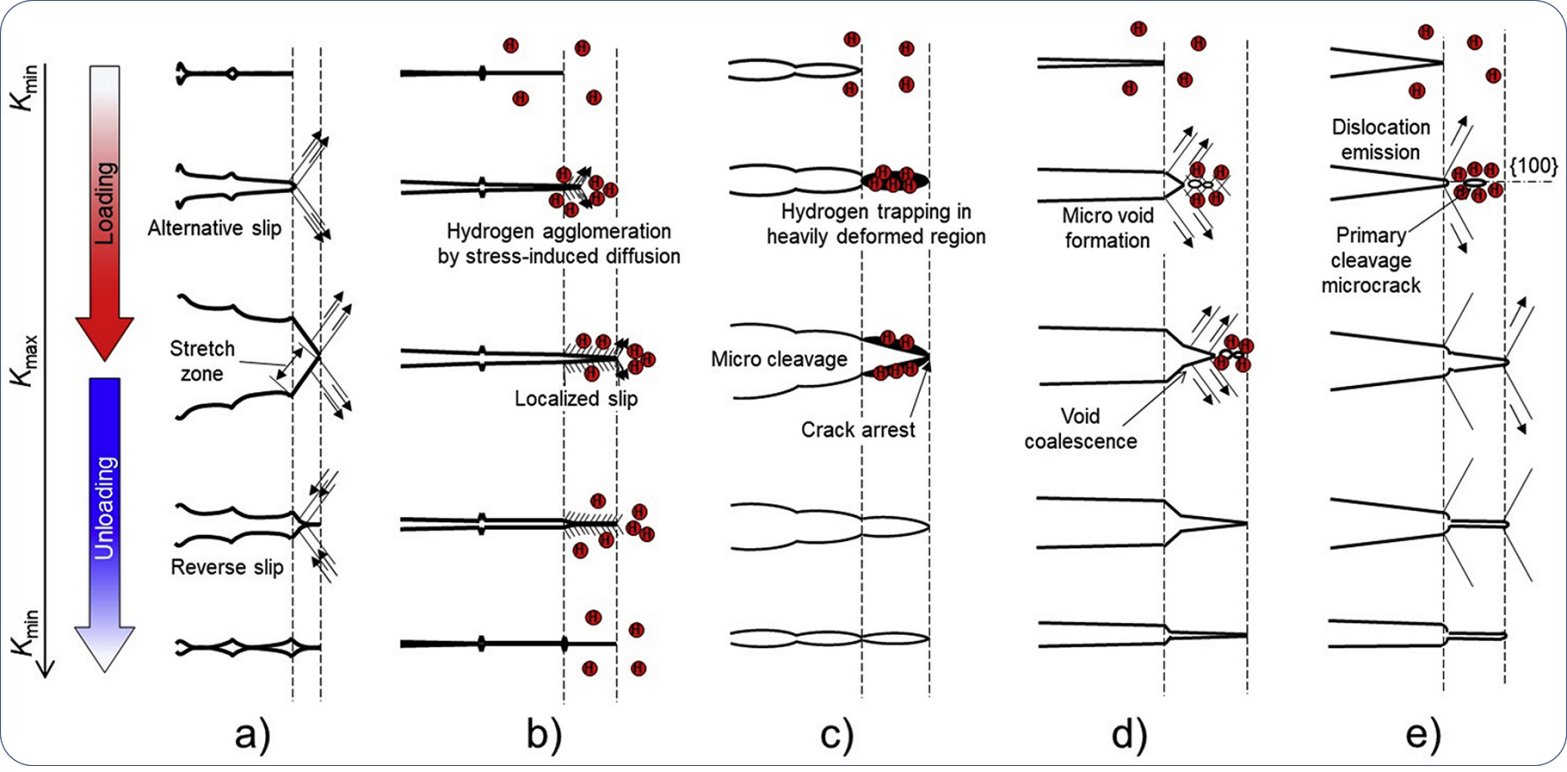
Models of H-accelerated FCG mechanisms.^779^ (a) ductile tearing model in the absence of H; (b) The so-called HESFCG model;^782^ (c) trapped H-induced cleavage cracking model;^784^ (d) localized slip-induced micro-void ncleation model;^768^ (e) H-reduced dislocation mobility and cleavage cracking model.^779^ Reprinted with permission from ref (779). Copyright 2018 Elsevier.

To effectively predict H-assisted crack growth, it is essential to consider specific micro-mechanisms for both TG and IG FCG. In terms of TG FCG, an important feature is brittle-like striations on the fatigue fracture surface,^778^ which correlates with the FCG rate.^768^Figure 47 shows an example of H-related brittle-like striations on a fatigue fracture surface. In a recent paper, Birenis et al.^779^ reviewed models based on these striations, as schematically illustrated in Figure 48. In air, FCG occurs through dislocation emissions, crack tip blunting, and resharpening, proceeding in a cycle-by-cycle manner,^780,781^ as illustrated in Figure 48a. H-accelerated TG FCG follows a similar pattern, evidenced by the striation spacing matching the FCG rate. The H Enhanced Successive FCG (HESFCG) model^782^ suggests that localized H atoms facilitate dislocation emission, leading to localized plastic deformation and reduced crack tip blunting (Figure 48b). A critical aspect of understanding the HESFCG model is the relationship between crack growth rate and dislocation emission. According to Pokluda,^783^ the ideal crack growth rate is determined by a geometrical relationship involving the crack tip opening and the number of dislocations emitted from the crack tip. Notably, the crack growth rate is independent of the plastic zone size in this model, that is, only dislocation emission from the crack tip is considered, in line with the AIDE mechanism. In practical scenarios, dislocations can also emerge from positions slightly behind the crack tip, contributing to the crack tip opening.

Another perspective, shown in Figure 48c, attributes the acceleration of FCG to H-induced local cleavage cracking.^784^ This involves crack tip blunting followed by a hydrostatic stress gradient and high dislocation density, with HELP possibly aiding the increase in dislocations. Thus, H localizes at the crack tip region through stress-driven diffusion and H trap at dislocations. The combination of the high local stress and high H concentration triggers HEDE on the cleavage plane, thus accelerating FCG. The third model^768^ shown in Figure 48d involves a combination of crack tip opening, micro-void nucleation ahead of the crack tip driven by localized slip, and ductile crack growth through void coalescence, which ties into the HELP and HESIV mechanisms. In addition, the model illustrated in Figure 48e focuses on the role of H in reducing the mobility of screw dislocations. This reduction in dislocation mobility hinders plastic stress accommodation, increasing tensile stress on the cleavage plane, particularly at high Δ*K*, leading to cleavage cracking. These various models, each highlighting different aspects and contributing factors, are collectively detailed in Table 5. They provide a comprehensive understanding of the mechanisms underpinning H-assisted FCG, emphasizing the complexity and multifaceted nature of this phenomenon.

**Table 5:** Summary of the TG H-Accelerated Crack Growth Models

Model	Figure 48b	Figure 48c	Figure 48d	Figure 48e
Critical process for acceleration	Enhanced crack tip plasticity	Cleavage	Micro-void formation	Cleavage
Main H effect	HELP and/or AIDE	HELP and HEDE	HELP and HESIV	Dislocation locking and HEDE
Crystallographic crack surface plane	{100}^a^	{100}	No specific crystallographic plane^785^	{100}

a In the case that the ideally symmetrical dislocation emission at the crack tip occurs on two {110} slip planes.

##### 2.4.3.5 Intergranular FCG

In the case of IG FCG, it is crucial to consider multi-scale H localization near the crack tip and GBs, the evolution of internal stress driven by dislocations, and decohesion-related cracking at GBs. Specifically in tempered martensitic steels, where H-related IG crack growth is common, an IG crack often initiates ahead of the main fatigue crack. The subsequent coalescence of the main crack with these smaller cracks leads to accelerated FCG. Thus, understanding the crack initiation mechanism near the main crack tip is critical for comprehending the micro-mechanism of H-accelerated IG FCG. According to Chen at al.,^786^ while large-scale plasticity evolution near intergranularly cracked regions is absent on a micrometer scale, minor and nano-scale dislocation motion may still contribute. Therefore, a key effect of H is the reduction in cohesive strength at GBs. The decohesion process is influenced by the maximum principal stress, acting as the driving force for crack growth, and the H content near the crack tip for each loading cycle. In a constant Δ*K* scenario, like in CT testing, both the stress level and the duration of each cycle, which allows H diffusion toward the crack tip region, are significant. These factors contribute to the observed substantial frequency dependence of the H-related FCG rate involving IG cracking. This emphasizes that H-accelerated IG FCG is not solely a function of H presence but also depends on the dynamic interactions of stress, H diffusion, and material properties within the microstructure, particularly at and around GBs. Understanding these interactions is essential for effectively addressing and predicting H-related FCG involving IG cracking.

##### 2.4.3.6 Effect of Crystallographic Structure

**Figure 49:**
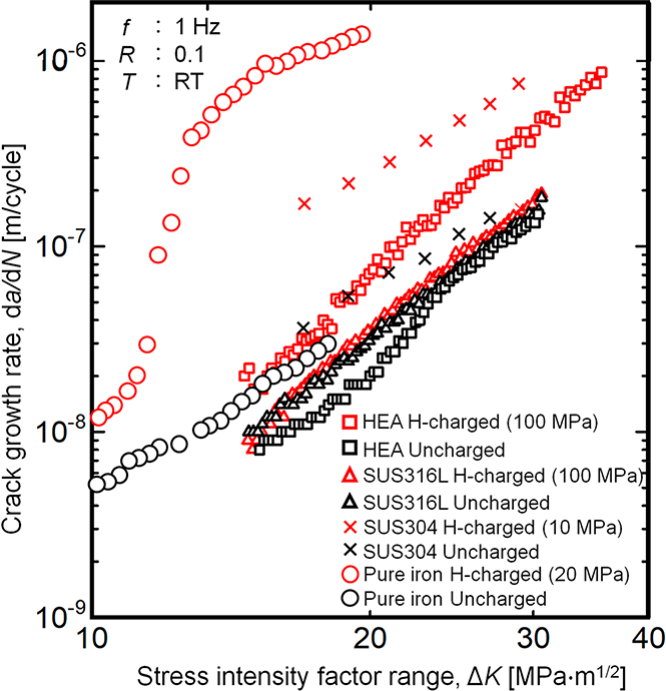
FCG rates plotted against stress intensity factor range in various materials. Reprinted with permission from ref (789). Copyright 2022 Elsevier. HEA: CoCrFeMnNi HEA. The values in brackets indicate H pressure. The data of stainless steels and *bcc* pure iron were extracted from refs (473 and 775).

An important stage in the mechanisms discussed earlier is H accumulation at the crack tip, driven by stress-induced H diffusion towards areas of maximum hydrostatic stress.^220,222^ In other words, the resistance to H-related FCG can be improved by suppressing H diffusion to hydrostatic stress concentration regions at the crack tip. An effective approach to suppressing the H diffusion is changing the crystal structures from *bcc* (e.g., martensitic and ferritic steels discussed above) to *fcc* lattice (typical of austenitic steels and Ni alloys). FeCrNi austenitic stainless steels like SUS316L,^787^ Fe-Mn twinning-induced plasticity steels,^788^ and CoCrFeMnNi HEA,^789^ with stable *fcc* phases, show markedly better FCG resistance in H environments than *bcc* steels (Figure 49). However, resistance decreases when the *fcc* phase is unstable or when precipitates are present. For example, in austenitic steels, the *fcc* austenite can transform to *bcc* martensite during fatigue loading, which accelerates crack growth under H environments, as *bcc* martensite provides a preferential H diffusion path.^787,790^ After pre-charging in 100 MPa H gas, H-related time-dependent FCG in the range of Δ*K* > 15 MPa m^1/2^ was observed in a nickel superalloy, which was attributed to the presence of δ-Ni_3_Nb precipitates at GBs triggering H-related cracking at δ-phase and γ-matrix/δ interfaces.^791^ Furthermore, γ′′-Ni_3_Nb and γ′-Ni_3_(Al, Ti) precipitates in Alloy 718 result in planar persistent slip on {111} planes due to precipitate shearing, which assists TG crack growth on {111} planes as well as IG crack growth.^792^ Hence, a stable single-phase *fcc* microstructure is essential for optimal resistance to H-related FCG. Another potential pathway for designing H-resistant alloys is employing *hcp* structure. According to an *ab initio* calculation, the H diffusivity of *hcp* phase in iron is lower than that of *fcc* phase.^162^ FeMn alloys, undergoing *fcc*–*hcp* martensitic transformation at the crack tip, did not show acceleration of FCG at room temperature in 0.7 MPa H gas.^793,794^

##### 2.4.3.7 Predictive Models: Physical Completeness vs Calculation Cost

From a predictive modeling perspective, early fracture mechanics-based models for cycle-dependent H-accelerated FCG focused on factors like H concentration and penetration distance from the crack tip, along with yield strength, but mechanisms of H–dislocation interaction, hydrostatic stress-driven H diffusion, and H trapping, were not sufficiently incorporated.^795^ Subsequent models have integrated stress and plasticity effects on H distribution and their influence on elasto-plastic deformation.^220,796^ Cohesive zone models were coupled with H diffusion analysis^797^ and trapping kinetics,^798^ capable of simulating the frequency dependence of H-related FCG rate. As the relationship between H and plasticity becomes more intricate, dislocation-based plasticity models, particularly those using a crystal plasticity approach coupled with H kinetics, are being explored.^799,800^ These models account for vacancy generation and dislocation-mediated H transport, moving toward a more comprehensive simulation of H-accelerated FCG.

However, due to the long-term nature of H-related fatigue involving complex phenomena like crack tip plasticity evolution, microstructure-dependent crack growth, and H diffusion/transport kinetics, a complex physics-based model can be computationally expensive. Therefore, simplified models with sufficient accuracy are valuable. Despite ongoing debates about the underlying HE mechanisms, one-dimensional dislocation-based models have shown potential in quantitatively simulating FCG.^801,802^ These models reduce three-dimensional dislocation motion and associated crack growth to a one-dimensional description, allowing accurate fatigue life predictions with lower computational costs.^803^ Incorporating additional factors like H diffusion and transport kinetics into these models could further enhance their predictive capability for fatigue properties in H environments.

##### 2.4.3.8 Key Challenges and Active Research Area

In summary, classification of H-accelerated FCG modes is important for clarifying the underlying mechanism. These modes are categorized as “cycle-dependent or time-dependent” and “TG or IG” based on the target condition. This categorization facilitates an in-depth analysis of H-related factors, including the impact of H content, test frequency, oxygen concentration, and the influence of H on cohesive strength.

In terms of material selection in structural design, the critical factors are resistance to H-related crack growth and simpleness of crack growth behavior. To obtain maximum resistance to H-related crack growth, stable austenitic stainless steel is the most promising material group. Therefore, the phase stability of *fcc* phase can be a criterion for estimating resistance to H-related FCG like the case for tensile properties. Furthermore, even when the *fcc* phase of the H-uncharged alloy is unstable, H can stabilize the *fcc* phase under cyclic loading condition.^205^ Therefore, a use of H as an alloying element may expand available material groups for cyclic loading under H environment. When considering strength and cost, tempered martensitic steel is another candidate. In this case, a material with high-strength and frequency independence (i.e. displaying cycle dependent crack growth) is preferable for the ease of structure design. Hence, improving the critical tensile strength for the transition from cycle- to frequency-dependent crack growth can be the next trend for development of H-compatible materials. Another aspect gaining attention is the effect of H blended in natural gas.^27,28,804^ FCG is still accelerated with a blend of the gases, while the degree of acceleration is manageable by regulating gas pressure and H content in the blend. Hence, the assessment and optimization of these factors in blended gas usage are important subjects of H-related fatigue research.

#### 2.4.4 Engineering Transferability of Testing Methods

HE is characterized by the reduction of mechanical properties across various testing methodologies. These methods are crucial for quantifying the extent of HE, understanding the underlying physical phenomena during H-assisted fracture, and aiding in the design and material selection for components in H environments.

Testing methods for HE encompass both standardized mechanical tests, as per ASTM and ISO guidelines, and alternative testing approaches. A key aspect of these methods is the distinction between screening approaches, which are more preliminary and broad, and design or parameter identification methods, which are more detailed and specific. In addition, acceleration strategies are implemented in these tests to simulate the effects of H over a component’s service life within feasible laboratory durations.

The procedures typically involve testing both smooth and notched specimens, as well as using fracture mechanics-based methods with pre-cracked samples. This dual approach reflects different philosophies within the framework of screening materials for compatibility with H environments. It is important to recognize that the development of H testing protocols is often based on established practices in the fields of corrosion and environment-assisted cracking, adapting them to the specific challenges posed by HE.

##### 2.4.4.1 Standard Testing

Mechanical testing for both tensile smooth and pre-cracked specimens is summarized following the common ASTM and ISO standards for both monotonic and cyclic loading. A detailed discussion on advantages and limitations of different monotonic tests for HE can be found in Dieztel et al.^805^ The focus here is put on the suitability of each method for determining HE susceptibility, i.e., the sensitivity to H-induced reduction in mechanical properties, and the elucidation of the operating mechanisms. These testing procedures can also be adopted to evaluate HE at cryogenic or at high temperatures, but the discussion is limited to room temperature.

**Figure 50:**
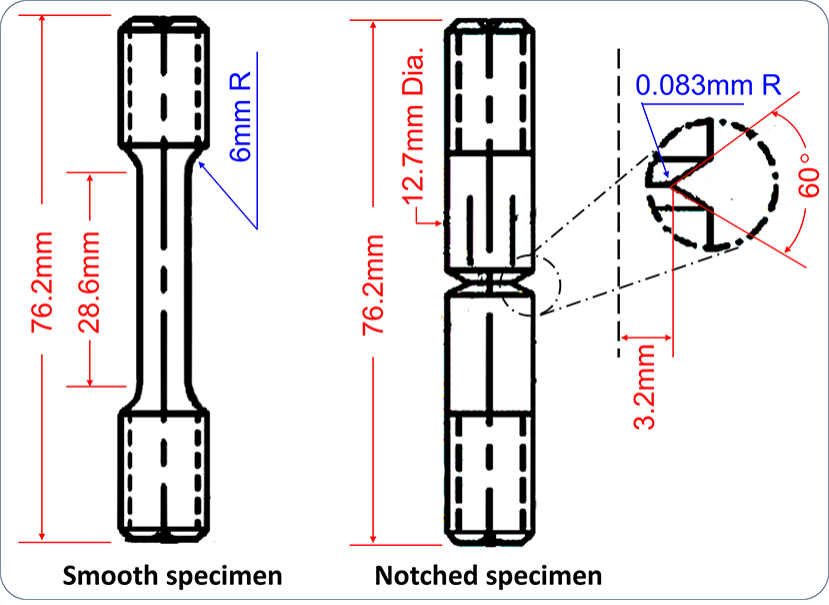
Specimens for SSRT H embrittlement testing. Redrawn based on ASTM G142 with permission. Copyright 2011 ASTM.

*Smooth and Notched Tests*. A direct application of monotonic tensile testing was developed for stress corrosion cracking studies in the 1960s and resulted in one of the most popular method to characterize environment-assisted cracking: SSRT test, also called Constant Extension Rate Test (CERT).^806^ The general standard for environmentally assisted cracking is covered by the ASTM G129 and the adoption of this procedure for HE in gaseous H_2_ is found in ASTM G142 for round specimens, both smooth and notched samples, as illustrated in Figure 50.

The ASTM G142 standard does not cover small size or flat specimens and therefore its application to very low diffusivity alloys, e.g., austenitic stainless steels or nickel alloys, requires dimension reduction and/or high-temperature pre-charging.

As shown in Figure 50, the notch considered in ATM G142 has a 60° V-shape with a notch tip radius of 0.083 mm, producing stress concentration factor *K_t_* around 5.6 to 6.6,^807,808^ which reveals an embrittlement susceptibility that is not observed in smooth samples. Different notch radius or configurations can be tested to evaluate the influence of triaxiality; there is consensus that high *K_t_* values result in an increase in H concentration near the notch tip and therefore in a higher susceptibility to HE.^807^ The notched geometries from ASTM G142 have also been used to assess fatigue properties in gaseous H_2_ environments.^809^ Reduction of notched tensile strength (RNTS) or relative reduction of area (RRA) is also considered as criteria for materials qualification according to the ANSI/CSA CHMC 1-2014 Standard (Test methods for evaluating material compatibility in compressed H applications - Metals). This code specifies different testing procedure including tensile testing, fracture toughness, FCG rate, or S-N fatigue curves but warns that results of these tests are intended to provide a basic comparison of materials performance and that additional testing considerations may be necessary to fully qualify the design. Differences in testing procedures from ANSI/CSA CHMC 1-2014 and ASTM G142, ASTM E1820 or ASTM E847 are discussed in the comprehensive review about fatigue-based codes and material testing for gaseous H by Fischer et al.^810^

The limitation of SSRT testing typically pointed out in the literature is that the results are dependent on displacement rate or on the specimen size, which is attributed to an inadequate measurement or monitoring. It should be noted that diffusion-controlled embrittlement can explain all these dependencies and sufficiently slow rates should correspond to the most critical concentration conditions. However, the appearance of sub-critical crack growth has also been identified as a source of uncertainty in SSRT since the remote stress does not represent the mechanical driving force triggering embrittlement. Martínez-Pañeda et al.^811^ proved numerically and experimentally that this limitation results in a disparity between the observed embrittlement depth and the expected H diffusion distance, and that this limitation is more critical in notched tests. Estimation of environment-induced cracking areas and the corresponding stress intensity factors is a possible way to improve interpretation of SSRT tests,^812^ but some authors claim that SSRT is only useful as a screening tool to assess H susceptibility but not for a reliable life prediction.^813^

As for stress corrosion cracking, constant displacement tests on smooth samples can be used to determine the threshold stress for cracking, after which stress relaxation might take place. The total time to failure is the other parameter that can be determined. Similarly, constant load testing at different stress levels can be used to estimate the stress threshold. Both tests can be carried out uniaxially or applying bending stresses, including U- or C-shaped specimens. Stress corrosion testing in different configurations is covered by the different parts in ISO 7539. The main advantage of constant load or displacement testing is the simplicity of the equipment in comparison to rising load testing machines; however, testing times can be excessive in comparison to SSRT.

The ISO 16573 standard deals with HE of high strength steels and includes both constant loading (Part 1) and SSRT (Part 2) procedures. This Standard normalized a pre-charging method and an H continuous charging method, considering cathodic charging, aqueous solutions at free corrosion potential, atmospheric corrosion environments or high-pressure H_2_. For pre-charged samples, this standard also proposes plating to suppress H effusion during *ex situ* testing. In contrast to other standards, the ISO 16753 covers post-test specimen treatment and analysis of diffusible H, especially by thermal desorption.

**Figure 51:**
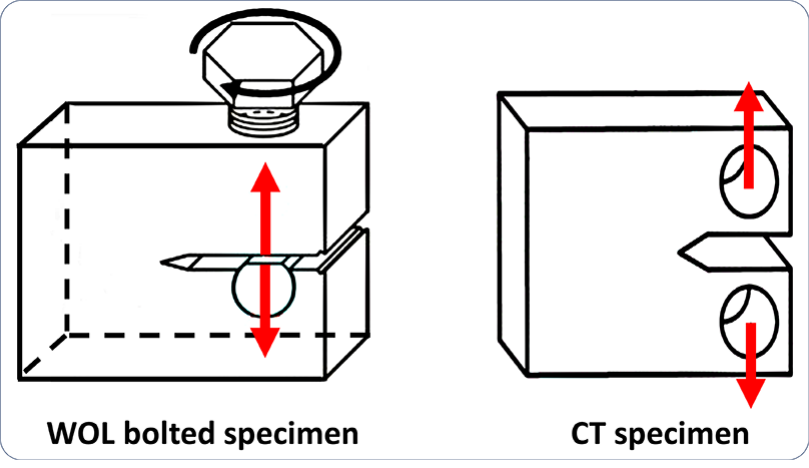
Schematic plots of wedge opening load and CT specimens for sustained and rising loading, respectively.

*Fracture Mechanics Tests*. Due to the simplicity of experimental setups for constant displacement or constant load testing, these approaches are also widely used considering pre-cracked specimens. Sustained bolted loads, as shown in Figure 51 for a wedge opening load (WOL) specimen, are addressed by the standards ASTM E1681 and ISO 7539-6, which are based on the determination of threshold stress intensity factors, *K*_EAC_ or *K*_th_. The long duration is the main limitation of these tests, but it must also be considered that the analysis is based on linear elastic fracture mechanics (LEFM) and small-scale yielding is assumed; therefore, thickness and specimen size need to be large in comparison to the plastic zone, which also limits the applicability for some ductile materials. In addition, plastic deformation can be a prerequisite for some environment assisted cracking and therefore constant load or constant displacement procedures can yield different results than dynamic testing.^814^

Rising load or rising displacement tests overcome the problem of excessively long testing times, but as a consequence the influence of testing rates must be assessed due to the required H diffusion to the fracture process zone. An intermediate alternative can be found in the incremental step loading test covered by the ASTM F1624, in which the load is maintained in discrete intervals until fracture occurs. This standard can be applied to many sample configurations, including smooth, pre-cracked specimens, fasteners or small punch samples.^815^

The extension of fracture mechanics standards, as ASTM E399 or ASTM E1820 for monotonic rising loading or ASTM E649 for fatigue testing, to gaseous HE requires the coupling of a high-pressure autoclave to a universal testing machine but it is not covered explicitly by any standard. Some authors have developed an apparatus to test multiple CT specimens in series, considerably reducing testing time.^804,816^

The design codes and standards of components for H storage or transport include material qualification strategies. For pipelines, the ASME B31.12 document consider two approaches: (i) a prescriptive design method without specific H testing but assuming a material performance factor to increase the design thickness, or (ii) to perform fracture testing in H environments according to ASME BPVC VIII.3 KD10, which is followed for H_2_ pressure vessels and considers the ASTM E1681 testing procedure for material qualification. Risk-based inspection standards, e.g., API RP 581, ASME PCC-3 or EN 1688, consider some H-induced degradation phenomena for the risk assessment; however, the determination of damage factors due to HE is not clearly defined and the inspection planning procedures are yet not optimized for components handling pressurized gaseous H.^817^

Regarding qualification of materials for components to operate under gaseous H_2_, the ISO 11114-4 standard is a relevant document for HE testing for material selection in transportable gas cylinders and includes three testing methods: Method A, Rupture Disc; Method B, rising-step-load for a CT specimen; and Method C, constant displacement test for a wedge opening load specimen. Fracture mechanics procedures, method B and C, are comparable to the previously mentioned standards. In contrast, the rupture disk method is a simpler alternative, also standardized by the ASTM F1459, where a membrane is submitted to H or helium pressure until burst. The susceptibility to HE is quantified by the ratio of burst pressures in He and H_2_. Modifications of the disk pressure test including thinning of the specimen center or circumferential notch to assess HE under different triaxiality states are under development.

##### 2.4.4.2 Alternative Testing

Non-standard testing procedures have been proposed as cost-effective alternative screening methods for HE susceptibility. For example, to circumvent the need of an autoclave containing a high volume of gaseous H_2_ at high pressure, hollow specimens have been proposed as a safer and simpler alternative. This procedure is based on a tubular specimen exposed to high-pressure H in the inner machined hole; it was firstly proposed by Chandler and Walter^818^ and has been also adopted by other authors.^819,820^ The comparison with conventional tensile specimens can be hindered by the inner surface roughness^821^ and by the different necking mechanisms during ductile failure.^748,822^

**Figure 52:**
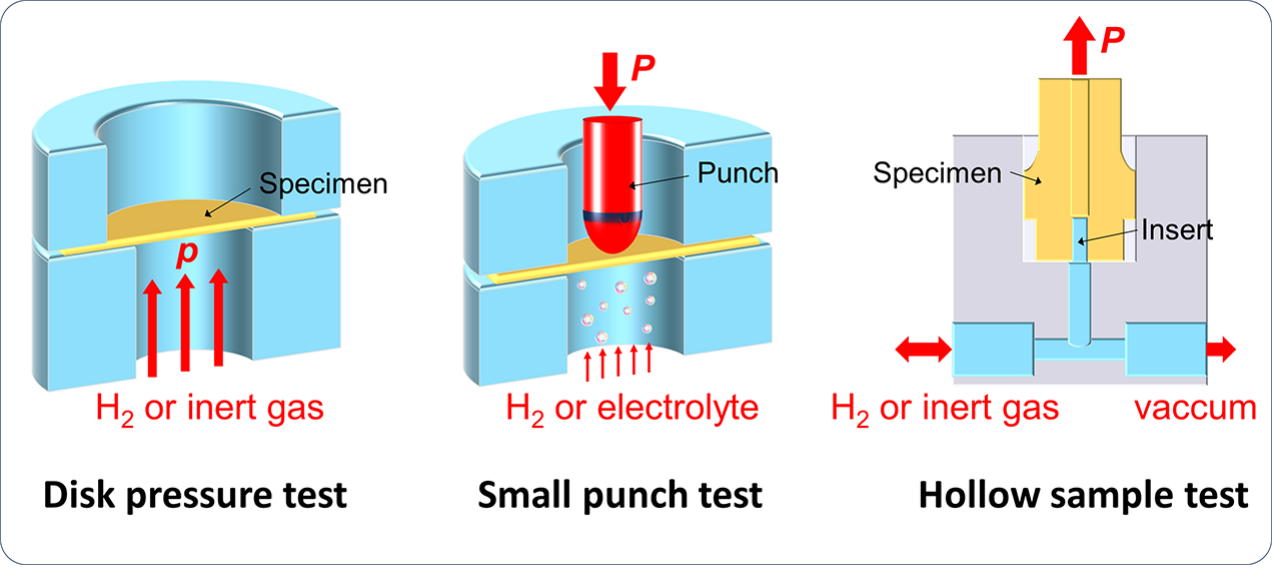
Scheme of experimental setups for the disk pressure test, the SPT and the hollow sample test. The disk pressure test is a standard screening method whereas SPT and hollow specimens are still considered alternative procedures to study HE.

The small punch test (SPT), which is commonly used as a screening tool for nuclear materials, requires a simple procedure to test miniature specimens, 10 × 10 × 0.5 mm^3^ approximately, and it has been recently standardized (ASTM E3205-20 or BS EN 10371:2021) for metallic materials. The application of SPT to the study of HE has been considered for pre-charged specimens,^823^ electrochemical *in situ* testing,^824,825^ or even with gaseous *in situ* setups.^826,827^ An illustration of the SPT setup is shown in Figure 52, where the loading similarities with the disk pressure test can be appreciated. The hollow specimen test is also described in Figure 52. Notched SPT samples have also been demonstrated to enhance H susceptibility due to the higher hydrostatic stress values.^828^ The extension of SPT to incremental step loading to determine threshold loads for HE has also been proposed based on the ASTM F1624.^829^ It must be noted that smaller specimen sizes are not here revisited and nanomechanical testing is discussed in Section 2.3.4.

##### 2.4.4.3 Screening Tests Vs Parameter Identifications

Following the definitions from environment assisted cracking literature,^814,830^ HE susceptibility can be assessed from different perspectives. A possible classification of testing approaches: (i) screening tests (“Go/No-Go” criterion, susceptibility classification, relative aggressivity of environments), (ii) design criteria or parameter identification, and (iii) investigation of mechanisms.

The distinction between screening methods and design criteria in HE testing is related to the purpose and level of confidence associated with each type of test. Screening methods are typically used in the early stages of material selection or during the development process. They aim to quickly identify materials that are highly susceptible to HE, allowing for their elimination from further consideration. Screening methods are relatively simple, cost-effective, and provide a preliminary assessment of a material susceptibility to embrittlement. If the material exhibits significant embrittlement or failure in these tests, it is flagged as potentially unsuitable for the intended application. They are not intended to provide definitive design criteria but rather serve as a cost-effective initial filter. For these reasons, screening testing usually considers: (i) smooth samples without pre-cracking, (ii) accelerated rates in comparison to service life, and (iii) simple loading configurations. Dietzel et al.^805^ point out that smooth specimen testing is essentially used as a screening method since real components are not free of defects. An extensive discussion between safe-life or damage-tolerance approaches in the context of HE testing is out of the scope of the present Review. As already mentioned, SPT has also been considered as a reproducible and consistent screening method where embrittlement can be analyzed.^831^ Screening the susceptibility of different materials to HE environments is usually addressed using the engineering concept of embrittlement index.

On the other hand, tests oriented to obtain design parameters for components to work in H environments are more rigorous and provide a higher level of confidence in evaluating a material resistance to HE. For material qualification, these tests are performed on selected materials that have passed the screening phase and are being considered for critical applications where failure due to embrittlement would have severe consequences. In contrast to the screening approaches, design-oriented testing requires conditions that simulate as closely as possible operating conditions. When this is not possible, physics-based analysis should be considered to ensure transferability or mimic worst case scenarios, e.g., pre-cracked specimens, plane strain conditions or loading rates that result in a maximum H concentration. The results from these tests are used to establish design guidelines, such as maximum allowable H concentrations, load thresholds, service life predictions, or inspection intervals. For these reasons, design-oriented testing within the HE context requires pre-cracked specimens and approaches from fracture mechanics.

It must be noted that some test results might be used both as screening methods and for design criteria, depending on the level of analysis and on the complimentary experimental or numerical analysis. For example, extrapolation of accelerated HE tests to real service life can be assisted by the determination of diffusion properties, e.g., permeation, and by the concentration measurement, using hot extraction or TDS, of samples exposed to equivalent charging conditions. Similarly, embrittlement indices can be translated into a local reduction of the critical energy release rate by using appropriate modeling that couples H accumulation with damage. On the other hand, some tests can be quantitatively used as design criteria when they are performed *in situ*, i.e., under H_2_ simultaneous exposure, while they can only be used to qualitatively classify susceptibility when carried out *ex situ* after H pre-charging. Limitations in the determination of design parameters using aqueous charging media must also be addressed and enriched by a deeper understanding of the correlation between electrochemical and gaseous uptake, as discussed in Section 2.2.1.5.

## 3 Perspectives and Future Directions

In the previous section, we thoroughly examined the existing knowledge surrounding HE, identifying key challenges and unresolved questions across various aspects of HE research. Building on this groundwork, this section will shift the focus toward potential research avenues and strategies aimed at addressing these challenges. The section will start by outlining the essential knowledge needed to accurately model and treat H entry, diffusion, and trapping within materials. Additionally, it will discuss potential trends in the exploration of H–metal interaction mechanisms. Further with innovative ideas on mitigation strategies, it aims to pave the way for developing materials that are more resistant to HE. A forward-looking element of this section will be the introduction of promising physics-based predictive models and of emerging ML tools for assessing HE. Although these concepts are still in the developmental phase and require further refinement, they represent exciting prospects for future research endeavors in predicting and preventing H-induced material failures.

### 3.1 Knowledge Needs for Determining H Content

Despite the progress made in theoretical frameworks and recent advancements in experimental techniques, there remains a substantial gap in our understanding of H uptake and H transport within materials. This gap poses a challenge in accurately quantifying H content in various materials. The complexity of H behavior in different material matrices, ranging from absorption and adsorption processes to diffusion and trapping mechanisms, necessitates advanced analytical techniques and sophisticated modeling approaches. Addressing these knowledge needs is crucial for understanding and developing more effective strategies against HE.

#### 3.1.1 Needs for Improved Understanding of H Uptake

The assumption that transient effects in HE are predominantly governed by diffusion to the fracture process zone has been questioned, especially for cases with external H,^832^ highlighting the importance of surface-limiting phenomena. Lynch^32^ showed some similitudes between HE and liquid metal embrittlement, where the surface interaction with the embrittling species is the limiting step. Similarly, Turnbull et al.^108^ showed that crack growth rates are limited by surface kinetics. Nevertheless, there is a considerable need for further understanding of H-surface interactions, necessitating advancements in both experimental and computational domains.^833^ The development of more repeatable measurement methods for H adsorption is crucial. This includes improvements in both manometric and gravimetric techniques,^834^ to enhance accuracy and reliability in quantifying H interactions with material surfaces.

Research in the fields of H storage materials and catalysis has pinpointed and addressed several challenges associated with H adsorption on metal surfaces. A critical issue in studying H adsorption using ab initio methods is the inherent inaccuracy in calculating weak interactions,^835^ and accommodating complex environmental conditions within these computational models remains a challenging task.^836^ Nonetheless, materials proposed for H storage often have intricate compositions, and insights gained about H adsorption in these contexts may not be directly applicable to the surfaces of engineering alloys targeted by this Review.

Despite the basic tools are already available to consider kinetic rate expression as well as equilibrium isotherms for adsorption processes,^837^ continuum modeling frameworks must incorporate them into proper boundary conditions. This is especially important for H-assisted cracking from electrochemical charging, since the electrolyte system is governed by coupled equations of fluid motion and electrochemistry, that invalidate the assumption of constant and uniform conditions.^838^ In addition, *ab initio* approaches for electrochemical approaches still show important computational limitations.^839^

Continuum modeling frameworks for H accumulation near a crack tip already need to consider kinetic boundary conditions or, at least, verify the validity of constant concentration assumptions. In the context of HE, two main factors are considered as necessary to accurately predict H uptake from the crack surface:

- Generalized entry fluxes derived from adsorption theory, i.e., driven by the imbalance between surface coverage and subsurface concentrations. To this end, Langmuir-based kinetic reactions for H_2_ dissociative chemisorption must be considered or, for electrochemical equivalent charging, kinetics from the H evolution reaction. The consideration of adsorption rates out of equilibrium is not only useful to account for transient effects but also facilitates the study of influencing factors on different sticking coefficients or adsorption/absorption constants. Additionally, this approach would contribute to a better understanding of permeation and TDS characterization since the validity of simplified boundary conditions could be verified and its applicability could be extended.
- Stress effects on H uptake. Even though thermodynamic arguments clearly indicate the need of stress-dependent boundary conditions on a crack tip surface,^104^ many numerical approaches still assume constant concentrations independent of stress. This might be caused by the difficult implementation of a stress-dependent concentration at nodes in finite element frameworks and could be overcome by the consideration of chemical potential as the dependent variable of diffusion equations. Coupling H uptake to damage modeling during crack propagation also requires a moving boundary condition, e.g., considering penalty methods,^690^ that does not usually take into account stress effects. In addition, the experimental verification of stress-dependent uptake must be validated, especially to assess the possible influence of non-hydrostatic stress terms. It must also be noted that some theories predict stress values at a crack tip that are orders of magnitude higher than for classical plasticity.^690^ An experimental verification of crack tip surface stresses and concentrations would be then a ground-breaking advance for the understanding of H–surface interactions.

Conditions of a real H–metal system must deviate from an ideal situation where a defect-free and impurity-free surface is assumed. These effects have been rarely addressed but are now increasingly considered in the context of mitigation measurements for HE: the use of barriers or inhibitors to reduce or suppress H uptake in metals will be fostered by a better understanding of H_2_ dissociative chemisorption and subsurface transport. In addition, the analysis of surface roughness influence on H uptake is not straightforward, because roughness does not only increase the effective area,^593^ but machining procedures can produce a change in dislocation density and microstructure.^840^

#### 3.1.2 Needs for Better Understanding of H Transport

The understanding of H transport mechanisms at both atomic and continuum scales remains incomplete and is intrinsically linked to the phenomena of time-dependent embrittlement. As elucidated earlier, distinguishing the impact of internal versus external H on mechanical properties necessitates consideration of trapping effects. However, a definitive delineation of the H population (diffusive versus trapped) that exacerbates HE remains lacking. Three critical areas have been identified needing intensive research effort: (i) the characterization of trapping mechanisms, (ii) non-lattice H transport pathways, and (iii) H migration through hierarchical or anisotropic microstructures.

##### 3.1.2.1 Challenges in Trapping Characterization

The two most common methods to characterize trapping influence on H transport are permeation test and TDS. As previously discussed, both still rely on simplified fitting procedures whose validity should be verified for some conditions. In the case of permeation modeling, the concept of apparent diffusivity as a material property should be abandoned when trapping effects are significant while consistent procedures to fit trap densities and binding energies need to be adopted. Further understanding of H adsorption kinetics for different electrochemical charging conditions is also required. Similarly, limitation of Kissinger’s detrapping-based models for TDS interpretation must be generalized by including diffusion-controlled conditions and the influence of non-instantaneous desorption and non-uniform initial distributions. For both methods, permeation and TDS, advanced regression techniques based on ML paradigms would have an enormous impact on trapping characterization.

Moreover, many modeling works extract from literature the relationship between plastic deformation and trap density, which is crucial for H accumulation near a loaded crack tip. The extensive use of Kumnick and Johnson’s^245^ relationship for iron is useful for benchmarking purposes, but this extrapolation to different alloys or microstructures cannot be assumed and each material condition should be characterized.

To incorporate trapping effects in diffusion modeling, discrete level approaches, i.e., partition of H species into trapping and lattice sites, are mostly considered due to the impact of the seminar works of McNabb and Foster,^244^ Oriani,^221^ and Sofronis and McMeeking,^220^ but there is a lack of a critical assessment of the limitations of trapping averaging into a finite number of defect types. The number of particular trapping sites to be modelled is a subjective choice that sometimes is based on qualitative microstructural observations or on TDS spectra that are difficult to interpret. Therefore, the relatively unexplored modeling frameworks that consider a continuum distribution of binding energies,^841^ should be revisited to address possible limitations of discrete energies and densities.

##### 3.1.2.2 Challenges in Non-lattice Transport Mechanisms

H transport is usually assumed to be controlled by lattice diffusion that is delayed by trapping effects. This simplification is based on the assumption that traps are isolated and immobile, but these are invalid in two main cases: for traps forming networks and for traps motion during straining. The first case is relevant for H transport through GBs, which has significance for nickel-based alloys. Fast diffusion dominates over trapping when the segregation energies are high but the migration energies are low.^842^ However, it still remains unclear for steels, where a different behavior is expected for *fcc* or *bcc* phases.^843^ Mogilny et al.^844^ have criticized GB acceleration interpretations, especially for electrochemical charging, and attributed fast diffusion to an increased flux by sliding dislocations.

On the other hand, H transport by dislocations is sometimes proposed as a mechanism that could explain the difference between observed and predicted embrittled regions,^845^ but it is difficult to measure and simulate. Some authors have used microprinting techniques to demonstrate H transport by dislocations in a Ni-Cr-Fe alloy^846^ or in an austenitic stainless steel,^847^ but novel experimental methods are required to confirm these findings. A mechanistic approach to model H transport by dislocations in continuum frameworks, as proposed by Dadfarnia et al.,^227^ has not been applied to explain experimental observations, and the cause might be that modeling dislocation velocity direction is not straightforward. Crystal plasticity approaches could be the key to understand these processes.^799^ A possible mechanism that is not commonly addressed but requires further understanding is H pipe diffusion along dislocation cores, which has been proved by DFT calculations for some metals. However, the process was regarded secondary in iron^848^ or at a macroscopic level.^849^ Similarly, vacancy-mediated diffusion is only important for hydride-forming systems^850^ or for heavier interstitial atoms.^851^

##### 3.1.2.3 Challenges in Transport through Hierarchical and Anisotropic Microstructures

Modeling and understanding of H diffusion through a multiphase microstructure has been already considered an outstanding challenge by Turnbull,^109^ due to the complexity of the involved phenomena: diffusivities between phases can be many orders of magnitude different, tortuosity and connectivity of phases critically impact the mesoscopic transport and trapping at the interfaces must also be considered. The examples of anisotropic diffusion resulting from multiphase microstructures are numerous, e.g., duplex stainless steels^852^ and ferritic–pearlitic steels,^853^ while diffusion through single grains also can present anisotropy.^271,854^ The interaction between mechanical phenomena and anisotropic H diffusion must also be addressed from crystal plasticity frameworks.^239^

Isotropic H diffusion is mostly assumed despite the importance of anisotropic and GB diffusion discussed above. Continuum level simulations that do not consider explicitly microstructural phases and grains require proper homogenization techniques, for instance, expressions based on effective medium approximations^855^ and modifications based on percolation theory.^236^ Additionally, microstructure is not static and strain-induced transformations should be considered when analyzing H transport. This is especially critical for austenitic stainless steels, where fast diffusion paths along strain-induced martensite are considered crucial in some embrittlement interpretations. The coupled simulation of strain-induced martensite formation and H accumulation near a crack tip has been only addressed in simplified frameworks.^856^ H trapping at hierarchical microstructures is also an open research field, gaining importance due to the rise of AM technologies. Some authors have proposed a diffusion enhancement through sub-grain cellular structures produced by SLM for 316L^585^ and for an HEA,^552^ but the generalization of these finding still remain unclear.^581^ Trapping at dislocation cells is also a topic worth of further investigation.^178,857^

### 3.2 Knowledge Needs for HE Mechanisms

We identify the following needs for future development of HE mechanisms, based on the historical overview of HE mechanisms presented earlier, including the controversies that persisted over decades, some consensus that was reached recently, and the questions yet to be answered.

#### 3.2.1 Unresolved Issues about HE Mechanisms

As detailed in Section 2.3, thanks to the development of experimental and numerical approaches, significant progress has been made in understanding the HE mechanisms in the past decades. Nevertheless, there are still many remaining fundamental questions. Some of the questions are exemplified here.

- How fracture is actually triggered as plasticity localizes in the presence of H. Current HE theories are predominantly based on post-mortem analyses, such as fractography and dislocation substructures. These analyses allow only speculative inferences about the events between the onset of plasticity localization and the eventual fracture. Direct, *in situ*, and time-resolved observation of crack initiation from areas of plasticity localization remains a significant challenge. A promising technique in this context is operando X-ray diffraction,^151,858^ but a great challenge with this technique is to pinpoint the local region where a crack is going to initiate. One potential solution to this problem is enhancing the technique to scan and record data across a larger volume of the specimen, thus increasing the likelihood of capturing the critical moment of crack initiation.
- Where exactly H resides inside a material. While it is accepted that a precipitate can act as a trap for H, it is debatable if the trapped H resides in the matrix close to the precipitate, exactly at the interface between the precipitate and the matrix, or inside the precipitate. Such information is important for an accurate interpretation of the mechanism and is necessary for developing appropriate HE mitigation measures through precipitate engineering.
- The controversial role that H plays in plasticity. As detailed in Section 2.3.2, H should facilitate the bow-out according to the Defactant concept, which can be simulated with MD assuming H atoms are bound to the dislocation core and do not segregate. However, the opposite effect of H is predicted if considering the different tendencies of H bonding to different sites along the dislocation line in an MD simulation. The debate persists largely because a standard way to set up an MD simulation has not been established, besides, MD simulation of the multiplication of several dislocation loops from an FR source coupled with H diffusion is still challenging. As the field of atomistic modeling continues to evolve and computational capabilities expand, this question as well as its related topics is expected to be answered.

#### 3.2.2 Boundary Conditions and Applicability of HE mechanisms

The inherent complexity of HE introduces significant challenges in exploring its fundamental mechanisms. It is generally agreed that a specific HE mechanism should apply if a combination of critical factors, such as material type, temperature, H and loading conditions, is uniquely defined. This mechanism may not be encapsulated by a singular theory but rather could represent the synergistic interaction of multiple theories, yet it remains deterministic in nature. Historically, research on HE predominantly concentrated on interactions between H and the microstructure, frequently attempting to correlate observed phenomena with a single or primary mechanism. Nevertheless, the past two decades have witnessed a growing acknowledgment of HE’s intricate complexity and the synergistic effects among various HE mechanisms, leading to more diversified research outcomes that are less deterministic. Given a set of material parameters, H concentration, and loading conditions, pinpointing the HE mechanism remains a formidable challenge, so many authors tended to provide a list of viable HE theories instead of a definitive answer. This pattern is evident in many recent HE studies, reflecting the current state of technology that, while advanced enough to uncover HE’s complexity, still falls short of revealing certain sophisticated aspects crucial to defining the underlying mechanism.

Future research is likely to focus on refining and categorizing existing HE theories. The aim will be to articulate these theories with greater precision and robustness, clearly defining their scope and applicability. As experimental and numerical techniques for studying HE continue to advance, the language and descriptions used in theoretical models are expected to contain less uncertainty. Consequently, the pursuit of a unified HE theory, while conceptually appealing, might not be the central objective of future studies. Instead, the emphasis may shift towards developing a more elaborate and comprehensive understanding of the various mechanisms involved in HE, tailored to specific conditions and materials.

#### 3.2.3 HE Database for Mechanism Mapping

As mentioned above, it should be possible to determine a unique underlying mechanism, given a combination of the factors influencing HE. For accurate prediction of H induced failure in engineering practice and effective mitigation measures against HE, it is necessary to do so. The exploding interest on green H has facilitated a rapidly growing database on HE, an important task is to analyze this diverse database and make the data converge to practical knowledge for engineering applications. A desirable outcome is a clear mapping between the existing HE mechanisms and the material, environmental, and loading conditions in engineering application, this will help clarify the range of applicability of a specific HE mechanism and importantly, pinpoint a mechanism given a specific engineering application. To achieve this, extensive, in-depth analysis and classification of the existing database, along with pattern recognition, are necessary: tasks that are likely beyond human capacity alone. In this direction, ML is expected to become an indispensable tool. Some thoughts regarding the application of ML in HE research will be presented in Section 3.5.

To enhance the classification and mapping of existing HE mechanisms, efforts must focus on rendering individual mechanisms more specific and quantifiable. Current HE theories comprehensively cover the interaction of H with a wide array of microstructural defects. However, the broad scope of these mechanisms often results in overlaps, making it challenging to distinguish between them. This ambiguity complicates the identification of the precise underlying mechanism for specific cases and poses difficulties in predictive modeling, which requires clearly defined mechanisms as its basis. Establishing a more explicit scope for each HE mechanism will aid in accurately interpreting the mechanism at play in particular instances. The process of narrowing down the scope of an HE mechanism does not diminish its validity. Given that it is widely recognized that multiple mechanisms can coexist and often work synergistically, there is no pressing need to seek a universal theory encompassing all aspects of HE.

#### 3.2.4 Needs for Further Development of HE Mechanisms

In addition to deepening the understanding of established HE mechanisms and enhancing their predictability, it may be beneficial to revisit some less emphasized HE mechanisms, previously considered secondary in failure initiation. This reconsideration is particularly relevant to the advent of green H applications. For instance, H-promoted cleavage, a significant research topic in the 1990s, observed experimentally at cryogenic temperatures, has received relatively less attention in recent decades. This decline in interest is partly because cleavage is rare at room or sub-zero temperatures, which are more relevant to conventional H applications like pipeline transport. However, as green H increasingly finds applications in the aviation sector, where cryogenic H storage is a critical aspect, revisiting the H-enhanced cleavage mechanism becomes necessary. Another area requiring renewed focus is the study of H-induced failures in turbine blades at elevated temperatures, a scenario relevant to H propulsion engines. Current studies rarely address high-temperature HE, often under the assumption that the high diffusivity of H at elevated temperatures prevents significant accumulation in metals. However, with emerging applications in sectors like aviation, a reevaluation of these less explored mechanisms is needed.

With the verification, improvement and reconsideration of existing HE mechanisms and the emergence of new applications, new theoretical insights into the HE mechanisms may naturally appear. When a new theory is proposed, it should be assessed with the same criteria mentioned above for existing mechanisms. While there is an inherent tendency to emphasize on the novelty of a mechanism, it would be helpful to clarify the similarity and relation, if any, of the new theory to existing HE mechanisms. A very good example in this regard is the H-enhanced and plasticity-mediated decohesion mechanism.^37,468^

#### 3.2.5 Knowledge Adaption to Innovative Material Manufacturing Technologies

The knowledge of HE must adapt and advance in response to innovative material manufacturing techniques like AM. The study of HE in the context of AM materials is an emerging field with much yet to be explored. The nature of AM, with its intense thermal gradients, quick cooling, and layer-by-layer building, creates unique microstructures like directional grains and cellular sub-grains, all of which profoundly influence HE susceptibility. Understanding on these aspects is indispensable in developing materials and processes that mitigate HE while leveraging the full potential of AM technologies.

One of the primary gaps in our understanding is the interaction between H and the unique microstructures of AMed materials. These materials often exhibit cellular structures and dislocation tangles that differ significantly from those produced by conventional methods. Understanding how H interacts with these structures, including its absorption, diffusion, and trapping at various sites, is crucial for predicting and preventing HE. Additionally, a comprehensive investigation into the influences of residual stresses and inherent defects, typical of AM, on HE susceptibility remains essential. These factors can significantly influence the initiation and propagation of cracks. Moreover, while some studies have begun to explore the role of printing orientation, layer interfaces, and other AM-specific variables on HE, there is still much to uncover. The anisotropic properties of materials produced via AM can lead to directional dependencies in mechanical properties and HE resistance, which need to be thoroughly understood and controlled.

Despite these challenges, the potential of AM to transform H applications is considerable. AM allows for unprecedented control over material microstructure, facilitating the optimization of properties beyond the capabilities of traditional manufacturing techniques. For example, through GB engineering, AM can potentially reduce initiation sites for H-assisted cracking by controlling the density and characteristics of high-angle GBs. Similarly, dislocation cell engineering can help mitigate the effects of H by optimizing the microstructure for reduced H diffusion and improved H trapping. At a larger scale, AM’s ability to create complex and customized geometries opens up new possibilities for designing H-resistant components. This could include optimized shapes that reduce stress concentration, or the integration of features that help to mitigate the effects of HE, such as barriers to crack propagation.

As AM technologies evolve, so do the microstructures they produce. A deeper understanding of these structures, including grain size, shape, orientation, and the distribution of defects, is crucial. Advanced characterization techniques such as electron microscopy, X-ray diffraction, and APT can provide the detailed insights needed to understand how these microstructures interact with H. Equally important is developing comprehensive models that can predict HE in AM materials, which is a significant challenge. These models must consider the complex and often anisotropic microstructures, as well as the dynamic conditions of AM processes. Combining empirical data with simulations at various scales will be critical in developing reliable models for this scenario. Exploring new ways to mitigate HE in AMed materials is another critical area of research. This could include new alloy compositions, surface treatments, or post-processing techniques designed to reduce H uptake, improve resistance to crack propagation, or relieve residual stresses. As the field progresses, developing standardized testing methods and benchmarks for HE in AMed materials will also become essential.

In summary, the future of HE research in the context of AM is rich with opportunities. A multidisciplinary approach combining materials science, chemistry, physics, and engineering is needed to address the complex interactions between H and AMed materials at various length scales.

### 3.3 Mitigation Strategy

The development of safety-critical infrastructures used in the field of H energy requires the proper selection of structural materials that can resist H-induced catastrophic failure in the corresponding application scenario. However, the frequently observed antagonism between strength and resistance to HE in metallic materials poses strong difficulty for the design of lightweight yet reliable structural components operating in H environments. Hence, further development of H-tolerant materials based on advanced processing and microstructural architecting, which ensures an enhanced HE resistance without any sacrifice of materials’ strength level, becomes a promising research direction that can partly solve the above challenge. Here in this section, we first provide a brief comparison of HE resistance among different engineering materials systems, after which the currently adopted strategies of HE mitigation methods and their limitations are reviewed. Future opportunities in this field are also discussed in the last part of this section.

#### 3.3.1 Comparison of HE Resistance among Different Material Systems

**Figure 53:**
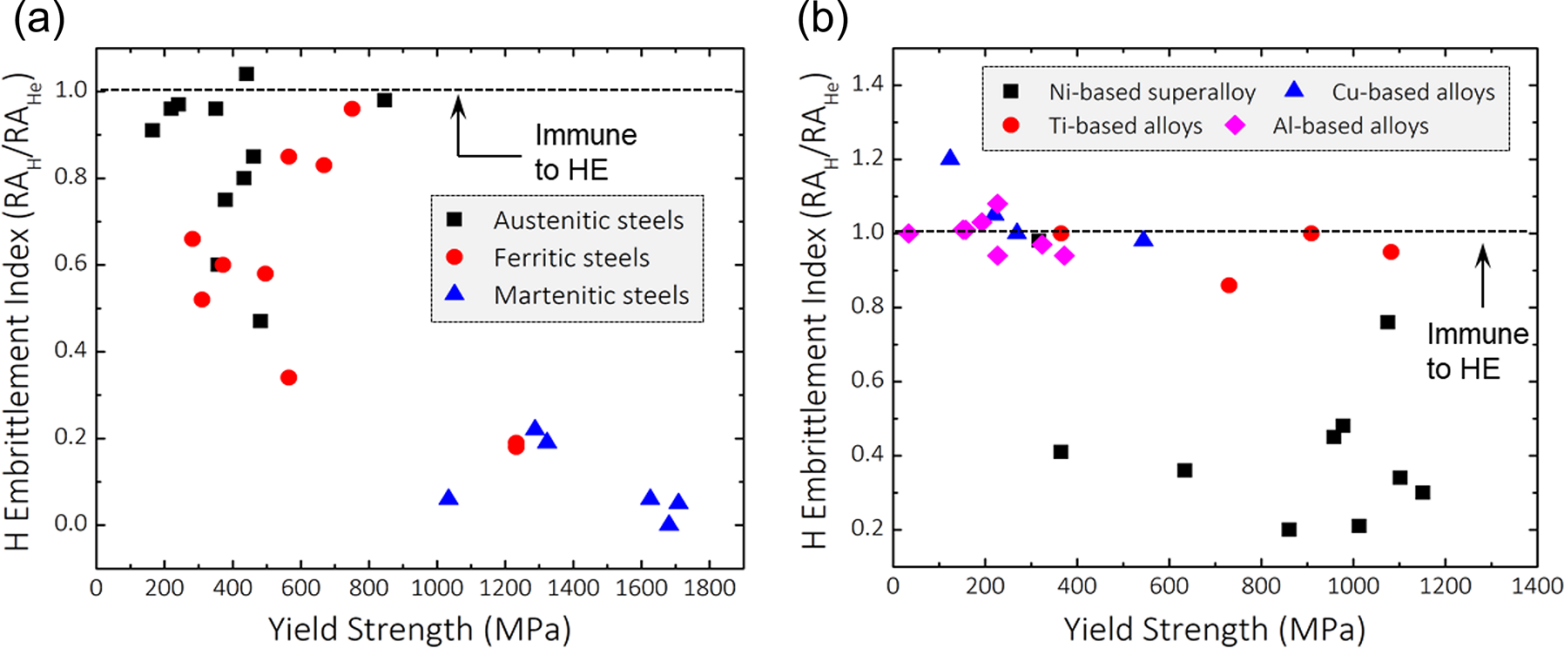
HE index as a function of yield strength for (a) steels and (b) Ni-based superalloys, Cu-based alloys, Ti-based alloys, and Al-based alloys. The index here is characterized by the ratio of reduction of area (RA) between samples tested under high-pressure H_2_ and under Helium, i.e., RA_H_/RA_He_. The H_2_ pressure of the selected data is 34.5 MPa for Ni-based superalloys and 68.9 MPa for other alloys. Data in (a) include austenitic steels (A286, 304L, 304LN, 305, 308L, 309S, 310, 316, Nitronic 32, Nitronic 40, Nitronic 40, Nitronic 50), ferritic steels (A372, A515-Gr.70, A517-F, HY-80, HY-100, iron (armco), 430F, 1020, 4140 (Q&T), 4140) and martensitic steels (H-11, Fe-9Ni-4Co-0.20C, 410, 440C, 17-7 PH, 18Ni-250 Maraging). Data in (b) include Ni-based superalloys (CM SX-2, Hastelloy X, Haynes 230, Haynes 242, IN 100, Inconel 625, Inconel 718, Inco 4005 and Rene N-4), Ti-based alloys (pure Ti, Ti-6Al-4V, Ti-5Al-2.5Sn), Cu-based alloys (pure Cu, Al bronze, Be-Cu alloy 25, 70-30 brass) and Al-based alloys (1100-T0, 201, 2024, 5086, 6061-T6, 6063, 7039, 7075-T73). All the data were taken from ref (860).

The overall mechanical properties of different materials at different H charging conditions (e.g., H pre-charging and *in situ* charging) have been systematically evaluated and can be accessed in a number of published technique reports (e.g., NASA reports NASA-TM-X-68088,^859^ NASA/TM-2016-218602,^860^ and Sandia report SAND 2012-7321^861^). These tested materials include austenitic and ferritic steels, Ni-, Fe-, and Co-based superalloys, Ti alloys, Al alloys, and Cu-based alloys.^860^ Some of the HE susceptibility data from different alloy groups (taken from ref (860)) are summarized and compared in Figure 53. It shows that under the testing condition (34.5 and 68.9 MPa H_2_ gas), some alloys including Ti-, Cu- and Ti-based alloys, are relatively immune to HE, in comparison to steels and Ni-based superalloys. Among different types of steels, it seems that martensitic steels possess the strongest susceptibility to HE, followed by ferritic steels and then austenitic steels. One reason is the higher H diffusivity within *bcc* structure compared with that in *fcc* structure (both differing by more than 2 orders of magnitude at room temperature^637,862^), and the other lies in the higher strength of martensitic steels compared with that of other steels (Figure 53a). It is interesting to find that austenitic steels generally have a higher HE resistance compared with Ni-based superalloys, despite the same crystal structure of the matrix phase and thus the similar H diffusivity (Figure 53b). This might be related to the existence of the strengthening phases in Ni-based superalloys. These phases enhance alloys’ yield strength, and on the other hand, introduce microstructure heterogeneity and heterointerfaces that can promote H-induced damages.^480,619^ Available experimental data like the those cited in Figure 53 provide important insights on the relative HE resistance among different alloys. Such information should be the first guideline for the selection of materials used in H-containing atmosphere before considering the application of any HE mitigating approaches.

#### 3.3.2 Current Strategies of HE Mitigation and Their Limitations

Despite ongoing debates and unresolved challenges in HE mechanisms, as well as a limited understanding of the prevalence and interplay of these mechanisms, the development of strategies to mitigate HE can proceed in a more straightforward manner. As discussed in Section 2, the process of HE occurs in the following steps: (a) H uptake from environment before or during the service of structural components; (b) H transport within the material, and segregation at local regions, driven by the heterogeneity of the stress field and chemical potential within the materials; (c) H-defect interactions and the associated crack nucleation and propagation. It is obvious that the suppression or delay of any of these steps should be beneficial for the enhancement of the overall HE resistance. In this regard, a number of HE mitigating strategies have been developed in the past years.

**Figure 54:**
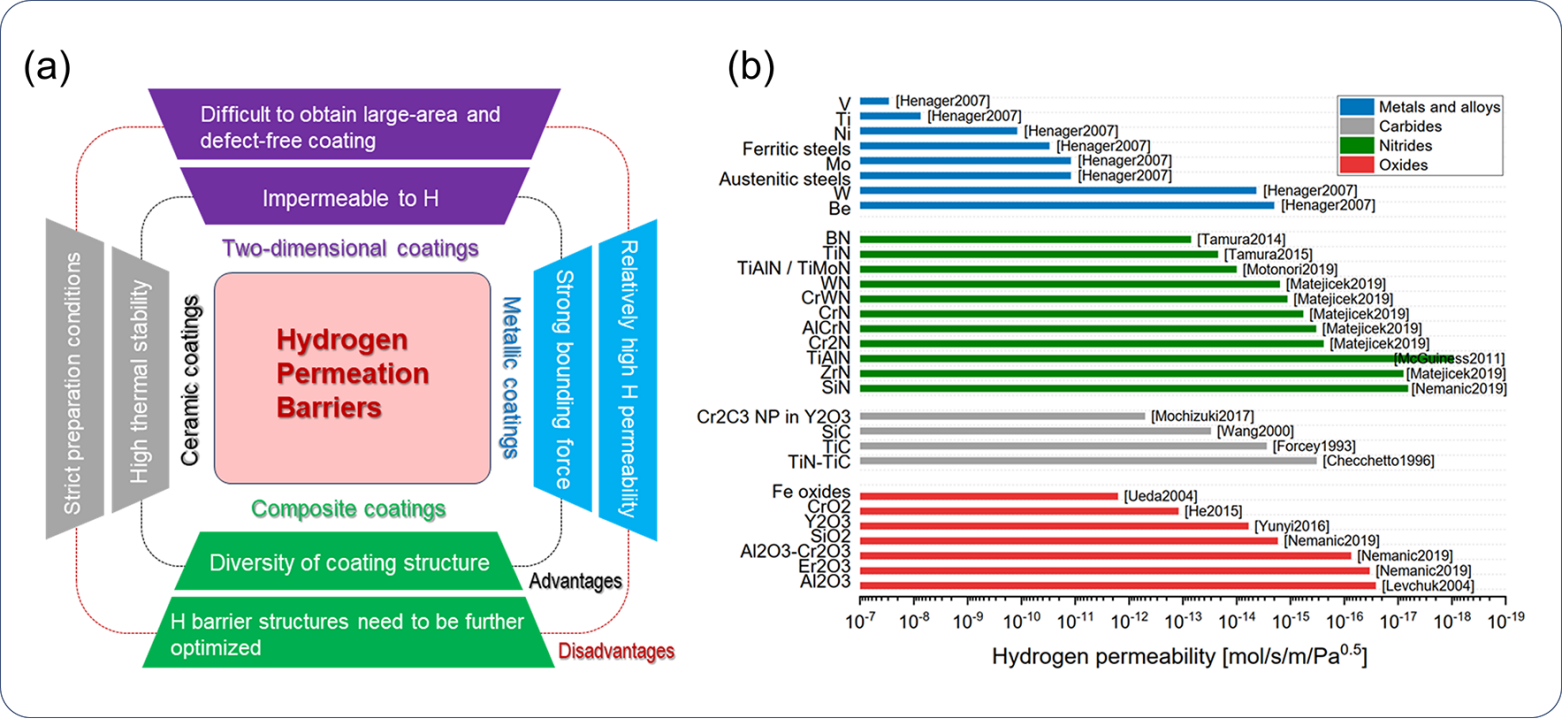
(a) Advantages and limitations of different types of H permeation barriers. Redrawn with permission based on ref (864). Copyright 2022 American Physical Society. (b) H permeabilities of various metal and ceramic coatings. Reprinted with permission from ref (866). Copyright 2023 MDPI under [CC BY 4.0 DEED] [https://creativecommons.org/licenses/by/4.0/].

The application of protective coatings or H permeation barriers is a widely applied approach to improving the H resistance of materials. Such strategy, developed in the late 1970s,^863,864^ operates simply by covering materials with one or more layers of coatings with a low H permeability or diffusivity, with the aim to prevent H ingress from H-containing environment. Reports have shown that H permeability can be reduced by up to 4 orders of magnitude^865^ after applying H permeation barriers. Currently developed H (or its isotopes) permeation barriers can be classified roughly into four groups: ceramic (oxides, carbides and nitrides) coatings, metal coatings, two-dimensional materials coatings and composite coatings.^864^ The advantages and disadvantages of these different types of H permeation barriers, along with the data of H permeability of some barrier coatings, are summarized in Figure 54 to guide future research efforts in this field. The detailed information of these H permeation barriers and their fabrication methods have been thoroughly reviewed in recent publications (e.g., refs (864 and 866)). Here we mainly contemplate a few shortcomings associated with this HE mitigating method. One of the main concerns of the coating approach is the durability problems in harsh (like abrasive and corrosive) environments. When a sacrificial coating is damaged and corrodes in service, H can be generated due to the electrochemical reaction between the exposed areas of the metal substrate and the coating material, which consequently causes significant H ingress and possibly H re-embrittlement.^867^ This shortcoming will particularly restrict the application of H permeation barriers in load-bearing components that are required to serve for long periods (e.g., H pipelines). On the other hand, it is known that H uptake can take place before coating (e.g., during casting and heat treatment of metallic materials) or during the coating process itself (e.g., electroplating). This residual H will be difficult to escape from the material after protective barriers being coated, which might lead to internal H-induced delayed fracture. As such, a low-temperature baking process is sometimes needed before or after coating to minimize the amount of residual H.

In addition to the aforementioned coating approach, one alternative is to design the composition and microstructure of the alloys to enable a more intrinsic resistance to HE. Reported alloying and microstructural strategies for such purpose include (a) the introduction of H-trapping second phases, (b) grain refinement, (c) GB engineering, (d) solute segregation and heterogeneity, and (e) surface treatment.^26,868,869^ Here we only provide a brief overview on their operating principles, advantages and limitations; more detailed information can be found elsewhere.^868^

Due to the interaction between H and certain types of second phases (e.g., V-, Ti-, and Nb-based carbides in steels), they can be used to trap H within the microstructure, thereby reducing the rate of H permeation as well as H diffusion within the material. This factor, in principle, will enhance alloys’ HE resistance, regardless of the prevalent damage mechanisms. However, the effect can become detrimental if the precipitate itself and the related hetero-interfaces are prone to H-induced cracking.^637,868,870,871^ On the other hand, the trapping effect of second-phases will essentially increase the uptake of H. This additional amount of H, if trapped not deeply enough, might become diffusible particularly under elevated temperatures, which can enhance the risks of HE. It should also be noted that the effectiveness of the second-phase trapping approach is highly dependent on the service environment of a material. Due to the constraint of H trapping/storage content arising from the typically low second-phase volume fraction (e.g., below 1% for strengthening carbides in ferritic steels^872^), such approach is expected to be most effective for the cases with only a limited H uptake from environments (e.g., during the pickling process). However, it should not be an ideal approach for materials operated in H-abundant atmosphere (e.g., H transport and storage) where continuous H ingress will occur and inevitably will saturate the H traps. Therefore, the application of such approach will need to carefully consider the targeted materials and their service scenarios.

The enhancement of HE resistance via grain refinement has been demonstrated in a number of materials including high-Mn TWIP steels,^510,873,874^ some austenitic stainless steels,^875−877^ and a few HEAs.^551^ The common feature of these alloys is that they all possess an *fcc* microstructure that is prone to H-induced IG cracking. It has been proposed that grain refinement (i.e., increased density of GBs) can effectively reduce the H coverage on GBs for a constant overall H amount, thus reducing the tendency of H-induced GB decohesion.^440,510,874^ Another reason for the improved HE resistance is the reduced stress concentration at the GBs due to the lower number of dislocation pile-ups therein and/or suppressed deformation twinning in samples with a smaller grain size.^874,878^ Although the grain refinement approach has been shown to be effective in some *fcc* alloys, its potency has been seldom verified in complex materials (e.g., martensitic or multiphase steels) with more complicated HE mechanisms and H-induced damage behaviors. Further, it is established that the strain-hardening ability (thus the uniform elongation) of materials is strongly reduced when the grain size reaches below a few micrometers.^879,880^

In contrast to the grain refinement approach that serves to increase the density of interfaces, the strategies of GB engineering and interfacial solute segregation work alternatively on changing the intrinsic property of interfaces. GB engineering aims to increase the fraction of lower-energy interfaces (or special boundaries) in order to lower the tendency for H-induced interface cracking.^881−883^ Annealing twin boundaries (coincidence site lattice Σ3 type) are typical low-energy interfaces that can be purposely introduced into materials for H tolerance. Such low-energy interfaces retain a lower binding energy with H which results in less H trapping. The separation energy of these interfaces is also higher than random high-angle GBs. Further, the network formation of special GBs can disrupt the continuity of the random GBs and thus interrupt the continuous IG cracking path. Solute segregation is another way to alter the interface property. Reports have exhibited an increasing effect of certain elements (e.g., C and B) on the cohesive strength of GBs.^884−887^ Such influence can be utilized to inhibit H-induced interface cracking, thus reducing the overall HE susceptibility.^888,889^ In addition to the effect on interface cohesive strength, the pre-occupation of solutes might also influence the H trapping behavior at the interfaces, which in turn influences the HE properties. For example, *ab initio* calculations have shown that the presence of C atoms at the tilt GBs in iron can enhance the H concentration at nearby vacant interstitial sites, due to the increased local strain induced by the segregating C.^890^ Nevertheless, it is obvious that both the GB engineering and interface segregation approaches can only be effective for materials whose failure is dominated by H-induced interfacial cracking. The processing methods for the adjustment of interface structure and chemistry in different materials also need to be explored in order to extend their application to a wider group of materials.

For some alloys, the change of composition significantly influences the deformation behavior, which can consequently alter the alloys’ HE resistance. In these circumstances, a local variation in element contents within the microstructure, which can be achieved by, for instance, novel thermomechanical treatment, is also expected to locally influence the resistance to H-induced cracking. Such chemical heterogeneity strategy, proposed by Sun et al.,^637^ has been used to improve the HE resistance of metastable high-strength steels undergoing deformation-induced martensite transformation. One particular advantage of this approach lies in its ability to reconcile the intrinsic conflict between the TRIP effect (providing strain-hardening ability and thus strength-ductility combination) on the one hand and HE resistance on the other. More specifically in their work,^637^ a confined Mn heterogeneity within the austenite was produced by multi-step annealing treatment. This microstructure creates a micromechanical composite effect consisting of adjacent stable (Mn-rich) and metastable (Mn-lean) austenite zones acting in a way that the H-induced cracks nucleated in the solute-lean metastable TRIP zones can be arrested by the adjacent solute-rich stable buffer regions. As a result, a two-fold improvement in HE resistance can be achieved without sacrificing the alloy’s overall strength and strain-hardening ability. In the following work by Zhang et al.,^494^ it was shown that such improvement can be further increased when the Mn-rich region was designed to form near the austenite GBs. In this case, the Mn-rich stable austenite region can even suppress the nucleation of the H-induced cracks at the interface regions. In comparison to the other strategies mentioned above, mitigating HE by chemical heterogeneity is a less straightforward method that requires a deeper understanding of the specific materials and their operating HE mechanisms. It is likely that the processing methods to produce controlled chemical heterogeneity against HE will differ significantly among different groups of materials, which will require future research efforts.

Unlike the aforementioned HE mitigating approaches that deal with the whole bulk of the materials, surface treatments aim to tailor the microstructure at or near the surface regions, which can sometimes be beneficial for HE resistance. Commonly reported surface treatment methods for such purpose include shot and laser peening,^147,635,891,892^ which can introduce compressive residual stress and H-trapping lattice defects (e.g., dislocations and GBs) at the surface regions. These near-surface microstructure change might suppress H permeation from the environment thus delaying the initiation of H-induced cracks at these regions. It should be noted that controversial findings exist pertaining to the actual effectiveness of these methods on suppressing H permeation and the occurrence of HE.^893−895^ A more systematic study thus needs to be conducted in the future to establish the correlation between surface treatment parameters, H permeation and internal diffusion, and HE resistance.

The applicability, advantages, and disadvantages of these different HE mitigating approaches discussed in this section are summarized in Table 6. It should be noted that we only focused on some major types of HE mitigating methods in this section. Sometimes for certain materials, the approaches can be very specific, which are not covered here. Given the growing demand of reliable infrastructures needed for a green H economy, research activities in this field are expected to expand in the coming years. However, it has to be noted that the HE problem in high-strength alloys is always a matter of the specific degree of embrittlement. Completely preventing the occurrence of HE under any loading or environmental conditions cannot be considered as a rational target in science. More realistic goal is to suppress it to make materials capable of tolerating the essential H-uptake over the service lifetime. The service conditions of the target materials thus need to be considered before selecting or designing HE mitigating approaches. In this regard, mechanism-based predictive models which allow the precise evaluation of the H compatibility and lifetime of a material at (near) service conditions becomes particularly important, which should also be further developed.

**Table 6:** Summary of the Applicability, Pros and Cons of Different HE Mitigating Approaches Discussed in This Section^a^

HE mitigating methods	Applicability	Advantages	Disadvantages
H-trapping precipitates	Typically ferritic/martensitic steels.	1. Strength at H-free condition is not sacrificed.	1. The ductility at H-free condition is typically sacrificed.
		2. Different types of precipitates with different H binding energies can be explored.	2. The effectiveness is strongly bounded by small volume fraction of precipitates.
			3. Once the precipitates are saturated with H, the approach becomes ineffective and often detrimental.
Grain refinement	Normally single-phase austenitic materials where H induces IG cracking and the interaction between deformation twins or dislocation arrays and GBs is the major factor for such cracking.	1. No change of alloy composition.	1. The strain-hardening and ductility at H-free condition are seriously decreased when the grain size is refined down to a few micrometers.
		2. Strength at H-free condition is normally improved.	2. Severe deformation is often needed, which has a very low scalability.
GB engineering and solute segregation	Normally materials where H-induced interface cracking is the major cause for HE.	1. Strength and ductility at H-free condition is not sacrificed.	1. Require additional thermomechanical treatments and/or addition of microalloying elements.
		2. In principle also effective in H-abundant environment.	2. Knowledge about the interplay between H, other segregating elements, interface structure and cohesive strength is limited.
Chemical heterogeneity	Steels containing metastable austenite.	1. No change of alloy composition.	1. Might require additional thermomechanical treatments.
		2. No sacrifice of alloys’ strength, strain-hardening and ductility at H-free condition.	
		3. A large variety of microstructure conditions, chemical patterning and processing routes can be explored.	
Surface treatment	The actual potency for different materials is not conclusive and need to be validated.	1. No change of alloy composition and the microstructure in the interior of the materials.	1. Might increase surface roughness that promotes H-induced crack nucleation at the surface region.
		2. No sacrifice of alloys’ strength.	2. Require additional processing step.
		3. Beneficial for materials’ fatigue life in H-free condition.	3. Difficult to be applied for complex structural components.

a Adapted with permission from ref (868). Copyright 2023 Wiley.

### 3.4 Microstructure-Informed Mechanism-Based Predictive Models

#### 3.4.1 Needs for Mechanism-Based Predictive Models

H poses a significant risk as a “silent assassin” in materials, potentially causing weakening without noticeable damage or deformation until catastrophic failure occurs. To mitigate such failures, two primary strategies are available: developing H-resistant materials or adjusting operational parameters to maintain a clear safety margin. Both strategies depend on predictive models, which are mechanistic models designed to forecast critical conditions leading to cracking, based on parameters like loading, H environment, and material properties. The absence of robust predictive models necessitates extensive testing and reliance on traditional rules of thumb, which can sometimes lead to accidents.^21^ Despite over a century of HE research and accumulated knowledge, regrettably, the predictive models have not achieved the same level of richness that aligns with this long history. Martin et al.^21^ stated in their recent review article that “possibly the most crucial shortcoming of the HE field is a dearth of predictive models”. From a practical perspective, the existing predictive models have not advanced in a manner that provide drastic improvements in life prediction of engineering components.^896^

Our experience in mechanism-based numerical modeling of HE indicates that mechanisms with a well-defined scope are more conducive to developing numerical models. Additionally, the more quantitatively defined a mechanism is, the better it serves predictive modeling. Most HE mechanisms, except for the HELP mechanism, were initially proposed qualitatively and many remain so. The HELP mechanism was initially supported by an H elastic shielding theory, which was derived by solving for the total stress field associated with an H atmosphere.^41,732^ Later, it was mathematically demonstrated that the HELP mechanism causes a plastic instability in a continuum body at a global scale, which can be interpreted as a type of failure (sudden loss of loading bearing capacity). Despite the limitations with HELP mechanism-based modeling, as detailed in Section 2.3.6.2, its inherent quantitative nature has made it a popular choice for numerical simulations.^692,693,709^ Conversely, the HEDE mechanism was conceptualized qualitatively but has since been quantified through atomistic calculations,^324^ and the mechanism has been frequently employed in numerical simulations.^25,219,680,681^ The Defactant concept and the AIDE mechanism, however, have not yet been quantified and are less commonly used in simulations.

While the Defactant concept and the AIDE mechanism need to be quantified, the quantitative aspects of the HELP and HEDE mechanisms need further refinement and specificity. For example, most of the H informed CZM simulations based on the HEDE mechanism have been based on the H degradation relation calibrated from DFT calculations, which has limitations. The applicability of DFT-rooted degradation laws to continuum analysis, spanning a significant length scale gap, is questionable. Additionally, while HEDE may mimic macroscopic fracture modes, direct decohesion has not been conclusively verified experimentally. Often, HEDE needs to be integrated with other mechanisms like HELP and HESIV to set a precondition for decohesion.

From a broad perspective, predicting H induced failure in materials involves several intertwined elements: (1) accurate account of H uptake, diffusion and trapping; (2) H-induced degradation or damage model based on HE mechanism(s); and (3) microstructure-specific parameters which can be transferred from lab-scale tests to in-service conditions. Whether dealing with brittle or ductile fracture, a cracking criterion invariably incorporates a length scale parameter, which is material-dependent and falls under microstructure-informed parameters. While the understanding and modeling of H-metal surface interactions need substantial improvement, H diffusion within the metal can be effectively analyzed through thermomechanical analysis, either in a sequentially or simultaneously coupled manner. However, the major challenge remains in developing a mechanism-based H degradation model and accurately determining microstructure-informed parameters.

#### 3.4.2 Promising Void-Based Predictive Models

As mentioned in Section 2.3.6, the combined action of HELP and HEDE mechanisms has been successfully implemented in numerical simulation and proven effective in capturing the failure point as well as the ductile to brittle fracture transition. It is appealing to implement the HESIV mechanism in predictive modeling, for its inherent link to HELP and to fracture, which has been highlighted in a number of recent review articles.^20,21^ Void-based predictive models provide a promising framework for incorporating the HESIV mechanism, together with the HELP and HEDE mechanisms.

Microvoids play a pivotal role in ductile fracture and ultimately lead to the formation of the characteristic dimpled fracture surface. Void nucleation, growth and coalescence-based mechanistic models are available and can be used for predicting ductile fracture. In the presence of H, the fracture surface can exhibit different forms, ranging from a dimpled ductile type to a quasi-cleavage flat surface, and in some cases IG fracture, contingent upon the H concentration level in the material and other pertinent factors. When the H concentration is low, the fracture surface is still dimple-like, and fracture is still governed by the void-based mechanism. However, in the case of high concentration, where a macroscopically flat quasi-cleavage surface is induced, it has been observed that voids are still present on these flat surfaces.^485,897^ A TEM study conducted on the fracture surface of a pipeline steel charged with H unveiled the presence of numerous nanoscale dimples, despite the surface appearing macroscopically flat.^9^ While the details are not fully understood, both experimental studies and MD simulations^659,675^ have shown that void-based failure mechanism is still viable in the presence of H. A distinction between the voids observed on ductile fracture and quasi-cleavage fracture surface with H is their size and shape. In the presence of H, the size of the dimples can be orders of magnitude smaller than those observed on a ductile fracture surface. All these findings tend to advocate for the development of void-based predictive models for HE.

Among the three steps of ductile fracture, growth of an existing void is well understood as it is controlled by plasticity and stress triaxiality. Void coalescence, which represents the final stage of ductile fracture, can now be accurately predicted by an analytical or micromechanical solution in the form of plastic localization or void sheeting. On the other hand, void nucleation, which arises from local chemical and mechanical inhomogeneity, remains the least understood aspect of ductile fracture. Specifically, voids involved in the fracture process can be classified into primary voids and secondary voids. The primary voids are those “easy-to-nucleate” voids and are usually assumed to be present from the beginning of plastic deformation. Primary voids can be nucleated by the fracture of particles or inclusions or debonding of the particle and matrix interface.^898^ The nucleation of secondary voids, or the deformation process-nucleated voids, is commonly assumed to be stress- or strain-controlled. Secondary voids can be nucleated from various microstructure features, including GBs, phase boundaries, twin-parent boundaries, inclusions/precipitates etc. The interaction between the primary and secondary voids is often neglected in the modeling of fracture.

Because of the “easy-to-nucleate” nature, the primary voids that occur in the H-free case may still exist in the presence of H and could be present at the onset of plastic deformation. In addition to the secondary voids mentioned above, nanovoids can be nucleated as a result of the accumulation of strain induced vacancies at GBs,^899^ along dislocation slip bands^480^ and at triple junctions promoted by HELP and HESIV mechanisms. They can also form as a result of the cracking of the GB precipitates or interfacial failure between the GB precipitates and the matrix. Unlike the ductile facture, the interaction between the primary voids and secondary (nano-) voids cannot be neglected when H is present. The nucleation of nanovoids can contribute to the weakening of the ligament between the primary voids or act as a softening zone, promoting the shear localization or shear sheeting and resulting in plasticity mediated inter-void decohesion. Depending on the void volume fraction and nucleation rate, the nucleation of voids can also lead to incipient decohesion.

**Figure 55:**
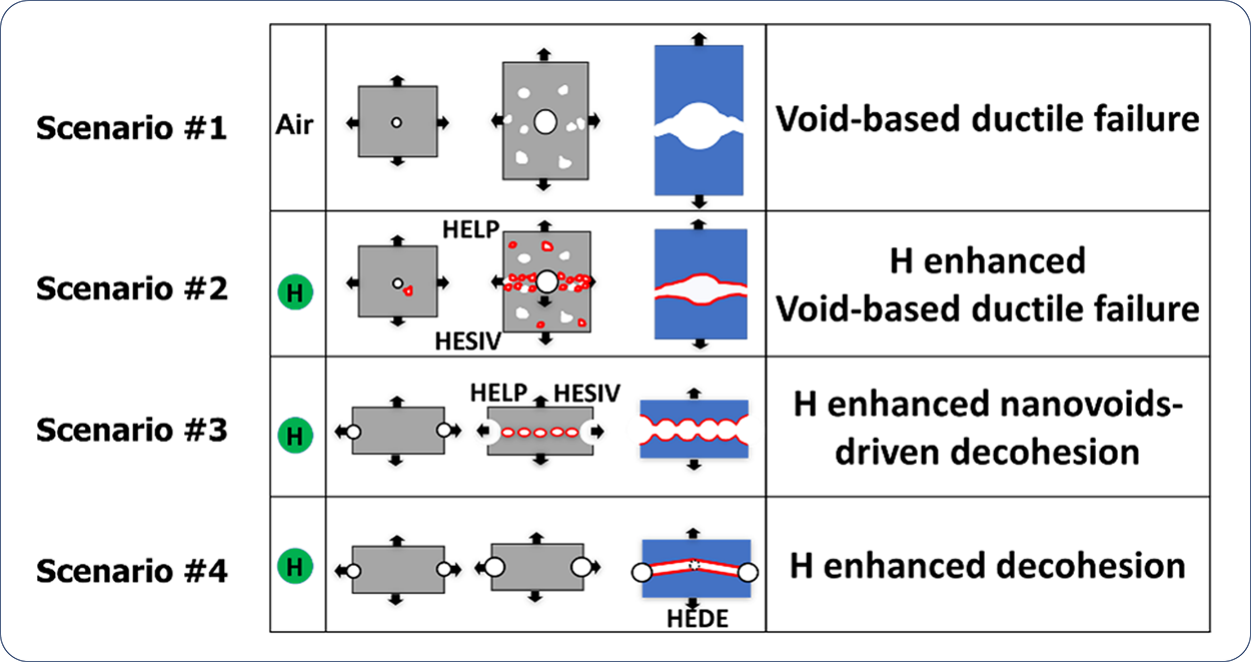
Schematic illustration of the four failure modes in a void-based predictive modeling framework. Scenario #1, void-based ductile failure (without H); Scenario #2, H-enhanced void-based failure (low H concentration); Scenario #3, H-enhanced nanovoid-driven decohesion failure; Scenario #4, stress- or energy-based H-enhanced decohesion failure (high H concentration).

Based on the above analyses, a promising void-based HE predictive framework can be established. The predictive framework should be able to account for the following scenarios, which are schematically illustrated in the Figure 55.

A ductile fracture process can be predicted, for example, by the well-known Gurson-Tvergaard-Needleman (GTN) model. In the original GTN model, both the void nucleation and coalescence parameters have to be selected beforehand. Recognizing that failure by coalescence of a porous solid is a natural outcome of the plastic deformation process, a modified, so-called complete Gurson model (CGM)^900^ was developed by implementing a physics-based coalescence criterion into the GTN model and thus eliminating the critical coalescence parameter. An H-informed CGM has already been developed which united the scenario #1 and #2^712^ into one framework. Much work remains to be done to realize the scenario #3 and #4. In scenario #3, both H enhanced strain-induced vacancies and microstructure feature-induced vacancies should be considered. The crucial task is to establish a correlation between the nanovoid volume fraction as a function of plastic strain and H concentration.

#### 3.4.3 Testing Method for Identifying Microstructure-Informed Material Parameters

Once a mechanism-based degradation model is established, the subsequent task is identifying material microstructure-informed parameters through specific laboratory experiments. Numerous testing methods are available for characterizing HE susceptibility, as detailed in section 2.4.4. Most of these tests, which allow for adjustable H charging conditions, are typically used for screening purposes or for ranking materials’ susceptibility to HE.^901^ However, the outcomes of these tests are often binary and do not necessarily indicate service performance due to the limited exposure time and somewhat arbitrary strain rates used. Additionally, these methods vary in terms of specimen shape and size, as well as stress concentration and triaxiality.

There is an urgent need to develop reliable testing methods that focus on relevant microstructures, uncover critical failure mechanisms under realistic levels of H and stress concentration, and facilitate the extraction of transferable model parameters for structural integrity assessment. Transferring results from accelerated laboratory conditions to predict service life under actual loading and environmental conditions remains a significant challenge. Whether a testing method can provide correct evaluation parameters depends on its capability to represent the in-service conditions.

### 3.5 Machine Learning in HE Research

In the last decade, ML has ushered in a new era of scientific discovery, being as a transformative force to revolutionize various disciplines with its ability to analyze vast datasets and uncover hidden patterns.^197,348−350,902−905^ In particular, ML has demonstrated exceptional proficiency in tackling the complexities of materials science.^906−909^ The integration of ML with materials science has thus opened new frontiers, enabling us to explore and comprehend complex material behaviors, predict material properties, design novel materials, and accelerate the discovery process.

In the case of HE, a conspicuous material challenges to the safety and integrity of H energy and many other engineering systems, the intricate interplay of H with material microstructures and the underlying mechanisms still remain enigmatic, hindering the development of effective strategies to mitigate and prevent HE.^8,26,436,661,910^ ML embraces data-driven analysis, facilitating the extraction of insights from extensive datasets that encapsulate the intricate interplay between H and material microstructures.^911−913^ By extracting patterns and relationships from these data, ML offers the promise of unlocking new knowledge about HE. In this section, we mainly focus on the current progress regarding ML-based approaches, particularly in the following two areas: (1) ML-based predictive modeling of HE and (2) accelerated atomistic simulations with ML.

#### 3.5.1 ML-Based Predictive Modeling of HE

Quantitative prediction of HE is still very challenging today due to the multiscale nature of metal–H interactions. The predictive power of ML extends beyond traditional models, which is deemed to capture multiscale information of HE not only in energetics and kinetics but also in macroscopic mechanical responses.

**Figure 56:**
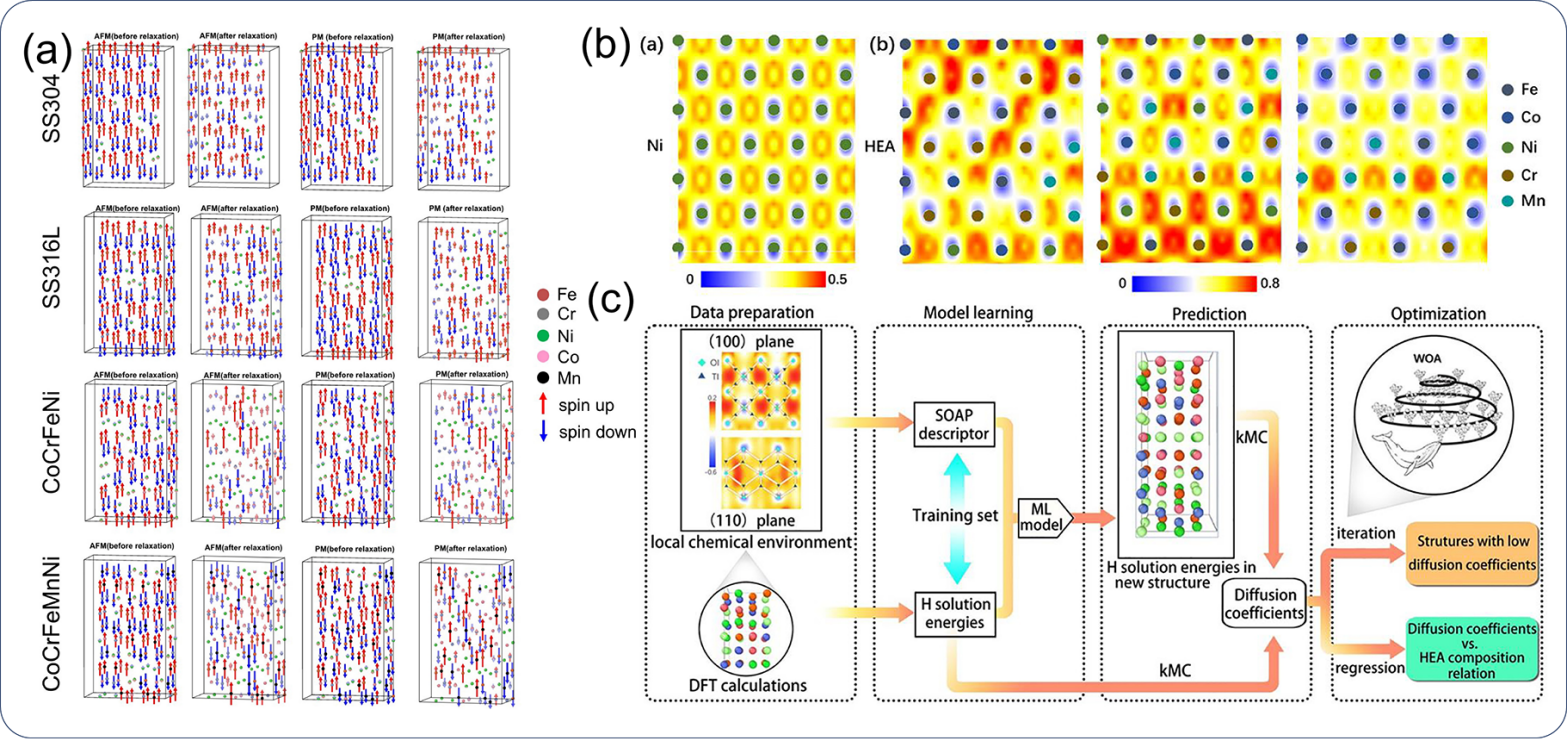
Local chemical and magnetic moment of HEAs and the workflow for predicting H solution energies in HEAs using ML model. (a) Local chemical and magnetic moment in HEAs. Reprinted with permission from ref (915). Copyright 2020 Elsevier under [CC BY 4.0 DEED] [https://creativecommons.org/licenses/by/4.0/]. (b) The distribution of H solution energies in HEAs. (c) Illustration of ML workflow. Reprinted with permission from ref (916). Copyright 2022 Elsevier.

Recently, a few works have attempted to study the energetic and kinetic properties of impurities, such as H in metals and alloys, using ML to establish a more rapid strategy for evaluating the basic properties at the atomic scale. For instance, Zeng et al.^914^ investigated the diffusion activation energies of various interstitial light element impurities in representative crystal structures such as *fcc*, *bcc*, and *hcp* pure metals by gradient boosting with decision tree regression algorithm. It is worth noting that gradient boosting with decision tree regression is not only able to predict diffusion activation energies using small databases, but can also filter features based on their importance, which reduces the complexity of the model by eliminating less important features. Based on this ML model, Zeng et al.^914^ found that the significant physical feature, elastic strain energy, plays the vital role in activating diffusion energy in the examined materials systems. Their result is consistent with intuitive understanding that the lattice distortion induced by the embedding of interstitial light element would contribute to the change of energetics of the whole system. In the ML model, the suitable selection of physical feature or so-called descriptors are of great importance. For more complex systems involved multiple compositions such as HEAs, the local chemical environments are distinct from pure metals, which renders ML prediction of H behavior more difficult. Selecting suitable physical features and ML algorithms that can effectively capture H solution and activation energies remains non-trivial. For instance, Zhou et al.^915^ used local chemical bonds and local magnetic moments as important descriptors in order to predict H solution energies, as seen in Figure 56a. However, although the local chemical and magnetic environments indeed affect H energetics, there seems no clear correlation between the defined physical features and H solution energies. Lately, Wu et al.^916^ combined the Smooth Overlap of Atomic Positions (SOAP) descriptors with different ML algorithms (i.e., Random Forest, Ridge Regression and Neural Network) to predict H solution energies in HEAs. It should be highlighted that the SOAP descriptors that includes the important hyperparameters seem able to capture the local chemical and crystallographic information more accurately.^916^ After scrutinization of the examined ML models, the Neural Network was finally chosen for predicting H solution energies because of the superior performance than others. The ML model was then used for predicting H solution energies at representative sites in newly generated HEA samples using Whale optimization algorithms. With the fast calculation of energetics by the ML model, H diffusion coefficient can be further evaluated using kinetic Monte Carlo simulations, which provides new insights toward fast characterization of H behaviors in complex alloys, as presented in Figure 56c.

**Figure 57:**
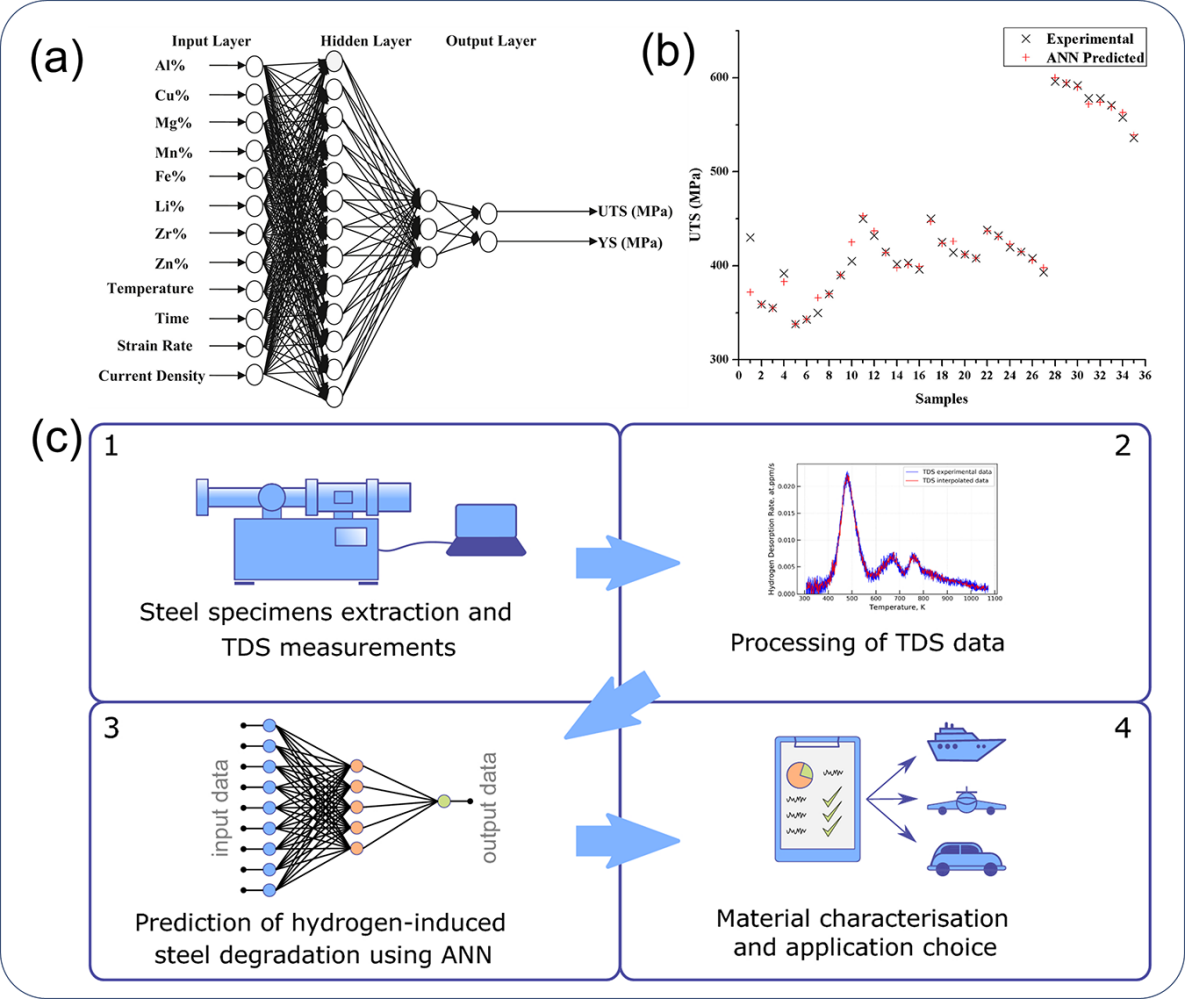
(a) ANN model for predicting HE susceptibility of Al alloy. (b) The correlation between ANN model predicted mechanical properties and experimentally measured results for Al alloy. Reprinted with permission from ref (917). Copyright 2017 Elsevier. (c) Schematic illustration of ANN-coupled TDS-based ML model for HE prediction. Reprinted with permission from ref (919). Copyright 2020 Springer Nature under [CC BY 4.0 DEED] [https://creativecommons.org/licenses/by/4.0/].

Beyond the predictive capacity of ML models for fundamental H properties in energetics and kinetics at the atomic scale, these models can be extended to include training on experimental datasets. A proficiently trained ML model enables the prediction of HE, attributable to a constellation of complex factors such as manufacturing processes and environmental conditions. This capability is particularly instructive for engineering applications. For instance, Campari et al.^913^ proposed one ML predictive model based on the Gradient Boosting algorithm to assess the risk of HE in steels. In particular, the experimentally measured embrittlement index, the degradation of mechanical properties, experimental conditions (e.g., pressure, temperature, etc.) as well as loading conditions were used for the construction of dataset, followed by the ML model training and prediction of HE severity. The trained ML model reached 88.6% accuracy, suggesting relatively high capability in HE prediction. Early attempts by Thankachan et al.^917^ were directed to predict the HE of aluminum alloys using Artificial Neural Network (ANN) model in which different parameters such as alloys compositions, temperature, strain rate, and H charging conditions were fed in ANN model as inputs (see Figure 57a). In accordance to ANN, single layer and multilayer feed forward back propagation algorithm is adopted as ANN model in order to train the collected mechanical data (ultimate tensile stress, yield stress and percentage elongation) from the literature. The trained model showed good predictive ability in mechanical response in the absence and presence of H. However, despite the good correlation between experimental mechanical properties and predicted values by the ANN model, the developed ANN model probably had unsatisfactory generalization capability. On the one hand, the datasets used in model training were insufficient where only 40 data points from literature were acquired. The limited datasets definitely constrained the model extrapolation, which cannot guarantee good prediction for unexplored cases. On the other hand, the physical features (or descriptors) selected in the ANN model seem to be too simple to uncover the underlying mechanisms or dominant factors that controlling HE. In particular, Thankachan et al.^917^ mainly concerned temperature, time, strain rate and current density of H charging as main factors that influence the HE susceptibility, which cannot capture the critical correlations between external environment conditions, H behavior in microstructures, or mechanical degradation caused by H.

To further correlate H transport in the microstructure to the susceptibility to HE, Malitckii et al.^918,919^ built one conceptual method that is able to link TDS with HE using ANN model. Worthy of note is that TDS is considered an effective way to assess the microstructure-dependent H trapping. Therefore, using TDS as inputs in ANN model can account for the influence of microstructure on H distribution. For better quantifying the susceptibility to HE, the well-defined H sensitivity parameter (HSP), calculated as HSP = (ε – ε_H_)/ε × 100% where ε and ε_H_ are the failure strains without and with H, was utilized for training. Based on the approach raised by Malitckii et al.,^918,919^ two ANN models were trained and validated for a series of materials including austenitic, ferritic, and ferritic-–artensitic steels. Both trained ANN models exhibited good accuracy in which a correlation of more than 90% is achieved between experimentally measured HSP and model predicted values, suggesting that the ANN models fed by TDS data can be a robust tool for predicting HE in metallic systems. However, although the trained ANN models exhibited good performance in the prediction of HE for the examined steels in that work, caution should still be taken regarding the application of those models to other types of alloys. In other words, the ANN model was developed based on the assumption that the trapping status of H in the microstructure does not change substantially and the TDS data is case-specific. Therefore, once the microstructure or H status is changed in different samples fabricated even by the same material, the ANN model probably fails to capture the corrected mechanical response in H. Similar to the aforementioned work, another workflow coupling with data collection, feature engineering and ML algorithm was proposed by Kim et al.^920^ for predicting HE in austenitic steels. In their work, four representative ML algorithms (i.e., Random Forest, Linear Regression, Bayesian Ridge, and Support Vector Machine) are fully examined and the Random Forest ML model was found to exhibit the best performance and highest accuracy. In addition, with the introduction of feature engineering (i.e., Pearson’s correlation coefficient and Maximum Information coefficient), the dominant elements affecting HE of austenitic steels were found to be the Ni and Mo. This ML-based workflow shows high potential in the design of novel materials that are resistant to HE.

The above-referenced endeavor elucidates some direct correlation between important characteristics of materials and their susceptibility to HE via ML. Yet, the ML methodologies detailed within the aforementioned analyses predominantly fall short in demystifying the underlying mechanisms influencing HE. To address this gap, Phan et al.^921^ employed ML algorithms, notably Random Forest models, to predict key variables that is related to the assessment of pipeline integrity under high H pressure. In particular, they merged the ML algorithms with fracture mechanics and finite element analysis to explore the possible reasons causing burst failure in the presence of H. The Random Forest ML model demonstrated high accuracy closely aligning with finite element results. However, the models still showed limitations in predicting premature failure dominated by the elastic regime. Despite the foregoing shortcomings, that work provided insight into the development of data-driven models for the assessment of HE, advocating for the integration of simplified and accessible methodologies at a macroscopic level.

Transferring microscale information to the macroscale fracture model, thus accounting for the multiscale nature of HE phenomena, remains a great challenge. ML holds promise for bridging this gap by enabling the transfer of information across scales. Hasan et al.^911,922^ presented a novel approach to predict the failure probability of hydrided zirconium materials, using a combination of deterministic physics-based continuum simulations and ML techniques. At the microstructure level, their approach was able to predict the onset of cracks by focusing on features that reflect the buildup of stationary and moving dislocations (microstructural defects). Their work demonstrated that mechanistic insights into HE can be realized using the combination of fracture mechanics and ML, suggesting a viable pathway for integrating detailed microscopic information into upper scale models for predicting material failure.

The remarkable predictive capacity of ML has been demonstrated, yet substantial effort is needed to address a number of critical issues to ensure the accurate and reliable application of ML models in the context of HE. Paramount among these is the quality and volume of data. The integrity and representativeness of data, procured from reputable sources and covering a comprehensive range of microstructural defects, H interactions, and material properties pertaining to HE are critical. Furthermore, the validation and interpretability of models are crucial. The rigorous validation of ML models using independent datasets is imperative to assess their performance and generalization capabilities. In particular, the interpretability of ML models, especially in safety-critical domains such as material design, is critical for elucidating the rationale behind predictions and gaining insights into underlying mechanisms. Lastly, the integration of domain knowledge, such as the foundational theories and models of solid mechanics, into ML processes is necessary for the comprehensive understanding of HE mechanisms. Therefore, developing physics-based or physics-informed ML models is of great importance.

#### 3.5.2 Acceleration of Atomistic Simulations with ML

In the preceding section, it was shown that the ML-based predictive models can significantly improve our ability to see the correlation between HE and potential influential factors. However, most of the established models are like “black boxes” and not clearly interpretable. This essentially means that, despite the ML models' efficacy in uncovering correlations between input variables (such as composition, processing, and H charging conditions) and output properties (like yield strength, ultimate strength, and failure strain), they fall short in demystifying the intricate physical mechanisms of HE.

Atomistic simulation offers an important means to explore the details of metal–H interactions at atomic/nano scale.^358,672,923^ However, there are limitations inherent in atomistic simulations. On the one hand, *ab initio* simulations which provide the most accurate energies for H interacting with various crystal defects, are computationally expensive and only applicable to very small simulation systems containing several hundred atoms. On the other hand, MD simulations are less-computationally intensive and are able to deal with large simulation systems with over a million or even a billion atoms with high efficiency. However, it must be noted that the simulation outcomes are completely dependent on and limited by the reliability and accuracy of the applied interatomic potentials, while the development of interatomic potentials with high fidelity is a challenging task.

**Figure 58:**
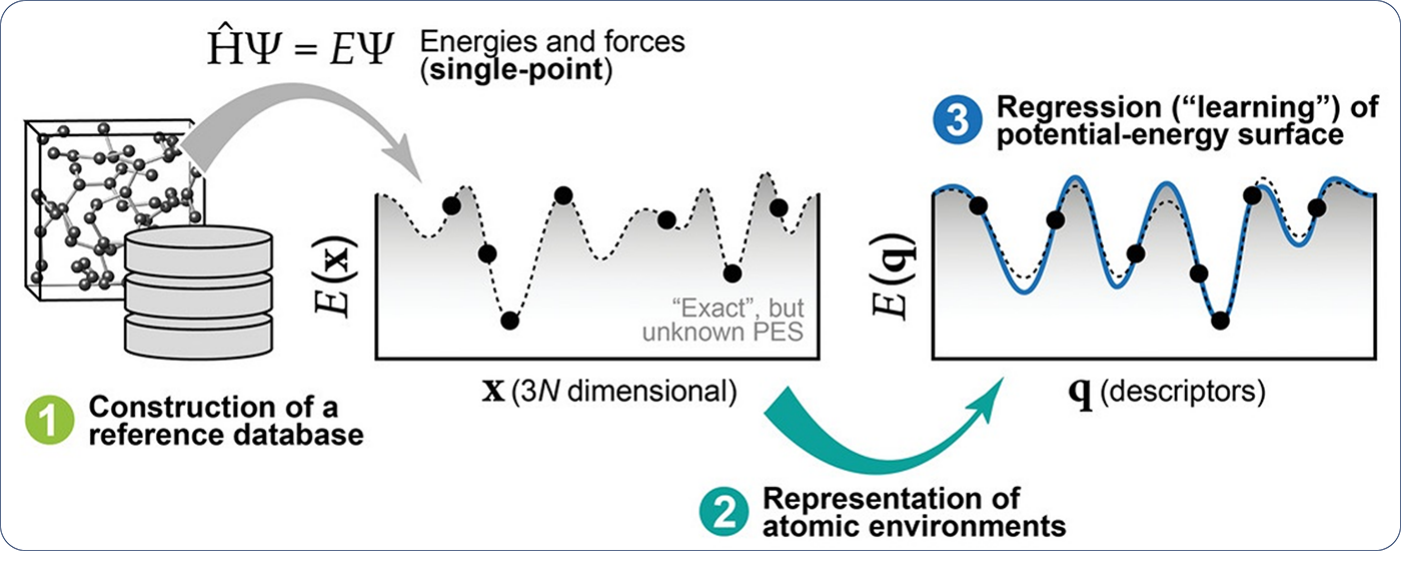
General workflow regarding the construction of ML interatomic potentials. The first step is the construction of a reference database, followed by the computation of energies and forces by means of first principle calculations; the second step is the description of atomic structure with machine-readable descriptor; and the final step is the regression (learning) of potential energy surface. Reprinted with permission from ref (924). Copyright 2019 Wiley.

In recent years, ML has emerged as one powerful tool to construct the interatomic potentials (i.e., ML potential) in order to narrow the gap between the accuracy of *ab initio* calculations and the efficiency of MD simulations, using semi-empirical interatomic potentials, for example, the embedded-atom method (EAM) and the modified EAM (MEAM) potentials.^925,926^ Unlike the philosophy of building semi-empirical interatomic potentials, where the properties-oriented objects are fitted by various optimization algorithms, the fundamental concept of ML potentials is the direct fitting of datasets of energies and atomic forces using ML algorithms. Afterwards, numerical interpolation is employed to predict the potential energy of an examined system. The reference datasets are often obtained from ab-initio calculations^924,927^ (see Figure 58). The advantage of ML potential is its ability to simulate the system with a level of accuracy comparable to *ab initio* calculations while being orders of magnitude faster, which expands the applicability of DFT simulation to substantially larger systems and a much longer timescale.

Despite that numerous ML potentials have been constructed and reported in the literature such as Moment Tensor Potential (MTP), Gaussian Approximation Potential (GAP), Neural Network Potential (NNP), and Deep Potential (DP),^928−931^ those are rarely suitable for H-containing metallic systems. The first ML potential for metal–H system focusing on HE was developed within the framework of GAP by Davidson et al.^932^ In their work, a grand canonical approach was established to comprehensively model the phenomenon of H trapping at vacancies in α-Fe. Leveraging the GAP Fe-H binary potential and employing statistical mechanical calculations, Davidson et al.^932^ determined the occupancy of H atoms trapped in vacancies, elucidating the dependence on temperature and H concentration. In contrast to prior assumptions, the investigation revealed that vacancy traps exhibit sub-saturation occupancy under industrially relevant conditions. This novel finding challenged conventional understanding and had significant implications for the characterization of H behavior in α-Fe. However, this GAP potential is only a purpose-made potential that does not consider Fe–Fe interaction and therefore cannot be applied for studying more complex cases involving multiple defects.

To conquer this issue, Meng et al.^933^ adopted the Neural Network framework to construct a general and versatile ML potential tailored for Fe–H binary system. This ML potential accurately reproduced the interactions between H and diverse defects in α-iron. They further used this ML potential to systematically study the critical events that is related to HE (e.g., H charging and discharging, H diffusion, trapping and desorption at defects, as well as H-assisted cracking at GBs). This general-purpose ML potential provided an alternative approach for gaining a better understanding of Fe–H interactions with the accuracy of DFT calculations and the efficiency of semi-empirical interatomic potentials. Similarly, Zhang et al.^934^ utilized Neural Network to construct an ML potential for Fe–H binary system. They adopted a single-atom Neural Network in which the atomic energies in *ab initio* calculation rather than total energy of system were fully trained. Based on this ML potential, the fracture properties of Fe–H system were examined, unveiling that an elevated H concentration ahead of the crack tip significantly enhances crack propagation, with the magnitude of enhancement contingent upon the specific GB type. These findings underscore the necessity for reliable and precise ML potential to gain deep insights into H’s impact on crack propagation in α-Fe.

Except for Fe–H binary system, there exist ML potentials for other metallic materials in the presence of H. For instance, Kimizuka et al.^935^ developed an ML potential for Pd-H system within the framework of Neural Network. Based on this ML potential, they delved into the pivotal role of nuclear quantum effects (NQEs) on the diffusion of H isotopes within palladium. With the help of path integral techniques, Kimizuka et al.^935^ accurately forecast H diffusivity in protium, deuterium, and tritium in palladium across a broad temperature range, which shed light on the significance of NQEs in governing the diffusion of H isotopes and provided valuable insights into the temperature-dependent activation free energies associated with H-isotope migration.

More recently, Kwon et al.^298^ employed MTP and path-integral MD simulations to systematically study H diffusion in three *bcc* metals (Nb, Fe, and W). Notably, the calculated diffusion coefficients and the observed isotope effects exhibited excellent agreement with experimental data within a realistic temperature range, indicating that MTP is capable of characterizing H diffusion in *bcc* metals.

**Figure 59:**
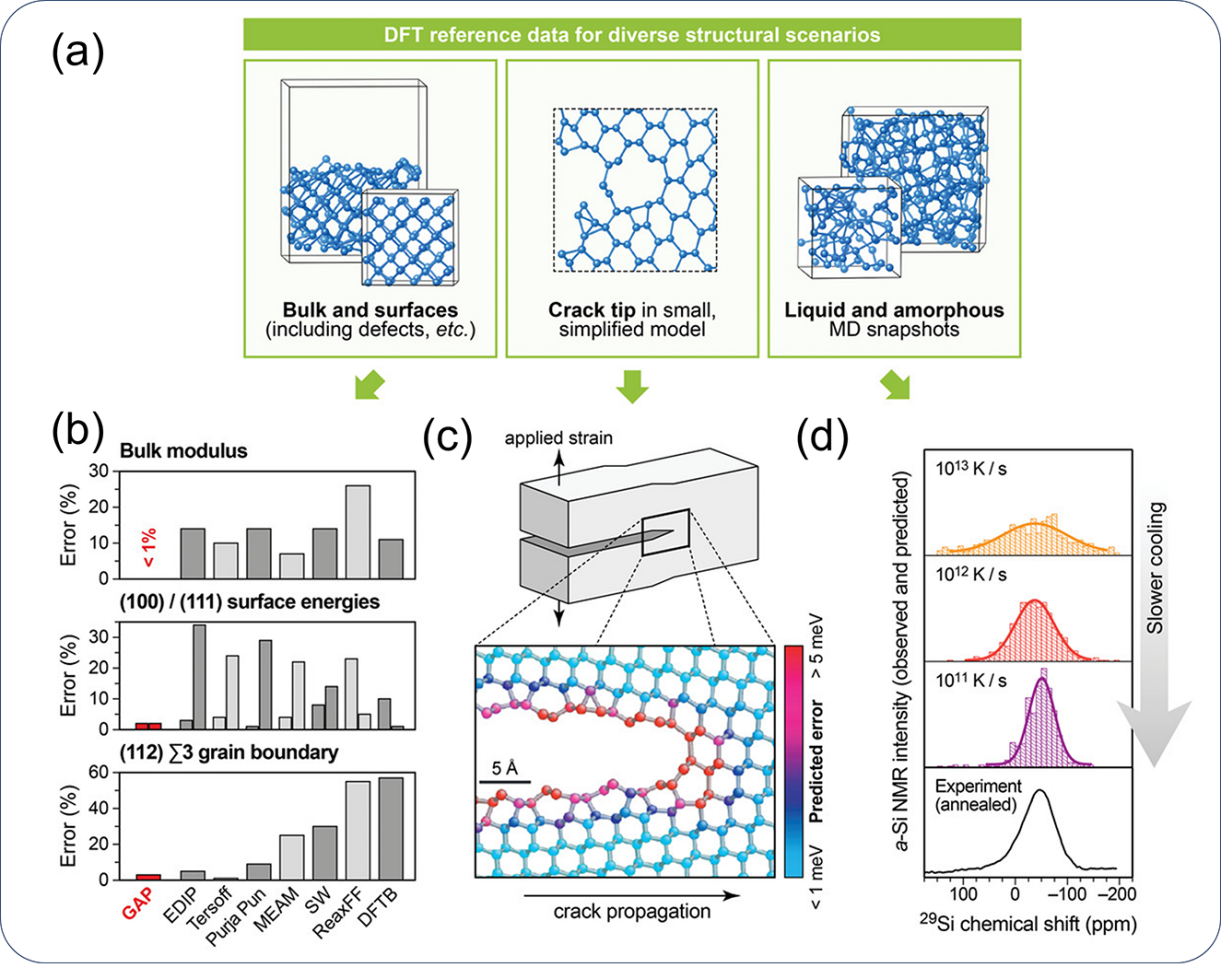
Modeling and simulation of various properties using ML potential. (a) DFT reference data obtained with diverse atomic configurations. (b) The prediction of bulk modulus via various interatomic potentials. (c) Simulation of crack propagation using ML potential. (d) Simulation of structure evolution using ML potential. Reprinted with permission from ref (924). Copyright 2019 Wiley.

The development of ML potentials significantly enhances the precision and effectiveness of large-scale MD simulations. However, careful attention must be given to the entire process of developing these potentials, particularly during the generation of datasets and benchmarking the MD simulations. It is well acknowledged that the choice of datasets and the corresponding data quality directly determine the reliability and the scope of applications of a trained ML potential. Given the vast dimensionality of the configurational space of a material, it is essential to identify the most representative configurations for the intended study and potential applications (see Figure 59). For instance, the ML potential trained with the database involving only H–surface configurations cannot successfully describe the behavior of H–dislocation interaction that is not within the training dataset. Furthermore, in dataset generation, consistent convergence criteria for energy and force in DFT calculations are vital to maintain uniform reference states across all examined configurations. Any discrepancy in DFT calculation setups can introduce artifacts into the ML potential, which should be cautiously avoided.

Another important aspect for the reliable application of interatomic potentials encompassing semi-empirical and ML potentials are the benchmarking and validation. For example, many interatomic potentials have been developed for Ni–H and Fe–H binary systems. However, these potentials are not universally applicable to all HE studies. Tehranchi et al.^936^ modified the original Ni–H EAM potential established by Angelo et al.^360^ using the binding energies of H to various GB trapping sites that were atomistically calibrated by Di stenfano et al. and Zhou et al..^329,923^ Remarkably, MD simulations with this potential successfully predicted the GB binding energies and misfit volume, suitable for investigating GB segregation and the interaction between H and crack tip stress fields. However, studying other defects such as H–dislocation interaction with the same potential might be inaccurate due to the lack of rigorous benchmarking. With respect to Fe–H system, Song et al.^47^ developed an EAM potential based on the one introduced by Ramasubramaniam et al.^116^ This potential eliminated the issue of fictitious H aggregation at high H concentrations and was able to accurately describe basic properties such as surface energy, diffusion barrier and FeH hydride formation energy. But this potential cannot correctly capture the phase transformation between *fcc* and *bcc* phases and therefore cannot be reliably used for the investigation of austenite–martensite interfaces. Another issue with this potential is the incorrect description of the energetics of screw dislocations, the authors explicitly pointed this out and therefore did not adopt the potential to simulate screw dislocations in that MD study.

As a brief summary, HE presents a complex material challenge, intricately involving various microstructural defects and interactions within multicomponent systems, such as alloys and H. Traditional semi-empirical interatomic potentials often fail to accurately depict the interactions with multiple defects or to correctly describe multicomponent interactions. In contrast, ML potentials, trained with datasets that include a wide variety of configurations and elements, have shown significant potential to overcome these limitations. As such, ML has emerged as a transformative tool in addressing HE. From ML-based predictive modeling to ML interatomic potentials, it provides a comprehensive toolkit that enhances understanding of HE and strengthens predictive capabilities. The synergy between ML-driven optimization algorithms and materials informatics facilitates efficient exploration of vast compositional spaces, potentially leading to the discovery of novel alloy compositions that exhibit superior resistance to H-rich environments.

## 4 Closing Remarks

In synthesizing the broad knowledge base on HE, including its precursors, mechanisms, and consequences, along with addressing the pervasive challenges and unanswered questions in HE research, we distill our final thoughts into three segments.

Overarching Statements on HE. We reflect on the broad landscape of HE research, acknowledging the considerable advancements and the intricate factors that govern HE. This segment underscores the challenges involved in mapping H within materials, the complexity of HE mechanisms, and the extensive variety of engineering alloys impacted by HE.

Immediate Research Endeavors. We pinpoint specific, urgent research topics that are primed for immediate exploration. These include deploying innovative experimental methods to measure and map H within materials, identifying the early onset of local failure events, and clarifying the relationship between H, plasticity, and fracture. Moreover, we emphasize the need to develop more sophisticated models for H diffusion and fracture simulation to enhance predictive accuracy.

Next-Generation Solutions. We highlight research directions that, through dedicated and interdisciplinary efforts, are expected to yield groundbreaking solutions to HE. This encompasses the fusion of advanced computational models and experimental data to bolster predictive capabilities, crafting H-resistant materials via innovative alloying and surface modification techniques, adopting cutting-edge technologies such as artificial intelligence in HE research, and contributing to the standardization informed by HE research findings.

These categories offer a structured overview of the current state and future directions of HE research, aiming to spur further investigation and innovation in understanding and combating this complex phenomenon.

### 4.1 HE as a Conspicuous Material Challenge

Despite the extensive history and substantial endeavors devoted to HE, it persists as a material challenge threatening the structural integrity of H energy infrastructure to date. The knowledge accumulated, albeit significant, facilitates a more qualitative, often binary comprehension of HE phenomena and falls short of enabling quantitative predictions of HE.

HE must be recognized as a vital failure mode in addition to the conventional ones, particularly in the context of designing and assessing H energy systems. Pinpointing the operational conditions that could lead to HE is essential to maintaining the durability and reliability of H energy infrastructure. Take the H transport pipelines for instance, the negative impact of HE can be reduced by adjusting the H content, such as mixing it with natural gas. The success of this approach depends on various factors including the specific mixing ratios, material characteristics, operating environments, and system design.

Understanding the interfacial phenomena associated with the entry of H, in particular the dissociative adsorption of H, is key to quantifying H uptake and subsequent transport within a material. This needs to be analyzed with a sophisticated electro-chemo-mechanical framework, with an adequate account of surface chemistry, morphology and stresses. While breakthrough is being made experimentally in probing residential sites of H, the reciprocal progress in modeling has not yet reached its full potential. Interdisciplinary, multi-physics, and multiscale approaches are necessary for accurate assessment of H distribution.

The H charging condition, such as electrochemical versus gas phase and *ex situ* versus *in situ*, profoundly influences HE properties. The fracture mode and the underlying HE mechanism can vary significantly with the charging condition applied in the experiment. It is crucial to correlate specific HE mechanisms to the H charging conditions. For engineering transferability, identifying the practical source of H and applying corresponding laboratory H charging conditions is necessary. While efforts are made to render laboratory H charging methods closely resemble real-life conditions, it is sometimes unavoidable to employ intentionally harsh charging conditions in the lab to expedite the process. These aggressive testing conditions can lead to phenomena not typically observed in actual environments, necessitating a careful interpretation of lab results in understanding HE in engineering structures.

Multiple HE mechanisms have been recognized over past decades. It is a general consensus that no single HE mechanism is universally applicable to all HE phenomena, as they often manifest concurrently. Critical analysis of the advantages and disadvantages of each mechanism, along with a precise demarcation of their operational boundaries, is necessary. Quantitative mapping of individual mechanisms with specific material types, microstructural characteristics, and H content is crucial. Such detailed mapping enables the predictive modeling of HE, facilitates the engineering of microstructures that resist HE, and improves the accuracy of structural assessment of engineering components for green H applications.

Steels and nickel alloys are primary categories of metallic materials on which HE research has been extensively conducted so far. This predominance stems from their widespread application in the oil and gas industry, where HE is a well-known critical threat. Caution needs to be taken when transferring the existing knowledge on HE properties of these materials to analyze failure in green H applications. For example, H is charged through electrochemical processes in oil and gas applications but is mainly from high pressure H_2_ gas in the new applications. This highlights the importance of correlating the two types of H charging methods.

The advent of green H applications necessitates a comprehensive investigation into HE in a broader spectrum of engineering alloys, including aluminum alloys and HEAs, which are directly exposed to H. These alloys are among the top candidates for the storage and transport of green H, due to their favorable strength-to-weight ratio and low temperature performance. Existing knowledge on HE needs to be carefully examined in these materials while keeping an open mind to the potential discovery of new mechanisms underlying HE.

### 4.2 Examples of Immediate Research Topics

Future research should focus on unraveling the complexities of H ingress in materials, specifically targeting the interfacial adsorption phenomena and the exact locations of H absorbed into the material. With the accurate mapping of H within the material and combining advanced microstructural characterization techniques, the precise origins and mechanisms of local failure initiation need further elucidation. A detailed understanding of these aspects will enable the strategic manipulation of material interactions to design microstructures that resist H-induced fracture. Simultaneously, there is a need for the establishment of reliable testing methodologies that can bridge the gap between laboratory experiments and real-world conditions, using parameters that are transferable and relevant to the actual service environments. In parallel, the development of robust predictive models that effectively capture H-induced changes in microstructure and the effects of various loading conditions must be put on the agenda. This is vital for accurately assessing the structural integrity and predicting the service life of H-affected energy systems, ensuring safety and efficiency in their application. The following are key research topics requiring immediate attention.

#### 4.2.1 Interfacial Phenomena Related to H Uptake

Compared to bulk diffusion, H–metal surface interaction, as the initial step in H entry, is less explored. To accurately characterize H adsorption, advanced experimental techniques are requisite. Further, a sophisticated electro-chemo-mechanical method should be devised, incorporating the influence of surface morphology and stress state. A clear distinction between H uptake from the gaseous phase and electrochemical charging is imperative.

Owing to the interdisciplinary nature of the problem, it is crucial to monitor progress in related fields such as thin film technology, solid-state H storage research, and battery studies. Notably, advanced experimental and computational tools employed in characterizing H uptake in metal hydride-based H storage materials and describing electrochemical processes in batteries may offer valuable insights for addressing interfacial phenomena in HE research.

#### 4.2.2 Improved Accuracy of H Mapping

The importance of accurate H mapping can never be overestimated. Accurate delineation of H accommodation is critical to identify the predominant HE mechanism and ascertain the locus of fracture initiation. Traditional methodologies like electrochemical permeation and TDS have been instrumental in characterizing H diffusion and trapping globally. However, the advent of high-resolution local techniques, notably SKPFM and APT, is anticipated to significantly augment future HE research. The SKPFM technique allows for the interrogation of H signal at phase boundaries and GBs, with recent studies demonstrating its utility in examining H transport by mobile dislocations.^937^ The cryo-APT technique enables the direct observation of individual H atoms at various trapping sites such as dislocations, GBs, and precipitates.^10,174^ Notably, Zhao et al. combined cryo-APT with cryo-plasma focused-ion beam (PFIB) specimen preparation to mitigate characterization artifacts by preventing H introduction during sample preparation.^318^

These enabling techniques are expected to facilitate unraveling several longstanding queries in HE research. One primary inquiry is about the specific location of H atoms post-trapping at a precipitate: whether inside the precipitate, at the interface, or in the matrix influenced by the precipitate’s stress field. Unraveling this will significantly inform precipitate engineering strategies aimed at augmenting HE resistance. Additionally, understanding the role of GBs as either H traps or rapid diffusion pathways, and correlating this behavior with the type of GB, is fundamental. This knowledge is crucial to reconcile the discrepancy often observed between the theoretically calculated H diffusion depth and the experimentally measured crack length in *fcc* materials. Furthermore, comprehending whether dislocations transport H atoms, and the kinetics when these H-transporting dislocations interact with precipitates or GBs, is vital. Experimentally quantifying these kinetics in specimens under mechanical loading conditions would constitute a significant advancement in the field, which will significantly contribute to the discourse on HE mechanism predominance in specific materials, thereby guiding targeted mitigation strategies.

#### 4.2.3 *In Situ* and Operando Characterization of Local Fracture Initiation in Bulk Material

It is widely acknowledged that H–dislocation interactions are fundamental in HE mechanisms. Yet, a direct correlation between HELP and fracture initiation remains elusive. Conventionally, the final phase of H-induced fracture is ascribed to decohesion, affecting GBs, phase boundaries, or interfaces at precipitates, reflecting a stress-based fracture paradigm. Alternatively, HELP can potentially lead to material separation through severe strain localization or plastic instability, a theory posited two decades ago. Recent findings demonstrate H’s role in promoting dislocation substructure formation in iron, with fracture initiation occurring at these locales due to pronounced local strain partitioning. These observations, however, are derived from post-mortem analyses. Despite the insights from *in situ* TEM studies on nano-scaled specimens regarding H–dislocation interactions, they fall short in capturing dislocation substructure formation or fracture in real-time. Experimentally tracing the kinetics of dislocation substructure development or plasticity evolution under H conditions in bulk materials remains a challenge.

Operando neutron and synchrotron diffraction and imaging, with their high sensitivity to H and strong penetrating capability, offer promising avenues to concurrently observe H, strain, and H-induced fracture in metals. These methods are poised to elucidate the evolution of plasticity, strain localization, and the inception of fracture in bulk materials, thereby addressing pivotal questions about the initiation sites of H-induced fractures and the potential of HELP in triggering fracture without microstructural interfaces. Additionally, the technique can be adopted to accurately map residual stresses and probe their impact on HE, which is an important aspect for both monotonic and cyclic loading scenarios.

#### 4.2.4 Multiscale and Predictive Modeling

Computational modeling of HE remains largely qualitative. The considerable advancement in experimental techniques has provided extensive data crucial for identifying plausible HE mechanisms in specific materials, including accurate H positioning and fracture paths. These developments have set the stage for creating mechanism-based predictive models of HE. The escalating demand for green H technologies underscores the need for new materials with enhanced HE resistance. Consequently, more effective material design and engineering are imperative. Predictive modeling serves as an essential instrument in this endeavor.

H–dislocation interaction is central to a wide range of HE phenomena, originating at the atomistic level and affecting plasticity properties at the continuum scale. A multiscale model that integrates these length scales with minimal information loss is vital. Combining existing numerical techniques into a multiscale framework appears promising, involving MD, DDD, and CPFE modeling. The primary challenge lies in effectively calibrating crystal plasticity model parameters with DDD simulations. While optimistic about this approach, alternative solutions are considered, such as super large-scale MD simulations potentially supplanting DDD, and continuum dislocation dynamics possibly replacing CPFE. Initially, models will assume dislocations and GBs as the sole defect types, subsequently incorporating other material defects like precipitates and phase boundaries, which is complex. Additionally, accurately describing H redistribution and integrating it with the multiscale model presents another critical challenge. Immediate attention is necessary in these areas, with prototype models expected soon. Nonetheless, realizing a first-principles-based multiscale modeling framework that accurately represents real engineering alloys remains a long-term goal. Such a modeling framework will be instrumental for interpreting HE phenomena and developing innovative HE mitigation concepts. For instance, recent studies have shown that H could benefit the mechanical properties of austenitic stainless steel, which is similar to the effect of an alloying element. This finding is counter-intuitive and deserves further investigation, where a multiscale modeling framework can be an enabling tool.

It is crucial to equally focus on developing models that can handle the combined effects of multiple HE mechanisms to simulate fracture accurately and match experimental fracture morphology. A few mechanism-based models have emerged as strong candidates for incorporating several HE mechanisms. The weakest-link statistical model has been effective in describing decohesion at GB carbides, taking into account the local stress and H accumulation associated with dislocation pile-ups, and aligning with plasticity-mediated decohesion.^467^ The phase field model provides a versatile means to simulate crack progression and plasticity, applied to both HEDE and HELP mechanisms.^687,714^ The unified mechanics theory, recently extended with H considerations, offers a way to quantify material degradation by considering entropy production due to the effects of H on micro-plasticity and decohesion,^718^ which is in line with the concept of synergistic action of HEDE and HELP mechanisms. Moreover, void-based predictive models, including unit cell and Gurson-type models, link plasticity, material separation, and vacancies and stand out as a platform to incorporate several major HE mechanisms, i.e., HELP, HEDE, and HESIV mechanisms.^20,710,712,723^ It is noted that these models represent a promising but not exhaustive list.

Accurate determination of model parameters is critical for successful predictive modeling. While first principles calculations and multiscale modeling offer valuable insights, experimental calibration is often the most reliable method for determining model parameters. However, it is not uncommon for sophisticated models to have numerous parameters that exceed the calibration capabilities of existing experimental methods, making the models impractical and limiting their applicability. Therefore, it is important for model developers to understand existing experimental techniques as thoroughly as the experimentalists themselves, and to carefully select their model platforms and parameters to facilitate easier experimental calibration. If current experimental methods are inadequate for calibrating new models, modelers should propose new testing methods and protocols to enable calibration of their models.

#### 4.2.5 Engineering Transferability and Scalability

Theories, predictive assessment tools, and mitigation strategies for HE, validated against laboratory experiments, can be ineffective under real-world conditions with more complex environmental and material variables. To enhance the scalability of HE research outcomes amid the growing green H applications, urgent improvements in engineering transferability from laboratory to engineering practice and across various engineering scenarios are necessary. This necessitates a rigorous alignment to realistic operating conditions in critical stages of HE research, encompassing H charging, mechanical testing, and model development.

Engineering components for H transport and storage in green H applications are often subjected to gaseous H. While electro-chemical H charging is less relevant, it remains a safe and economic laboratory charging method for research and routine testing by industries. Electro-chemical H charging is essentially a mimic of real-world gaseous H conditions. Hence, determining the correlation between these two charging conditions is essential. The primary distinction is the surface adsorption of H, either as atoms or ions. Assuming similarities in H diffusion and trapping post-absorption, the criterion for correlating these two charging methods might be based on matching of sub-surface H concentrations. Continued research aims to establish a reliable correlation soon.^118^

Discrepancies in mechanical testing between laboratory and engineering applications often arise from different geometric constraints; engineering components possess more complex constraints than laboratory specimens. Selection and design of proper mechanical tests are crucial, with fracture toughness tests often offering higher engineering transferability than tensile tests. The design of loading type and history should accurately represent the engineering application intended.

In predictive model development, the influence of geometric constraints and loading paths need to be considered. Physics-based models, like Gurson-type models, typically account better for these factors, offering enhanced engineering transferability over empirical models based on simple stress or strain failure criteria. Prior to application, models must be rigorously verified against established benchmarks.

#### 4.2.6 Adaption to Emerging Green H Applications

The focus and content of HE research are poised for adaptation in response to green H applications. Below are examples of imminent or potential adaptions to be made.

Temperature dependence of HE properties: addressing the structural safety requirements for cryogenic H storage necessitates comprehensive evaluation of engineering alloys in liquid H. H-powered jet engine applications mandate the assessment of H-induced fracture at elevated temperatures. Investigations may explore the influence of H on creep properties, potentially unveiling new insights into its effect on dislocation climb, the underlying mechanism of creep.

Novel engineering alloys and material manufacturing approaches: while studies on steels and nickel alloys progress, HE research will be increasingly focused on other advanced alloys such as aluminum alloys and HEAs. The interest in HE research of AMed materials has been escalating. Investigations into new materials, combined with enhanced control over manufacturing processes, are expected to yield novel HE mitigation strategies.

H-induced damage in solid-state H storage materials: at present, these materials are predominantly studied in powder form to optimize surface-to-volume ratio for enhanced H absorption/desorption kinetics and capacity. However, as these materials transition into practical applications, H-induced damage concerns, particularly impacting charging/discharging cyclability and load-bearing capacity, will become pronounced. This is especially critical when these materials are employed simultaneously as structural components, such as in conformal H storage devices. Consequently, extensive research into understanding and mitigating H-induced damage in these materials will become necessary.

The aforementioned areas represent a promising yet non-exhaustive list of potential research trends in HE. These domains are anticipated to substantially augment the existing knowledge base of HE and have the potential to address enduring questions and challenges in the field.

### 4.3 Paradigms of Long-Term Research Endeavors in HE

In the long-term perspective, HE research must support green H applications by enabling precise structural assessment of components for H storage and transport, promoting standardization of related products and test methods, and developing novel materials with resistance to HE. Success in these domains relies on the synergistic integration of emergent technologies and paradigms such as big data science, ML, materials genome, and digital twin concepts.

#### 4.3.1 Database of Materials and HE Properties

A critical gap in HE knowledge is the lack of a definitive, quantitative connection between HE mechanisms and variables like material type, microstructure, and H exposure/loading conditions. Establishing a database by analyzing extensive material property data, experimental outcomes, and simulations is essential for identifying patterns and correlations pertinent to HE. Continual updates and maintenance of this database are necessary due to the rapidly growing influx of data, necessitating the use of big data science and ML.

#### 4.3.2 ML-Based and Data-Driven HE Research

With the rapid progress in AI and robotics, high-throughput approaches in computing, mechanical testing, and material characterization are set to revolutionize HE research. Perhaps the only question is how fast this transformation will happen; it may actually emerge as an immediate result rather than a distant research objective, given the exponential expansion of AI technology. By utilizing these advanced techniques, researchers will be able to quickly and efficiently test a vast number of materials for HE susceptibility. This approach will significantly enhance the HE database, providing deeper insights and a more extensive understanding of how different materials react to HE.

ML-based tools will greatly facilitate the application of materials genome concept in HE study. This integrated approach involves understanding material attributes and potentials by fusing computational tools, experimental data, and digital databases. Establishing an HE database and employing high-throughput screening are critical in fostering the development of a materials genome specific to HE, which will greatly accelerate the discovery, design, and deployment of new materials for green H applications through a shared, collaborative effort.

Future developments might see the emergence of a digital twin, encapsulating HE considerations for H storage and transport. Such a twin would draw upon the HE database and predictive models, necessitating real-time monitoring and a feedback loop for continuous environmental and operational data assimilation. Crafting a digital twin considering HE is a multifaceted venture, demanding a comprehensive integration of material science, data technology, and domain expertise to assure precise predictions, informed decision-making, and heightened safety and reliability of H-exposed products.

#### 4.3.3 Standardization

Prolonged advancements in HE research will significantly aid the standardization pertinent to green H applications. There is a pressing need for standardization in emerging sectors like H-powered maritime and aviation. A better understanding of HE mechanisms will facilitate the establishment of reproducible testing methods. Efforts should be directed to standards for both screening procedures and to fracture mechanics-based characterization.

In summation, HE has historically been a conspicuous material challenge for engineering and continues to be so in the realm of green H applications. Vast knowledge has been accumulated regarding HE in the past decades, which with caution, can be extrapolated to the novel contexts. The surging interest in H, coupled with the advent of cutting-edge technologies, presents unprecedented opportunities to elucidate long-standing HE queries and to depict a holistic picture of this scientific domain.

## Abbreviations

AI: artificial intelligence
AIDE: adsorption-induced dislocation emission
AM: additive manufacturing
AMed: additively manufactured
APT: atom probe tomography
*bcc*: body-centered cubic
cryo-APT: APT at a cryogenic condition
DDD: discrete dislocation dynamics
DED: direct energy deposition
DFT: density functional theory
*fcc*: face-centered cubic
FCG: fatigue crack growth
FR source: Frank-Read source
GB: grain boundary
GCMC: Grand Canonical Monte Carlo
H: hydrogen
*hcp*: hexagonal close-packed
HE: hydrogen embrittlement
HEA: high entropy alloys
HEDE: hydrogen enhanced decohesion
HELP: hydrogen enhanced localized plasticity
HESIV: hydrogen-enhanced strain-induced vacancies
HIP: hot isostatic pressing
IG: intergranular
L-PBF: Laser Powder Bed Fusion
MD: molecular dynamics
MS: molecular statics
ML: machine learning
MPB: melt pool boundary
MVC: microvoid coalescence
PAS: positron annihilation spectroscopy
SEM: scanning electron microscope
SFE: stacking fault energy
SIMS: secondary ion mass spectrometry
SKPFM: scanning kelvin probe force microscopy
SLM: Selective Laser Melting
SSRT test: slow strain rate tensile test
TDS: thermal desorption spectroscopy
TEM: transmission electron microscopy
TG: transgranular
TRIP: transformation-induced plasticity
TWIP: twinning-induced plasticity
VH: vacancy-hydrogen cluster/complex
WAAM: wire arc additive manufacturing
